# Palladium-Catalyzed Asymmetric Carbonylation of Alkynes to Axially Chiral Carbothioate Esters

**DOI:** 10.1021/jacs.5c11864

**Published:** 2025-09-22

**Authors:** Wang Yao, Yang Yuan, Ting Yang, Yong-Gui Zhou, Xiao-Feng Wu

**Affiliations:** † Dalian National Laboratory for Clean Energy, 58279 Dalian Institute of Chemical Physics, Chinese Academy of Sciences, 116023 Dalian, Liaoning, China; ‡ 28392 Leibniz-Institut für Katalyse e.V., 18059 Rostock, Germany

## Abstract

Herein, we report a novel palladium-catalyzed asymmetric hydrothioesterification of alkynes under mild conditions, enabling the precise construction of axially chiral carboxylic thioester derivatives with excellent yields, regioselectivity, *E*/*Z* selectivity, and enantioselectivity. Disulfides and thiosulfonates were applicable, as well.

## Graphical abstract

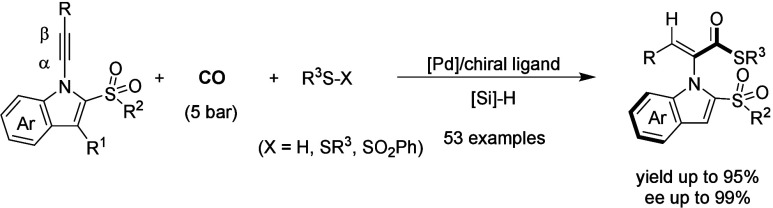

Axially chiral structures represent core scaffolds in diverse natural products,^1^ synthetic materials,^2^ and drug molecules,^3^ while also serving as chiral catalysts^4^ and ligands^5^ in asymmetric synthesis. However, compared to well-established biaryl atropisomers, the development of acyclic axially chiral styrenes has lagged significantly,^6^ primarily due to the unique structural constraints imposed by their olefinic frameworks. Due to their substantially lower rotational energy barrier compared to rigid aromatic systems, constructing these structures requires the incorporation of sterically demanding groups to achieve sufficient conformational stability (Scheme 1A). Current strategies for assembling these frameworks include central-to-axial chirality transfer, asymmetric alkyne functionalization, asymmetric coupling, and enantioselective C–H bond activation.^7^ However, a direct asymmetric carbonylation strategy for constructing these scaffolds has not been reported to date.

**Scheme 1:**
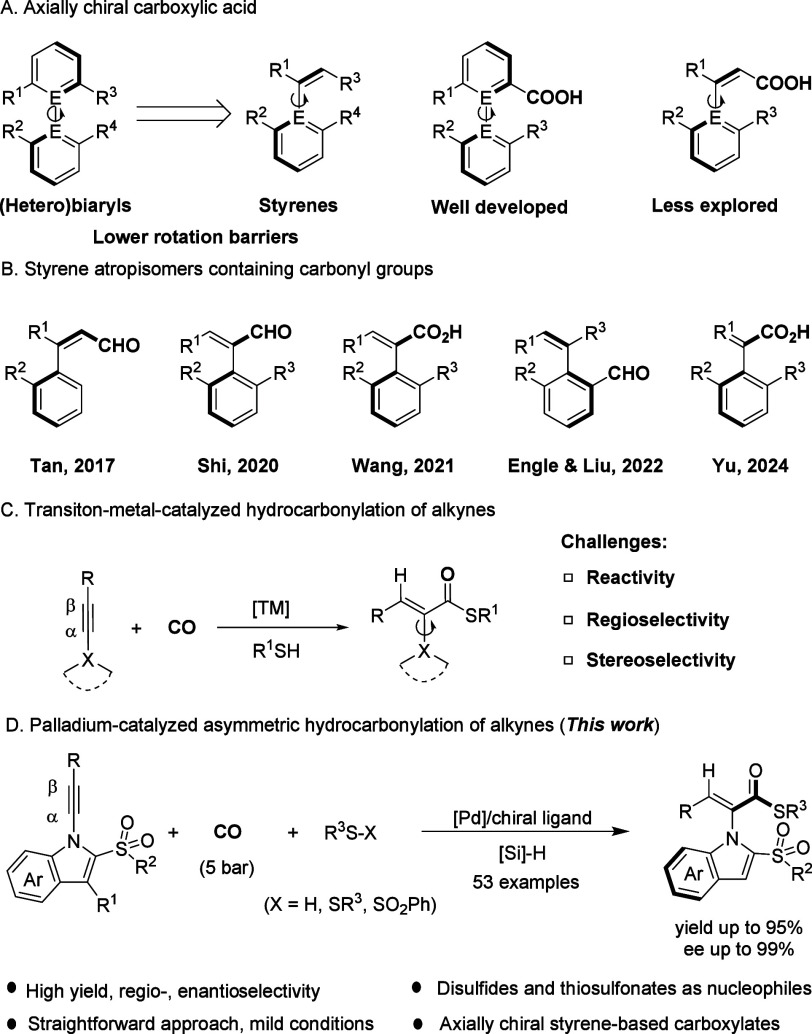
Significance of Atropisomeric Styrene-Based Carbonylated Compounds and Asymmetric Carbonylation of Alkynes

Carbonyl-containing axially chiral styrene atropisomers represent a highly diverse and significant class of compounds, garnering sustained and widespread attention as a novel scaffold. Research groups led by Tan,^8^ Shi,^9^ Wang,^10^ Engle and Liu,^11^ Tanaka,^12^ Song,^13^ and Yu^14^ have all achieved the synthesis of axially chiral styrene frameworks incorporating carbonyl functional groups (Scheme 1B). Notably, the Shi group implemented a transient directing group (TDG)-enabled C–H activation strategy to construct axially chiral styrenes bearing aldehyde groups.^15^ Furthermore, they developed an axially chiral carboxylic acid derived from the enantioselective amidation of thioamides.

Carbonylation reactions have garnered significant attention due to their high atom economy and broad applicability.^16^ Since the pioneering work from Reppe,^17^ impressive progresses have been achieved on transition-metal-catalyzed carbonylation of alkynes.^18^ For the hydroesterification, the alkyne first coordinates with the metal hydride, and the orientation of this coordination dictates the stereo- and regioselectivity of the product. This inherent step significantly complicates the attainment of the selectivity for a single product. Thioester compounds play irreplaceable roles in biological systems, such as acetyl coenzyme A.^19^ Thiocarbonylation of alkynes is an ideal strategy for constructing unsaturated thioesters.^20^ Nevertheless, thiocarbonylation employing thiols as nucleophiles faces challenges, such as their pungent odor and catalyst poisoning (Scheme 1C). Moreover, while recent advances in asymmetric alkyne carbonylation have predominantly enabled intramolecular cyclization to access axially chiral compounds,^21^ the construction of styrene-based atropisomers remains unexplored. Herein, we report a palladium-catalyzed intermolecular hydrothioesterification of alkynes with thiols, disulfides, and thiosulfonates, which enables the precise and efficient generation of axially chiral carboxylic thioesters (Scheme 1D).

We selected 1-alkynylindole **1a** and 4-methylbenzenethiol **2a** as model substrates and employed Pd­(TFA)_2_ and triethylsilane to optimize the reaction conditions (Table 1). Initially, ligand screening was performed in acetonitrile (entries 1–10). The results indicated that bisphosphine ligands generally outperformed monophosphine ligands in terms of both yield and enantioselectivity (entry 10). The functional groups attached to the phosphorus atoms proved to be crucial for enantiocontrol. Smaller substituents failed to provide sufficient steric bulk during the coordination of the Pd–H species to the alkyne, resulting in lower enantioselectivities (entries 1–5). In contrast, ligands featuring bulky substituents significantly enhanced their enantioselectivities (entries 6–9). Among these, ligand **L7** afforded the product **3a** with moderate yield and 92% ee (entry 7). *p*-Toluenesulfonic acid (TsOH) significantly increased the yield, while it caused a drastic decrease in the ee to 40% (entry 11). Diphenylphosphinic acid as an additive provided the product with moderate results (entry 12). We subsequently employed chiral phosphonic acids as additives. Chiral phosphonic acid **A2** was found to better preserve the enantioselectivity of the product compared to racemic phosphonic acid **A1**, while also further improving the yield (entries 13, 14). We then investigated the solvent effect (entries 15–17). The reaction proceeded poorly in THF. In dichloromethane (DCM) and trifluorotoluene, the product was obtained with reduced yield and moderate enantioselectivity. Further reductions in catalyst and ligand loadings were tested. Using 2.5 mol % Pd­(TFA)_2_ and 3 mol % bisphosphine ligand **L7**, the product was still obtained with 92% ee, albeit in reduced yield (entry 18). Shortening the reaction time also led to a decrease in the yield (entry 19). Dropped yield was obtained if we perform the reaction under atmospheric pressure of CO. It is also worth mentioning that noncarbonylation was the main side reaction during the optimization process, which has regioselectivity and stereoselectivity issues.

**Table 1:**
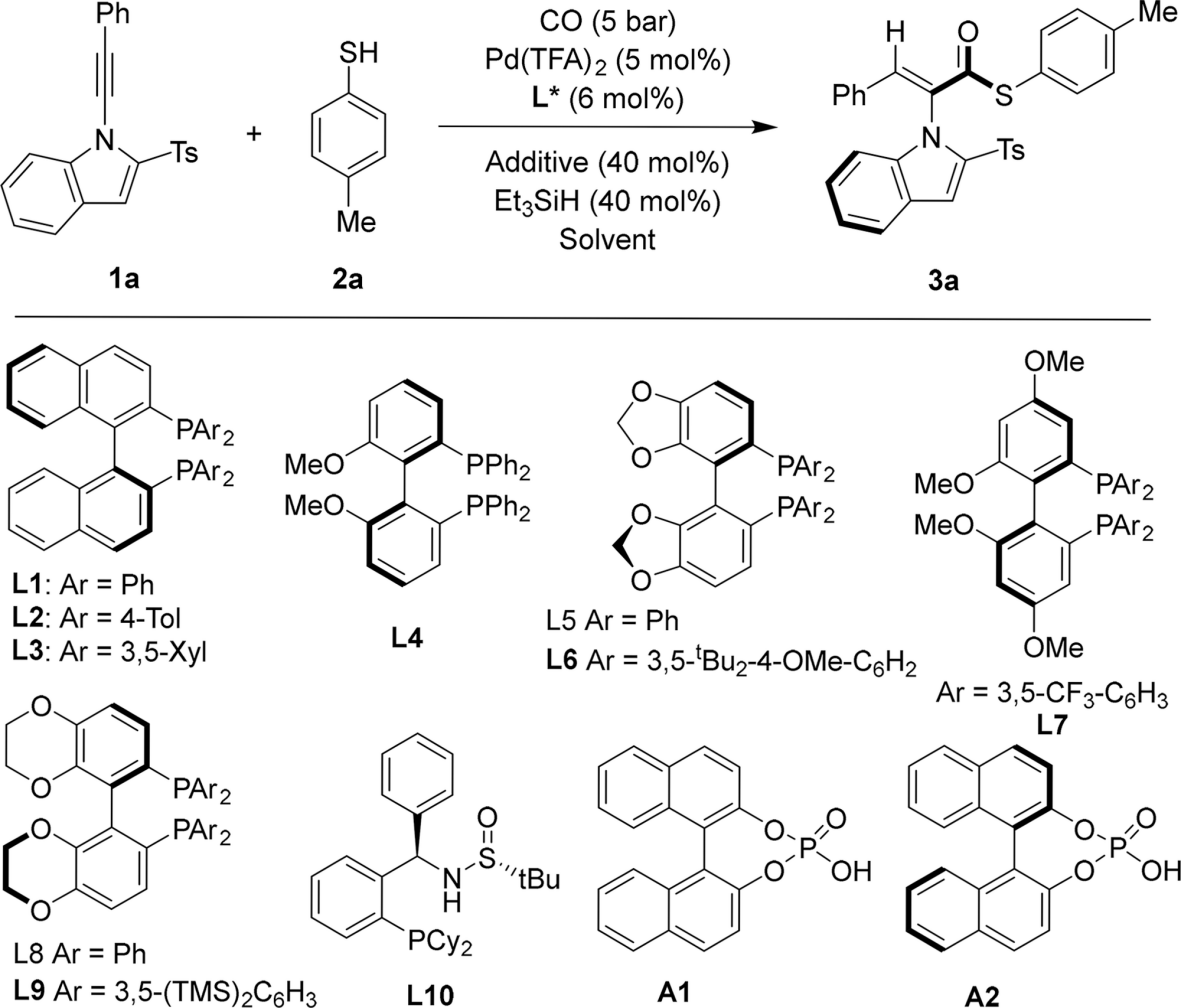
Optimization of the Reaction Conditions^a^

Entry	L*	Additive	Solvent	Yield [%]^b^	Ee [%]^c^
1	**L1**	-	MeCN	75	17
2	**L2**	-	MeCN	70	23
3	**L3**	-	MeCN	66	26
4	**L4**	-	MeCN	80	35
5	**L5**	-	MeCN	72	40
6	**L6**	-	MeCN	77	80
7	**L7**	-	MeCN	70	92
8	**L8**	-	MeCN	73	35
9	**L9**	-	MeCN	32	87
10	**L10**	-	MeCN	10	3
11	**L7**	PTSA·H_2_O	MeCN	94	40
12	**L7**	Ph_2_POOH	MeCN	55	78
13	**L7**	A1	MeCN	95	88
14	**L7**	A2	MeCN	95	92
15	**L7**	A2	DCM	49	89
16	**L7**	A2	THF	-	-
17	**L7**	A2	CF_3_Ph	21	92
18^d^	**L7**	A2	MeCN	76	92
19^d^ ^,^ ^e^	**L7**	A2	MeCN	70	92

a Reaction conditions: **1a** (0.1 mmol), **2a** (0.15 mmol), Pd­(TFA)_2_ (5 mol %), L* (6 mol %), additive (40 mol %), Et_3_SiH (40 mol %), solvent (0.5 mL) under CO (5 bar) at r.t. for 24 h.

b Isolated yields of **3a**.

c Ee value of **3a** was determined by HPLC analysis.

d Pd­(TFA)_2_ (2.5 mol %), **L7** (3 mol %).

e The reaction was performed at r.t. for 12 h.

With the optimized conditions in hand, we first investigated the scope and generality of the indole backbone in 1-alkynylindoles (Scheme 2). Substrates bearing halogen, alkyl, or alkoxy substituents at the 4- (**3b–3d**), 5- (**3e–3g**), or 6-position (**3h–3k**) of the indole ring all underwent the reaction smoothly, affording the axially chiral products in 80–92% yields and 87–96% ee. The absolute configuration of the major enantiomer was unambiguously assigned by single-crystal X-ray diffraction analysis of **3a** (CCDC 2465220).^22^ Interestingly, the C3-indole-derived alkyne also participated efficiently in the reaction, yielding **3l**, albeit with a moderate reduction in yield and ee to 72% and 90%, respectively. The alkyne moiety of 1-alkynylindoles was also examined. A wide range of functional groups on the terminal arylacetylene were well tolerated. Substrates bearing halogen atoms, methyl groups, or trifluoromethyl groups at the *para*-position (**3m**–**3p**), *meta*-position (**3q**–**3s**), or *ortho*-position (**3t**, **3u**) of the phenyl ring all underwent the asymmetric hydrothioesterification reaction smoothly, delivering the corresponding products in 70–95% yields and with 90–99% ee. Heterocyclic substituents such as thiophene were also viable, successfully affording product **3v** in 83% yield with 90% ee. Furthermore, the fused-ring system proved to be a suitable substrate, furnishing product **3w** in 81% yield and 90% ee. It should be noted that alkyl-substituted alkyne was also compatible, although a slight decrease in enantioselectivity was observed, to give chiral product **3x** in 85% yield with 82% ee. It should be noted that the ee decreased dramatically when the Cy group was replaced with an nBu group. Various sulfonyl groups were also compatible with this catalytic system (**3y**–**3aa**), affording products in 88–91% yield with 90–93% ee. However, the alkylsulfonyl-derived product **3ab** exhibited a decrease in enantioselectivity, attributed to the reduced steric bulk of the alkylsulfonyl group, which is also in line with our hypothesis. Under our standard conditions, no desired product was detected when the indole ring was replaced with a pyrrole ring.

**Scheme 2:**
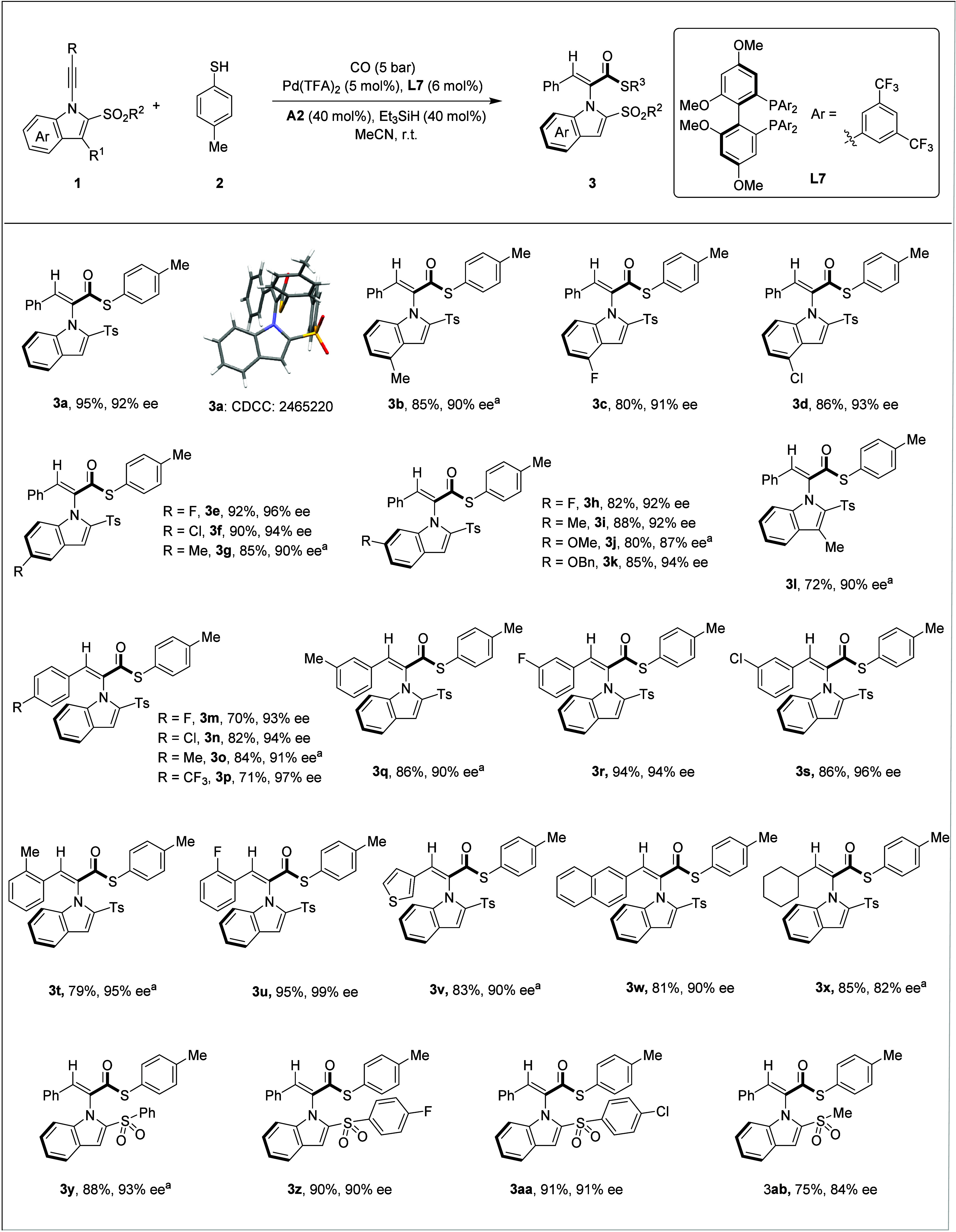
^a^

a Reaction conditions: **1** (0.1 mmol), **2** (0.15 mmol), Pd­(TFA)_2_ (5 mol %), **L7** (6 mol %), **A2** (40 mol %), Et_3_SiH (40 mol %), MeCN (0.5 mL) under CO (5 bar) at r.t. for 24 h. [a] **L6** was used.

After studying the substrates with alkyne skeletons, we expanded the substrate scope by using different thiols as coupling partners (Scheme 3). Substituted thiophenols bearing various electron-poor groups, halogens (**3ac**–**3ae**, **3ai**), trifluoromethyl (**3af**) or electron-rich groups, methyl (**3ah**), and methoxy (**3ag**) at the *meta*-position or *para*-position were well tolerated, affording the corresponding products in 60–72% yields with 91–96% ee without compromising enantioselectivity. Substrates with *ortho*-substituted functional groups also reacted successfully (**3ak**, **3al**), yielding 82–85% yields and 90–93% enantioselectivities. 3,5-Difluorobenzenethiol proved to be a suitable substrate, delivering the axially chiral product **3aj** in 75% yield with 94% ee. Notably, the fused-ring system of 2-naphthalenethiol underwent smooth asymmetric coupling to furnish, in excellent yield and enantioselectivity, product **3am**. The alkyl thiol was compatible under the reaction conditions, providing product **3an** in 72% yield with 90% ee. Furthermore, we introduced core structures of natural products via thiol derivatization. Atropisomeric styrenes incorporating dl-menthol (**3ao**), d-fructopyranose diacetonide (**3ap**), and cholesterol (**3aq**) were obtained in excellent yields with a high diastereoselectivity. Additionally, aniline, phenol, and water as other types of nucleophiles were also tested under our standard condition, but led to no desired product.

**Scheme 3:**
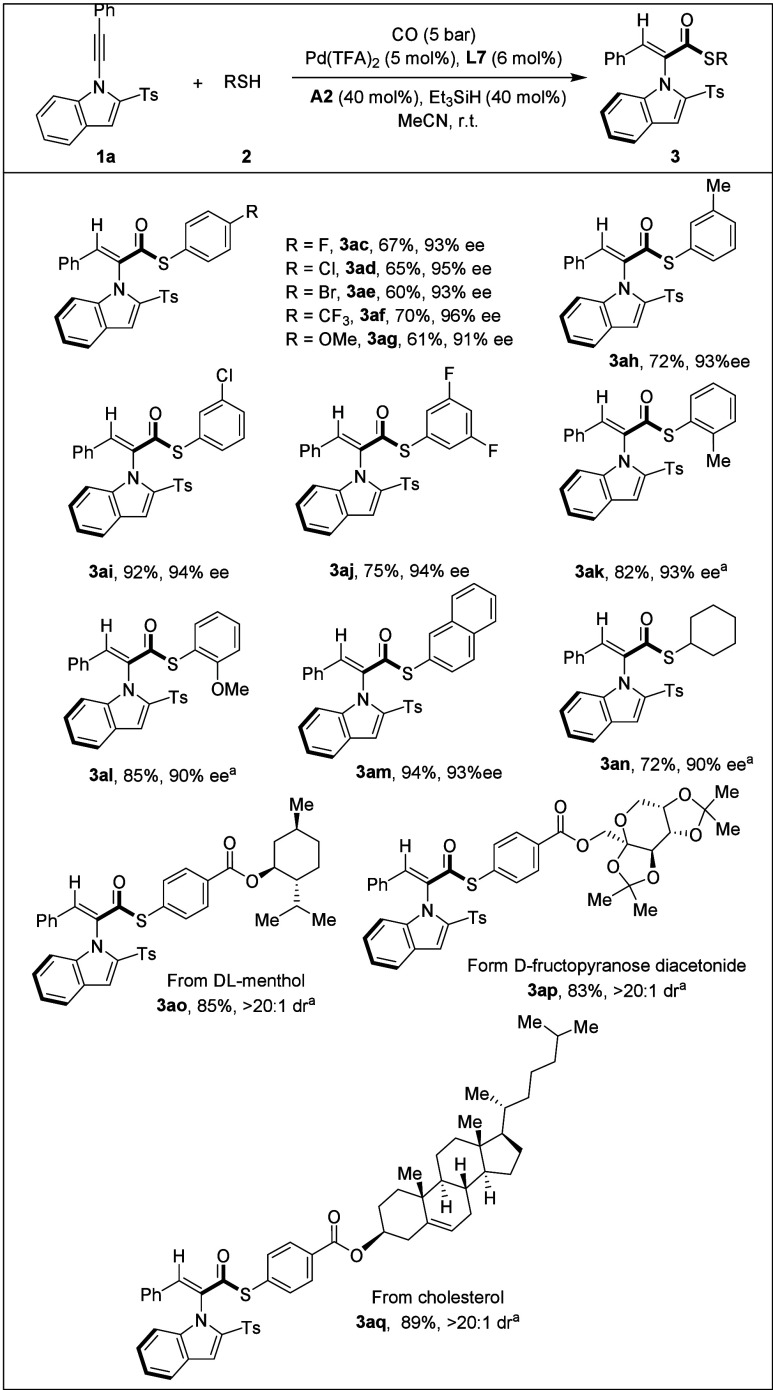
^a^

a Reaction conditions: **1a** (0.1 mmol), **2** (0.15 mmol), Pd­(TFA)_2_ (5 mol %), **L7** (6 mol %), **A2** (40 mol %), Et_3_SiH (40 mol %), MeCN (0.5 mL) under CO (5 bar) at r.t. for 24 h. [a] **L6** was used.

To further broaden the practicality of this reaction, we also developed asymmetric carbonylation reactions of alkynes with disulfides and sulfonyl thioesters (Scheme 4). Since thiols possess a strong offensive odor and create the catalyst poisoning that often causes inconvenience in organic synthesis, disulfides and sulfonyl thioesters can circumvent this issue. In the hydrothioesterification involving disulfides, it was necessary to increase both the catalyst loading and the amount of silane. In contrast, sulfonyl thioesters required only additional equivalents of silane, in which case the metal hydride species was generated by the silane. This approach once again afforded styrene atropisomers in excellent yields of 78–96% and enantioselectivities of 90–92% (**5a**–**5d**, **6a**–**6d**).

**Scheme 4:**
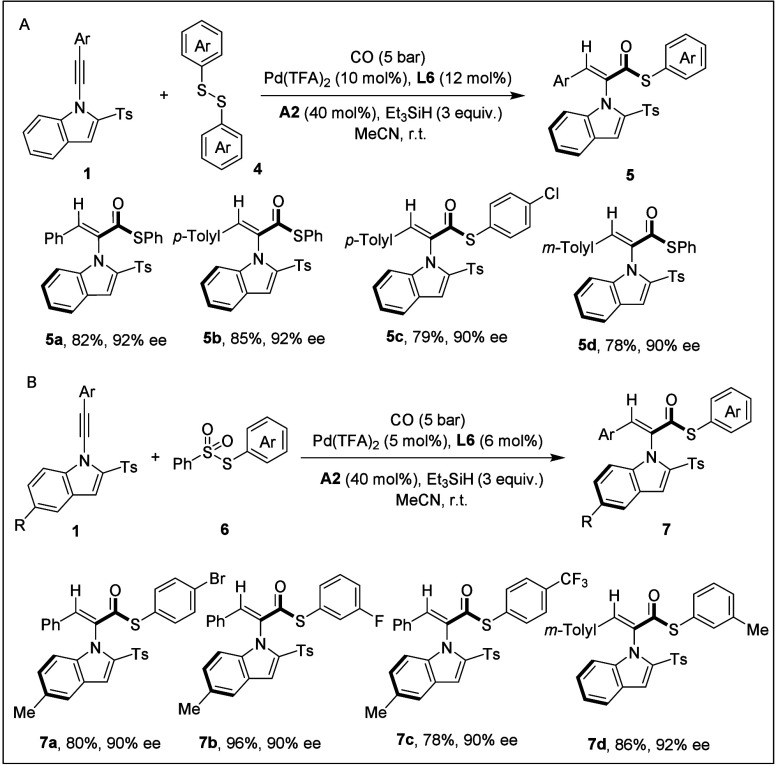
^a^

a Reaction conditions of A: **1** (0.1 mmol), **4** (0.15 mmol), Pd­(TFA)_2_ (10 mol %), **L6** (12 mol %), **A2** (40 mol %), Et_3_SiH (3 equiv), MeCN (1 mL) under CO (5 bar) at r.t. for 24 h. B: **1** (0.1 mmol), **6** (0.15 mmol), Pd­(TFA)_2_ (5 mol %), **L6** (6 mol %), **A2** (40 mol %), Et_3_SiH (3 equiv), MeCN (1 mL) under CO (5 bar) at r.t. for 24 h.

To evaluate the practical utility of this asymmetric hydrothioesterification reaction, we performed a gram-scale reaction of 1-alkynylindole **1a** with 4-methylbenzenethiol **2a**, and the product **3a** was obtained in 95% yield with 92% ee, without compromising either the yield or enantioselectivity. Subsequent hydrolysis of **3a** afforded the axially chiral carboxylic acid **8** in 85% yield with 95% ee. Additionally, the thioester underwent a smooth Fukuyama coupling reaction with phenylboronic acid, furnishing the asymmetric carbonylative arylation product **9** in 81% yield with 80% ee. However, the reaction with aniline and phenol failed, leading to products without enantioselectivity. The stability of the atropisomers proved crucial for further investigations. Consequently, we measured the rotational barriers for **3a**, **3m**, and **3i**. The rotational barrier for **3a** in toluene at 100 °C was determined to be 30.7 kcal/mol. For **3m** and **3i**, the barriers measured in toluene at 120 °C were 31.5 and 30.8 kcal/mol. According to the stability classification system for atropisomers proposed by LaPlante and Edwards, these compounds fall into class-3 atropisomers (Scheme 5).^23^ Their half-lives were also calculated.

**Scheme 5:**
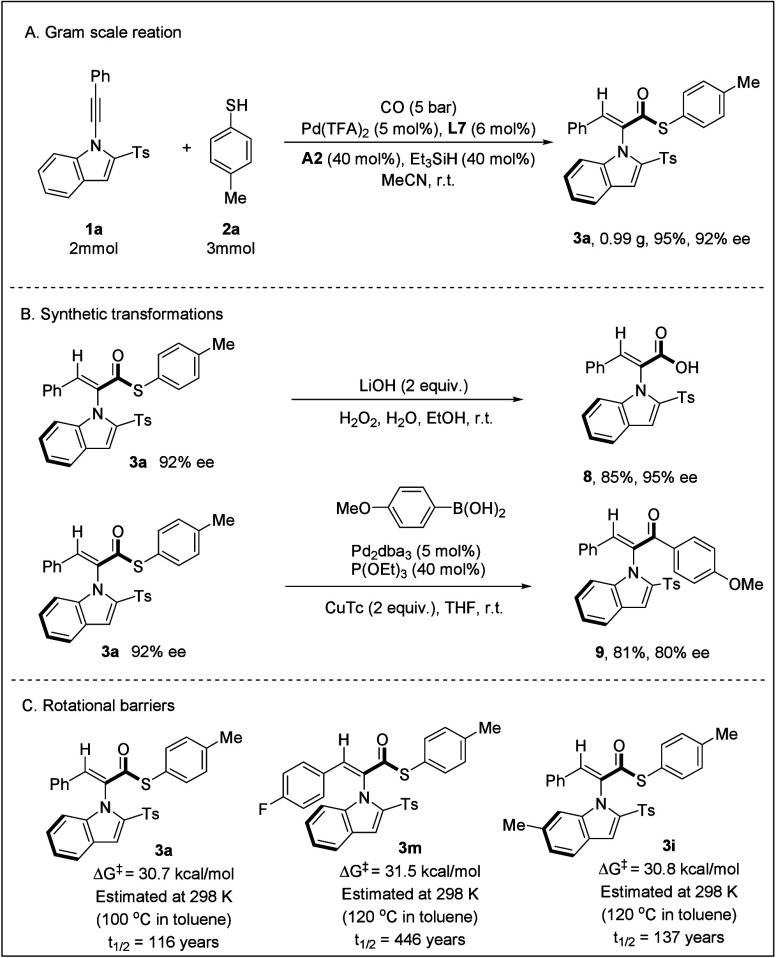
Gram-Scale Reaction and Synthetic Transformations

In our control experiments, the reaction failed to proceed when silane was excluded. When the additive **A2** was omitted and PhSiD_2_ was employed instead of Et_3_SiH, no deuterium incorporation was observed at the β-position of the alkene moiety, indicating that the hydrogen atom at this site does not originate from the silane reductant. However, a deuterated product was detected in the reactions with disulfide and sulfonyl thioester with PhSiD_2_. Subsequently, the reaction was performed using 4-fluorobenzenethiol **2ac-D** as the nucleophile, which showed 25% deuterium incorporation at the β-position of the alkene. These results collectively demonstrate that the hydrogen source for the metal hydride species is the thiophenol reagent (Scheme 6). Silane was the main proton source in the case of reactions with disulfides and sulfonyl thioesters. In all cases, the solvent and trace amounts of moisture in the reaction system could partially be the proton source as well.

**Scheme 6:**
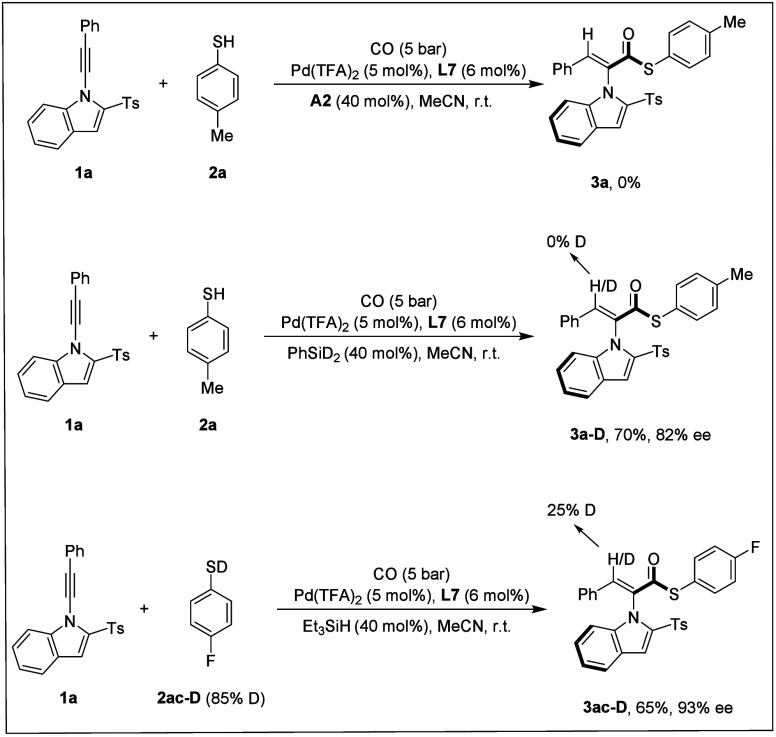
Control Experiments

We proposed a plausible reaction mechanism for this reaction (Scheme 7). Initially, the Pd^II^ precatalyst can be reduced by silane at room temperature to Pd^0^, which then react with acid to give a Pd^II^–H intermediate. The palladium hydride Pd^II^–H species can also be formed from a Pd^II^ precursor in the presence of thiol, silane, and ligand without acid. Then the Pd^II^–H species coordinates to the alkyne substrate followed by insertion to give vinyl-palladium intermediate **I**. Subsequent coordination and insertion of carbon monoxide yield the key acylpalladium intermediate **II**. Finally, nucleophilic attack by the thiophenol on the acylpalladium intermediate **II** affords the desired product **3** and regenerates palladium hydride species for the next catalytic cycle.

**Scheme 7:**
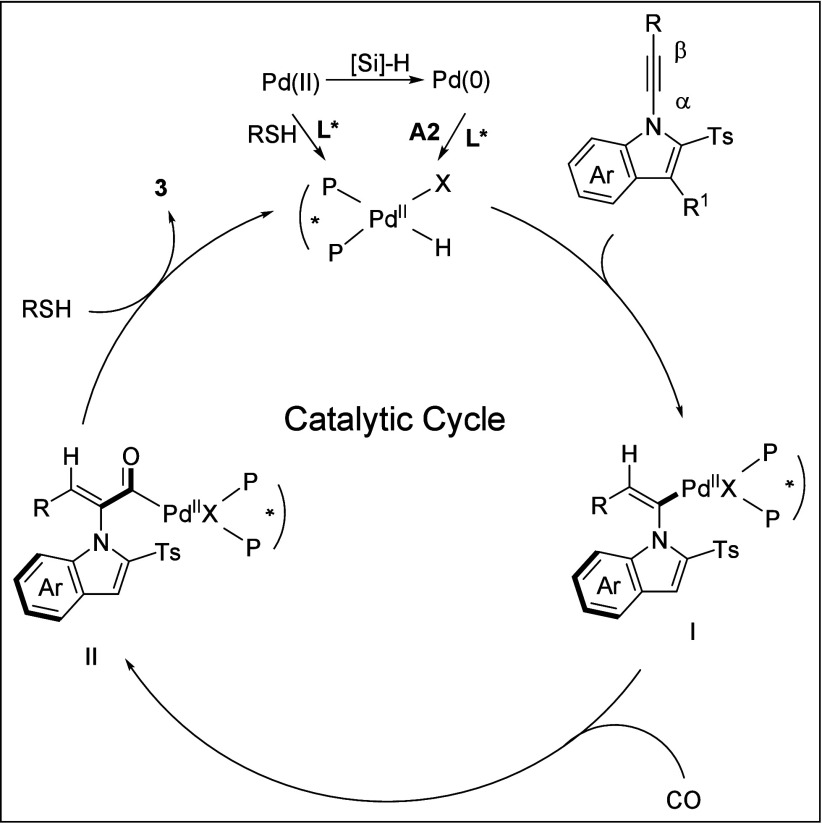
Proposed Catalytic Cycle

In summary, we have developed a simple and efficient method for the palladium-catalyzed asymmetric hydrothioesterification of alkynes with thiols, which enables the construction of a series of axially chiral carbothioate esters under mild conditions with high yield, stereoselectivity, regioselectivity, and enantioselectivity. Besides the success with a gram-scale reaction, the reactions with disulfides and sulfonyl thioesters were also successful.

## Supplementary Material



## Notes

General comments, general procedure, optimization details, analytic data, and NMR spectra (PDF)
