# Supplementary material for: Palladium-Catalyzed Asymmetric Carbonylation of Alkynes to Axially Chiral Carbothioate Esters
Source: J Am Chem Soc. 2025 Sep 22;147(39):35203–10. doi: 10.1021/jacs.5c11864 (PMC12498392; doi:10.1021/jacs.5c11864)
Supplement: Supplementary file 1 [file ja5c11864_si_001.pdf]

Supporting Information

Palladium-Catalyzed Asymmetric Carbonylation of Alkynes to Axially Chiral Carbothioate Esters

Wang Yao,[a] Yang Yuan,[a] Ting Yang,[a] Yong-Gui Zhou,[a] and Xiao-Feng Wu*[a,b]

[a]Dalian National Laboratory for Clean Energy, Dalian Institute of Chemical Physics, Chinese Academy of Sciences, 116023 Dalian, Liaoning, China. [b]Leibniz-Institut für Katalyse e.V., 18059 Rostock, Germany. E-mail: xwu2020@dicp.ac.cn

Table of Contents

1	General information	S1
2	Synthesis of substrates **1**	S2
3	Optimization of the reaction conditions	S3
4	Synthesis of **3**	S4
5	Characterization, NMR spectra, and HPLC of products **3**	S5
6	Synthesis of **5**	S142
7	Characterization, NMR spectra, and HPLC of products **5**	S143
8	Synthesis of **7**	S155
9	Characterization, NMR spectra, and HPLC of products **7**	S156
10	Gram scale reaction and Synthetic transformations	S170
11	Characterization and NMR spectra of products 8-9	S171
12	Control experiments	S177
13	Crystallographic data for **3a**	S181
14	Rotational barriers	S184
15	Enantiomeric conversion half-life calculation	S188
16	References	S189

1. General information

Unless stated otherwise, all reagents were purchased from commercial sources and used without further purification. Solvents were dried and distilled before use by standard procedures. Reactions were monitored by thin layer chromatography (TLC) using silica gel plates. Flash column chromatography was performed over silica gel (200-300 mesh). NMR spectra were recorded on a Bruker Avance operating at for **^{1}H NMR** at 400 MHz, **^{13}C NMR** at 100 MHz, **^{19}F NMR** at 376 MHz and chemical shifts (δ) are reported in ppm relative to those of residual solvent signals: $CDCl_3$ (**^{1}H NMR** δ 7.26, **^{13}C NMR** δ 77.00), $(CD_3)_2SO$ (**^{1}H NMR** δ 2.50, **^{13}C NMR** δ 39.60). All coupling constants (*J*) are reported in Hz. The following abbreviations were used to describe peak splitting patterns when appropriate: s = singlet, d = doublet, t = triplet, q = quartet, m = multiplet, br = broad. HRMS data was obtained with Micromass HPLC-Q-TOF mass spectrometer (ESI) or Agilent 6540 Accurate-MS spectrometer (Q-TOF), using Chiralpak AD-H (4.6 mm Φ × 250 mmL), IA-H (4.6 mm Φ × 250 mmL) columns purchased from Daicel Chemical Industries.

Because of the high toxicity of carbon monoxide, all the reactions should be performed in an autoclave. The laboratory should be well-equipped with a CO detector and alarm system.

2. Synthesis of substrates 1[1]

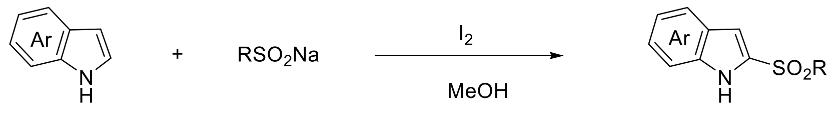

To a solution of indole (5 mmol) and sodium benzenesulfinate **II** (15 mmol) in MeOH (20 mL) was added iodine **I** (1.905 g, 7.5 mmol) and stirred the mixture at room temperature for 2 hours. Quench the reaction by the addition of saturated aqueous $Na_2S_2O_3$ (50 mL) and extract the reaction mixture with EtOAc (2 x 40 mL). Dry the combined organic extracts over MgSO4, filter the combined organic extracts and concentrate the combined organic extracts. The residue was purified by silica gel column chromatography (petroleum ether/EtOAc = 5:1) to obtain **III** as a colorless solid.

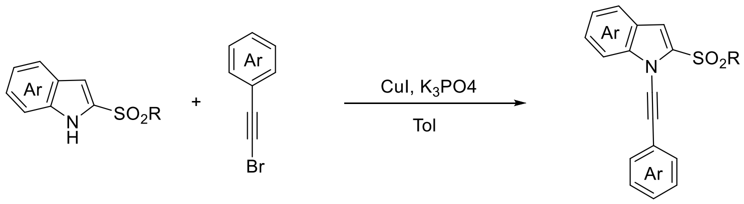

Add a solution of phenylbromoethyne **III** (3.3 mmol) in toluene (18 mL), followed by N, N-dimethylethylene diamine (0.3 mmol) to a 100 mL round bottom flask charged with 2-sulfonylindole **IV** (3 mmol), K_3PO_4 (6 mmol) and CuI (0.15 mmol). Heat the reaction mixture to 110 °C and allow to stir at this temperature for 15 h. Cool the reaction mixture to room temperature, filter through a thin pad of silica gel and concentrate in vacuo. The residue was purified by silica gel column chromatography (petroleum ether/DCM = 2:1) to obtain **1**.

3. Optimization of the reaction conditions[a]

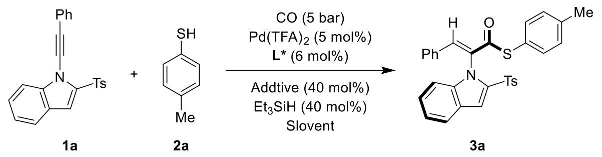

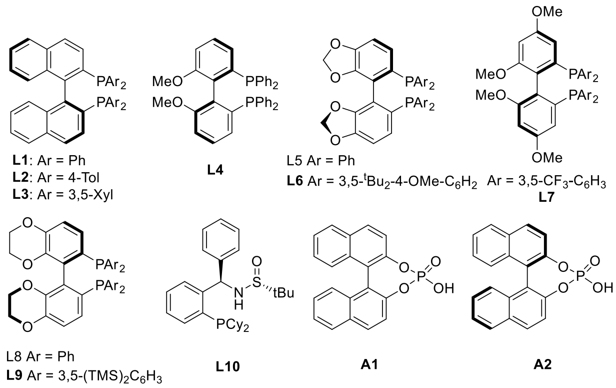

Entry	L*	Additive	Solvent	Yield	Ee
1	**L1**	-	MeCN	75	17
2	**L2**	-	MeCN	70	23
3	**L3**	-	MeCN	66	26
4	**L4**	-	MeCN	80	35
5	**L5**	-	MeCN	72	40
6	**L6**	-	MeCN	77	80
7	**L7**	-	MeCN	70	92
8	**L8**	-	MeCN	73	35
9	**L9**	-	MeCN	32	87
10	**L10**	-	MeCN	10	3
11	**L7**	PTSA·H_2O	MeCN	94	40
12	**L7**	Ph_2POOH	MeCN	55	78
13	**L7**	A1	MeCN	95	88
14	**L7**	A2	MeCN	95	92
15	**L7**	A2	DCM	49	89
16	**L7**	A2	THF	-	-
17	**L7**	A2	CF_3Ph	21	92
18[d]	**L7**	A2	MeCN	76	92
19[d][e]	**L7**	A2	MeCN	70	92

[a] Reaction conditions: **1a** (0.1 mmol), **2a** (0.15 mmol), $Pd(TFA)_2$ (5 mol%), **L*** (6 mol%), additive (40 mol%), Et_3SiH (40 mol%), solvent (0.5 mL) under CO (5 bar) at r.t. for 24 h. [b] Isolated yields of **3a**. [c] Ee values of **3a** was determined by HPLC analysis. [d] $Pd(TFA)_2$ (2.5 mol %), **L7** (3 mol %). [e] The reaction was performed at r.t. for 12h.

4. Synthesis of 3

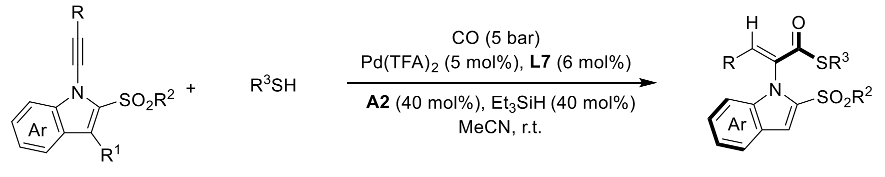

Typical Procedure A: To a mixture of **1** (0.1 mmol), **2** (0.15 mmol), $Pd(TFA)_2$ (0.005 mmol), L**7** (0.006 mmol) and **A2** (0.04 mmol) was added MeCN (0.5 mL), last added Et_3SiH (0.04 mmol). After flushing the autoclave three times with CO, a pressure of 5 bar of CO was adjusted at ambient temperature. Then, the reaction was performed for 24 h at room temperature. After the reaction was complete, the pressure of autoclave was released carefully. The mixture was diluted with EtOAc (5 mL) and extracted with EtOAc (5 mL x 3). The combined organic layers were washed with brine (10 mL), dried (Na_2SO_4), and concentrated. Further purification by flash column chromatography on silica gel (petroleum ether: EtOAc = 5:1) provided **3**.

5. Characterization, NMR spectra, and HPLC of products 3

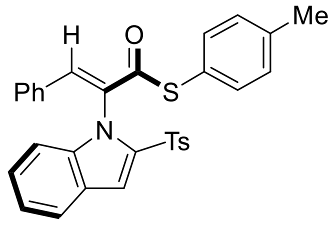

S-(p-tolyl) (Z)-3-phenyl-2-(2-tosyl-1H-indol-1-yl)prop-2-enethioate (3a) was synthesized by following Procedure A. The crude material was purified by column chromatography (SiO_2, petroleum ether: EtOAc = 5:1) to provide **3a** as a yellow solid (49 mg, 95% yield).

^{1}H NMR (400 MHz, $CDCl_3$) δ 7.85 (s, 1H), 7.82 (dt, *J* = 7.7, 1.0 Hz, 1H), 7.74 (d, *J* = 0.9 Hz, 1H), 7.69 – 7.61 (m, 2H), 7.32 (ddd, *J* = 8.4, 7.1, 1.3 Hz, 1H), 7.29 – 7.25 (m, 1H), 7.21 (d, *J* = 8.3 Hz, 2H), 7.16 (d, *J* = 8.0 Hz, 2H), 7.12 (ddt, *J* = 9.8, 7.3, 1.2 Hz, 2H), 6.96 – 6.83 (m, 4H), 6.51 – 6.33 (m, 2H), 2.34 (s, 3H), 2.10 (s, 3H).

^{13}C NMR (100 MHz, $CDCl_3$) δ 189.8, 144.1, 139.5, 138.7, 137.2, 136.8, 135.8, 134.5, 130.9, 130.9, 130.7, 129.8, 129.3, 128.8, 128.4, 128.3, 126.9, 126.0, 123.6, 123.1, 122.5, 113.0, 111.3, 21.3, 21.3.

HRMS: (ESI) m/z: $[M+H]^+$ Calcd for $C_{31}H_{26}NO_3S_2^+$ 524.1349; Found 524.1352.

Optical $[\alpha]_{25}^{D}$ = -44.9 ° (c = 0.25, CH_2Cl_2, 92% ee)

HPLC (IA-H, ^{i}PrOH/n-hexane = 30/70, flow rate = 0.5 mL/min, 250 nm) t_R = 49.5 min (major), 32.9 min (minor).

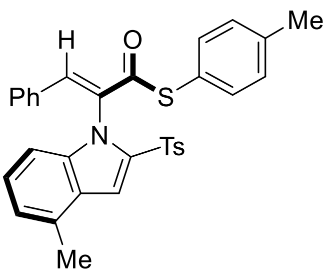

S-(p-tolyl) (Z)-2-(4-methyl-2-tosyl-1H-indol-1-yl)-3-phenylprop-2-enethioate (3b) was synthesized by following Procedure A. The crude material was purified by column chromatography (SiO_2, petroleum ether: EtOAc = 5:1) to provide **3b** as a yellow oil (46 mg, 85% yield).

^{1}H NMR (400 MHz, $CDCl_3$) δ 7.84 (s, 1H), 7.79 (d, *J* = 0.9 Hz, 1H), 7.70 – 7.64 (m, 2H), 7.25 – 7.22 (m, 1H), 7.22 – 7.19 (m, 2H), 7.18 (s, 1H), 7.18 – 7.15 (m, 1H), 7.12 (dd, *J* = 7.3, 1.4 Hz, 1H), 7.06 (dt, *J* = 7.1, 1.0 Hz, 1H), 6.99 – 6.95 (m, 1H), 6.95 (d, *J* = 1.7 Hz, 1H), 6.92 (d, *J* = 8.4 Hz, 2H), 6.89 (s, 1H), 6.53 – 6.43 (m, 2H), 2.69 (s, 3H), 2.36 (s, 3H), 2.13 (s, 3H).

^{13}C NMR (100 MHz, $CDCl_3$) δ 189.9, 144.1, 139.5, 138.7, 137.1, 137.0, 135.1, 134.6, 132.9, 131.0, 131.0, 130.7, 129.8, 129.3, 129.0, 128.5, 128.4, 127.1, 126.1, 123.7, 122.7, 111.8, 108.8, 21.3, 21.3, 18.6.

HRMS: (ESI) m/z: $[M+H]^+$ Calcd for $C_{32}H_{28}NO_3S_2^+$ 538.1511; Found 538.1511.

Optical $[\alpha]_{25}^{D}$ = -2.4 ° (c = 0.25, CH_2Cl_2, 90% ee)

HPLC (IA-H, ^{i}PrOH/n-hexane = 30/70, flow rate = 0.7 mL/min, 250 nm) t_R = 18.0 min (major), 33.5 min (minor).

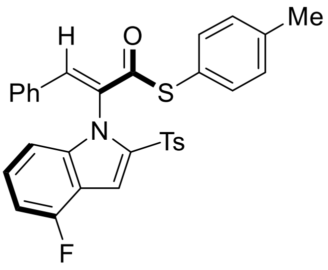

S-(p-tolyl) (Z)-2-(4-fluoro-2-tosyl-1H-indol-1-yl)-3-phenylprop-2-enethioate (3c) was synthesized by following Procedure A. The crude material was purified by column chromatography (SiO_2, petroleum ether: EtOAc = 5:1) to provide **3c** as a yellow oil (43 mg, 80% yield).

^{1}H NMR (400 MHz, $CDCl_3$) δ 7.86 (s, 1H), 7.83 (d, *J* = 0.9 Hz, 1H), 7.71 – 7.65 (m, 2H), 7.30 – 7.25 (m, 2H), 7.24 – 7.21 (m, 1H), 7.20 (d, *J* = 4.7 Hz, 3H), 7.17 – 7.13 (m, 1H), 7.04 (dt, *J* = 7.9, 1.1 Hz, 1H), 6.95 (t, *J* = 7.8 Hz, 2H), 6.91 (d, *J* = 8.2 Hz, 2H), 6.50 – 6.43 (m, 2H), 2.36 (s, 3H), 2.13 (s, 3H).

^{13}C NMR (100 MHz, $CDCl_3$) δ 189.3, 144.5, 139.7, 139.2, 137.5, 136.7, 136.5, 134.5, 131.0, 130.9, 130.7, 129.9, 129.4, 128.6, 128.6, 128.5, 128.4, 127.4, 125.2, 123.3, 122.3, 111.3, 110.0, 21.4, 21.3.

^{19}F NMR (376 MHz, $CDCl_3$) δ -120.20.

HRMS: (ESI) m/z: $[M+H]^+$ Calcd for $C_{31}H_{24}FNO_3S_2^+$ 542.1254; Found 542.1257.

Optical $[\alpha]_{25}^{D}$ = -42.5 ° (c = 0.25, CH_2Cl_2, 91% ee)

HPLC (IA-H, iPrOH/n-hexane = 30/70, flow rate = 0.7 mL/min, 250 nm) t_R = 34.0 min (major), 19.5 min (minor).

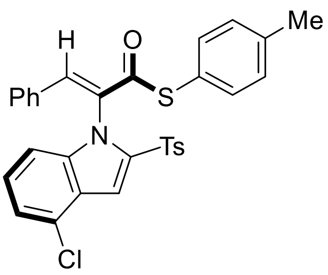

S-(p-tolyl) (Z)-2-(4-chloro-2-tosyl-1H-indol-1-yl)-3-phenylprop-2-enethioate (3d) was synthesized by following Procedure A. The crude material was purified by column chromatography (SiO_2, petroleum ether: EtOAc = 5:1) to provide **3d** as a yellow oil (48 mg, 86% yield).

^{1}H NMR (400 MHz, $CDCl_3$) δ 7.86 (s, 1H), 7.81 (d, *J* = 0.8 Hz, 1H), 7.70 – 7.65 (m, 2H), 7.28 – 7.24 (m, 1H), 7.24 – 7.22 (m, 1H), 7.21 (d, *J* = 6.7 Hz, 2H), 7.18 (d, *J* = 1.7 Hz, 1H), 7.15 (dt, *J* = 7.3, 1.3 Hz, 1H), 6.98 – 6.93 (m, 2H), 6.93 – 6.92 (m, 2H), 6.91 (d, *J* = 2.4 Hz, 2H), 6.50 – 6.43 (m, 2H), 2.36 (s, 3H), 2.13 (s, 3H).

^{13}C NMR (100 MHz, $CDCl_3$) δ 189.4, 157.1 (d, *J* = 252.6 Hz), 144.4, 140.6 (d, *J* = 9.1 Hz), 139.7, 137.5, 136.6, 136.3, 134.6, 131.0, 130.9, 130.8, 129.9, 129.4, 128.6, 128.6, 128.5, 127.7 (d, *J* = 7.8 Hz), 123.4, 115.9 (d, *J* = 23.0 Hz), 109.0, 107.4 (d, *J* = 4.1 Hz), 107.2 (d, *J* = 18.4 Hz), 21.4, 21.3.

HRMS: (ESI) m/z: $[M+H]^+$ Calcd for $C_{31}H_{25}ClNO_3S_2^+$ 558.0959; Found 558.0959.

Optical $[\alpha]_{25}^{D}$ = +29.6 ° (c = 0.25, CH_2Cl_2, 93% ee)

HPLC (IA-H, iPrOH/n-hexane = 30/70, flow rate = 0.7 mL/min, 250 nm) t_R = 29.2 min (major), 19.1 min (minor).

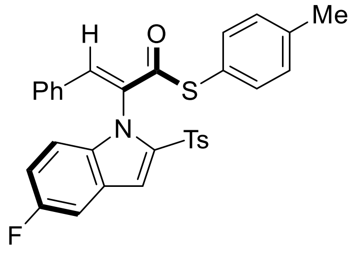

Ethyl (R, E)-3-(1-(2-formyl-5-methyl-1H-indol-1-yl)-1H-pyrrol-2-yl)acrylate (3e) was synthesized by following Procedure A. The crude material was purified by column chromatography (SiO_2, petroleum ether: EtOAc = 5:1) to provide **3e** as a yellow oil (50 mg, 92% yield).

^{1}H NMR (400 MHz, $CDCl_3$) δ 9.72 (s, 1H), 7.58 (dt, *J* = 1.8, 0.9 Hz, 1H), 7.36 (d, *J* = 0.9 Hz, 1H), 7.27 – 7.25 (m, 1H), 6.98 – 6.95 (m, 2H), 6.90 (d, *J* = 8.6 Hz, 1H), 6.77 (dd, *J* = 4.3, 1.7 Hz, 1H), 6.41 (dd, *J* = 4.3, 2.9 Hz, 1H), 5.59 (d, *J* = 16.0 Hz, 1H), 4.07 (qd, *J* = 7.1, 1.2 Hz, 2H), 2.47 (s, 3H), 1.19 (t, *J* = 7.1 Hz, 3H).

^{13}C NMR (100 MHz, $CDCl_3$) δ 179.8 (d, *J* = 4.3 Hz), 167.0, 138.5, 134.9, 132.7, 130.7 (t, *J* = 4.4 Hz), 130.0, 129.9 (d, *J* = 6.9 Hz), 126.2 – 125.5 (m), 124.3, 122.9 (d, *J* = 5.0 Hz), 114.8 (d, *J* = 7.3 Hz), 114.6, 111.8, 109.6, 109.4, 60.2, 22.5 – 19.9 (m), 14.1.

^{19}F NMR (376 MHz, $CDCl_3$) δ -120.17.

HRMS: (ESI) m/z: $[M+H]^+$ Calcd for $C_{31}H_{24}FNO_3S_2^+$ 542.1254; Found 542.1261.

Optical $[\alpha]_{25}^{D}$ = +35.7 ° (c = 0.25, CH_2Cl_2, 96% ee)

HPLC (IA-H, iPrOH/n-hexane = 30/70, flow rate = 0.7 mL/min, 250 nm) t_R = 33.0 min (major), 26.2 min (minor).

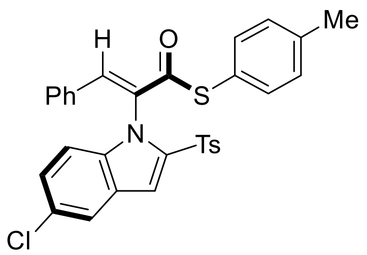

S-(p-tolyl) (Z)-2-(5-chloro-2-tosyl-1H-indol-1-yl)-3-phenylprop-2-enethioate (3f) was synthesized by following Procedure A. The crude material was purified by column chromatography (SiO_2, petroleum ether: EtOAc = 5:1) to provide **3f** as a yellow oil (50 mg, 90% yield).

^{1}H NMR (400 MHz, $CDCl_3$) δ 7.85 (s, 1H), 7.81 (d, J = 2.0 Hz, 1H), 7.70 – 7.63 (m, 3H), 7.30 – 7.26 (m, 1H), 7.23 – 7.18 (m, 4H), 7.15 (dt, J = 7.3, 1.3 Hz, 1H), 7.06 (d, J = 8.9 Hz, 1H), 6.96 (d, J = 1.6 Hz, 1H), 6.91 (d, J = 8.0 Hz, 3H), 6.47 – 6.41 (m, 2H), 2.36 (s, 3H), 2.13 (s, 3H).

^{13}C NMR (100 MHz, $CDCl_3$) δ 189.4, 144.5, 139.7, 137.5, 137.2, 137.0, 136.5, 134.5, 131.0, 130.9, 130.7, 129.9, 129.4, 128.6, 128.5, 128.5, 128.2, 127.5, 126.8, 123.3, 122.4, 112.6, 112.1, 21.4, 21.3.

HRMS: (ESI) m/z: $[M+H]^+$ Calcd for $C_{31}H_{25}ClNO_3S_2^+$ 558.0959; Found 558.0967.

Optical $[\alpha]_{25}^{D}$ = -37.4 ° (c = 0.25, CH_2Cl_2, 94% ee)

HPLC (IA-H, iPrOH/n-hexane = 30/70, flow rate = 0.7 mL/min, 250 nm) t_R = 33.0 min (major), 25.9 min (minor).

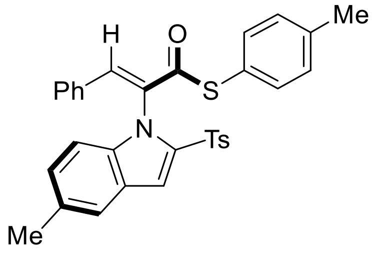

S-(p-tolyl) (Z)-2-(5-methyl-2-tosyl-1H-indol-1-yl)-3-phenylprop-2-enethioate (3g) was synthesized by following Procedure A. The crude material was purified by column chromatography (SiO_2, petroleum ether: EtOAc = 5:1) to provide **3g** as a yellow oil (46 mg, 85% yield).

^{1}H NMR (400 MHz, $CDCl_3$) δ 7.84 (s, 1H), 7.69 – 7.65 (m, 3H), 7.63 – 7.58 (m, 1H), 7.23 (d, *J* = 6.0 Hz, 1H), 7.20 (d, *J* = 9.4 Hz, 2H), 7.17 (d, *J* = 1.9 Hz, 1H), 7.17 – 7.14 (m, 1H), 7.12 (dt, *J* = 7.2, 1.3 Hz, 1H), 7.03 (d, *J* = 8.5 Hz, 1H), 6.95 – 6.86 (m, 4H), 6.51 – 6.45 (m, 2H), 2.46 (s, 3H), 2.36 (s, 3H), 2.12 (s, 3H).

^{13}C NMR (100 MHz, $CDCl_3$) δ 189.9, 144.0, 139.5, 137.1, 137.0, 137.0, 135.6, 134.5, 132.0, 131.0, 130.9, 130.7, 129.8, 129.3, 128.9, 128.8, 128.4, 128.3, 126.2, 123.7, 122.5, 112.6, 111.0, 21.4, 21.3, 21.3.

HRMS: (ESI) m/z: $[M+H]^+$ Calcd for $C_{32}H_{28}NO_3S_2^+$ 538.1511; Found 538.1516.

Optical $[\alpha]_{25}^{D}$ = -44.0 ° (c = 0.25, CH_2Cl_2, 90% ee)

HPLC (IA-H, iPrOH/n-hexane = 30/70, flow rate = 0.7 mL/min, 250 nm) t_R = 24.4 min (major), 35.0 min (minor).

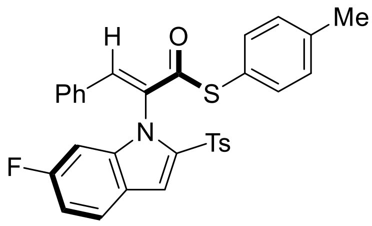

S-(p-tolyl) (Z)-2-(6-fluoro-2-tosyl-1H-indol-1-yl)-3-phenylprop-2-enethioate (3h) was synthesized by following Procedure A. The crude material was purified by column chromatography (SiO_2, petroleum ether: EtOAc = 5:1) to provide **3h** as a yellow oil (44 mg, 82% yield).

^{1}H NMR (400 MHz, $CDCl_3$) δ 7.86 (s, 1H), 7.78 (dd, *J* = 8.8, 5.2 Hz, 1H), 7.72 (d, *J* = 0.9 Hz, 1H), 7.70 – 7.65 (m, 2H), 7.24 (d, *J* = 6.0 Hz, 1H), 7.22 (s, 1H), 7.20 (s, 1H), 7.19 (d, *J* = 6.0 Hz, 1H), 7.15 (dt, *J* = 7.2, 1.3 Hz, 1H), 7.03 (td, *J* = 9.0, 2.3 Hz, 1H), 6.94 (t, *J* = 7.9 Hz, 2H), 6.91 (d, *J* = 8.0 Hz, 2H), 6.80 (dd, *J* = 9.1, 2.2 Hz, 1H), 6.50 – 6.45 (m, 2H), 2.36 (s, 3H), 2.13 (s, 3H).

^{13}C NMR (100 MHz, $CDCl_3$) δ 189.3, 162.4 (d, *J* = 245.8 Hz), 144.3, 139.7, 139.1 (d, *J* = 12.2 Hz), 137.5, 136.7, 136.5 (d, *J* = 3.9 Hz), 134.6, 131.0, 130.9, 130.7, 129.9, 129.4, 128.5, 128.5, 128.4, 124.5 (d, *J* = 10.4 Hz), 123.4, 122.4, 113.1, 112.2 (d, *J* = 25.3 Hz), 97.7 (d, *J* = 26.8 Hz), 21.3, 21.3.

^{19}F NMR (376 MHz, $CDCl_3$) δ -112.16 (dt, *J* = 9.4, 4.7 Hz).

HRMS: (ESI) m/z: $[M+H]^+$ Calcd for $C_{31}H_{25}FNO_3S_2^+$ 542.1258; Found 524.1260.

Optical $[\alpha]_{25}^{D}$ = -43.0 ° (c = 0.25, CH_2Cl_2, 92% ee)

HPLC (IA-H, iPrOH/n-hexane = 30/70, flow rate = 0.7 mL/min, 250 nm) t_R = 29.2 min (major), 20.2 min (minor).

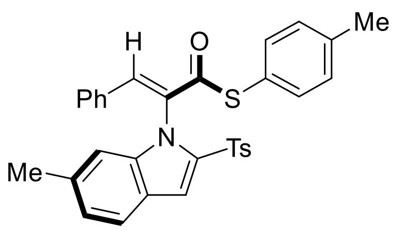

S-(p-tolyl) (Z)-2-(6-methyl-2-tosyl-1H-indol-1-yl)-3-phenylprop-2-enethioate (3i) was synthesized by following Procedure A. The crude material was purified by column chromatography (SiO_2, petroleum ether: EtOAc = 5:1) to provide **3i** as a yellow oil (47 mg, 88% yield).

^{1}H NMR (400 MHz, $CDCl_3$) δ ^{1}H NMR (400 MHz, $CDCl_3$) δ 7.84 (s, 1H), 7.74 – 7.67 (m, 2H), 7.66 – 7.62 (m, 2H), 7.22 (d, *J* = 8.3 Hz, 2H), 7.17 (d, *J* = 8.1 Hz, 2H), 7.15 – 7.11 (m, 1H), 7.09 (dd, *J* = 8.3, 1.5 Hz, 1H), 6.95 – 6.89 (m, 3H), 6.87 (d, *J* = 8.1 Hz, 2H), 6.50 – 6.44 (m, 2H), 2.37 (s, 3H), 2.35 (s, 3H), 2.11 (s, 3H).

^{13}C NMR (100 MHz, $CDCl_3$) δ 179.6, 166.9, 157.4 (d, *J* = 255.0 Hz), 141.6 (d, *J* = 8.6 Hz), 134.8, 130.0, 129.6 (d, *J* = 7.9 Hz), 129.5, 125.7, 115.0, 114.2 (d, *J* = 23.6 Hz), 112.0, 111.0, 109.9, 107.7 (d, *J* = 18.6 Hz), 105.8 (d, *J* = 4.3 Hz), 60.3, 14.2.

HRMS: (ESI) m/z: $[M+H]^+$ Calcd for $C_{32}H_{27}NO_3S_2^+$ 538.1511; Found 538.1507.

Optical $[\alpha]_{25}^{D}$ = -70.0 ° (c = 0.25, CH_2Cl_2, 92% ee)

HPLC (IA-H, iPrOH/n-hexane = 30/70, flow rate = 0.7 mL/min, 250 nm) t_R = 27.4 min (major), 17.2 min (minor).

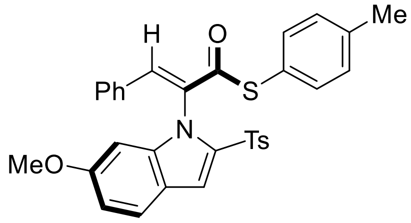

S-(p-tolyl) (Z)-2-(6-methoxy-2-tosyl-1H-indol-1-yl)-3-phenylprop-2-enethioate (3j) was synthesized by following Procedure A. The crude material was purified by column chromatography (SiO_2, petroleum ether: EtOAc = 5:1) to provide **3j** as a yellow oil (44 mg, 80% yield).

^{1}H NMR (400 MHz, $CDCl_3$) δ 7.84 (s, 1H), 7.71 – 7.67 (m, 2H), 7.67 – 7.62 (m, 2H), 7.24 (d, *J* = 6.1 Hz, 1H), 7.21 (d, *J* = 10.1 Hz, 2H), 7.17 (d, *J* = 5.6 Hz, 1H), 7.16 – 7.11 (m, 1H), 6.97 – 6.92 (m, 2H), 6.92 – 6.90 (m, 1H), 6.88 (d, *J* = 8.1 Hz, 2H), 6.54 – 6.46 (m, 3H), 3.70 (s, 3H), 2.36 (s, 3H), 2.13 (s, 3H).

^{13}C NMR (100 MHz, $CDCl_3$) δ 189.9, 159.9, 143.9, 140.1, 139.5, 137.2, 137.1, 134.5, 134.2, 131.0, 130.9, 130.8, 129.8, 129.2, 128.8, 128.5, 128.2, 123.9, 123.7, 120.0, 113.8, 113.5, 93.2, 55.5, 21.3, 21.3.

HRMS: (ESI) m/z: $[M+H]^+$ Calcd for $C_{32}H_{28}NO_4S_2^+$ 554.1460; Found 554.1461.

Optical $[\alpha]_{25}^{D}$ = -8.3 ° (c = 0.25, CH_2Cl_2, 87% ee)

HPLC (IA-H, iPrOH/n-hexane = 30/70, flow rate = 0.7 mL/min, 250 nm) t_R = 35.6 min (major), 22.1 min (minor).

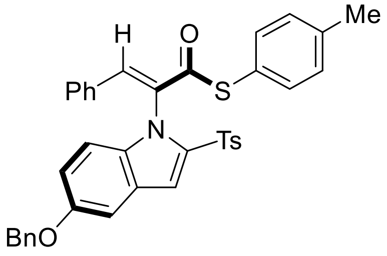

S-(p-tolyl) (Z)-2-(5-(benzyloxy)-2-tosyl-1H-indol-1-yl)-3-phenylprop-2-enethioate (3k) was synthesized by following Procedure A. The crude material was purified by column chromatography (SiO_2, petroleum ether: EtOAc = 5:1) to provide **3k** as a yellow oil (53 mg, 85% yield).

^{1}H NMR (400 MHz, $CDCl_3$) δ 7.82 (s, 1H), 7.69 (d, *J* = 8.8 Hz, 1H), 7.66 (d, *J* = 0.8 Hz, 1H), 7.65 – 7.61 (m, 2H), 7.33 – 7.29 (m, 2H), 7.29 – 7.24 (m, 2H), 7.24 – 7.20 (m, 2H), 7.19 (d, *J* = 6.6 Hz, 2H), 7.17 – 7.15 (m, 1H), 7.14 – 7.08 (m, 1H), 6.99 (dd, *J* = 8.8, 2.2 Hz, 1H), 6.91 (t, *J* = 7.9 Hz, 2H), 6.86 (d, *J* = 7.9 Hz, 2H), 6.58 (d, *J* = 2.1 Hz, 1H), 6.49 – 6.43 (m, 2H), 5.00 – 4.84 (m, 2H), 2.34 (s, 3H), 2.10 (s, 3H).

^{13}C NMR (100 MHz, $CDCl_3$) δ 189.8, 158.9, 143.9, 140.0, 139.5, 137.2, 137.1, 136.2, 134.5, 134.4, 130.9, 130.9, 130.7, 129.8, 129.2, 128.8, 128.5, 128.4, 128.2, 127.9, 127.5, 124.0, 123.7, 120.2, 114.3, 113.5, 94.5, 70.3, 21.3, 21.3.

HRMS: (ESI) m/z: $[M+H]^+$ Calcd for $C_{38}H_{32}NO_4S_2^+$ 630.1773; Found 630.1766.

Optical $[\alpha]_{25}^{D}$ = -103.2 ° (c = 0.25, CH_2Cl_2, 94% ee)

HPLC (IA-H, iPrOH/n-hexane = 30/70, flow rate = 0.7 mL/min, 250 nm) t_R = 43.5 min (major), 40.1 min (minor).

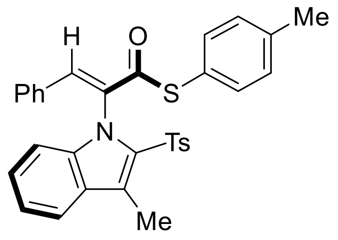

S-(p-tolyl) (Z)-2-(3-methyl-2-tosyl-1H-indol-1-yl)-3-phenylprop-2-enethioate (3l) was synthesized by following Procedure A. The crude material was purified by column chromatography (SiO_2, petroleum ether: EtOAc = 5:1) to provide **3l** as a yellow oil (39 mg, 72% yield).

^{1}H NMR (400 MHz, $CDCl_3$) δ 7.85 (dt, J = 7.9, 1.1 Hz, 1H), 7.77 – 7.74 (m, 2H), 7.70 – 7.65 (m, 2H), 7.35 (ddd, J = 8.4, 7.1, 1.3 Hz, 1H), 7.32 – 7.27 (m, 1H), 7.24 (d, J = 6.1 Hz, 1H), 7.23 (s, 1H), 7.20 (s, 1H), 7.18 (s, 1H), 7.11 (dd, J = 8.3, 1.1 Hz, 1H), 7.08 (ddd, J = 8.0, 2.1, 1.0 Hz, 1H), 6.94 (d, J = 8.1 Hz, 2H), 6.85 (t, J = 7.9 Hz, 1H), 6.34 (dt, J = 8.0, 1.3 Hz, 1H), 6.30 (t, J = 1.9 Hz, 1H), 2.36 (s, 3H), 2.15 (s, 3H).

^{13}C NMR (100 MHz, $CDCl_3$) δ 189.7, 144.3, 139.7, 138.5, 136.9, 135.6, 135.3, 134.5, 134.4, 132.6, 130.6, 130.5, 130.1, 129.9, 129.6, 129.4, 128.7, 128.3, 127.1, 126.0, 123.4, 123.3, 122.7, 113.3, 111.1, 21.3, 21.3.

HRMS: (ESI) m/z: $[M+H]^+$ Calcd for $C_{32}H_{28}NO_3S_2^+$ 538.1511; Found 538.1512.

Optical $[\alpha]_{25}^{D}$ = -141.8 ° (c = 0.25, CH_2Cl_2, 90% ee)

HPLC (IA-H, iPrOH/n-hexane = 30/70, flow rate = 0.7 mL/min, 250 nm) t_R = 20.5 min (major), 30.9 min (minor).

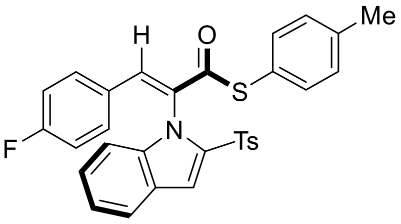

S-(p-tolyl) (Z)-3-(4-fluorophenyl)-2-(2-tosyl-1H-indol-1-yl)prop-2-enethioate (3m) was synthesized by following Procedure A. The crude material was purified by column chromatography (SiO_2, petroleum ether: EtOAc = 5:1) to provide **3m** as a yellow oil (38 mg, 70% yield).

^{1}H NMR (400 MHz, $CDCl_3$) δ 7.87 – 7.81 (m, 2H), 7.75 (d, *J* = 0.9 Hz, 1H), 7.70 – 7.66 (m, 2H), 7.35 (ddd, *J* = 8.4, 7.1, 1.4 Hz, 1H), 7.29 (ddd, *J* = 8.0, 7.1, 1.1 Hz, 1H), 7.21 – 7.16 (m, 4H), 7.13 (dq, *J* = 8.3, 0.9 Hz, 1H), 6.97 – 6.92 (m, 2H), 6.66 – 6.58 (m, 2H), 6.50 – 6.44 (m, 2H), 2.35 (s, 3H), 2.18 (s, 3H).

^{13}C NMR (100 MHz, $CDCl_3$) δ 189.7, 163.8 (d, *J* = 254.6 Hz), 144.3, 139.6, 138.6, 136.9, 135.9, 135.8, 134.5, 133.1 (d, *J* = 8.8 Hz), 129.9, 129.3, 128.5 (d, *J* = 2.7 Hz), 128.4, 127.4 (d, *J* = 3.3 Hz), 127.0, 126.0, 123.5, 123.2, 122.7, 115.7 (d, *J* = 21.8 Hz), 113.2, 111.2, 21.3, 21.3.

^{19}F NMR (376 MHz, $CDCl_3$) δ -106.91.

HRMS: (ESI) m/z: $[M+H]^+$ Calcd for $C_{31}H_{25}FNO_3S_2^+$ 542.1250; Found 542.1260.

Optical $[\alpha]_{25}^{D}$ = -52.5 ° (c = 0.25, CH_2Cl_2, 93% ee)

HPLC (IA-H, iPrOH/n-hexane = 30/70, flow rate = 0.7 mL/min, 250 nm) t_R = 38.2 min (major), 24.3 min (minor).

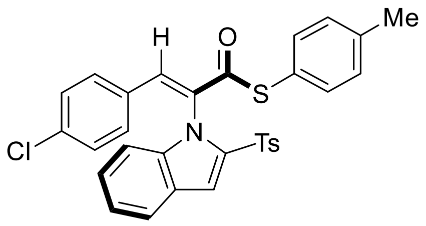

S-(p-tolyl) (Z)-3-(4-chlorophenyl)-2-(2-tosyl-1H-indol-1-yl)prop-2-enethioate (3n) was synthesized by following Procedure A. The crude material was purified by column chromatography (SiO_2, petroleum ether: EtOAc = 5:1) to provide **3n** as a yellow oil (46 mg, 82% yield).

^{1}H NMR (400 MHz, $CDCl_3$) δ 7.83 (d, J = 7.8 Hz, 1H), 7.81 (s, 1H), 7.74 (s, 1H), 7.65 (d, J = 8.3 Hz, 2H), 7.34 (ddd, J = 8.4, 7.0, 1.3 Hz, 1H), 7.31 – 7.26 (m, 1H), 7.19 (q, J = 8.2 Hz, 4H), 7.12 (d, J = 8.2 Hz, 1H), 6.92 (d, J = 8.1 Hz, 2H), 6.89 – 6.83 (m, 2H), 6.39 – 6.32 (m, 2H), 2.34 (s, 3H), 2.17 (s, 3H).

^{13}C NMR (100 MHz, $CDCl_3$) δ 189.6, 144.5, 139.6, 138.6, 137.0, 136.8, 135.8, 135.5, 134.5, 131.9, 129.9, 129.5, 129.3, 128.8, 128.6, 128.3, 127.1, 126.0, 123.5, 123.2, 122.7, 113.2, 111.2, 21.3, 21.3.

HRMS: (ESI) m/z: $[M+H]^+$ Calcd for $C_{31}H_{25}ClNO_3S_2^+$ 385.1523; Found 385.1529.

Optical $[\alpha]_{25}^{D}$ = -56.4 ° (c = 0.25, CH_2Cl_2, 94% ee)

HPLC (IA-H, iPrOH/n-hexane = 30/70, flow rate = 0.7 mL/min, 250 nm) t_R = 41.0 min (major), 25.7 min (minor).

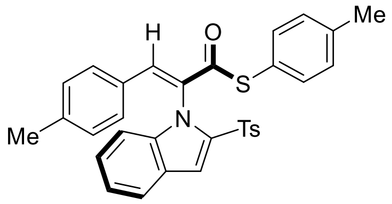

S-(p-tolyl) (Z)-3-(p-tolyl)-2-(2-tosyl-1H-indol-1-yl)prop-2-enethioate (3o) was synthesized by following Procedure A. The crude material was purified by column chromatography (SiO_2, petroleum ether: EtOAc = 5:1) to provide **3o** as a yellow oil (45 mg, 84% yield).

^{1}H NMR (400 MHz, $CDCl_3$) δ 7.85 (s, 1H), 7.83 (dd, *J* = 7.8, 1.3 Hz, 1H), 7.74 (d, *J* = 0.9 Hz, 1H), 7.71 – 7.65 (m, 2H), 7.33 (ddd, *J* = 8.3, 6.9, 1.3 Hz, 1H), 7.30 – 7.25 (m, 1H), 7.21 (d, *J* = 5.8 Hz, 1H), 7.19 (d, *J* = 5.8 Hz, 2H), 7.16 (s, 1H), 7.16 – 7.12 (m, 1H), 6.91 (d, *J* = 8.1 Hz, 2H), 6.74 (d, *J* = 8.1 Hz, 2H), 6.35 (d, *J* = 8.1 Hz, 2H), 2.35 (s, 3H), 2.19 (s, 3H), 2.14 (s, 3H).

^{13}C NMR (100 MHz, $CDCl_3$) δ 189.7, 144.1, 141.7, 139.4, 138.7, 137.4, 136.9, 135.9, 134.5, 131.0, 129.8, 129.3, 129.2, 128.4, 128.3, 127.9, 126.8, 125.9, 123.8, 123.0, 122.5, 112.9, 111.3, 21.4, 21.3, 21.3.

HRMS: (ESI) m/z: $[M+H]^+$ Calcd for $C_{32}H_{28}NO_3S_2^+$ 538.1511; Found 538.1509.

Optical $[\alpha]_{25}^{D}$ = -51.3 ° (c = 0.25, CH_2Cl_2, 91% ee)

HPLC (IA-H, iPrOH/n-hexane = 30/70, flow rate = 0.7 mL/min, 250 nm) t_R = 37.3 min (major), 22.8 min (minor).

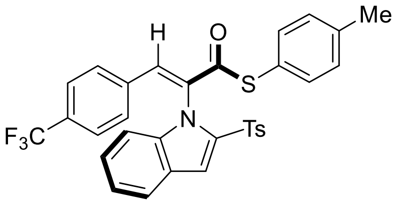

S-(p-tolyl) (Z)-2-(2-tosyl-1H-indol-1-yl)-3-(4-(trifluoromethyl)phenyl)prop-2-enethioate (3p) was synthesized by following Procedure A. The crude material was purified by column chromatography (SiO_2, petroleum ether: EtOAc = 5:1) to provide **3p** as a yellow oil (42 mg, 71% yield).

^{1}H NMR (400 MHz, $CDCl_3$) δ 7.89 – 7.83 (m, 2H), 7.76 (d, *J* = 0.9 Hz, 1H), 7.69 – 7.62 (m, 2H), 7.37 (ddd, *J* = 8.4, 7.1, 1.4 Hz, 1H), 7.31 (ddd, *J* = 8.1, 7.0, 1.1 Hz, 1H), 7.23 (d, *J* = 8.4 Hz, 2H), 7.21 – 7.16 (m, 3H), 7.16 – 7.10 (m, 2H), 6.94 – 6.84 (m, 2H), 6.56 (d, *J* = 8.2 Hz, 2H), 2.36 (s, 3H), 2.12 (s, 3H).

^{13}C NMR (100 MHz, $CDCl_3$) δ 189.7, 144.5, 139.8, 138.7, 137.0, 135.7, 135.0, 134.6, 134.3, 132.1, 131.0, 130.9, 130.0, 129.4, 128.4, 127.3, 126.1, 125.3 (q, *J* = 4.0 Hz), 123.4 (q, *J* = 272.6 Hz), 123.4, 123.3, 122.9, 113.5, 111.1, 21.4, 21.2.

^{19}F NMR (376 MHz, $CDCl_3$) δ -63.19.

HRMS: (ESI) m/z: $[M+H]^+$ Calcd for $C_{32}H_{25}F_3NO_3S_2^+$ 592.1228; Found 592.1233.

Optical $[\alpha]_{25}^{D}$ = -48.1 ° (c = 0.25, CH_2Cl_2, 97% ee)

HPLC (IA-H, iPrOH/n-hexane = 30/70, flow rate = 0.7 mL/min, 250 nm) t_R = 29.8 min (major), 23.4 min (minor).

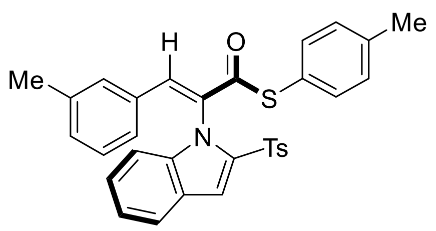

S-(p-tolyl) (Z)-3-(m-tolyl)-2-(2-tosyl-1H-indol-1-yl)prop-2-enethioate (3q) was synthesized by following Procedure A. The crude material was purified by column chromatography (SiO_2, petroleum ether: EtOAc = 5:1) to provide **3q** as a yellow oil (46 mg, 86% yield).

^{1}H NMR (400 MHz, $CDCl_3$) δ 7.82 (d, *J* = 6.9 Hz, 2H), 7.73 (d, *J* = 0.9 Hz, 1H), 7.70 – 7.61 (m, 2H), 7.31 (ddd, *J* = 8.3, 7.1, 1.3 Hz, 1H), 7.28 – 7.25 (m, 1H), 7.23 (d, *J* = 6.2 Hz, 1H), 7.21 (s, 1H), 7.18 (s, 1H), 7.16 (d, *J* = 1.9 Hz, 1H), 7.12 (dq, *J* = 8.2, 1.0 Hz, 1H), 6.92 (ddt, *J* = 7.6, 1.8, 0.9 Hz, 1H), 6.90 – 6.85 (m, 2H), 6.78 (t, *J* = 7.7 Hz, 1H), 6.25 – 6.21 (m, 1H), 6.19 (d, *J* = 1.9 Hz, 1H), 2.35 (s, 3H), 2.11 (s, 3H), 1.96 (s, 3H).

^{13}C NMR (100 MHz, $CDCl_3$) δ 189.9, 144.1, 139.6, 138.8, 138.0, 137.4, 137.0, 135.8, 134.7, 131.8, 131.7, 130.9, 129.9, 129.3, 128.7, 128.4, 128.4, 128.1, 126.9, 126.0, 123.8, 123.1, 122.5, 113.0, 111.4, 21.4, 21.4, 21.1.

HRMS: (ESI) m/z: $[M+H]^+$ Calcd for $C_{32}H_{28}NO_3S_2^+$ 538.1505; Found 538.1510.

Optical $[\alpha]_{25}^{D}$ = -41.1 ° (c = 0.25, CH_2Cl_2, 90% ee)

HPLC (IA-H, iPrOH/n-hexane = 30/70, flow rate = 0.7 mL/min, 250 nm) t_R = 28.4 min (major), 20.3 min (minor).

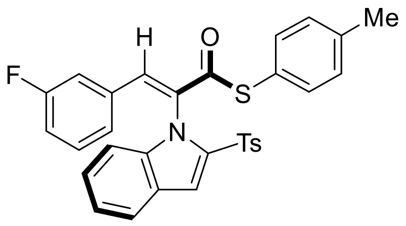

S-(p-tolyl) (Z)-3-(3-fluorophenyl)-2-(2-tosyl-1H-indol-1-yl)prop-2-enethioate (3r) was synthesized by following Procedure A. The crude material was purified by column chromatography (SiO_2, petroleum ether: EtOAc = 5:1) to provide **3r** as a yellow solid (51 mg, 94% yield).

^{1}H NMR (400 MHz, $CDCl_3$) δ 7.85 (dt, J = 7.8, 1.1 Hz, 1H), 7.81 (s, 1H), 7.76 (d, J = 0.9 Hz, 1H), 7.72 – 7.65 (m, 2H), 7.35 (ddd, J = 8.3, 7.1, 1.3 Hz, 1H), 7.29 (ddd, J = 8.1, 7.1, 1.1 Hz, 1H), 7.23 (d, J = 6.1 Hz, 1H), 7.22 (s, 1H), 7.19 (s, 1H), 7.17 (s, 1H), 7.13 (dt, J = 8.3, 1.0 Hz, 1H), 6.98 – 6.90 (m, 3H), 6.83 (tdd, J = 8.3, 2.6, 1.0 Hz, 1H), 6.43 (dt, J = 7.8, 1.2 Hz, 1H), 5.91 (dt, J = 10.2, 2.2 Hz, 1H), 2.36 (s, 3H), 2.15 (s, 3H).

^{13}C NMR (100 MHz, $CDCl_3$) δ 189.7, 162.1 (d, J = 246.9 Hz), 144.2, 139.7, 138.6, 136.9, 135.7, 135.5 (d, J = 2.8 Hz), 134.5, 133.0 (d, J = 8.1 Hz), 130.0, 129.9, 129.8, 129.3, 128.3, 127.1, 127.1, 126.0, 123.4, 123.3, 122.7, 117.7 (d, J = 21.4 Hz), 116.6 (d, J = 23.1 Hz), 113.4, 111.1, 21.3, 21.3.

^{19}F NMR (376 MHz, $CDCl_3$) δ -111.87.

HRMS: (ESI) m/z: $[M+H]^+$ Calcd for $C_{31}H_{25}FNO_3S_2^+$ 542.1260; Found 542.1260.

Optical $[\alpha]_{25}^{D}$ = -56.2 ° (c = 0.25, CH_2Cl_2, 94% ee)

HPLC (IA-H, iPrOH/n-hexane = 30/70, flow rate = 0.7 mL/min, 250 nm) t_R = 33.4 min (major), 23.4 min (minor).

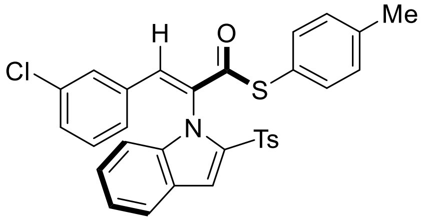

S-(p-tolyl) (Z)-3-(3-chlorophenyl)-2-(2-tosyl-1H-indol-1-yl)prop-2-enethioate (3s) was synthesized by following Procedure A. The crude material was purified by column chromatography (SiO_2, petroleum ether: EtOAc = 3:1) to provide **3s** as a yellow oil (48 mg, 86% yield).

^{1}H NMR (400 MHz, $CDCl_3$) δ 7.85 (dt, *J* = 7.9, 1.1 Hz, 1H), 7.77 – 7.74 (m, 2H), 7.70 – 7.65 (m, 2H), 7.35 (ddd, *J* = 8.4, 7.1, 1.3 Hz, 1H), 7.32 – 7.27 (m, 1H), 7.24 (d, *J* = 6.1 Hz, 1H), 7.23 (s, 1H), 7.20 (s, 1H), 7.18 (s, 1H), 7.11 (dd, *J* = 8.3, 1.1 Hz, 1H), 7.08 (ddd, *J* = 8.0, 2.1, 1.0 Hz, 1H), 6.94 (d, *J* = 8.1 Hz, 2H), 6.85 (t, *J* = 7.9 Hz, 1H), 6.34 (dt, *J* = 8.0, 1.3 Hz, 1H), 6.30 (t, *J* = 1.9 Hz, 1H), 2.36 (s, 3H), 2.15 (s, 3H).

^{13}C NMR (100 MHz, $CDCl_3$) δ 189.7, 144.3, 139.7, 138.5, 136.9, 135.6, 135.3, 134.5, 134.4, 132.6, 130.6, 130.5, 130.1, 129.9, 129.6, 129.4, 128.7, 128.3, 127.1, 126.0, 123.4, 123.3, 122.7, 113.3, 111.1, 21.3, 21.3.

HRMS: (ESI) m/z: $[M+H]^+$ Calcd for $C_{31}H_{25}ClNO_3S_2^+$ 558.0964; Found 558.0963.

Optical $[\alpha]_{25}^{D}$ = -50.7 ° (c = 0.25, CH_2Cl_2, 96% ee)

HPLC (IA-H, iPrOH/n-hexane = 30/70, flow rate = 0.7 mL/min, 250 nm) t_R = 30.0 min (major), 23.4 min (minor).

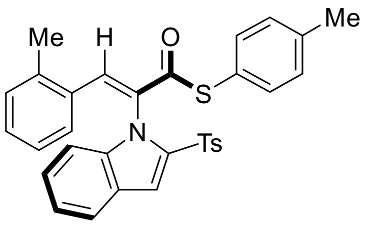

S-(p-tolyl) (Z)-3-(o-tolyl)-2-(2-tosyl-1H-indol-1-yl)prop-2-enethioate (3t) was synthesized by following Procedure A. The crude material was purified by column chromatography (SiO_2, petroleum ether: EtOAc = 5:1) to provide **3t** as a yellow oil (42 mg, 79% yield).

^{1}H NMR (400 MHz, $CDCl_3$) δ 8.20 (s, 1H), 7.82 (dt, *J* = 8.0, 1.1 Hz, 1H), 7.71 (d, *J* = 0.7 Hz, 2H), 7.69 (d, *J* = 1.8 Hz, 1H), 7.34 (ddd, *J* = 8.4, 7.1, 1.2 Hz, 1H), 7.29 – 7.24 (m, 1H), 7.22 (d, *J* = 5.9 Hz, 1H), 7.19 (s, 2H), 7.18 (d, *J* = 5.7 Hz, 1H), 7.12 (dd, *J* = 8.3, 1.0 Hz, 1H), 7.07 (dd, *J* = 7.8, 1.5 Hz, 1H), 7.01 (td, *J* = 7.4, 1.3 Hz, 1H), 6.97 (d, *J* = 8.0 Hz, 2H), 6.43 (td, *J* = 7.7, 1.5 Hz, 1H), 5.76 (dd, *J* = 8.1, 1.2 Hz, 1H), 2.48 (s, 3H), 2.36 (s, 3H), 2.18 (s, 3H).

^{13}C NMR (100 MHz, $CDCl_3$) δ 190.1, 144.1, 140.0, 139.5, 139.1, 137.0, 135.8, 134.5, 133.8, 130.6, 130.4, 129.8, 129.6, 129.3, 128.9, 128.2, 127.8, 126.9, 126.1, 125.9, 123.7, 123.1, 122.4, 113.3, 111.4, 21.4, 21.3, 20.3.

HRMS: (ESI) m/z: $[M+H]^+$ Calcd for $C_{32}H_{28}NO_3S_2^+$ 538.1511; Found 538.1512.

Optical $[\alpha]_{25}^{D}$ = -67.9 ° (c = 0.25, CH_2Cl_2, 95% ee)

HPLC (IA-H, iPrOH/n-hexane = 30/70, flow rate = 0.7 mL/min, 250 nm) t_R = 26.6 min (major), 21.1 min (minor).

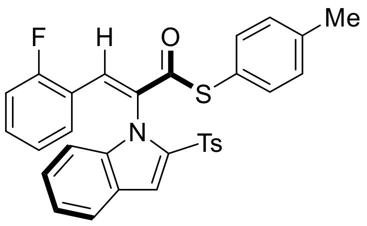

S-(p-tolyl) (Z)-3-(2-fluorophenyl)-2-(2-tosyl-1H-indol-1-yl)prop-2-enethioate (3u) was synthesized by following Procedure A. The crude material was purified by column chromatography (SiO_2, petroleum ether: EtOAc = 5:1) to provide **3u** as a yellow oil (51 mg, 95% yield).

^{1}H NMR (400 MHz, $CDCl_3$) δ 8.22 (s, 1H), 7.83 (dt, *J* = 7.9, 1.1 Hz, 1H), 7.74 (d, *J* = 1.0 Hz, 1H), 7.72 (d, *J* = 1.9 Hz, 1H), 7.71 (d, *J* = 1.8 Hz, 1H), 7.35 (ddd, *J* = 8.4, 7.1, 1.3 Hz, 1H), 7.31 – 7.27 (m, 1H), 7.22 – 7.16 (m, 4H), 7.15 – 7.09 (m, 2H), 7.00 – 6.97 (m, 2H), 6.97 – 6.91 (m, 1H), 6.44 – 6.36 (m, 1H), 5.63 (td, *J* = 7.7, 1.7 Hz, 1H), 2.36 (s, 3H), 2.17 (s, 3H).

^{13}C NMR (100 MHz, $CDCl_3$) δ 189.3, 161.9 (d, *J* = 255.8 Hz), 144.3, 139.6, 138.7, 136.8, 135.8, 134.5, 132.5 (d, *J* = 9.2 Hz), 130.1 (d, *J* = 2.3 Hz), 129.8, 129.4, 128.6, 128.4, 128.1 (d, *J* = 8.1 Hz), 127.0, 125.9, 124.2 (d, *J* = 3.7 Hz), 123.4, 123.2, 122.6, 119.2 (d, *J* = 9.9 Hz), 115.3 (d, *J* = 22.4 Hz), 113.3, 111.2, 21.4, 21.3.

^{31}P NMR (243 MHz, $CDCl_3$) δ -112.59.

HRMS: (ESI) m/z: $[M+H]^+$ Calcd for $C_{31}H_{25}FNO_3S_2^+$ 542.1260; Found 542.1262.

Optical $[\alpha]_{25}^{D}$ = -51.3 ° (c = 0.25, CH_2Cl_2, 99% ee)

HPLC (IA-H, iPrOH/n-hexane = 30/70, flow rate = 0.7 mL/min, 250 nm) t_R = 21.0 min (major), 17.6 min (minor).

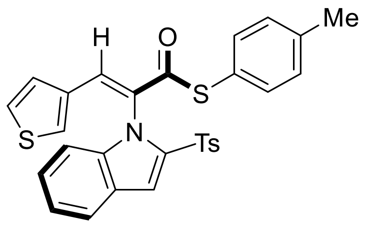

S-(p-tolyl) (Z)-3-(thiophen-3-yl)-2-(2-tosyl-1H-indol-1-yl)prop-2-enethioate (3v) was synthesized by following Procedure A. The crude material was purified by column chromatography (SiO_2, petroleum ether: EtOAc = 5:1) to provide **3v** as a yellow oil (44 mg, 83% yield).

^{1}H NMR (400 MHz, $CDCl_3$) δ 7.91 (s, 1H), 7.83 (dt, J = 7.8, 1.1 Hz, 1H), 7.74 (d, J = 0.9 Hz, 1H), 7.70 – 7.58 (m, 2H), 7.35 (ddd, J = 8.4, 7.1, 1.3 Hz, 1H), 7.31 – 7.26 (m, 1H), 7.25 – 7.22 (m, 1H), 7.20 (d, J = 11.0 Hz, 2H), 7.18 (d, J = 5.8 Hz, 1H), 7.14 (dq, J = 8.3, 0.9 Hz, 1H), 6.98 – 6.90 (m, 2H), 6.82 (dd, J = 3.0, 1.2 Hz, 1H), 6.77 (dd, J = 5.1, 2.9 Hz, 1H), 5.67 (dd, J = 5.1, 1.3 Hz, 1H), 2.36 (s, 3H), 2.20 (s, 3H).

^{13}C NMR (100 MHz, $CDCl_3$) δ 189.6, 144.2, 139.5, 138.8, 136.8, 135.9, 134.6, 133.2, 132.9, 131.1, 129.8, 129.2, 128.4, 127.3, 127.2, 126.9, 126.0, 125.8, 123.7, 123.1, 122.5, 112.9, 111.3, 21.4, 21.3.

HRMS: (ESI) m/z: $[M+H]^+$ Calcd for $C_{29}H_{24}NO_3S_3^+$ 530.0918; Found 530.0918.

Optical $[\alpha]_{25}^{D}$ = -41.9 ° (c = 0.25, CH_2Cl_2, 90% ee)

HPLC (IA-H, iPrOH/n-hexane = 30/70, flow rate = 0.7 mL/min, 250 nm) t_R = 40.1 min (major), 25.2 min (minor).

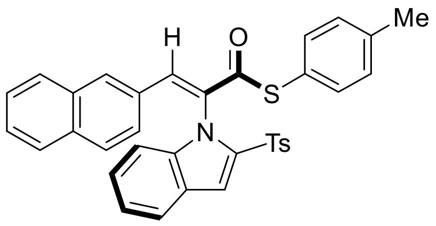

S-(p-tolyl) (Z)-3-(naphthalen-2-yl)-2-(2-tosyl-1H-indol-1-yl)prop-2-enethioate (3w) was synthesized by following Procedure A. The crude material was purified by column chromatography (SiO_2, petroleum ether: EtOAc = 5:1) to provide **3w** as a yellow oil (46 mg, 81% yield).

^{1}H NMR (400 MHz, $CDCl_3$) δ 8.02 (s, 1H), 7.90 – 7.85 (m, 1H), 7.80 (d, *J* = 0.9 Hz, 1H), 7.65 – 7.61 (m, 2H), 7.59 (d, *J* = 8.4 Hz, 1H), 7.49 – 7.42 (m, 2H), 7.41 – 7.36 (m, 1H), 7.33 – 7.29 (m, 1H), 7.29 – 7.25 (m, 3H), 7.23 – 7.21 (m, 1H), 7.20 (d, *J* = 3.5 Hz, 2H), 7.18 (d, *J* = 3.3 Hz, 1H), 7.16 (d, *J* = 1.8 Hz, 1H), 6.65 (d, *J* = 8.1 Hz, 2H), 6.19 (dd, *J* = 8.7, 1.8 Hz, 1H), 2.36 (s, 3H), 1.67 (s, 3H).

^{13}C NMR (100 MHz, $CDCl_3$) δ 189.8, 144.1, 139.5, 139.0, 137.1, 136.7, 136.0, 134.6, 133.9, 133.4, 132.5, 129.9, 129.1, 128.9, 128.8, 128.6, 128.6, 128.2, 128.0, 127.2, 127.0, 126.5, 126.0, 125.5, 123.7, 123.1, 122.6, 113.0, 111.4, 21.3, 20.8.

HRMS: (ESI) m/z: $[M+H]^+$ Calcd for $C_{35}H_{28}NO_3S_2^+$ 574.1511; Found 574.1512.

Optical $[\alpha]_{25}^{D}$ = -71.1 ° (c = 0.25, CH_2Cl_2, 90% ee)

HPLC (AD-H, ^{i}PrOH/n-hexane = 30/70, flow rate = 0.5 mL/min, 254 nm) t_R = 56.7 min (major), 30.5 min (minor).

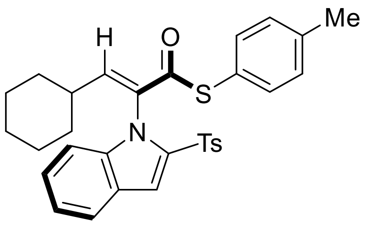

S-(p-tolyl) (Z)-3-cyclohexyl-2-(2-tosyl-1H-indol-1-yl)prop-2-enethioate (3x) was synthesized by following Procedure A. The crude material was purified by column chromatography (SiO_2, petroleum ether: EtOAc = 5:1) to provide **3x** as a yellow oil (45 mg, 85% yield).

^{1}H NMR (400 MHz, $CDCl_3$) δ 7.86 – 7.81 (m, 2H), 7.78 (dt, *J* = 8.1, 1.0 Hz, 1H), 7.64 (d, *J* = 0.8 Hz, 1H), 7.39 (ddd, *J* = 8.3, 7.0, 1.2 Hz, 1H), 7.29 – 7.24 (m, 3H), 7.20 – 7.17 (m, 1H), 7.16 (d, *J* = 1.8 Hz, 1H), 7.11 (d, *J* = 8.0 Hz, 2H), 6.94 – 6.90 (m, 2H), 2.37 (s, 3H), 2.32 (s, 3H), 1.95 – 1.83 (m, 1H), 1.73 – 1.54 (m, 4H), 1.54 – 1.35 (m, 2H), 1.27 – 1.02 (m, 4H), 1.02 – 0.80 (m, 2H).

^{13}C NMR (100 MHz, $CDCl_3$) δ 187.9, 150.3, 144.5, 140.7, 139.4, 137.5, 136.3, 134.1, 130.1, 129.8, 129.6, 128.6, 126.6, 125.4, 123.4, 122.9, 122.1, 113.2, 111.3, 37.7, 31.3, 30.7, 25.5, 25.0, 24.7.

HRMS: (ESI) m/z: $[M+H]^+$ Calcd for $C_{31}H_{32}NO_3S_2^+$ 530.1824; Found 530.1823.

Optical $[\alpha]_{25}^{D}$ = -2.5 ° (c = 0.25, CH_2Cl_2, 82% ee)

HPLC (IA-H, iPrOH/n-hexane = 30/70, flow rate = 0.7 mL/min, 250 nm) t_R = 13.8 min (major), 12.0 min (minor).

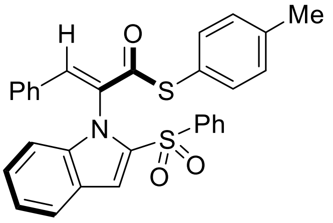

S-(p-tolyl) (Z)-3-phenyl-2-(2-(phenylsulfonyl)-1H-indol-1-yl)prop-2-enethioate (3y) was synthesized by following Procedure A. The crude material was purified by column chromatography (SiO_2, petroleum ether: EtOAc = 5:1) to provide **3y** as a yellow oil (45 mg, 88% yield).

^{1}H NMR (400 MHz, $CDCl_3$) δ 7.87 (s, 1H), 7.83 (ddd, *J* = 8.7, 7.1, 1.3 Hz, 3H), 7.76 (d, *J* = 0.9 Hz, 1H), 7.36 – 7.31 (m, 1H), 7.30 – 7.26 (m, 1H), 7.23 (d, *J* = 6.0 Hz, 1H), 7.22 (s, 1H), 7.21 – 7.16 (m, 3H), 7.13 (tdd, *J* = 7.7, 3.2, 1.5 Hz, 4H), 6.91 (t, *J* = 7.9 Hz, 2H), 6.49 – 6.42 (m, 2H), 2.36 (s, 3H).

^{13}C NMR (100 MHz, $CDCl_3$) δ 189.8, 139.9, 139.6, 138.7, 137.1, 135.6, 134.6, 133.1, 131.0, 130.9, 130.8, 129.9, 128.9, 128.7, 128.6, 128.3, 127.1, 126.0, 123.6, 123.1, 122.6, 113.5, 111.4, 21.3.

HRMS: (ESI) m/z: $[M+H]^+$ Calcd for $C_{30}H_{24}NO_3S_2^+$ 510.1198; Found 510.1196.

Optical $[\alpha]_{25}^{D}$ = -47.3 ° (c = 0.25, CH_2Cl_2, 93% ee)

HPLC (IA-H, iPrOH/n-hexane = 30/70, flow rate = 0.7 mL/min, 250 nm) t_R = 30.3 min (major), 21.6 min (minor).

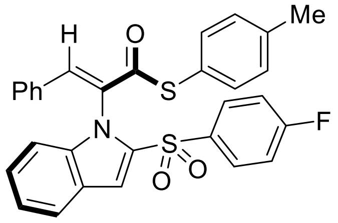

S-(p-tolyl) (Z)-2-(2-((4-fluorophenyl)sulfonyl)-1H-indol-1-yl)-3-phenylprop-2-enethioate (3z) was synthesized by following Procedure A. The crude material was purified by column chromatography (SiO_2, petroleum ether: EtOAc = 5:1) to provide **3z** as a yellow oil (48 mg, 90% yield).

^{1}H NMR (400 MHz, $CDCl_3$) δ 7.86 (d, J = 2.4 Hz, 1H), 7.86 – 7.82 (m, 1H), 7.82 – 7.77 (m, 2H), 7.76 (d, J = 1.0 Hz, 1H), 7.36 (ddd, J = 8.3, 7.0, 1.3 Hz, 1H), 7.30 (ddd, J = 8.0, 7.1, 1.1 Hz, 1H), 7.25 – 7.23 (m, 1H), 7.21 (d, J = 9.7 Hz, 2H), 7.20 – 7.10 (m, 3H), 6.95 (t, J = 7.9 Hz, 2H), 6.79 – 6.72 (m, 2H), 6.50 – 6.42 (m, 2H), 2.36 (s, 3H).

^{13}C NMR (100 MHz, $CDCl_3$) δ 189.7, 165.3 (d, J = 256.4 Hz), 139.7, 138.9, 137.2, 135.9, 135.2, 134.6, 131.2, 131.1, 130.8 (d, J = 1.9 Hz), 129.9, 128.9, 128.7, 127.2, 125.9, 123.5, 123.2, 122.7, 116.0, 115.8, 113.6, 111.4, 21.3.

^{19}F NMR (376 MHz, $CDCl_3$) δ -103.98.

HRMS: (ESI) m/z: $[M+H]^+$ Calcd for $C_{30}H_{23}FNO_3S_2^+$ 528.1103; Found 528.1104.

Optical $[\alpha]_{25}^{D}$ = -38.7 ° (c = 0.25, CH_2Cl_2, 90% ee)

HPLC (IA-H, iPrOH/n-hexane = 30/70, flow rate = 0.7 mL/min, 250 nm) t_R = 32.5 min (major), 19.7 min (minor).

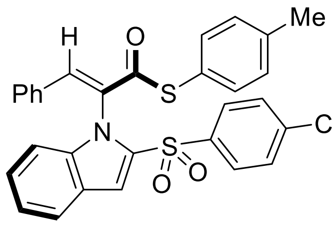

S-(p-tolyl) (Z)-2-(2-((4-chlorophenyl)sulfonyl)-1H-indol-1-yl)-3-phenylprop-2-enethioate (3aa) was synthesized by following Procedure A. The crude material was purified by column chromatography (SiO_2, petroleum ether: EtOAc = 5:1) to provide **3aa** as a yellow oil (50 mg, 91% yield).

^{1}H NMR (400 MHz, $CDCl_3$) δ 7.86 (d, *J* = 9.4 Hz, 2H), 7.77 (s, 1H), 7.73 – 7.67 (m, 2H), 7.37 (ddd, *J* = 8.3, 7.0, 1.3 Hz, 1H), 7.33 – 7.28 (m, 1H), 7.23 (d, *J* = 2.2 Hz, 1H), 7.21 (d, *J* = 4.2 Hz, 3H), 7.19 (d, *J* = 1.6 Hz, 1H), 7.18 – 7.16 (m, 1H), 6.96 (t, *J* = 7.7 Hz, 2H), 6.46 (dd, *J* = 8.0, 1.4 Hz, 2H), 2.36 (s, 3H).

^{13}C NMR (100 MHz, $CDCl_3$) δ 189.7, 139.9, 139.7, 139.0, 138.3, 137.4, 134.9, 134.6, 131.3, 130.8, 130.7, 130.0, 129.7, 129.0, 128.7, 128.7, 127.3, 125.9, 123.4, 123.2, 122.7, 113.8, 111.4, 77.2, 21.3.

HRMS: (ESI) m/z: $[M+H]^+$ Calcd for $C_{30}H_{23}ClNO_3S_2^+$ 544.0808; Found 544.0809.

Optical $[\alpha]_{25}^{D}$ = -55.1 ° (c = 0.25, CH_2Cl_2, 91% ee)

HPLC (IA-H, iPrOH/n-hexane = 30/70, flow rate = 0.7 mL/min, 250 nm) t_R = 39.2 min (major), 21.3 min (minor).

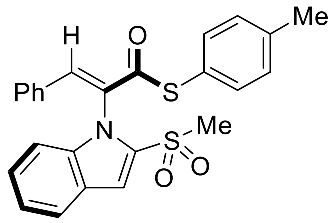

S-(p-tolyl) (Z)-2-(2-(methylsulfonyl)-1H-indol-1-yl)-3-phenylprop-2-enethioate (3ab) was synthesized by following Procedure A. The crude material was purified by column chromatography (SiO_2, petroleum ether: EtOAc = 5:1) to provide **3ab** as a yellow solid (34 mg, 75% yield).

^{1}H NMR (400 MHz, $CDCl_3$) δ 8.07 (s, 1H), 7.83 (dt, *J* = 8.0, 1.0 Hz, 1H), 7.59 (d, *J* = 0.9 Hz, 1H), 7.40 (ddd, *J* = 8.3, 7.0, 1.2 Hz, 1H), 7.33 – 7.30 (m, 1H), 7.29 – 7.24 (m, 4H), 7.20 – 7.13 (m, 4H), 6.88 – 6.83 (m, 2H), 2.93 (s, 3H), 2.35 (s, 3H).

^{13}C NMR (100 MHz, $CDCl_3$) δ 189.7, 139.9, 139.7, 139.0, 138.3, 137.4, 134.9, 134.6, 131.3, 130.8, 130.7, 130.0, 129.7, 129.0, 128.7, 128.7, 127.3, 125.9, 123.4, 123.2, 122.7, 113.8, 111.4, 21.3.

HRMS: (ESI) m/z: $[M+H]^+$ Calcd for $C_{25}H_{22}NO_3S_2^+$ 448.1041; Found 448.1041.

Optical $[\alpha]_{25}^{D}$ = -166.0 ° (c = 0.25, CH_2Cl_2, 84% ee)

HPLC (IA-H, iPrOH/n-hexane = 30/70, flow rate = 0.7 mL/min, 250 nm) t_R = 45.3 min (major), 57.3 min (minor).

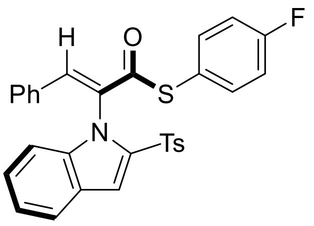

S-(4-fluorophenyl) (Z)-3-phenyl-2-(2-tosyl-1H-indol-1-yl)prop-2-enethioate (3ac) was synthesized by following Procedure A. The crude material was purified by column chromatography (SiO_2, petroleum ether: EtOAc = 5:1) to provide **3ac** as a yellow solid (35 mg, 67% yield).

^{1}H NMR (400 MHz, $CDCl_3$) δ 7.87 – 7.81 (m, 2H), 7.75 (d, *J* = 0.9 Hz, 1H), 7.69 – 7.62 (m, 2H), 7.37 – 7.33 (m, 2H), 7.33 – 7.30 (m, 1H), 7.29 – 7.26 (m, 1H), 7.16 – 7.04 (m, 4H), 6.95 – 6.84 (m, 4H), 6.49 – 6.35 (m, 2H), 2.10 (s, 3H).

^{13}C NMR (100 MHz, $CDCl_3$) δ 189.6, 163.5 (d, *J* = 249.9 Hz), 144.3, 138.7, 137.4, 136.9, 136.8 (d, *J* = 8.7 Hz), 135.8, 131.0, 130.9, 130.8, 129.4, 128.6, 128.5, 128.4, 127.1, 126.1, 123.2, 122.7, 122.6 (d, *J* = 3.4 Hz), 116.4 (d, *J* = 22.1 Hz), 113.2, 111.3, 21.4.

^{19}F NMR (376 MHz, $CDCl_3$) δ -111.31.

HRMS: (ESI) m/z: $[M+H]^+$ Calcd for $C_{30}H_{23}FNO_3S_2^+$ 528.1103; Found 528.1100.

Optical $[\alpha]_{25}^{D}$ = -20.6 ° (c = 0.25, CH_2Cl_2, 93% ee)

HPLC (IA-H, iPrOH/n-hexane = 30/70, flow rate = 0.7 mL/min, 250 nm) t_R = 30.2 min (major), 25.5 min (minor).

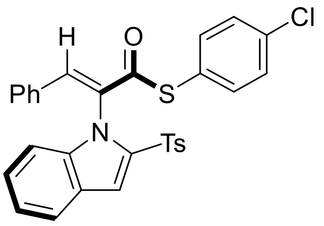

S-(4-chlorophenyl) (Z)-3-phenyl-2-(2-tosyl-1H-indol-1-yl)prop-2-enethioate (3ad) was synthesized by following Procedure A. The crude material was purified by column chromatography (SiO_2, petroleum ether: EtOAc = 5:1) to provide **3ad** as a yellow solid (35 mg, 65% yield).

^{1}H NMR (400 MHz, $CDCl_3$) δ 7.88 – 7.81 (m, 2H), 7.75 (d, J = 0.9 Hz, 1H), 7.68 – 7.61 (m, 2H), 7.37 – 7.31 (m, 3H), 7.31 – 7.26 (m, 3H), 7.16 – 7.09 (m, 2H), 6.95 – 6.85 (m, 4H), 6.49 – 6.39 (m, 2H), 2.10 (s, 3H).

^{13}C NMR (100 MHz, $CDCl_3$) δ 189.1, 144.2, 138.6, 137.5, 136.8, 135.9, 135.8, 135.7, 131.0, 130.9, 130.8, 129.3, 129.3, 128.6, 128.5, 128.3, 128.2, 127.0, 126.0, 125.9, 123.2, 122.7, 113.2, 111.2, 21.3.

HRMS: (ESI) m/z: $[M+H]^+$ Calcd for $C_{30}H_{23}ClNO_3S_2^+$ 544.0808; Found 544.0807.

Optical $[\alpha]_{25}^{D}$ = -44.2 ° (c = 0.25, CH_2Cl_2, 95% ee)

HPLC (IA-H, iPrOH/n-hexane = 30/70, flow rate = 0.7 mL/min, 250 nm) t_R = 35.4 min (major), 28.4 min (minor).

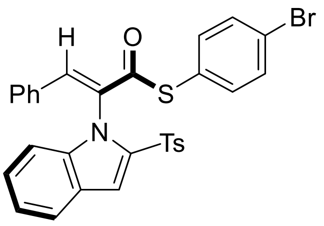

S-(4-bromophenyl) (Z)-3-phenyl-2-(2-tosyl-1H-indol-1-yl)prop-2-enethioate (3ae) was synthesized by following Procedure A. The crude material was purified by column chromatography (SiO_2, petroleum ether: EtOAc = 5:1) to provide **3ae** as a yellow solid (35 mg, 60% yield).

^{1}H NMR (400 MHz, $CDCl_3$) δ 7.90 – 7.84 (m, 2H), 7.77 (d, *J* = 0.9 Hz, 1H), 7.72 – 7.65 (m, 2H), 7.56 – 7.49 (m, 2H), 7.38 – 7.29 (m, 2H), 7.29 – 7.26 (m, 1H), 7.24 (d, *J* = 1.9 Hz, 1H), 7.19 – 7.12 (m, 2H), 6.97 – 6.86 (m, 4H), 6.49 – 6.41 (m, 2H), 2.12 (s, 3H).

^{13}C NMR (100 MHz, $CDCl_3$) δ 189.0, 144.2, 138.6, 137.5, 136.8, 136.1, 135.8, 132.2, 131.0, 130.9, 130.7, 129.3, 128.5, 128.4, 128.3, 127.0, 126.5, 126.0, 124.0, 123.2, 122.7, 113.1, 111.2, 21.3.

HRMS: (ESI) m/z: $[M+H]^+$ Calcd for $C_{30}H_{23}BrNO_3S_2^+$ 588.0303; Found 588.0304.

Optical $[\alpha]_{25}^{D}$ = -41.0 ° (c = 0.25, CH_2Cl_2, 93% ee)

HPLC (IA-H, iPrOH/n-hexane = 30/70, flow rate = 0.7 mL/min, 250 nm) t_R = 40.1 min (major), 32.3 min (minor).

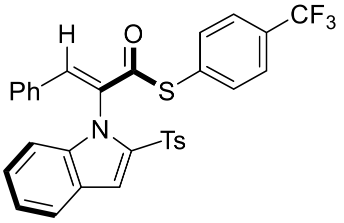

S-(4-(trifluoromethyl)phenyl) (Z)-3-phenyl-2-(2-tosyl-1H-indol-1-yl)prop-2-enethioate (3af) was synthesized by following Procedure A. The crude material was purified by column chromatography (SiO_2, petroleum ether: EtOAc = 5:1) to provide **3af** as a yellow solid (40 mg, 70% yield).

^{1}H NMR (400 MHz, $CDCl_3$) δ 7.88 – 7.82 (m, 2H), 7.75 (d, *J* = 0.8 Hz, 1H), 7.69 – 7.59 (m, 4H), 7.50 (d, *J* = 8.2 Hz, 2H), 7.38 – 7.24 (m, 3H), 7.17 – 7.10 (m, 2H), 6.95 – 6.84 (m, 4H), 6.47 – 6.40 (m, 2H), 2.09 (s, 3H).

^{13}C NMR (100 MHz, $CDCl_3$) δ 188.5, 144.3, 138.6, 137.8, 136.8, 135.8, 134.8, 132.3, 132.3, 131.1, 131.0, 130.7, 129.3, 128.5, 128.4, 128.3, 127.1, 126.1, 125.8 (q, *J* = 3.7 Hz), 123.4 (q, *J* = 272.6 Hz), 123.3, 122.7, 113.2, 111.2, 21.3.

^{19}F NMR (376 MHz, $CDCl_3$) δ -62.82.

HRMS: (ESI) m/z: $[M+H]^+$ Calcd for $C_{31}H_{23}F_3NO_3S_2^+$ 578.1071; Found 578.1071.

Optical $[\alpha]_{25}^{D}$ = -36.5 ° (c = 0.25, CH_2Cl_2, 96% ee)

HPLC (IA-H, iPrOH/n-hexane = 30/70, flow rate = 0.7 mL/min, 250 nm) t_R = 30.0 min (major), 23.6 min (minor).

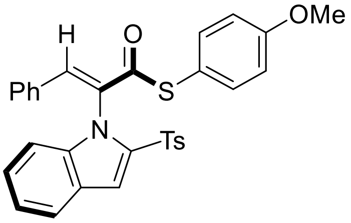

S-(4-methoxyphenyl) (Z)-3-phenyl-2-(2-tosyl-1H-indol-1-yl)prop-2-enethioate (3ag) was synthesized by following Procedure A. The crude material was purified by column chromatography (SiO_2, petroleum ether: EtOAc = 5:1) to provide **3ag** as a yellow solid (33 mg, 61% yield).

^{1}H NMR (400 MHz, $CDCl_3$) δ 7.86 (s, 1H), 7.84 (dt, *J* = 7.6, 1.0 Hz, 1H), 7.74 (d, *J* = 0.9 Hz, 1H), 7.70 – 7.65 (m, 2H), 7.34 (ddd, *J* = 8.3, 7.0, 1.4 Hz, 1H), 7.30 – 7.27 (m, 1H), 7.25 (d, *J* = 3.9 Hz, 1H), 7.18 (dt, *J* = 4.0, 1.8 Hz, 2H), 7.16 – 7.10 (m, 3H), 6.94 – 6.87 (m, 4H), 6.46 (dd, *J* = 8.4, 1.3 Hz, 2H), 2.35 (s, 3H), 2.12 (s, 3H).

^{13}C NMR (100 MHz, $CDCl_3$) δ 190.3, 160.6, 144.1, 138.7, 137.1, 136.9, 136.2, 135.8, 130.9, 130.9, 130.7, 129.3, 128.8, 128.5, 128.4, 126.9, 126.0, 123.1, 122.5, 117.8, 114.7, 113.0, 111.3, 55.3, 21.3.

HRMS: (ESI) m/z: $[M+H]^+$ Calcd for $C_{31}H_{26}NO_4S_2^+$ 540.1303; Found 540.1302.

Optical $[\alpha]_{25}^{D}$ = -23.2 ° (c = 0.25, CH_2Cl_2, 91% ee)

HPLC (IA-H, iPrOH/n-hexane = 30/70, flow rate = 0.7 mL/min, 250 nm) t_R = 50.0 min (major), 34.7 min (minor).

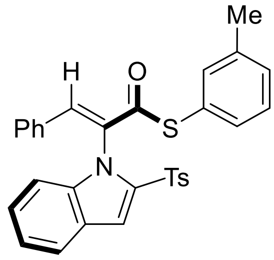

S-(m-tolyl) (Z)-3-phenyl-2-(2-tosyl-1H-indol-1-yl)prop-2-enethioate (3ah) was synthesized by following Procedure A. The crude material was purified by column chromatography (SiO_2, petroleum ether: EtOAc = 5:1) to provide **3ah** as a yellow solid (38 mg, 72% yield).

^{1}H NMR (400 MHz, $CDCl_3$) δ 7.85 (d, *J* = 14.3 Hz, 2H), 7.74 (s, 1H), 7.68 (d, *J* = 8.1 Hz, 2H), 7.34 (ddd, *J* = 8.3, 7.0, 1.3 Hz, 1H), 7.29 (d, *J* = 7.0 Hz, 1H), 7.25 (d, *J* = 7.5 Hz, 1H), 7.20 – 7.16 (m, 2H), 7.16 – 7.09 (m, 3H), 6.96 – 6.86 (m, 4H), 6.46 (d, *J* = 7.8 Hz, 2H), 2.35 (s, 3H), 2.12 (s, 3H).

^{13}C NMR (100 MHz, $CDCl_3$) δ 189.6, 144.2, 138.8, 138.7, 137.2, 136.9, 135.8, 135.1, 131.6, 130.9, 130.8, 130.2, 129.3, 128.8, 128.6, 128.5, 128.4, 128.3, 126.9, 126.8, 126.0, 123.1, 122.5, 113.1, 111.3, 21.3, 21.3.

HRMS: (ESI) m/z: $[M+H]^+$ Calcd for $C_{31}H_{26}NO_3S_2^+$ 524.1354; Found 524.1352.

Optical $[\alpha]_{25}^{D}$ = -47.1 ° (c = 0.25, CH_2Cl_2, 93% ee)

HPLC (IA-H, iPrOH/n-hexane = 30/70, flow rate = 0.7 mL/min, 250 nm) t_R = 18.8 min (major), 15.7 min (minor).

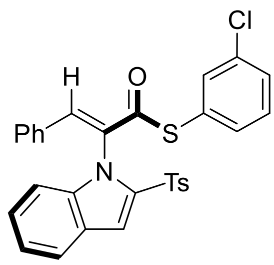

S-(3-chlorophenyl) (Z)-3-phenyl-2-(2-tosyl-1H-indol-1-yl)prop-2-enethioate (3ai) was synthesized by following Procedure A. The crude material was purified by column chromatography (SiO_2, petroleum ether: EtOAc = 5:1) to provide **3ai** as a yellow solid (51 mg, 94% yield).

^{1}H NMR (400 MHz, $CDCl_3$) δ 7.89 – 7.82 (m, 2H), 7.75 (d, *J* = 0.9 Hz, 1H), 7.70 – 7.64 (m, 2H), 7.37 – 7.32 (m, 3H), 7.30 (d, *J* = 2.7 Hz, 1H), 7.29 – 7.26 (m, 1H), 7.26 – 7.23 (m, 1H), 7.17 – 7.10 (m, 2H), 6.96 – 6.88 (m, 4H), 6.51 – 6.44 (m, 2H), 2.13 (s, 3H).

^{13}C NMR (100 MHz, $CDCl_3$) δ 188.7, 144.3, 138.6, 137.8, 136.8, 135.8, 134.5, 134.2, 132.8, 131.0, 130.8, 130.0, 129.5, 129.4, 129.1, 128.7, 128.5, 128.4, 128.3, 127.1, 126.0, 123.2, 122.7, 113.2, 111.2, 21.4.

HRMS: (ESI) m/z: $[M+H]^+$ Calcd for $C_{30}H_{23}ClNO_3S_2^+$ 544.0808; Found 544.0810.

Optical $[\alpha]_{25}^{D}$ = -45.7 ° (c = 0.25, CH_2Cl_2, 94% ee)

HPLC (IA-H, iPrOH/n-hexane = 30/70, flow rate = 0.7 mL/min, 250 nm) t_R = 19.4 min (major), 17.6 min (minor).

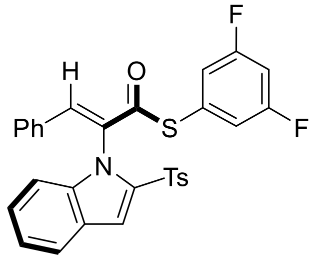

S-(4-bromophenyl) (Z)-3-phenyl-2-(2-tosyl-1H-indol-1-yl)prop-2-enethioate (3aj) was synthesized by following Procedure A. The crude material was purified by column chromatography (SiO_2, petroleum ether: EtOAc = 5:1) to provide **3aj** as a yellow solid (41 mg, 75% yield).

^{1}H NMR (400 MHz, $CDCl_3$) δ 7.87 (s, 1H), 7.85 (dt, *J* = 7.7, 1.0 Hz, 1H), 7.75 (d, *J* = 0.9 Hz, 1H), 7.70 – 7.64 (m, 2H), 7.35 (ddd, *J* = 8.4, 7.1, 1.4 Hz, 1H), 7.29 (ddd, *J* = 8.1, 7.1, 1.2 Hz, 1H), 7.18 – 7.13 (m, 1H), 7.11 (dq, *J* = 8.3, 1.0 Hz, 1H), 6.95 (d, *J* = 1.3 Hz, 1H), 6.94 (s, 1H), 6.92 – 6.91 (m, 2H), 6.91 – 6.87 (m, 2H), 6.83 (tt, *J* = 8.9, 2.3 Hz, 1H), 6.50 – 6.45 (m, 2H), 2.13 (s, 3H).

^{13}C NMR (100 MHz, $CDCl_3$) δ 188.1, 162.6 (d, *J* = 251.2 Hz), 162.5 (d, *J* = 251.2 Hz), 144.4, 138.6, 138.1, 136.8, 135.8, 131.2, 131.1, 130.7, 130.6, 129.4, 128.6, 128.4, 128.1, 127.1, 123.3, 122.8, 113.4, 111.1, 105.1 (t, *J* = 25.2 Hz), 21.3.

^{19}F NMR (376 MHz, $CDCl_3$) δ -108.89.

HRMS: (ESI) m/z: $[M+H]^+$ Calcd for $C_{30}H_{22}F_2NO_3S_2^+$ 546.1009; Found 546.1010.

Optical $[\alpha]_{25}^{D}$ = -44.1 ° (c = 0.25, CH_2Cl_2, 94% ee)

HPLC (IA-H, iPrOH/n-hexane = 30/70, flow rate = 0.7 mL/min, 250 nm) t_R = 14.2 min (major), 13.5 min (minor).

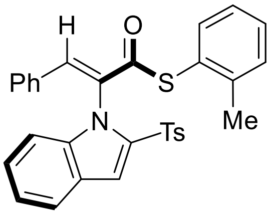

S-(o-tolyl) (Z)-3-phenyl-2-(2-tosyl-1H-indol-1-yl)prop-2-enethioate (3ak) was synthesized by following Procedure A. The crude material was purified by column chromatography (SiO_2, petroleum ether: EtOAc = 5:1) to provide **3ak** as a yellow solid (43 mg, 82% yield).

^{1}H NMR (400 MHz, $CDCl_3$) δ 7.87 – 7.81 (m, 2H), 7.74 (d, J = 0.9 Hz, 1H), 7.68 – 7.60 (m, 2H), 7.37 – 7.32 (m, 1H), 7.32 – 7.26 (m, 4H), 7.21 – 7.14 (m, 2H), 7.14 – 7.08 (m, 1H), 6.93 – 6.82 (m, 4H), 6.44 – 6.37 (m, 2H), 2.36 (s, 3H), 2.11 (s, 3H).

^{13}C NMR (100 MHz, $CDCl_3$) δ 188.9, 144.1, 142.9, 138.8, 136.9, 136.1, 135.8, 130.9, 130.9, 130.6, 130.0, 129.2, 129.0, 128.4, 128.3, 126.9, 126.5, 126.3, 126.1, 123.1, 122.5, 113.0, 111.3, 21.3, 20.7.

HRMS: (ESI) m/z: $[M+H]^+$ Calcd for $C_{31}H_{26}NO_3S_2^+$ 523.1354; Found 523.1354.

Optical $[\alpha]_{25}^{D}$ = -46.2 ° (c = 0.25, CH_2Cl_2, 93% ee)

HPLC (IA-H, iPrOH/n-hexane = 30/70, flow rate = 0.7 mL/min, 250 nm) t_R = 19.2 min (major), 16.2 min (minor).

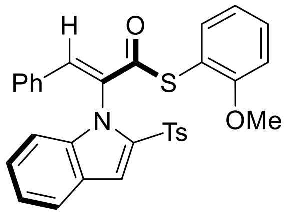

S-(2-methoxyphenyl) (Z)-3-phenyl-2-(2-tosyl-1H-indol-1-yl)prop-2-enethioate (3al) was synthesized by following Procedure A. The crude material was purified by column chromatography (SiO_2, petroleum ether: EtOAc = 5:1) to provide **3al** as a yellow solid (46 mg, 85% yield).

^{1}H NMR (400 MHz, $CDCl_3$) δ 7.87 (s, 1H), 7.81 (dt, J = 7.9, 1.1 Hz, 1H), 7.71 (d, J = 0.9 Hz, 1H), 7.69 – 7.64 (m, 2H), 7.41 – 7.36 (m, 1H), 7.36 – 7.30 (m, 2H), 7.28 – 7.23 (m, 1H), 7.17 (dq, J = 8.4, 1.0 Hz, 1H), 7.15 – 7.09 (m, 1H), 6.96 (td, J = 7.5, 1.2 Hz, 1H), 6.94 – 6.92 (m, 1H), 6.92 (t, J = 2.0 Hz, 1H), 6.90 (d, J = 7.6 Hz, 2H), 6.87 (s, 1H), 6.48 – 6.40 (m, 2H), 3.81 (s, 3H), 2.12 (s, 3H).

^{13}C NMR (100 MHz, $CDCl_3$) δ 188.3, 159.5, 144.0, 138.8, 137.3, 137.0, 136.8, 135.8, 131.5, 131.0, 130.8, 130.6, 129.2, 129.2, 128.4, 128.3, 126.8, 126.0, 123.0, 122.5, 121.0, 115.4, 113.0, 111.5, 111.5, 56.0, 21.3.

HRMS: (ESI) m/z: $[M+H]^+$ Calcd for $C_{31}H_{26}NO_4S_2^+$ 548.1303; Found 548.1308.

Optical $[\alpha]_{25}^{D}$ = -29.9 ° (c = 0.25, CH_2Cl_2, 90% ee)

HPLC (IA-H, iPrOH/n-hexane = 30/70, flow rate = 0.7 mL/min, 250 nm) t_R = 31.4 min (major), 29.2 min (minor).

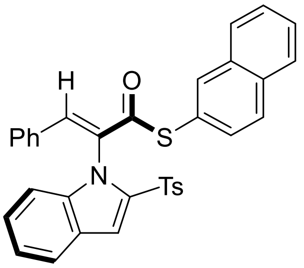

S-(naphthalen-2-yl) (Z)-3-phenyl-2-(2-tosyl-1H-indol-1-yl)prop-2-enethioate (3am) was synthesized by following Procedure A. The crude material was purified by column chromatography (SiO_2, petroleum ether: EtOAc = 5:1) to provide **3am** as a yellow solid (53 mg, 94% yield).

^{1}H NMR (400 MHz, $CDCl_3$) δ 7.89 (s, 1H), 7.88 – 7.82 (m, 4H), 7.79 (d, *J* = 10.7 Hz, 2H), 7.72 – 7.68 (m, 2H), 7.50 (tt, *J* = 7.0, 5.2 Hz, 2H), 7.43 (dd, *J* = 8.5, 1.8 Hz, 1H), 7.36 (ddd, *J* = 8.3, 7.0, 1.3 Hz, 1H), 7.30 (td, *J* = 7.6, 1.1 Hz, 1H), 7.18 (d, *J* = 8.1 Hz, 1H), 7.14 (t, *J* = 7.5 Hz, 1H), 6.95 – 6.88 (m, 4H), 6.50 – 6.45 (m, 2H), 2.11 (s, 3H).

^{13}C NMR (100 MHz, $CDCl_3$) δ 189.6, 144.2, 138.7, 137.3, 136.9, 135.8, 134.5, 133.5, 133.3, 131.0, 131.0, 130.9, 130.8, 129.3, 128.7, 128.6, 128.5, 128.4, 128.3, 127.9, 127.7, 127.0, 126.4, 126.0, 124.7, 123.2, 122.6, 113.2, 111.3, 21.4.

HRMS: (ESI) m/z: $[M+H]^+$ Calcd for $C_{34}H_{26}NO_3S_2^+$ 530.1354; Found 530.1356.

Optical $[\alpha]_{25}^{D}$ = -22.8 ° (c = 0.25, CH_2Cl_2, 93% ee)

HPLC (IA-H, iPrOH/n-hexane = 30/70, flow rate = 0.7 mL/min, 250 nm) t_R = 56.0 min (major), 46.0 min (minor).

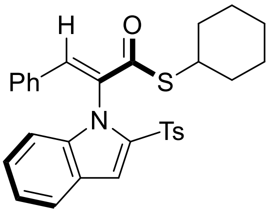

S-cyclohexyl (Z)-3-phenyl-2-(2-tosyl-1H-indol-1-yl)prop-2-enethioate (3an) was synthesized by following Procedure A. The crude material was purified by column chromatography (SiO_2, petroleum ether: EtOAc = 5:1) to provide **3an** as a yellow solid (37 mg, 72% yield).

^{1}H NMR (400 MHz, $CDCl_3$) δ 7.84 (s, 1H), 7.82 – 7.78 (m, 1H), 7.68 (d, J = 0.9 Hz, 1H), 7.66 – 7.60 (m, 2H), 7.30 – 7.20 (m, 3H), 7.15 – 7.09 (m, 1H), 7.03 (dq, J = 8.3, 1.0 Hz, 1H), 6.94 – 6.87 (m, 4H), 6.49 – 6.43 (m, 2H), 3.42 (tt, J = 10.4, 3.8 Hz, 1H), 2.14 (s, 3H), 1.91 – 1.78 (m, 2H), 1.68 – 1.51 (m, 4H), 1.37 – 1.21 (m, 4H).

^{13}C NMR (100 MHz, $CDCl_3$) δ 190.6, 144.0, 138.8, 137.0, 136.4, 135.7, 131.1, 130.8, 130.5, 129.5, 129.2, 128.4, 128.4, 126.7, 125.9, 123.0, 122.3, 112.8, 111.3, 43.1, 32.6, 26.0, 25.5, 21.3.

HRMS: (ESI) m/z: $[M+H]^+$ Calcd for $C_{30}H_{30}NO_3S_2^+$ 516.1667; Found 516.1666.

Optical $[\alpha]_{25}^{D}$ = -15.3 ° (c = 0.25, CH_2Cl_2, 90% ee)

HPLC (IA-H, iPrOH/n-hexane = 30/70, flow rate = 0.7 mL/min, 250 nm) t_R = 13.2 min (major), 12.5 min (minor).

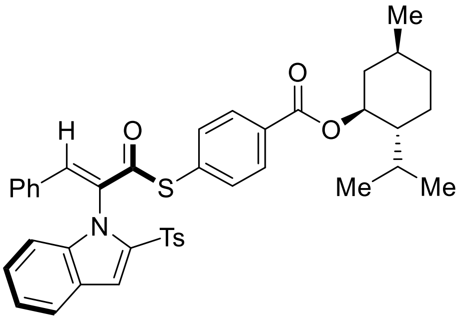

(1S,2R,5S)-2-isopropyl-5-methylcyclohexyl 4-(((Z)-3-phenyl-2-(2-tosyl-1H-indol-1-yl)acryloyl)thio)benzoate (3ao) was synthesized by following Procedure A. The crude material was purified by column chromatography (SiO_2, petroleum ether: EtOAc = 5:1) to provide **3ao** as a yellow solid (59 mg, 85% yield).

^{1}H NMR (400 MHz, $CDCl_3$) δ 8.06 – 8.01 (m, 2H), 7.88 – 7.83 (m, 2H), 7.76 (s, 1H), 7.67 (d, *J* = 8.1 Hz, 2H), 7.47 – 7.41 (m, 2H), 7.34 (ddd, *J* = 8.3, 7.0, 1.4 Hz, 1H), 7.31 – 7.27 (m, 1H), 7.14 (dt, *J* = 7.4, 3.4 Hz, 2H), 6.92 (d, *J* = 7.7 Hz, 2H), 6.88 (d, *J* = 7.5 Hz, 2H), 6.50 – 6.40 (m, 2H), 4.94 (td, *J* = 10.9, 4.4 Hz, 1H), 2.10 (d, *J* = 1.9 Hz, 4H), 1.94 (ddt, *J* = 11.4, 9.5, 3.4 Hz, 1H), 1.76 – 1.69 (m, 2H), 1.56 (tt, *J* = 12.1, 2.8 Hz, 2H), 1.18 – 1.06 (m, 2H), 0.93 (p, *J* = 4.6 Hz, 7H), 0.79 (d, *J* = 6.9 Hz, 3H).

^{13}C NMR (100 MHz, $CDCl_3$) δ 188.6, 188.5, 165.4, 144.2, 138.6, 137.7, 137.7, 136.8, 135.8, 134.3, 134.3, 132.9, 131.4, 131.0, 130.7, 129.9, 129.3, 128.5, 128.4, 128.3, 127.0, 126.0, 123.2, 122.7, 113.2, 111.2, 77.2, 75.1, 47.2, 40.8, 34.2, 31.4, 26.4, 26.4, 23.6, 23.5, 22.0, 21.3, 20.7, 16.5, 16.4.

HRMS: (ESI) m/z: $[M+H]^+$ Calcd for $C_{41}H_{42}NO_5S_2^+$ 692.2504; Found 692.2505.

Optical $[\alpha]_{25}^{D}$ = -37.8 ° (c = 0.25, CH_2Cl_2, >20:1 dr)

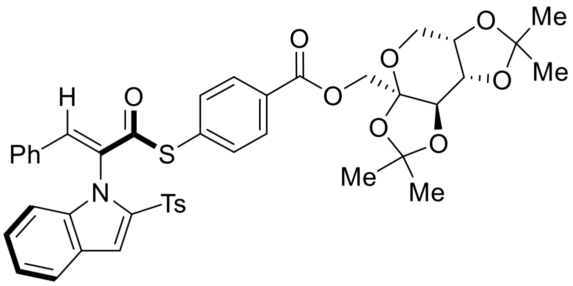

((3aR,5aS,8aS,8bR)-2,2,7,7-tetramethyltetrahydro-3aH-bis([1,3]dioxolo)[4,5-b:4',5'-d]pyran-3a-yl)methyl 4-(((Z)-3-phenyl-2-(2-tosyl-1H-indol-1-yl)acryloyl)thio)benzoate (3ap) was synthesized by following Procedure A. The crude material was purified by column chromatography (SiO_2, petroleum ether: EtOAc = 5:1) to provide **3ap** as a yellow solid (66 mg, 83% yield).

^{1}H NMR (400 MHz, $CDCl_3$) δ 8.08 – 8.02 (m, 2H), 7.85 (s, 1H), 7.83 (dt, *J* = 7.7, 1.0 Hz, 1H), 7.74 (d, *J* = 0.9 Hz, 1H), 7.67 – 7.63 (m, 2H), 7.47 – 7.42 (m, 2H), 7.35 – 7.26 (m, 2H), 7.12 (dd, *J* = 7.6, 1.3 Hz, 2H), 6.93 – 6.84 (m, 4H), 6.46 – 6.40 (m, 2H), 4.69 – 4.61 (m, 2H), 4.44 (d, *J* = 2.7 Hz, 1H), 4.35 – 4.31 (m, 1H), 4.27 – 4.23 (m, 1H), 2.08 (s, 3H), 1.54 (s, 3H), 1.45 (s, 3H), 1.39 (s, 1H), 1.36 (s, 3H), 1.33 (s, 3H).

^{13}C NMR (100 MHz, $CDCl_3$) δ 188.3, 165.3, 144.2, 138.5, 137.7, 136.7, 135.7, 134.2, 133.6, 131.0, 131.0, 130.7, 130.3, 130.0, 129.3, 128.5, 128.3, 128.3, 127.0, 126.0, 123.2, 122.7, 113.2, 111.1, 109.1, 109.0, 108.8, 108.4, 103.0, 101.5, 83.0, 70.9, 70.7, 70.7, 70.4, 70.0, 70.0, 65.4, 65.4, 61.3, 61.2, 26.4, 26.4, 25.8, 25.7, 25.5, 25.3, 24.5, 24.5, 24.5, 23.9, 23.9, 21.3.

HRMS: (ESI) m/z: $[M+H]^+$ Calcd for $C_{43}H_{42}NO_{10}S_2^+$ 796.2250; Found 796.2251.

Optical $[\alpha]_{25}^{D}$ = -39.4 ° (c = 0.25, CH_2Cl_2, >20:1 dr)

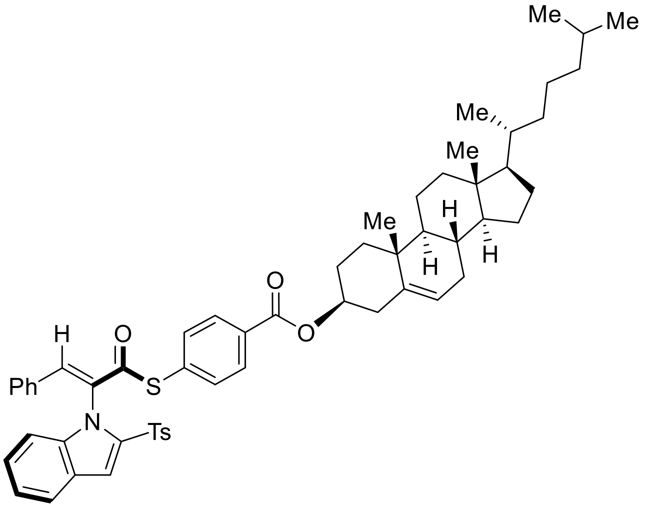

(3S,8S,9S,10R,13R,14S,17R)-10,13-dimethyl-17-((R)-6-methylheptan-2-yl)-2,3,4,7,8,9,10,11,12,13,14,15,16,17-tetradecahydro-1H-cyclopenta[a]phenanthren-3-yl 4-(((Z)-3-phenyl-2-(2-tosyl-1H-indol-1-yl)acryloyl)thio)benzoate (3aq) was synthesized by following Procedure A. The crude material was purified by column chromatography (SiO_2, petroleum ether: EtOAc = 5:1) to provide **3aq** as a yellow solid (82 mg, 89% yield).

^{1}H NMR (400 MHz, $CDCl_3$) δ 8.05 – 7.99 (m, 2H), 7.87 – 7.82 (m, 2H), 7.75 (d, J = 0.9 Hz, 1H), 7.68 – 7.65 (m, 2H), 7.44 – 7.41 (m, 2H), 7.37 – 7.32 (m, 1H), 7.31 – 7.27 (m, 1H), 7.16 – 7.12 (m, 2H), 6.95 – 6.86 (m, 4H), 6.48 – 6.42 (m, 2H), 5.44 – 5.38 (m, 1H), 4.86 (ddd, J = 8.5, 6.4, 4.1 Hz, 1H), 2.46 (d, J = 7.7 Hz, 2H), 2.11 (s, 3H), 2.04 – 1.70 (m, 8H), 1.63 – 1.41 (m, 10H), 1.07 (s, 5H), 1.01 (s, 2H), 0.92 (dd, J = 6.6, 3.4 Hz, 4H), 0.87 (dt, J = 6.6, 1.7 Hz, 8H), 0.69 (d, J = 4.8 Hz, 4H).

^{13}C NMR (100 MHz, $CDCl_3$) δ 188.5, 165.4, 144.3, 139.6, 138.6, 137.7, 136.8, 135.8, 134.2, 133.0, 131.4, 131.0, 130.8, 129.9, 129.3, 128.5, 128.5, 128.4, 127.1, 126.0, 123.2, 122.8, 122.7, 121.7, 113.2, 111.2, 77.2, 74.8, 56.7, 56.1, 50.0, 42.3, 39.7, 39.5, 38.2, 37.0, 36.6, 36.5, 36.2, 35.8, 31.9, 31.6, 28.2, 28.0, 27.8, 24.3, 23.8, 22.8, 22.6, 21.4, 21.0, 19.4, 18.7, 11.8.

HRMS: (ESI) m/z: $[M+H]^+$ Calcd for $C_{58}H_{68}NO_5S_2^+$ 922.4539; Found 922.4540.

Optical $[\alpha]_{25}^{D}$ = -34.2 ° (c = 0.25, CH_2Cl_2, >20:1 dr)

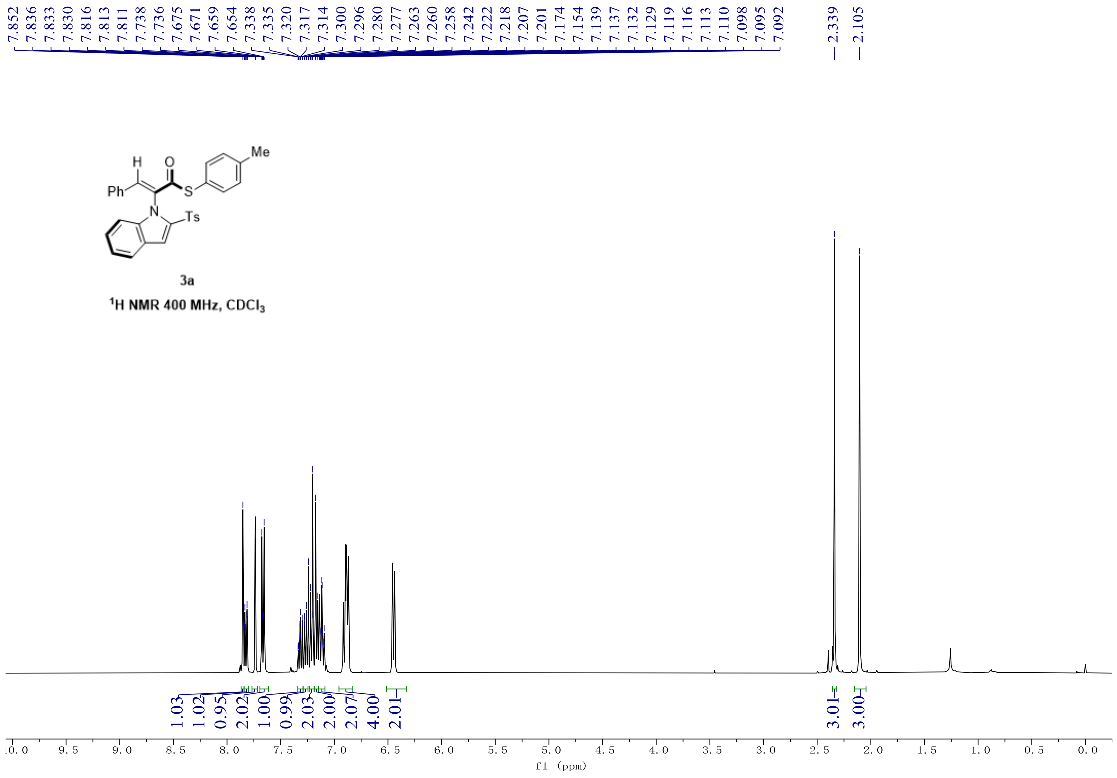
7.852
7.836
7.833
7.830
7.816
7.813
7.811
7.738
7.736
7.675
7.671
7.659
7.654
7.338
7.335
7.320
7.317
7.314
7.300
7.296
7.280
7.277
7.263
7.260
7.258
7.242
7.222
7.218
7.207
7.201
7.174
7.154
7.139
7.137
7.132
7.129
7.119
7.116
7.113
7.110
7.098
7.095
7.092
2.339
2.105
Me
H
O
Ph
S
N
Ts
3a
¹H NMR 400 MHz, CDCl3
1.03
1.02
0.95
2.02
1.00
0.99
2.03
2.00
2.07
4.00
2.01
3.01
3.00
f1 (ppm)

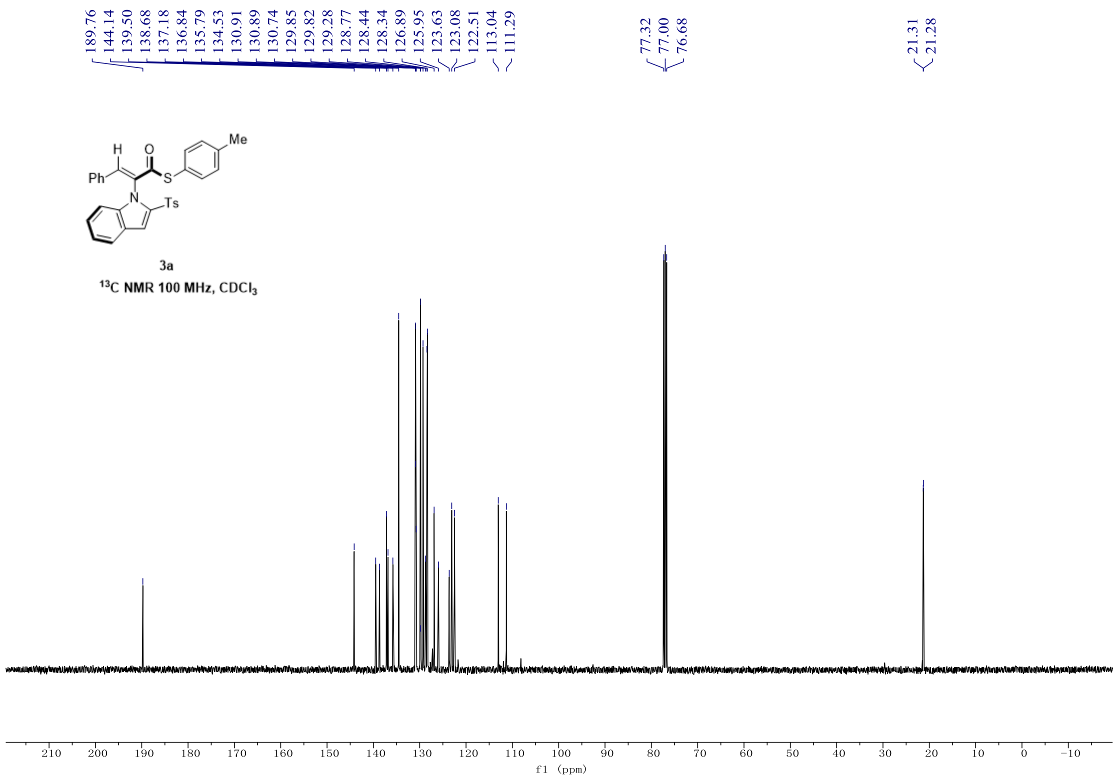
189.76
144.14
139.50
138.68
137.18
136.84
135.79
134.53
130.91
130.89
130.74
129.85
129.82
129.28
128.77
128.44
128.34
126.89
125.95
123.63
123.08
122.51
113.04
111.29
77.32
77.00
76.68
21.31
21.28
Me
H
O
Ph
S
N
Ts
3a
¹³C NMR 100 MHz, CDCl3
f1 (ppm)

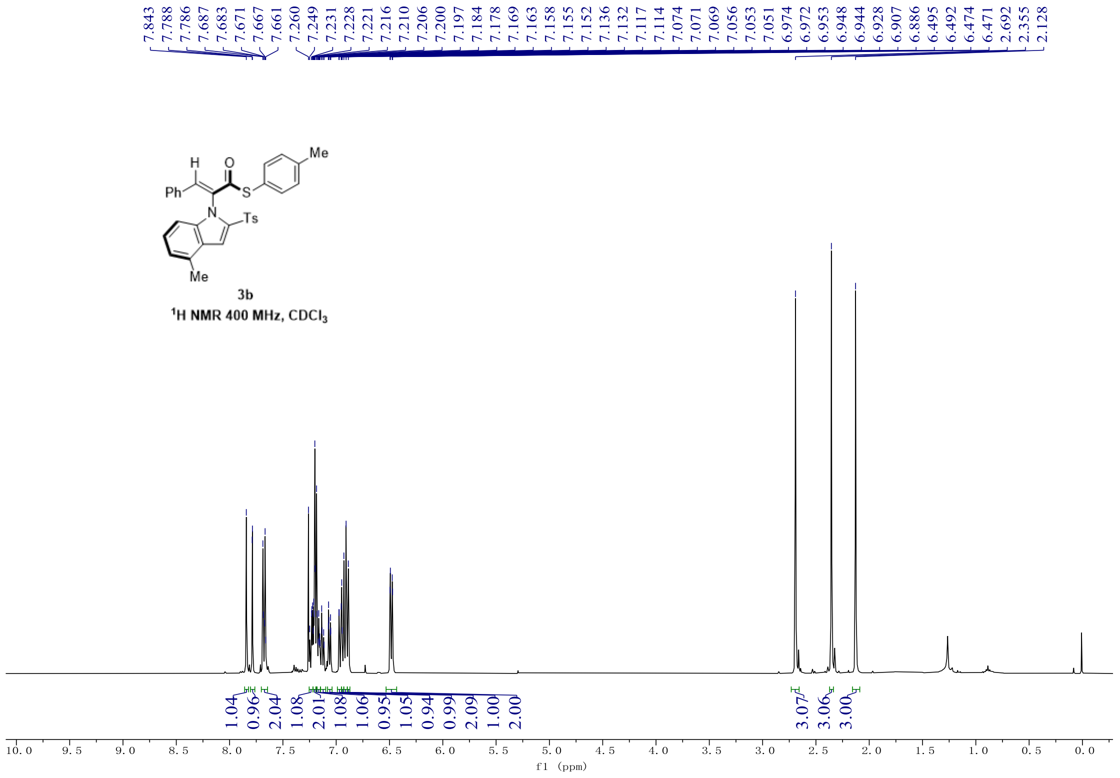
7.843 7.788 7.786 7.687 7.683 7.671 7.667 7.661 7.260 7.249 7.231 7.228 7.221 7.216 7.210 7.206 7.200 7.197 7.184 7.178 7.169 7.163 7.158 7.155 7.152 7.136 7.132 7.117 7.114 7.074 7.071 7.069 7.056 7.053 7.051 6.974 6.972 6.953 6.948 6.944 6.928 6.907 6.886 6.495 6.492 6.474 6.471 2.692 2.355 2.128
Me
H
O
S
Ph
N
Ts
Me
3b
1H NMR 400 MHz, CDCl3
1.04 0.96 2.04 1.08 2.01 1.08 1.06 0.95 1.05 0.94 0.99 2.09 1.00 2.00 3.07 3.06 3.00
10.0 9.5 9.0 8.5 8.0 7.5 7.0 6.5 6.0 5.5 5.0 4.5 4.0 3.5 3.0 2.5 2.0 1.5 1.0 0.5 0.0
f1 (ppm)

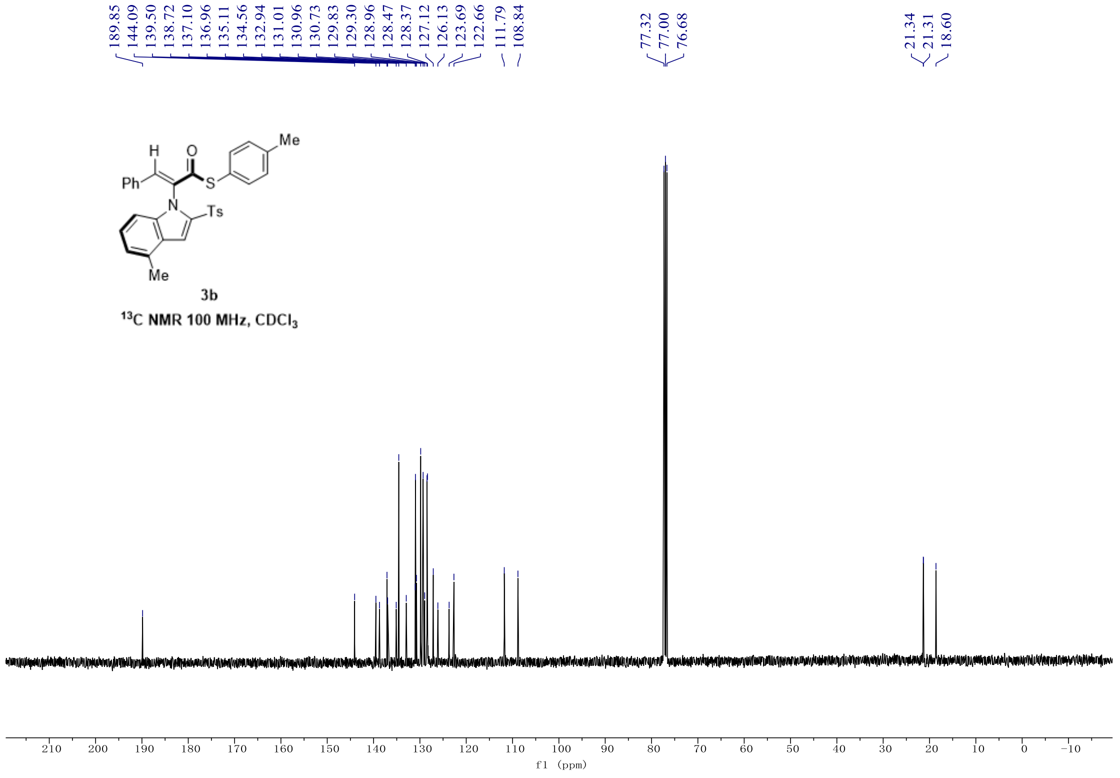
189.85 144.09 139.50 138.72 137.10 136.96 135.11 134.56 132.94 131.01 130.96 130.73 129.83 129.30 128.96 128.47 128.37 127.12 126.13 123.69 122.66 111.79 108.84 77.32 77.00 76.68 21.34 21.31 18.60
Me
H
O
S
Ph
N
Ts
Me
3b
13C NMR 100 MHz, CDCl3
210 200 190 180 170 160 150 140 130 120 110 100 90 80 70 60 50 40 30 20 10 0 −10
f1 (ppm)

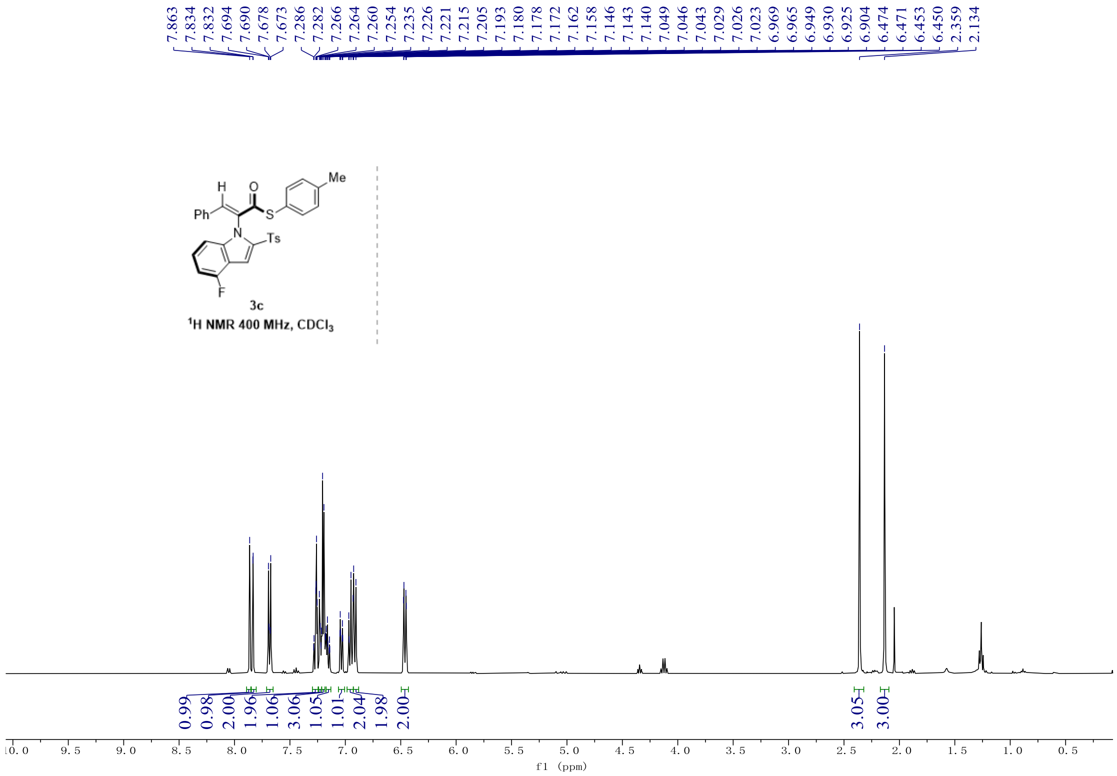
3c
1H NMR 400 MHz, CDCl3
f1 (ppm)

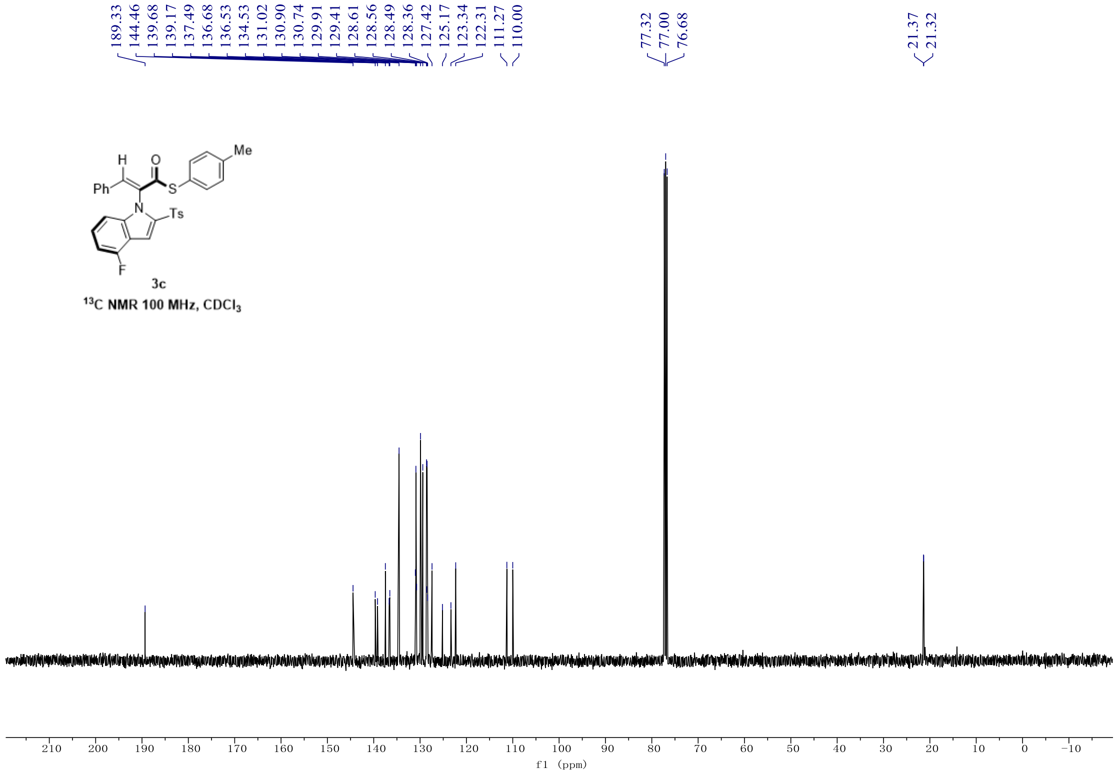
3c
13C NMR 100 MHz, CDCl3
f1 (ppm)

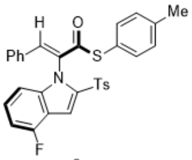

3c

^{19}F NMR 376 MHz，CDCl$_3$

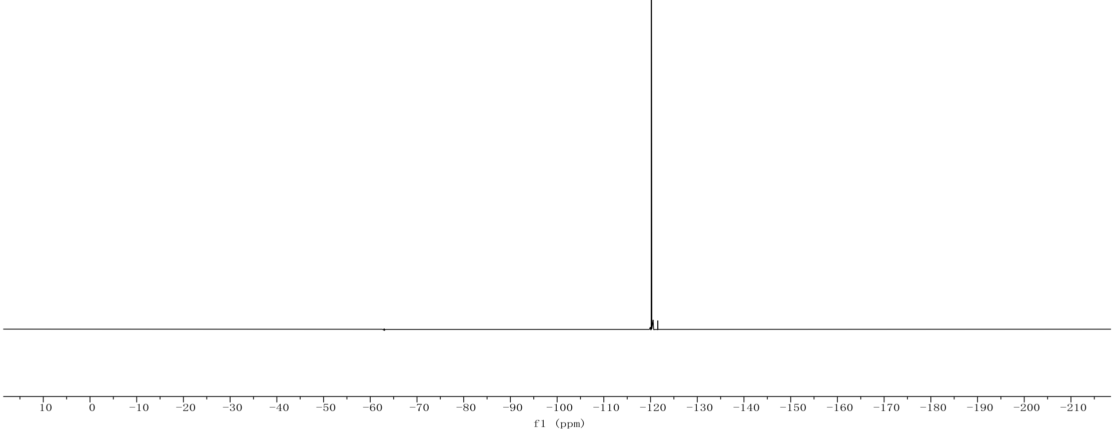

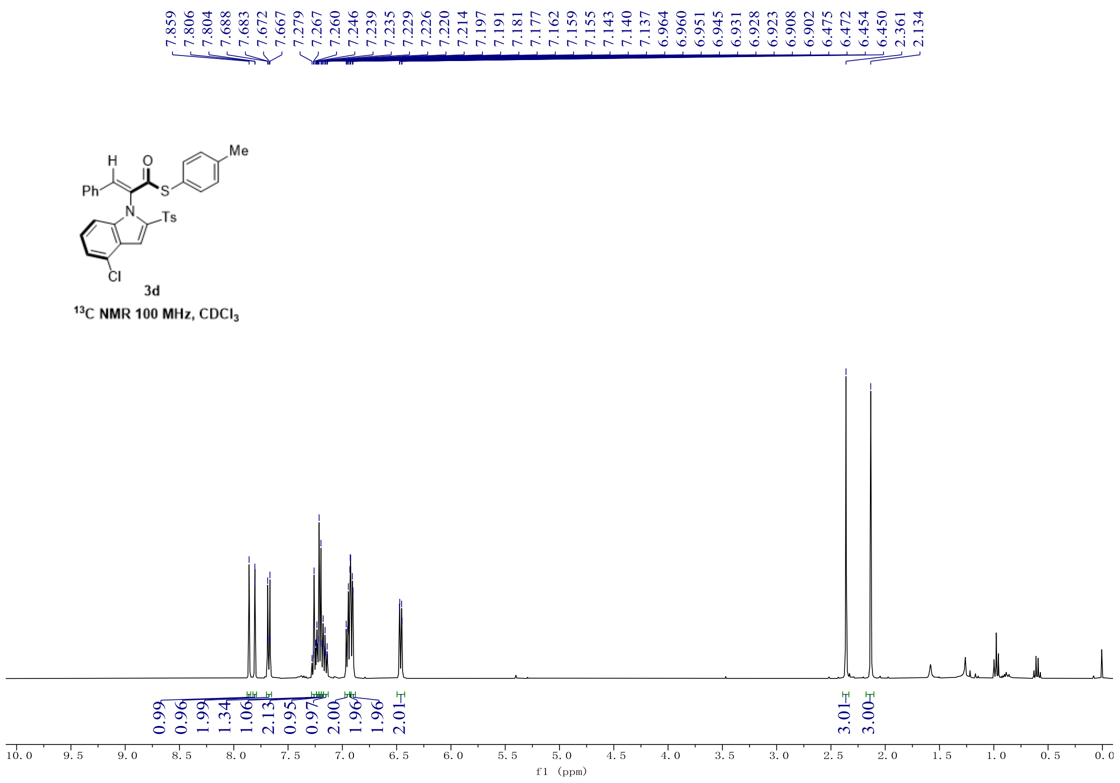
7.859 7.806 7.804 7.688 7.683 7.672 7.667 7.279 7.267 7.260 7.246 7.239 7.235 7.229 7.226 7.220 7.214 7.197 7.191 7.181 7.177 7.162 7.159 7.155 7.143 7.140 7.137 6.964 6.960 6.951 6.945 6.931 6.928 6.923 6.908 6.902 6.475 6.472 6.454 6.450 2.361 2.134
H
O
Me
Ph
S
N
Ts
Cl
3d
^{13}C NMR 100 MHz, CDCl$_3$
0.99 0.96 1.99 1.34 1.06 2.13 0.95 0.97 2.00 1.96 1.96 2.01 3.01 3.00
f1 (ppm)

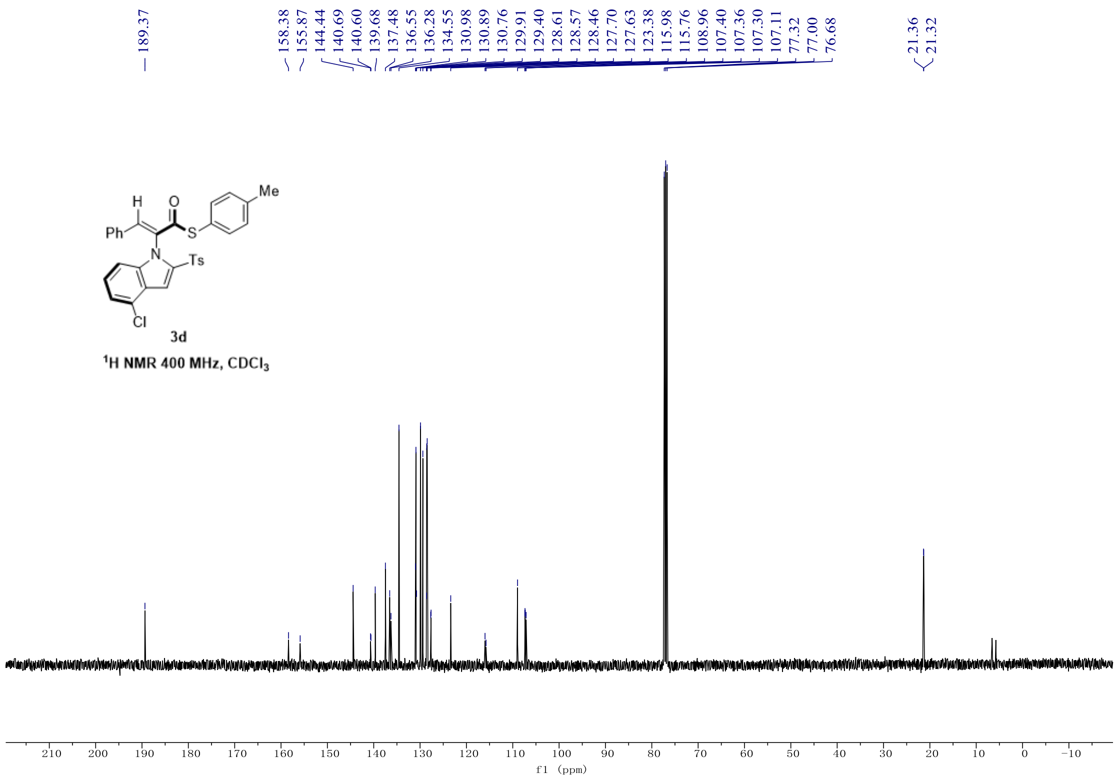
189.37
158.38 155.87 144.44 140.69 140.60 139.68 137.48 136.55 136.28 134.55 130.98 130.89 130.76 129.91 129.40 128.61 128.57 128.46 127.70 127.63 123.38 115.98 115.76 108.96 107.40 107.36 107.30 107.11 77.32 77.00 76.68
21.36 21.32
H
O
Me
Ph
S
N
Ts
Cl
3d
^{1}H NMR 400 MHz, CDCl$_3$
f1 (ppm)

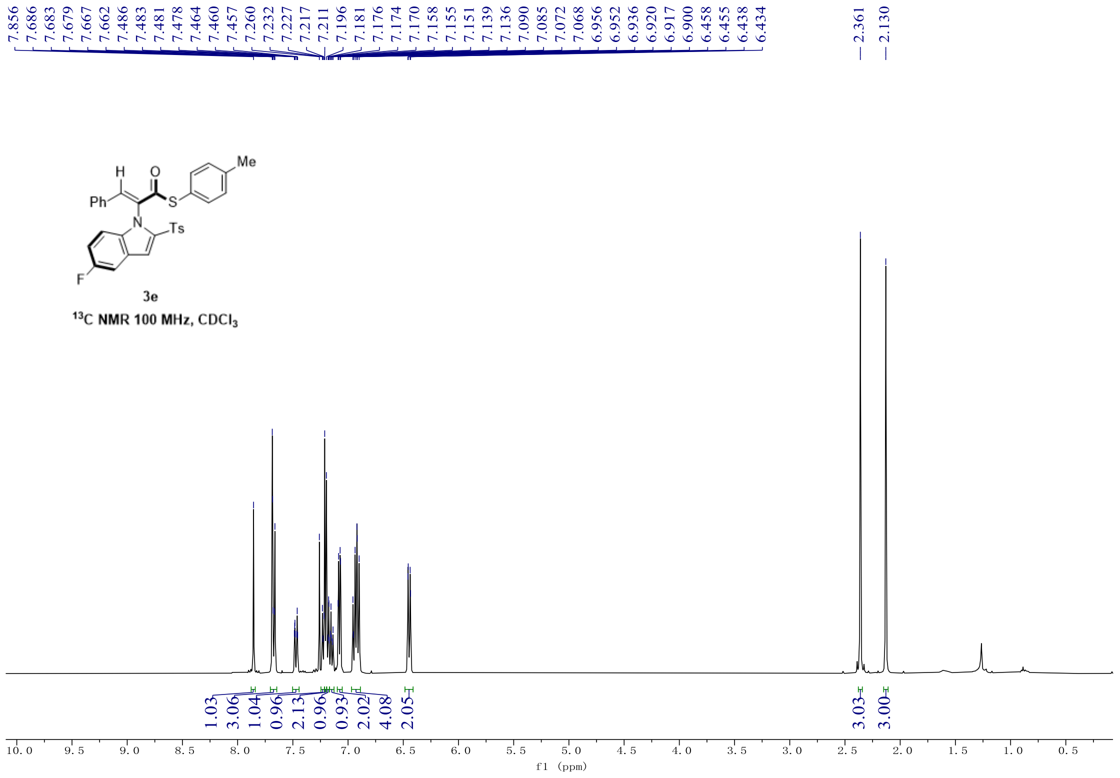
7.856 7.686 7.683 7.679 7.667 7.662 7.486 7.483 7.481 7.478 7.464 7.460 7.457 7.260 7.232 7.227 7.217 7.211 7.196 7.181 7.176 7.174 7.170 7.158 7.155 7.151 7.139 7.136 7.090 7.085 7.072 7.068 6.956 6.952 6.936 6.920 6.917 6.900 6.458 6.455 6.438 6.434
2.361 2.130
H
O
Me
Ph
S
N
Ts
F
3e
^{13}C NMR 100 MHz, CDCl$_3$
1.03 3.06 1.04 0.96 2.13 0.96 0.93 2.02 4.08 2.05
3.03 3.00
10.0 9.5 9.0 8.5 8.0 7.5 7.0 6.5 6.0 5.5 5.0 4.5 4.0 3.5 3.0 2.5 2.0 1.5 1.0 0.5
f1 (ppm)

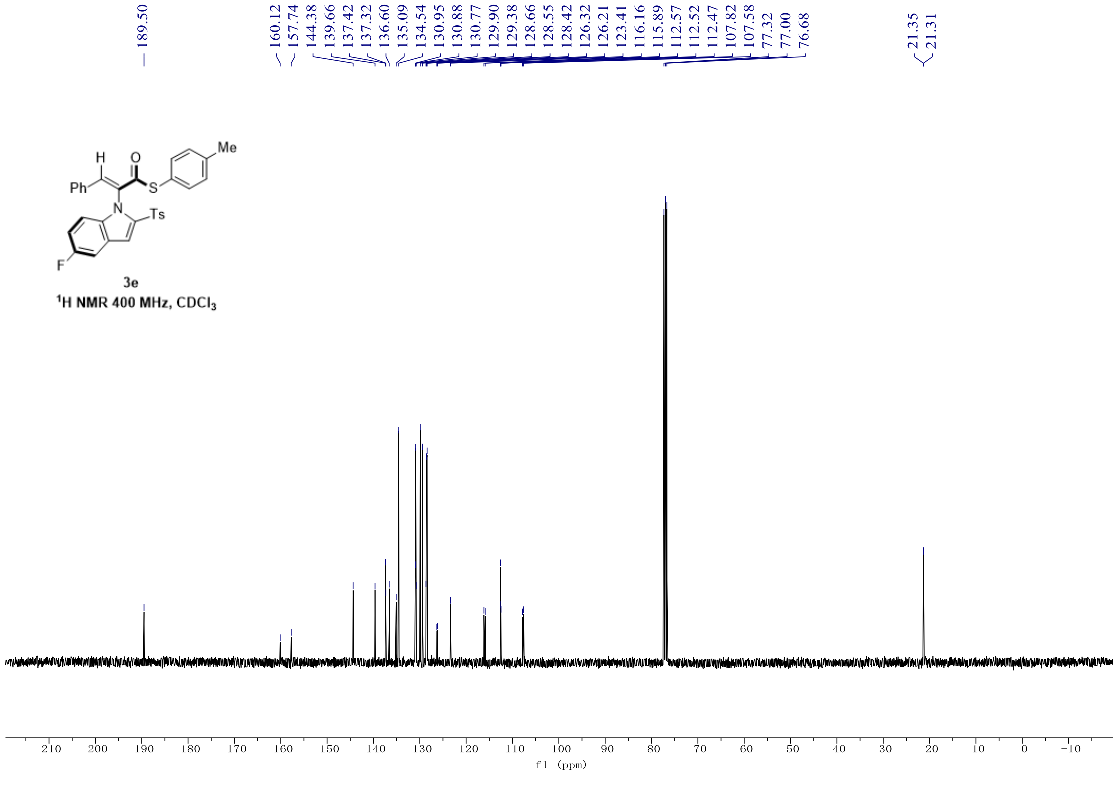
189.50
160.12 157.74 144.38 139.66 137.42 137.32 136.60 135.09 134.54 130.95 130.88 130.77 129.90 129.38 128.66 128.55 128.42 126.32 126.21 123.41 116.16 115.89 112.57 112.52 112.47 107.82 107.58 77.32 77.00 76.68
21.35 21.31
H
O
Me
Ph
S
N
Ts
F
3e
^{1}H NMR 400 MHz, CDCl$_3$
210 200 190 180 170 160 150 140 130 120 110 100 90 80 70 60 50 40 30 20 10 0 −10
f1 (ppm)

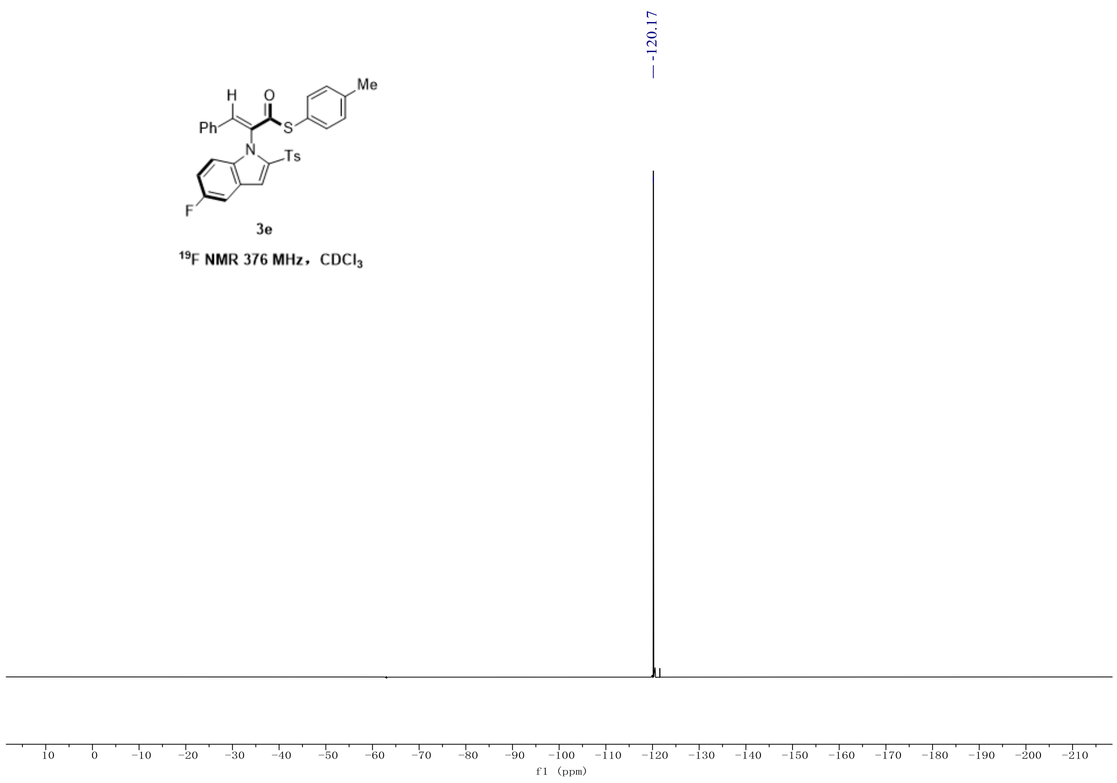
-120.17
H
O
Me
Ph
S
N
Ts
F
3e
^{19}F NMR 376 MHz，CDCl$_3$
10
0
−10
−20
−30
−40
−50
−60
−70
−80
−90
−100
−110
−120
−130
−140
−150
−160
−170
−180
−190
−200
−210
f1 (ppm)

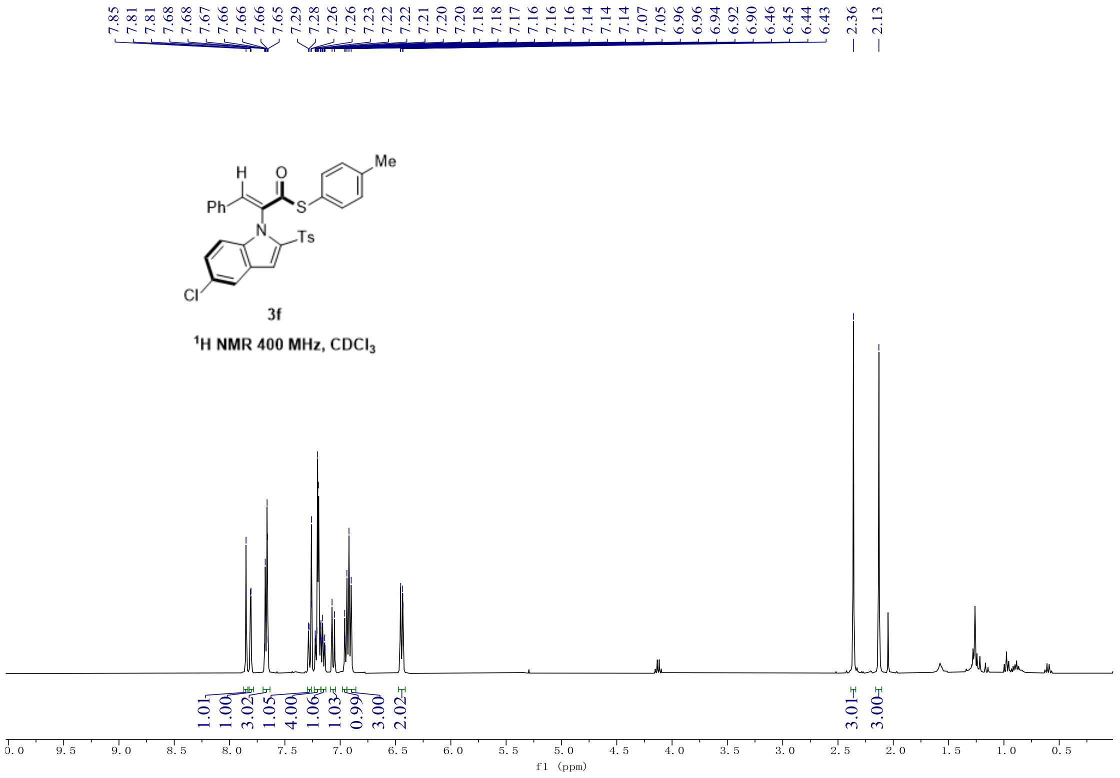
7.85 7.81 7.81 7.68 7.67 7.66 7.66 7.65 7.29 7.28 7.26 7.26 7.23 7.22 7.22 7.21 7.20 7.18 7.18 7.17 7.16 7.16 7.14 7.14 7.07 7.05 6.96 6.96 6.94 6.92 6.90 6.46 6.45 6.44 6.43
2.36
2.13
H
O
Me
Ph
S
N
Ts
Cl
3f
1H NMR 400 MHz, CDCl3
1.01 1.00 3.02 1.05 4.00 1.06 1.03 0.99 3.00 2.02
3.01 3.00
f1 (ppm)

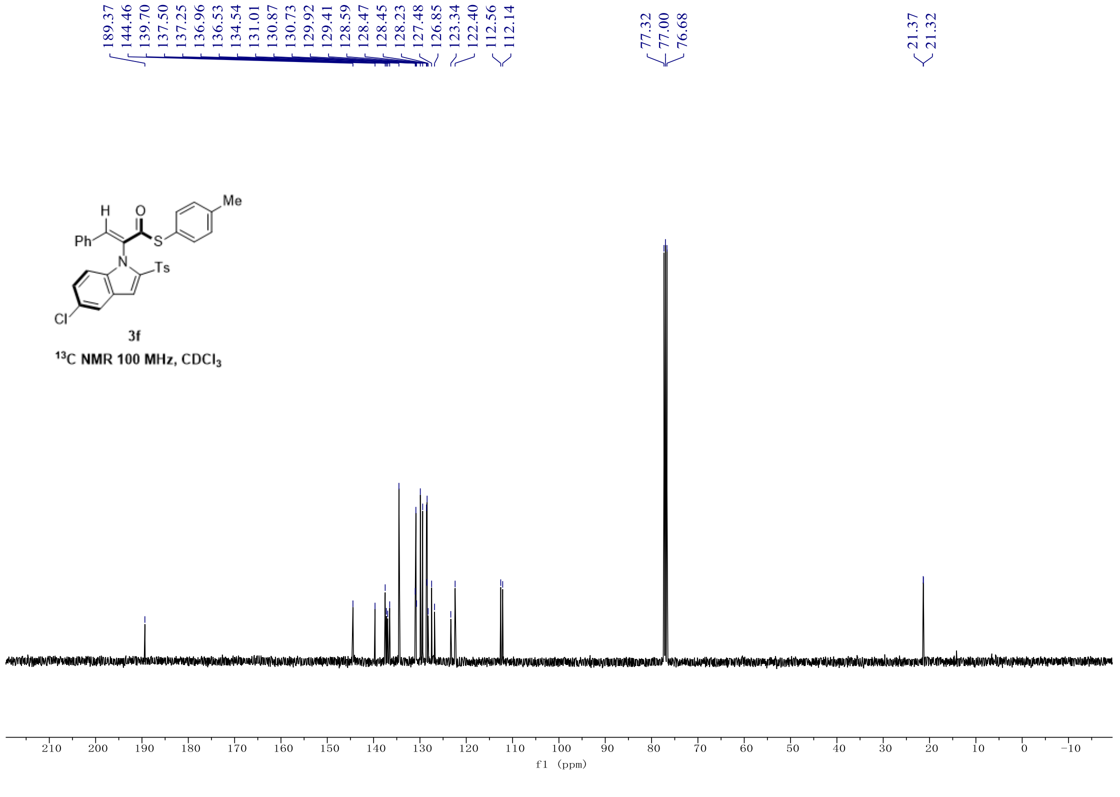
189.37 144.46 139.70 137.50 137.25 136.96 136.53 134.54 131.01 130.87 130.73 129.92 129.41 128.59 128.47 128.45 128.23 127.48 126.85 123.34 122.40 112.56 112.14
77.32 77.00 76.68
21.37 21.32
H
O
Me
Ph
S
N
Ts
Cl
3f
13C NMR 100 MHz, CDCl3
f1 (ppm)

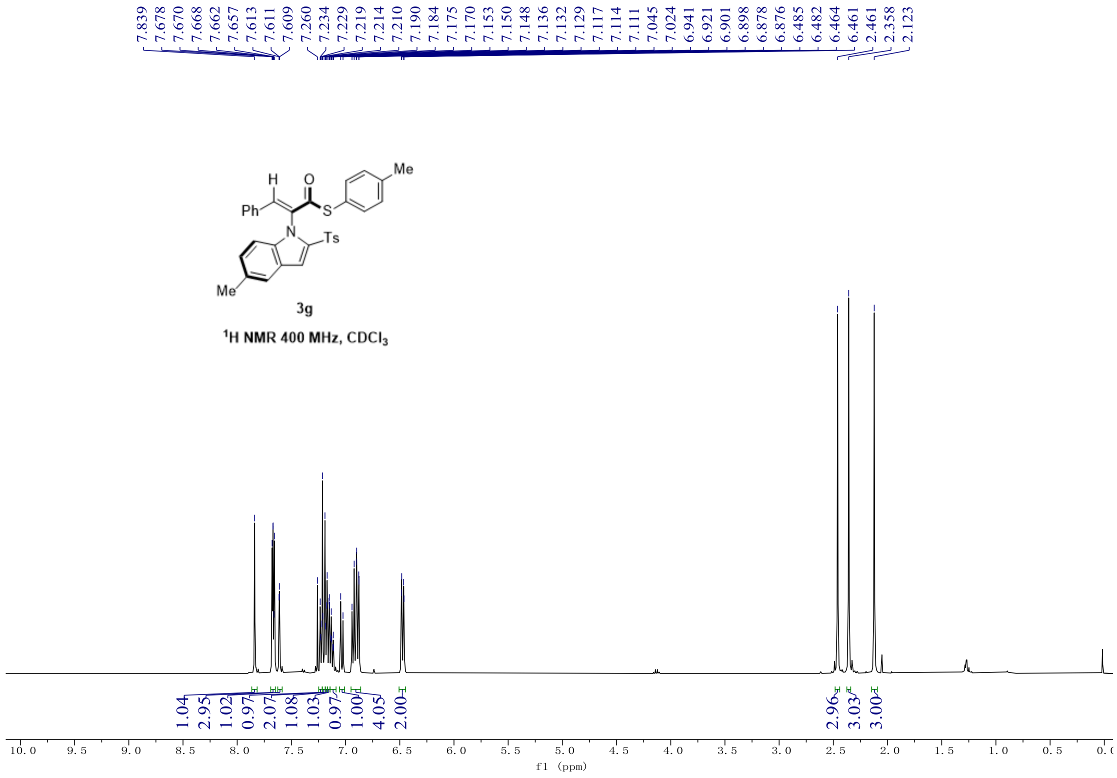
7.839 7.678 7.670 7.668 7.662 7.657 7.613 7.611 7.609 7.260 7.234 7.229 7.219 7.214 7.210 7.190 7.184 7.175 7.170 7.153 7.150 7.148 7.136 7.132 7.129 7.117 7.114 7.111 7.045 7.024 6.941 6.921 6.901 6.898 6.878 6.876 6.485 6.482 6.464 6.461 2.461 2.358 2.123
H
O
Ph
S
Me
N
Ts
Me
3g
1H NMR 400 MHz, CDCl3
1.04 2.95 1.02 0.97 2.07 1.08 1.03 0.97 1.00 4.05 2.00
2.96 3.03 3.00
10.0 9.5 9.0 8.5 8.0 7.5 7.0 6.5 6.0 5.5 5.0 4.5 4.0 3.5 3.0 2.5 2.0 1.5 1.0 0.5 0.0
f1 (ppm)

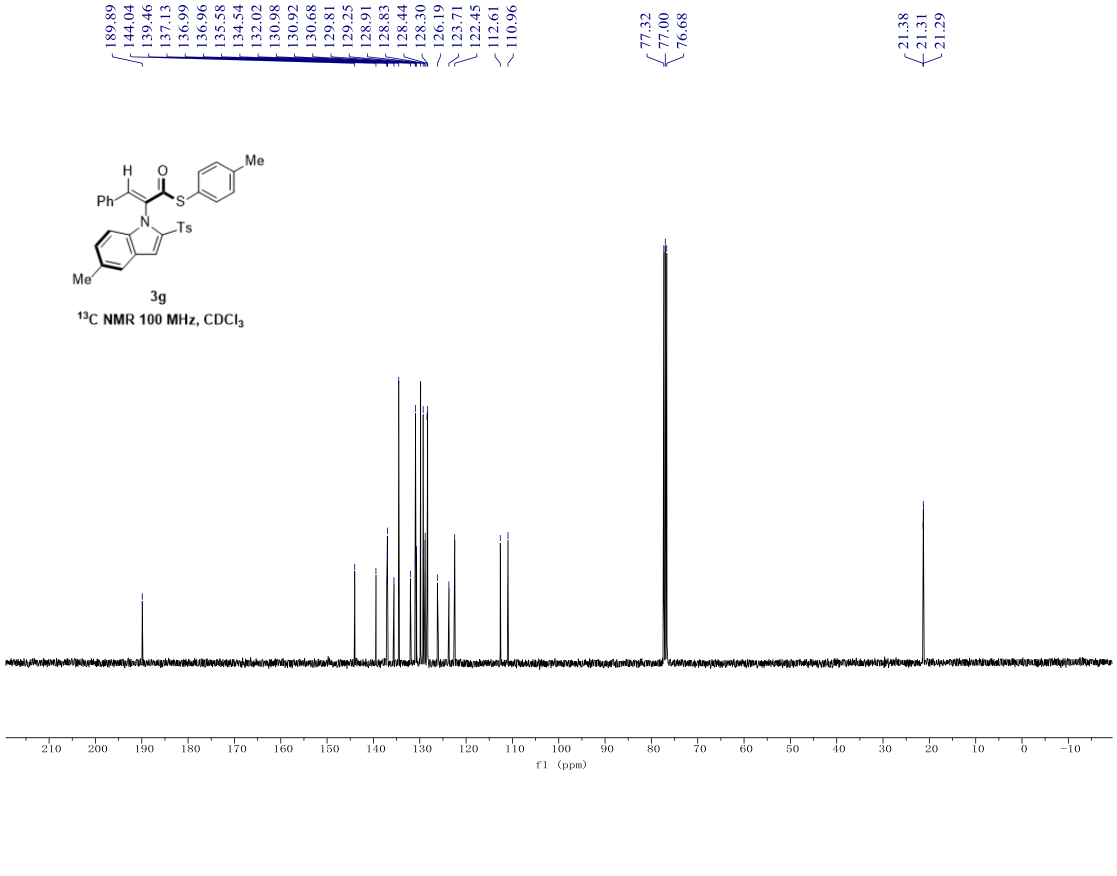
189.89 144.04 139.46 137.13 136.99 136.96 135.58 134.54 132.02 130.98 130.92 130.68 129.81 129.25 128.91 128.83 128.44 128.30 126.19 123.71 122.45 112.61 110.96
77.32 77.00 76.68
21.38 21.31 21.29
H
O
Ph
S
Me
N
Ts
Me
3g
13C NMR 100 MHz, CDCl3
210 200 190 180 170 160 150 140 130 120 110 100 90 80 70 60 50 40 30 20 10 0 −10
f1 (ppm)

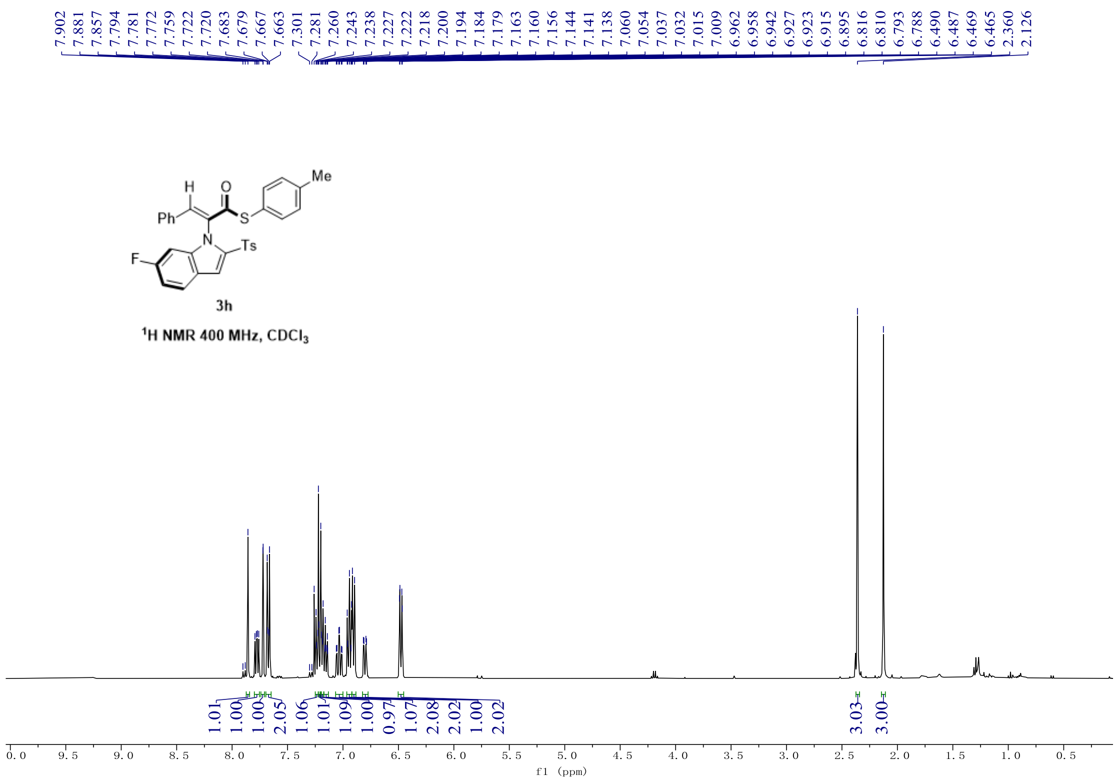
Me
H
O
Ph
S
N
Ts
F
3h
1H NMR 400 MHz, CDCl3
f1 (ppm)

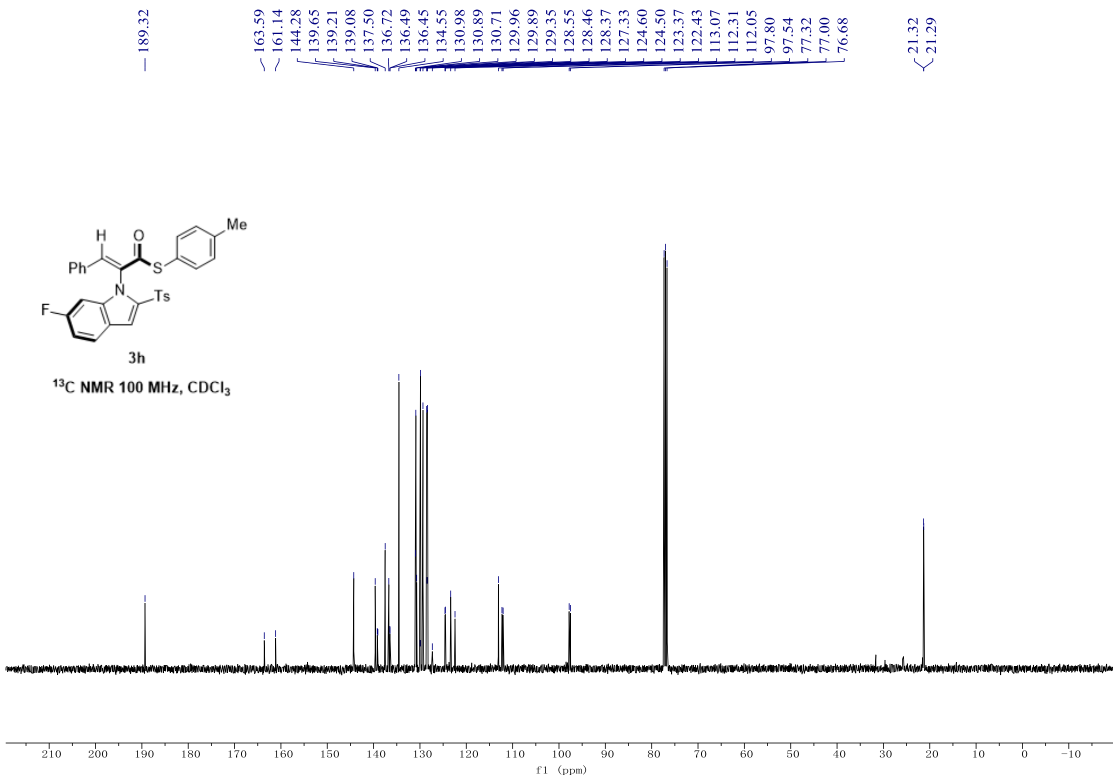
Me
H
O
Ph
S
N
Ts
F
3h
13C NMR 100 MHz, CDCl3
f1 (ppm)

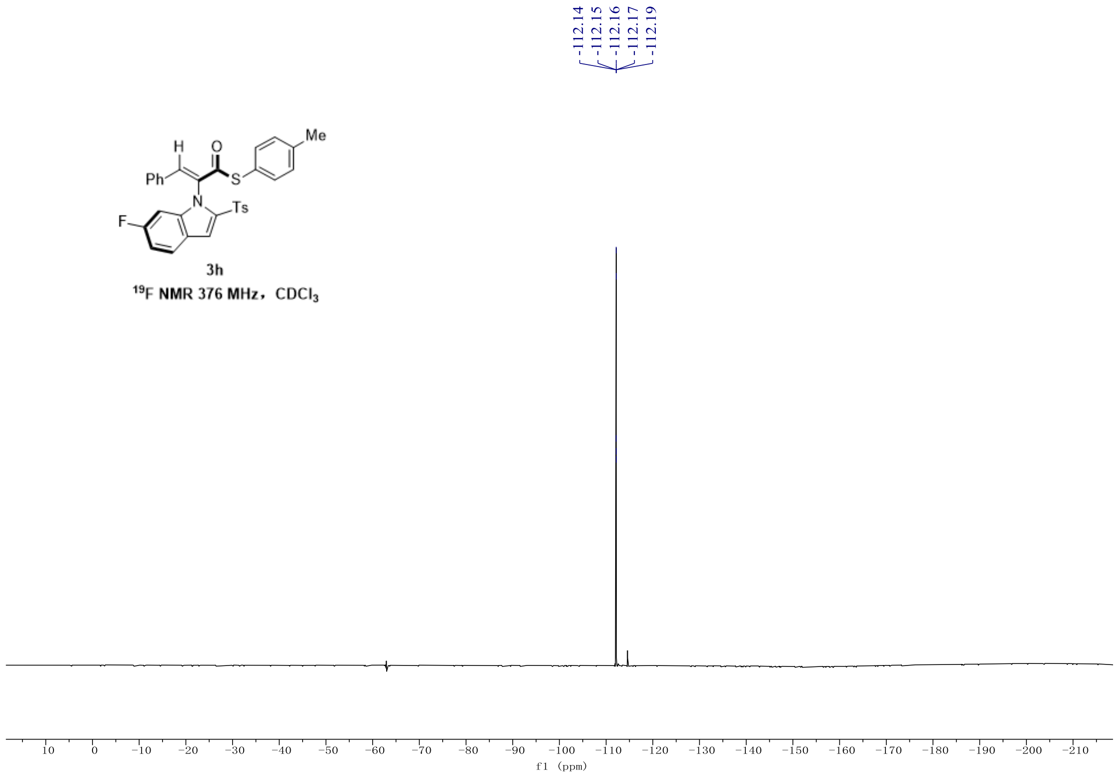
-112.14
-112.15
-112.16
-112.17
-112.19
Me
H
O
S
Ph
N
Ts
F
3h
19F NMR 376 MHz，CDCl3
10 0 −10 −20 −30 −40 −50 −60 −70 −80 −90 −100 −110 −120 −130 −140 −150 −160 −170 −180 −190 −200 −210
f1 (ppm)

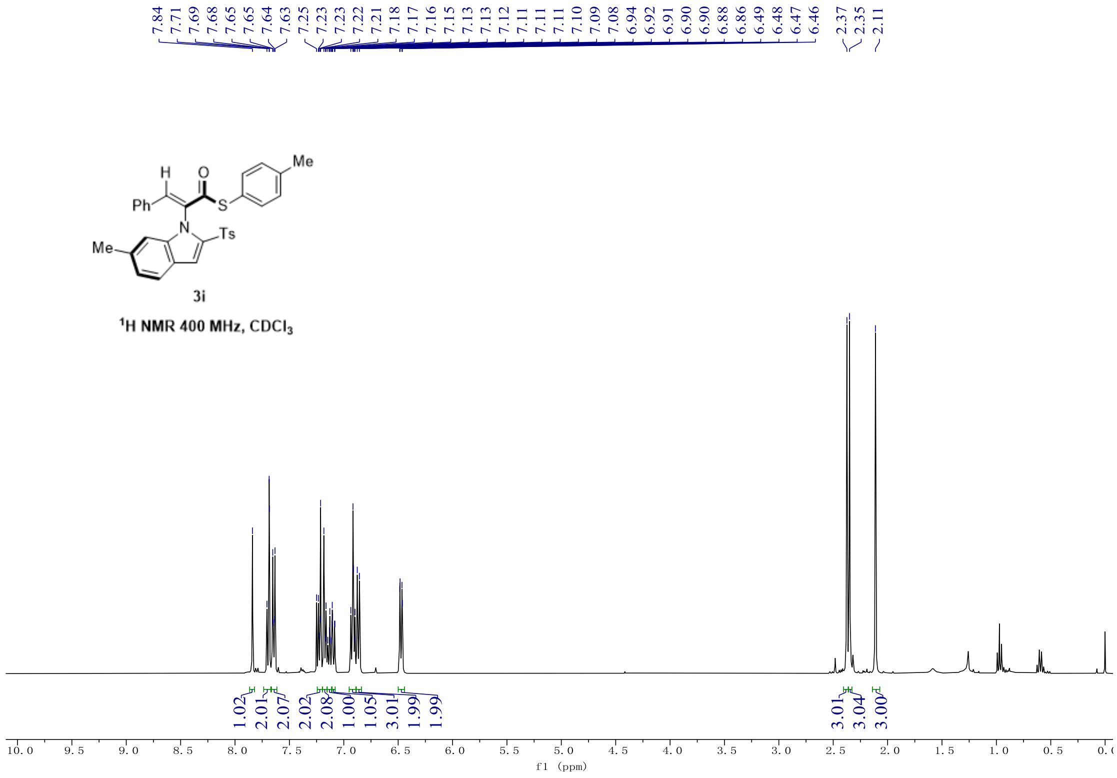
7.84 7.71 7.69 7.68 7.65 7.65 7.64 7.63 7.25 7.23 7.23 7.22 7.21 7.18 7.17 7.16 7.15 7.13 7.13 7.12 7.11 7.11 7.11 7.10 7.09 7.08 6.94 6.92 6.91 6.90 6.90 6.88 6.86 6.49 6.48 6.47 6.46 2.37 2.35 2.11
H
O
Me
Ph
S
N
Ts
Me
3i
^{1}H NMR 400 MHz, CDCl$_3$
1.02 2.01 2.07 2.02 2.08 1.00 1.05 3.01 1.99 1.99 3.01 3.04 3.00
10.0 9.5 9.0 8.5 8.0 7.5 7.0 6.5 6.0 5.5 5.0 4.5 4.0 3.5 3.0 2.5 2.0 1.5 1.0 0.5
f1 (ppm)

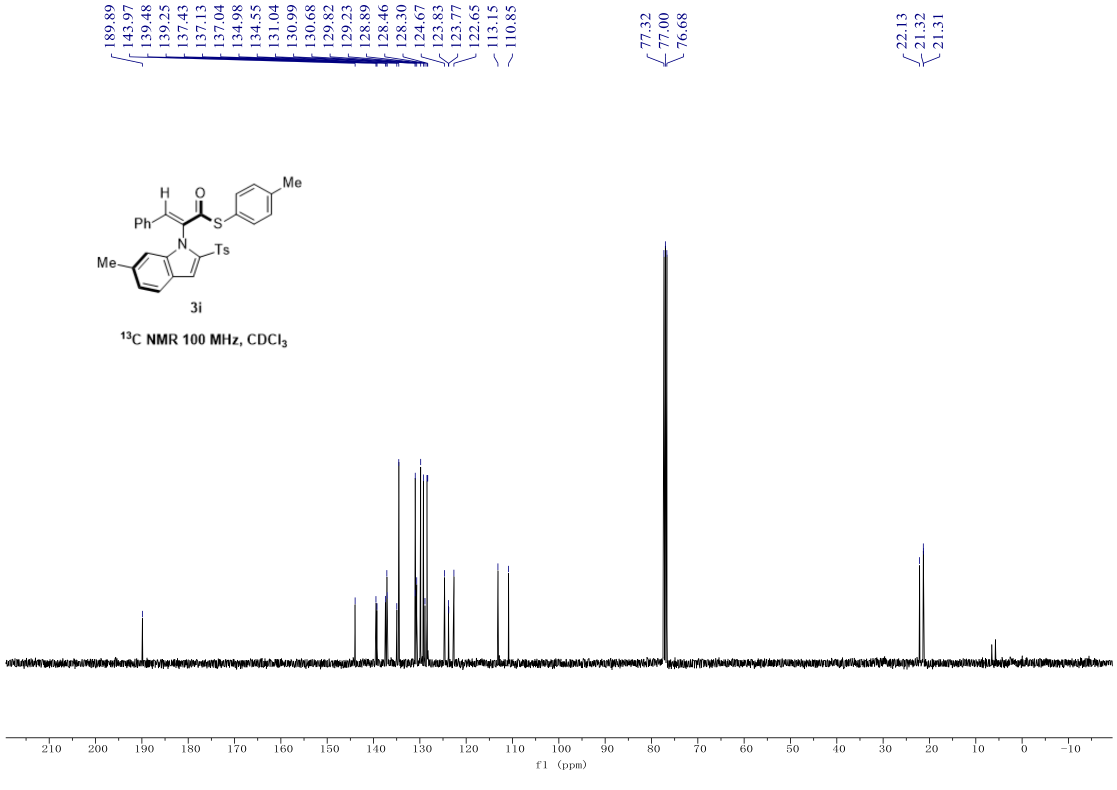
189.89 143.97 139.48 139.25 137.43 137.13 137.04 134.98 134.55 131.04 130.99 130.68 129.82 129.23 128.89 128.46 128.30 124.67 123.83 123.77 122.65 113.15 110.85 77.32 77.00 76.68 22.13 21.32 21.31
H
O
Me
Ph
S
N
Ts
Me
3i
^{13}C NMR 100 MHz, CDCl$_3$
210 200 190 180 170 160 150 140 130 120 110 100 90 80 70 60 50 40 30 20 10 0 −10
f1 (ppm)

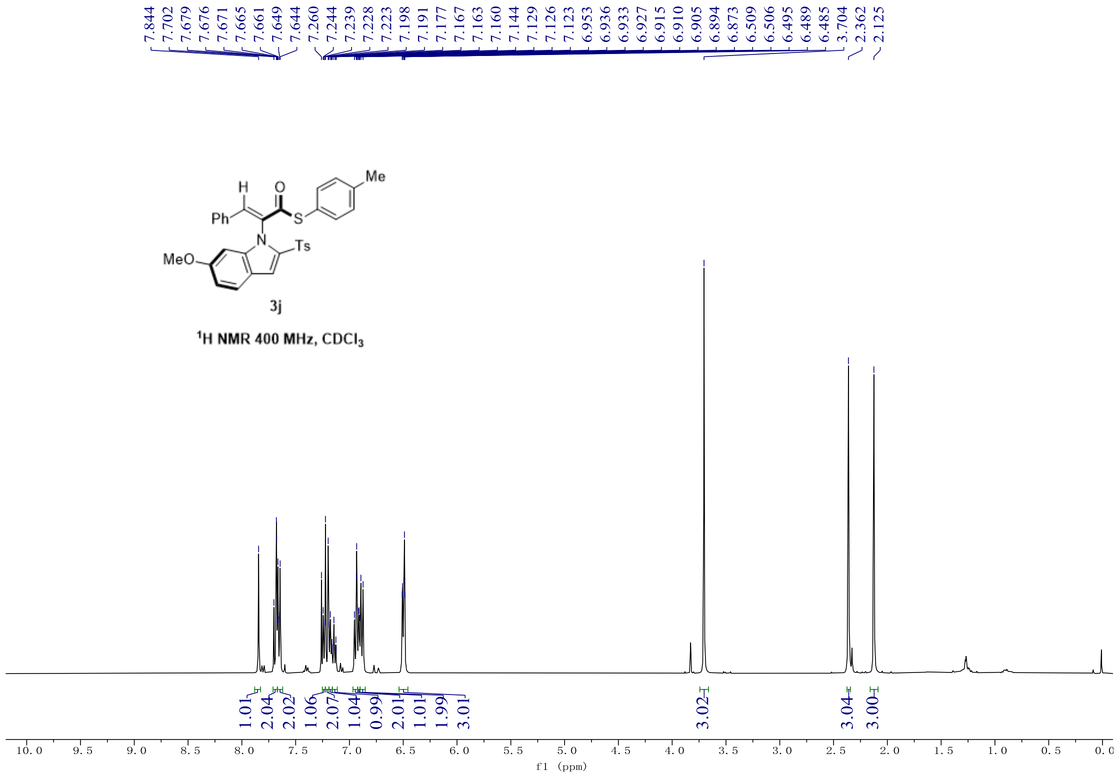
7.844
7.702
7.679
7.676
7.671
7.665
7.661
7.649
7.644
7.260
7.244
7.239
7.228
7.223
7.198
7.191
7.177
7.167
7.163
7.160
7.144
7.129
7.126
7.123
6.953
6.936
6.933
6.927
6.915
6.910
6.905
6.894
6.873
6.509
6.506
6.495
6.489
6.485
3.704
2.362
2.125
H
O
Me
Ph
S
N
Ts
MeO
3j
^{1}H NMR 400 MHz, CDCl$_3$
1.01
2.04
2.02
1.06
2.07
1.04
0.99
2.01
1.01
1.99
3.01
3.02
3.04
3.00
10.0
9.5
9.0
8.5
8.0
7.5
7.0
6.5
6.0
5.5
5.0
4.5
4.0
3.5
3.0
2.5
2.0
1.5
1.0
0.5
0.0
f1 (ppm)

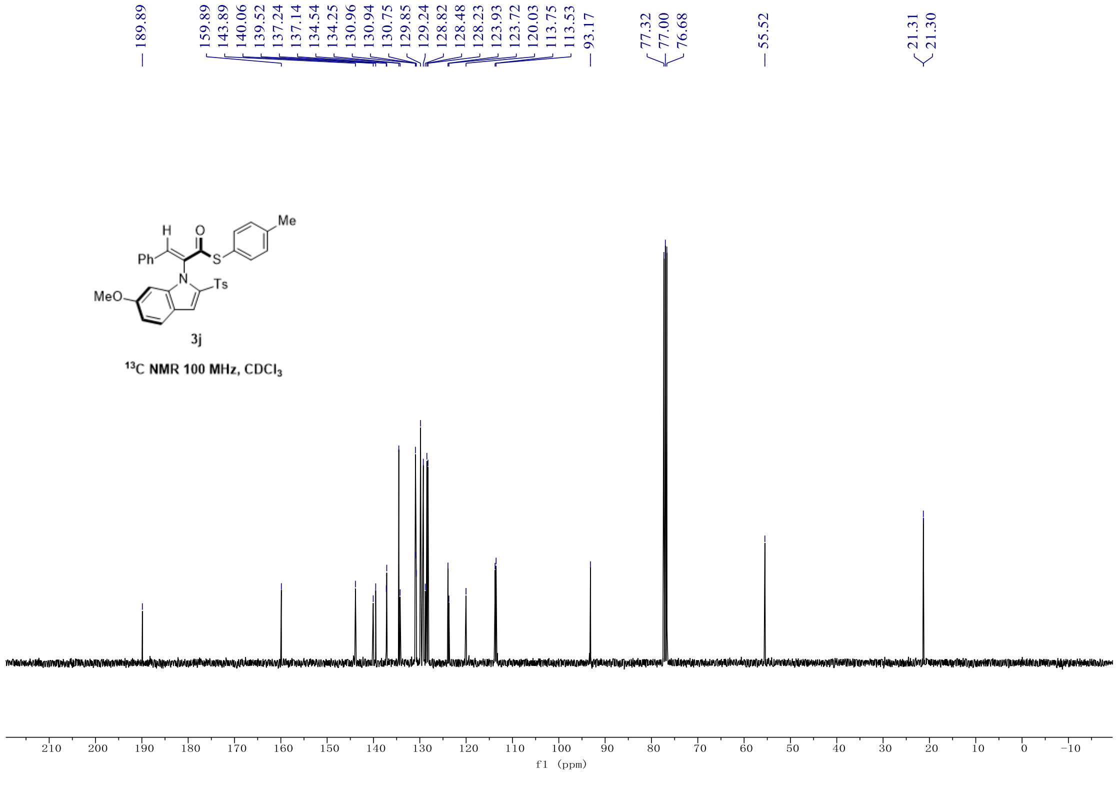
189.89
159.89
143.89
140.06
139.52
137.24
137.14
134.54
134.25
130.96
130.94
130.75
129.85
129.24
128.82
128.48
128.23
123.93
123.72
120.03
113.75
113.53
93.17
77.32
77.00
76.68
55.52
21.31
21.30
H
O
Me
Ph
S
N
Ts
MeO
3j
^{13}C NMR 100 MHz, CDCl$_3$
210
200
190
180
170
160
150
140
130
120
110
100
90
80
70
60
50
40
30
20
10
0
−10
f1 (ppm)

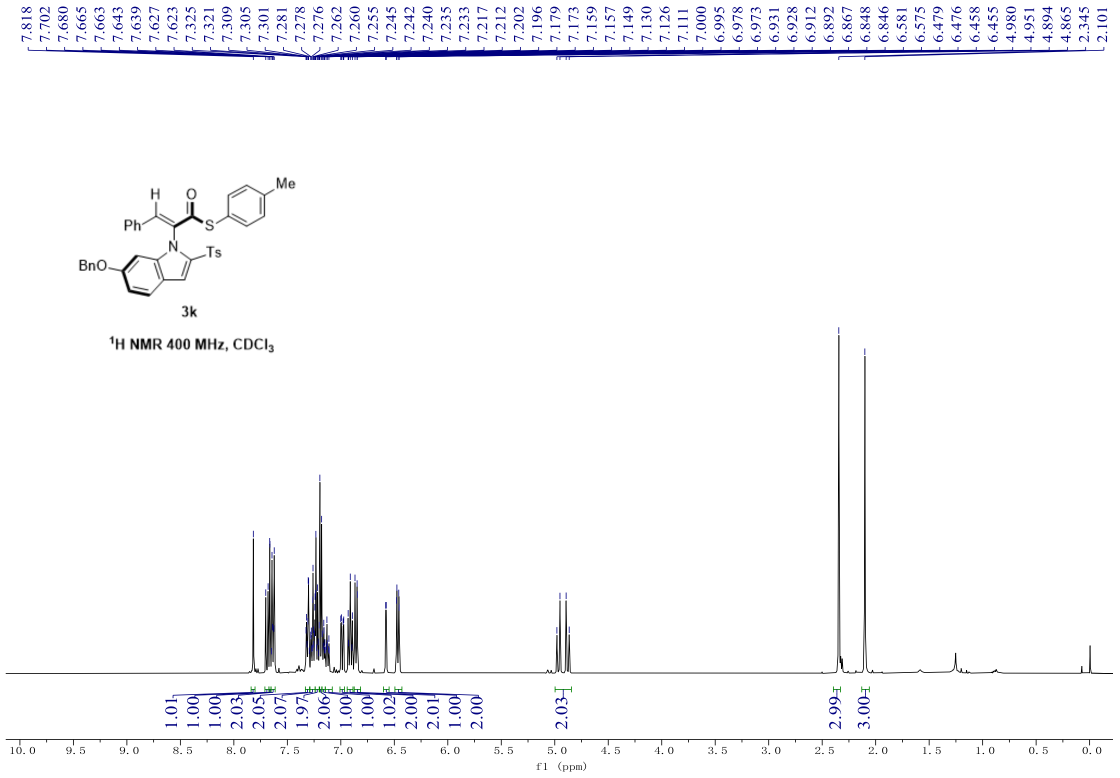
7.818 7.702 7.680 7.665 7.663 7.643 7.639 7.627 7.623 7.325 7.321 7.309 7.305 7.301 7.281 7.278 7.276 7.262 7.260 7.255 7.245 7.242 7.240 7.235 7.233 7.217 7.212 7.202 7.196 7.179 7.173 7.159 7.157 7.149 7.130 7.126 7.111 7.000 6.995 6.978 6.973 6.931 6.928 6.912 6.892 6.867 6.848 6.846 6.581 6.575 6.479 6.476 6.458 6.455 4.980 4.951 4.894 4.865 2.345 2.101
H
O
Me
Ph
S
N
Ts
BnO
3k
1H NMR 400 MHz, CDCl3
1.01 1.00 1.00 2.03 2.05 2.07 1.97 2.06 1.00 1.00 1.02 2.00 2.01 1.00 2.00 2.03 2.99 3.00
10.0 9.5 9.0 8.5 8.0 7.5 7.0 6.5 6.0 5.5 5.0 4.5 4.0 3.5 3.0 2.5 2.0 1.5 1.0 0.5 0.0
f1 (ppm)

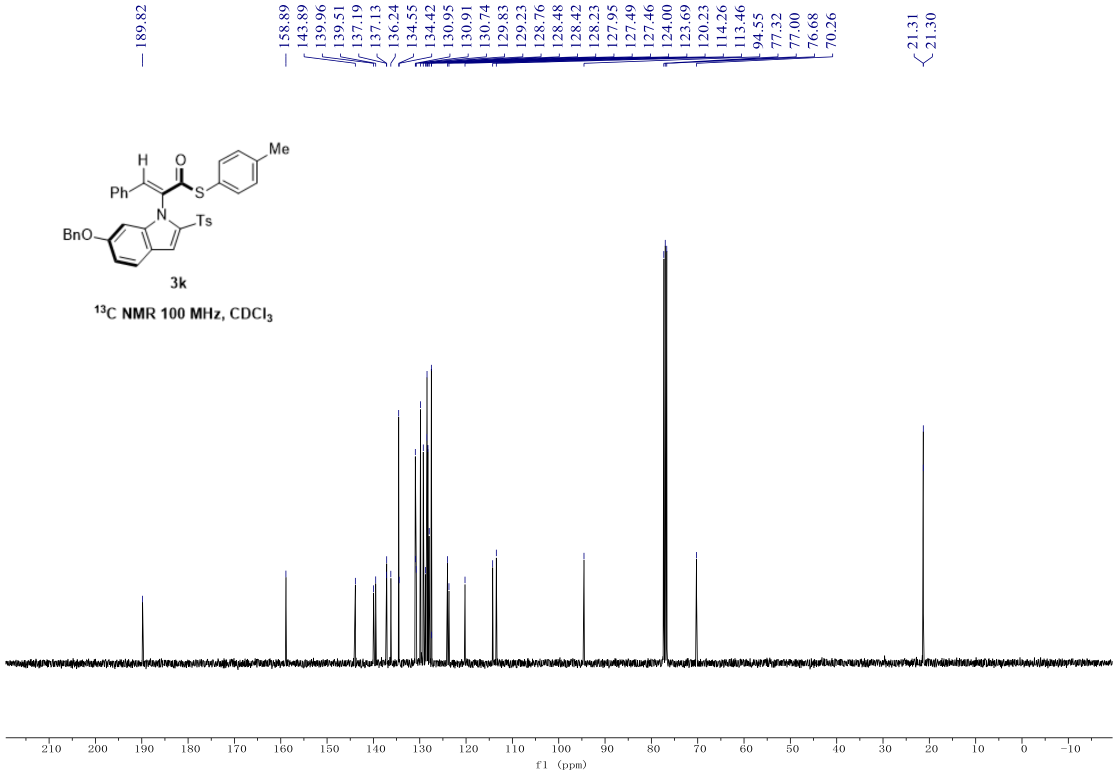
189.82 158.89 143.89 139.96 139.51 137.19 137.13 136.24 134.55 134.42 130.95 130.91 130.74 129.83 129.23 128.76 128.48 128.42 128.23 127.95 127.49 127.46 124.00 123.69 120.23 114.26 113.46 94.55 77.32 77.00 76.68 70.26 21.31 21.30
H
O
Me
Ph
S
N
Ts
BnO
3k
13C NMR 100 MHz, CDCl3
210 200 190 180 170 160 150 140 130 120 110 100 90 80 70 60 50 40 30 20 10 0 -10
f1 (ppm)

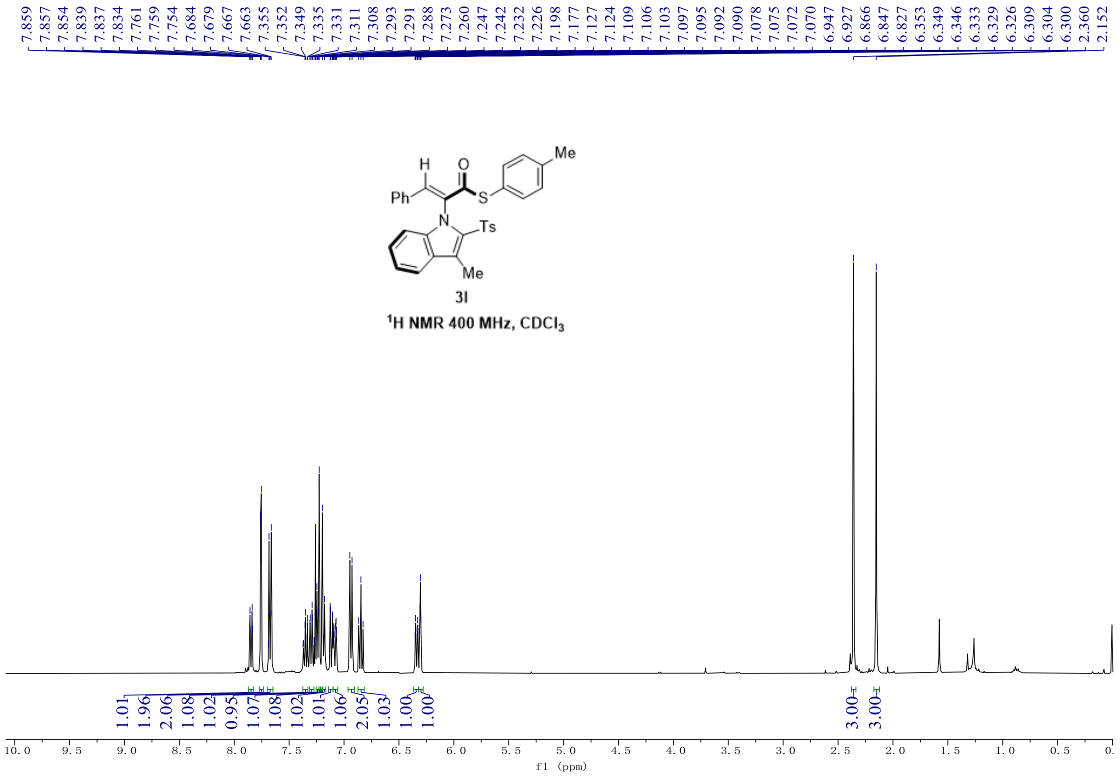
7.859 7.857 7.854 7.839 7.837 7.834 7.761 7.759 7.754 7.684 7.679 7.667 7.663 7.355 7.352 7.349 7.335 7.331 7.311 7.308 7.293 7.291 7.288 7.273 7.260 7.247 7.242 7.232 7.226 7.198 7.177 7.127 7.124 7.109 7.106 7.103 7.097 7.095 7.092 7.090 7.078 7.075 7.072 7.070 6.947 6.927 6.866 6.847 6.827 6.353 6.349 6.346 6.333 6.329 6.326 6.309 6.304 6.300 2.360 2.152
H
O
S
Me
Ph
N
Ts
Me
3l
¹H NMR 400 MHz, CDCl₃
1.01 1.96 2.06 1.08 1.02 0.95 1.07 1.08 1.02 1.01 1.06 2.05 1.03 1.00 1.00
3.00 3.00
10.0 9.5 9.0 8.5 8.0 7.5 7.0 6.5 6.0 5.5 5.0 4.5 4.0 3.5 3.0 2.5 2.0 1.5 1.0 0.5 0.
f1 (ppm)

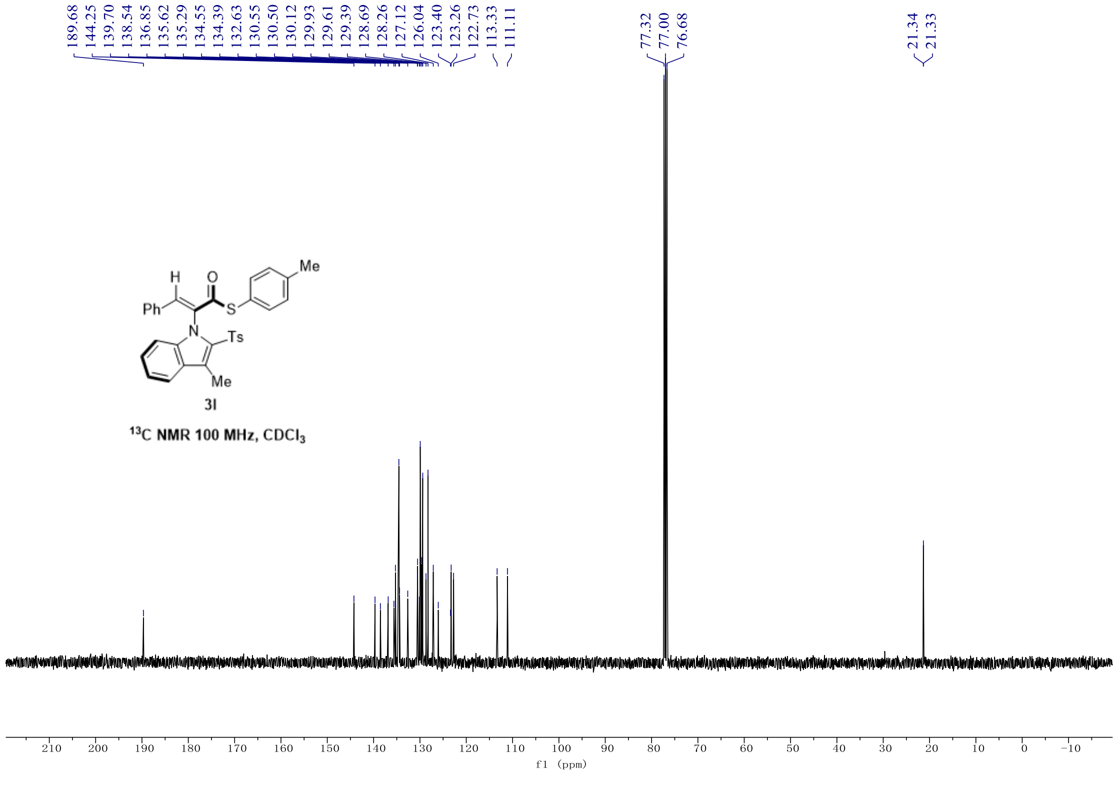
189.68 144.25 139.70 138.54 136.85 135.62 135.29 134.55 134.39 132.63 130.55 130.50 130.12 129.93 129.61 129.39 128.69 128.26 127.12 126.04 123.40 123.26 122.73 113.33 111.11
77.32 77.00 76.68
21.34 21.33
H
O
S
Me
Ph
N
Ts
Me
3l
¹³C NMR 100 MHz, CDCl₃
210 200 190 180 170 160 150 140 130 120 110 100 90 80 70 60 50 40 30 20 10 0 −10
f1 (ppm)

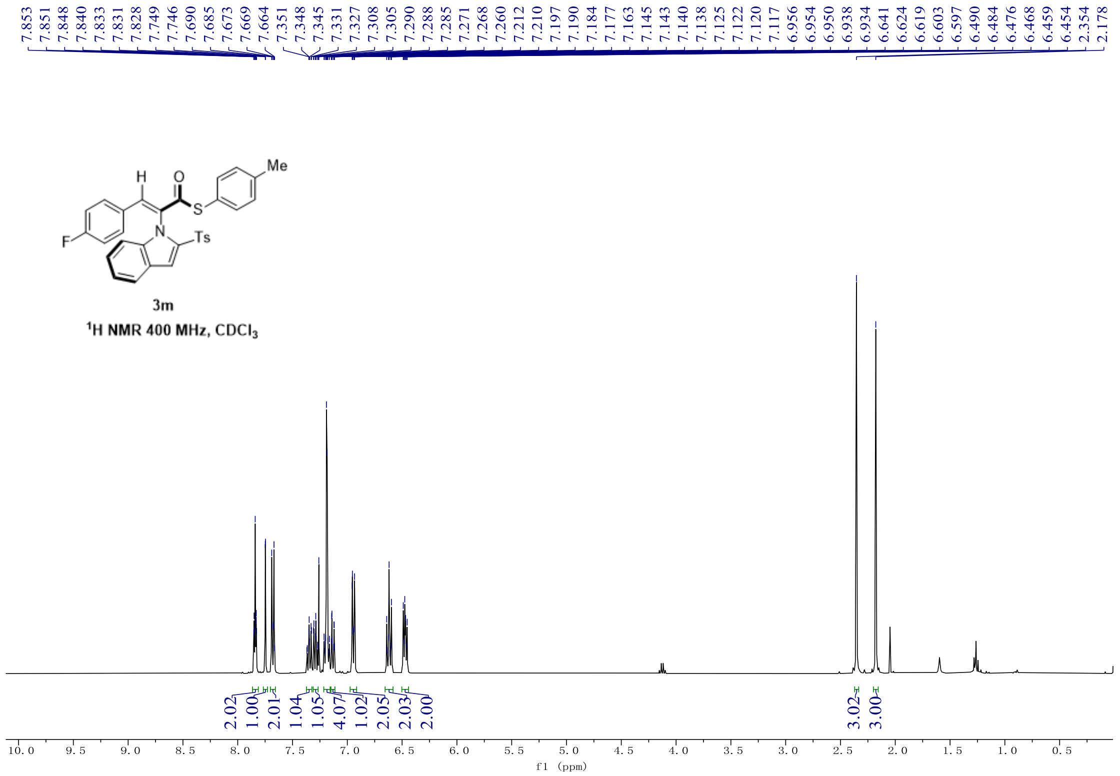
7.853 7.851 7.848 7.840 7.833 7.831 7.828 7.749 7.746 7.690 7.685 7.673 7.669 7.664 7.351 7.348 7.345 7.331 7.327 7.308 7.305 7.290 7.288 7.285 7.271 7.268 7.260 7.212 7.210 7.197 7.190 7.184 7.177 7.163 7.145 7.143 7.140 7.138 7.125 7.122 7.120 7.117 6.956 6.954 6.950 6.938 6.934 6.641 6.624 6.619 6.603 6.597 6.490 6.484 6.476 6.468 6.459 6.454 2.354 2.178
H
O
S
Me
F
N
Ts
3m
1H NMR 400 MHz, CDCl3
2.02 1.00 2.01 1.04 1.05 4.07 1.02 2.05 2.03 2.00
3.02 3.00
10.0 9.5 9.0 8.5 8.0 7.5 7.0 6.5 6.0 5.5 5.0 4.5 4.0 3.5 3.0 2.5 2.0 1.5 1.0 0.5
f1 (ppm)

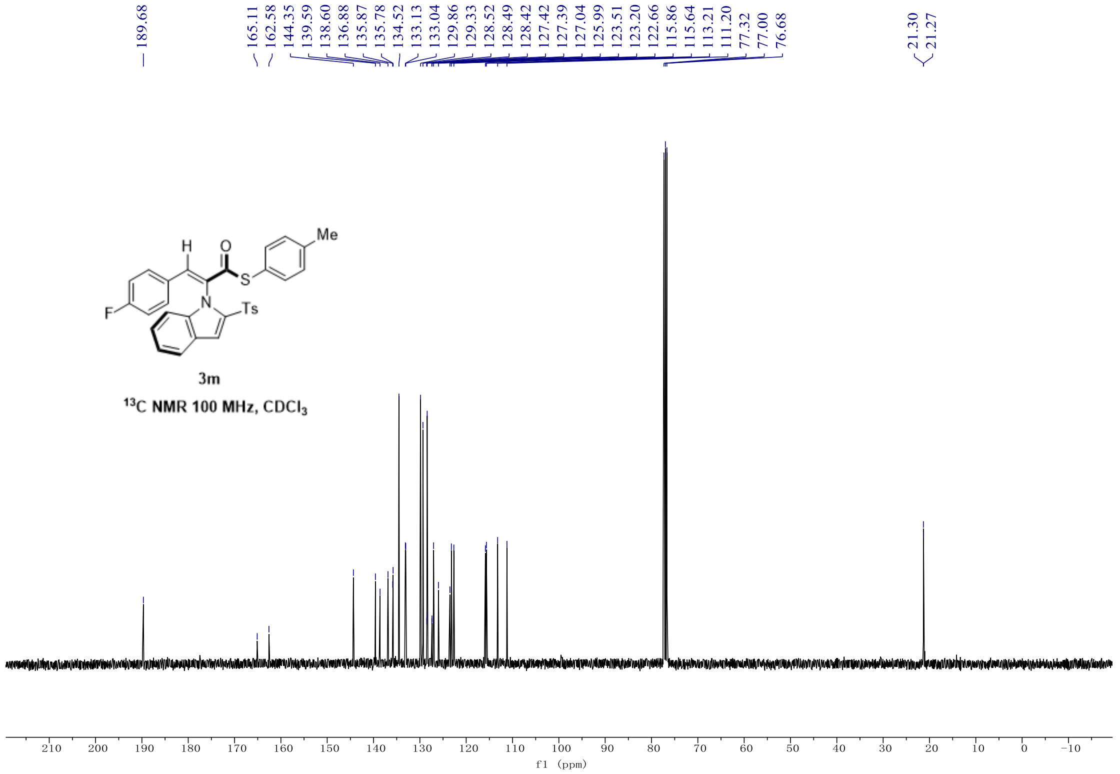
189.68
165.11 162.58 144.35 139.59 138.60 136.88 135.87 135.78 134.52 133.13 133.04 129.86 129.33 128.52 128.49 128.42 127.42 127.39 127.04 125.99 123.51 123.20 122.66 115.86 115.64 113.21 111.20 77.32 77.00 76.68
21.30 21.27
H
O
S
Me
F
N
Ts
3m
13C NMR 100 MHz, CDCl3
210 200 190 180 170 160 150 140 130 120 110 100 90 80 70 60 50 40 30 20 10 0 −10
f1 (ppm)

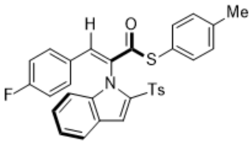

3m

^{19}F NMR 376 MHz，CDCl$_3$

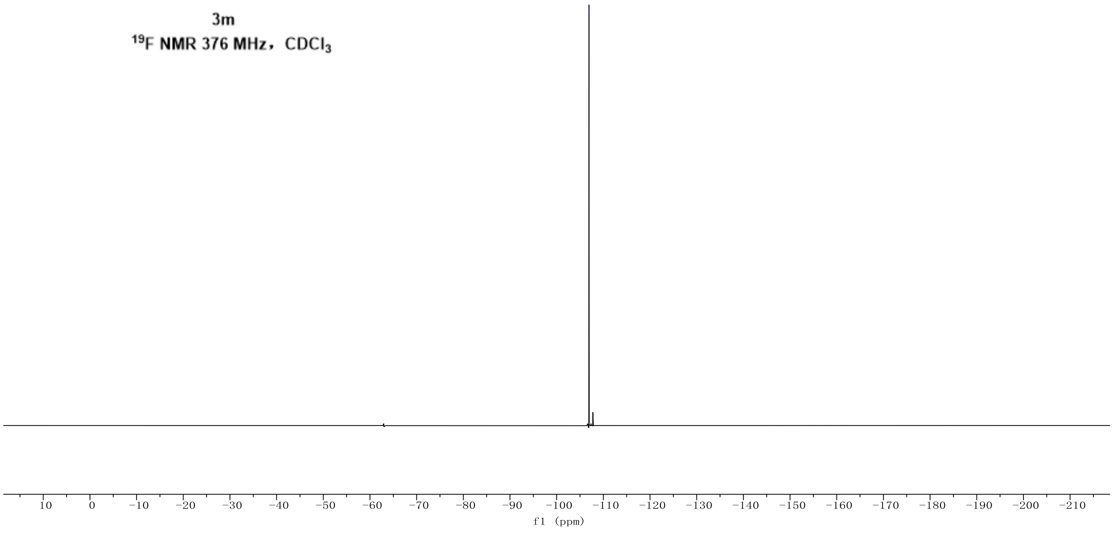

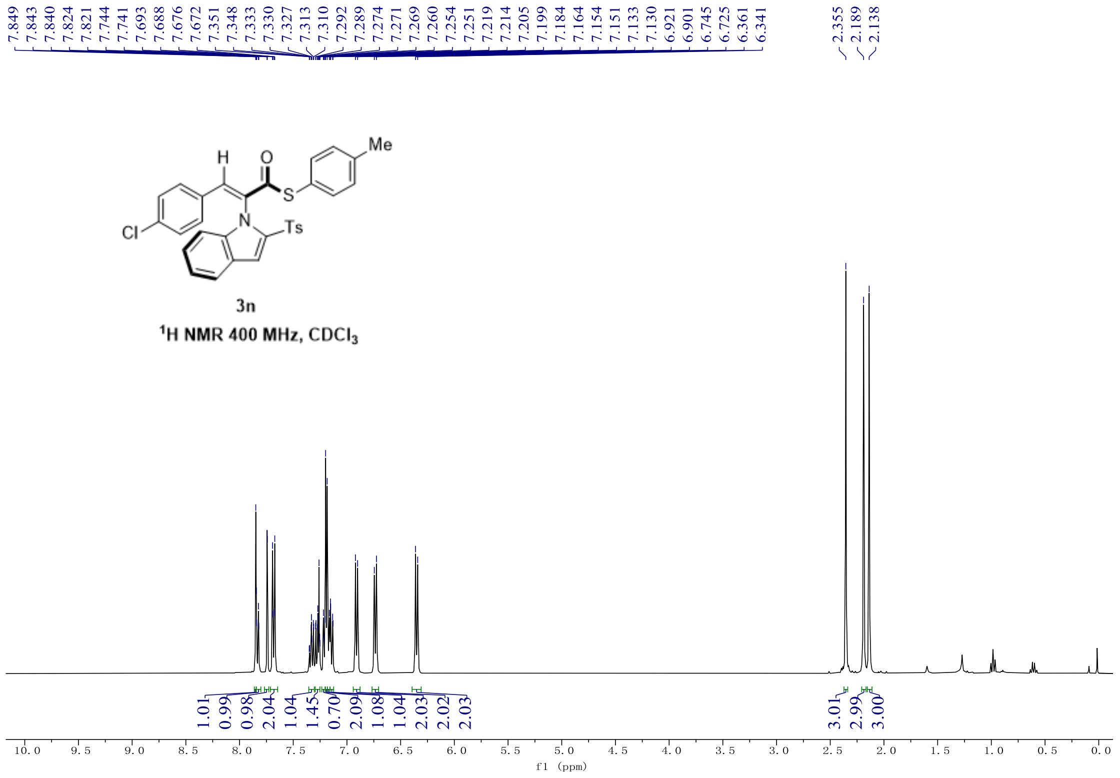
7.849 7.843 7.840 7.824 7.821 7.744 7.741 7.693 7.688 7.676 7.672 7.351 7.348 7.333 7.330 7.327 7.313 7.310 7.292 7.289 7.274 7.271 7.269 7.260 7.254 7.251 7.219 7.214 7.205 7.199 7.184 7.164 7.154 7.151 7.133 7.130 6.921 6.901 6.745 6.725 6.361 6.341
2.355 2.189 2.138
H
O
S
Me
Cl
N
Ts
3n
1H NMR 400 MHz, CDCl3
1.01 0.99 0.98 2.04 1.04 1.45 0.70 2.09 1.08 1.04 2.03 2.02 2.03
3.01 2.99 3.00
10.0 9.5 9.0 8.5 8.0 7.5 7.0 6.5 6.0 5.5 5.0 4.5 4.0 3.5 3.0 2.5 2.0 1.5 1.0 0.5 0.0
f1 (ppm)

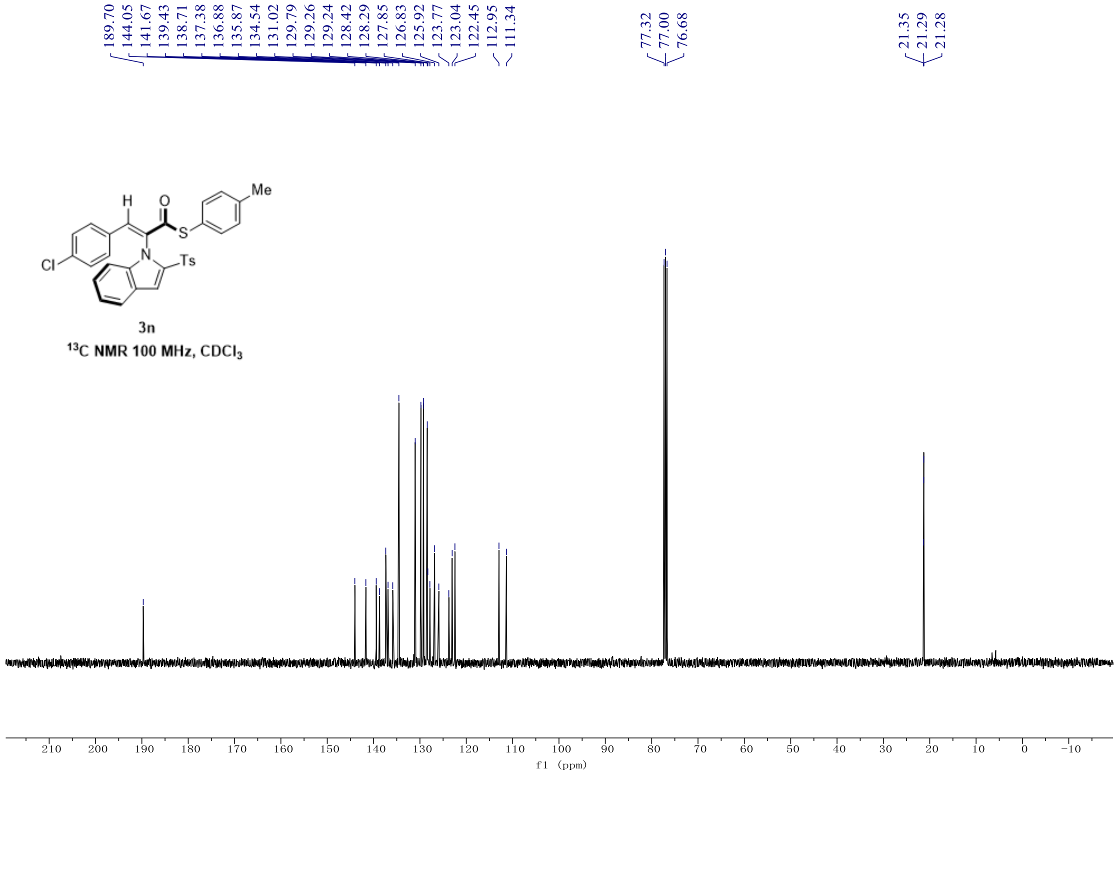
189.70 144.05 141.67 139.43 138.71 137.38 136.88 135.87 134.54 131.02 129.79 129.26 129.24 128.42 128.29 127.85 126.83 125.92 123.77 123.04 122.45 112.95 111.34
77.32 77.00 76.68
21.35 21.29 21.28
H
O
S
Me
Cl
N
Ts
3n
13C NMR 100 MHz, CDCl3
210 200 190 180 170 160 150 140 130 120 110 100 90 80 70 60 50 40 30 20 10 0 -10
f1 (ppm)

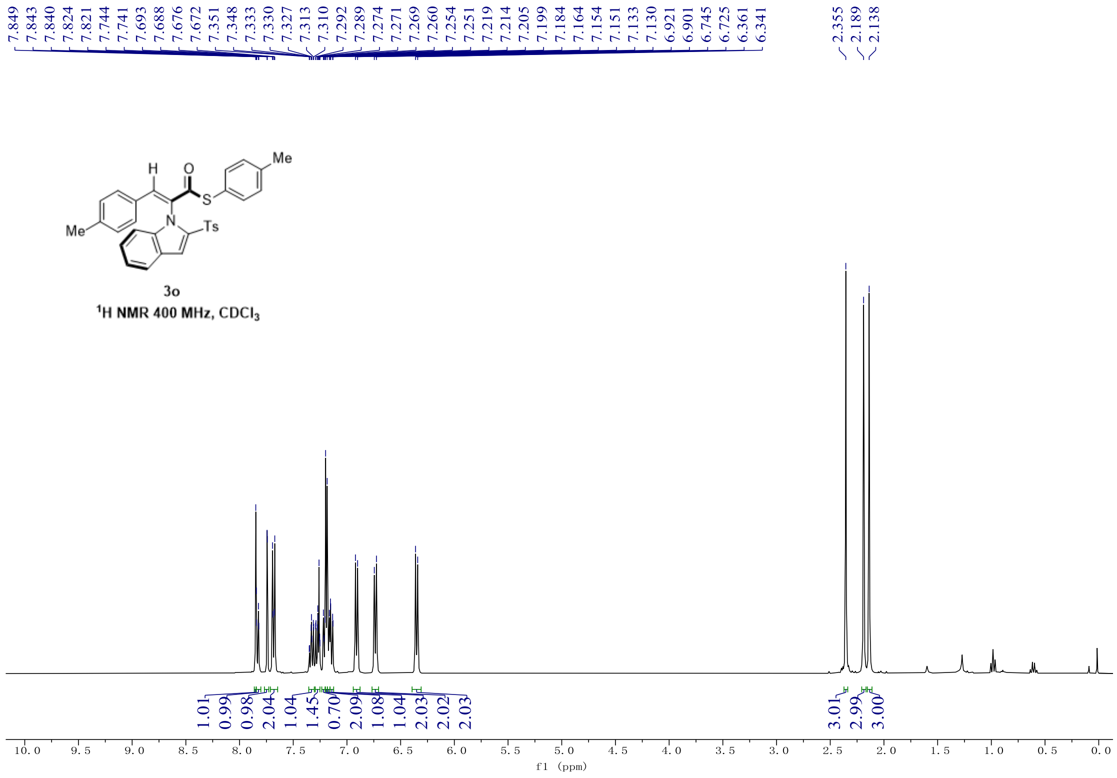
7.849 7.843 7.840 7.824 7.821 7.744 7.741 7.693 7.688 7.676 7.672 7.351 7.348 7.333 7.330 7.327 7.313 7.310 7.292 7.289 7.274 7.271 7.269 7.260 7.254 7.251 7.219 7.214 7.205 7.199 7.184 7.164 7.154 7.151 7.133 7.130 6.921 6.901 6.745 6.725 6.361 6.341
2.355 2.189 2.138
Me
H
O
S
N
Ts
3o
^{1}H NMR 400 MHz, CDCl$_3$
1.01 0.99 0.98 2.04 1.04 1.45 0.70 2.09 1.08 1.04 2.03 2.02 2.03
3.01 2.99 3.00
10.0 9.5 9.0 8.5 8.0 7.5 7.0 6.5 6.0 5.5 5.0 4.5 4.0 3.5 3.0 2.5 2.0 1.5 1.0 0.5 0.0
f1 (ppm)

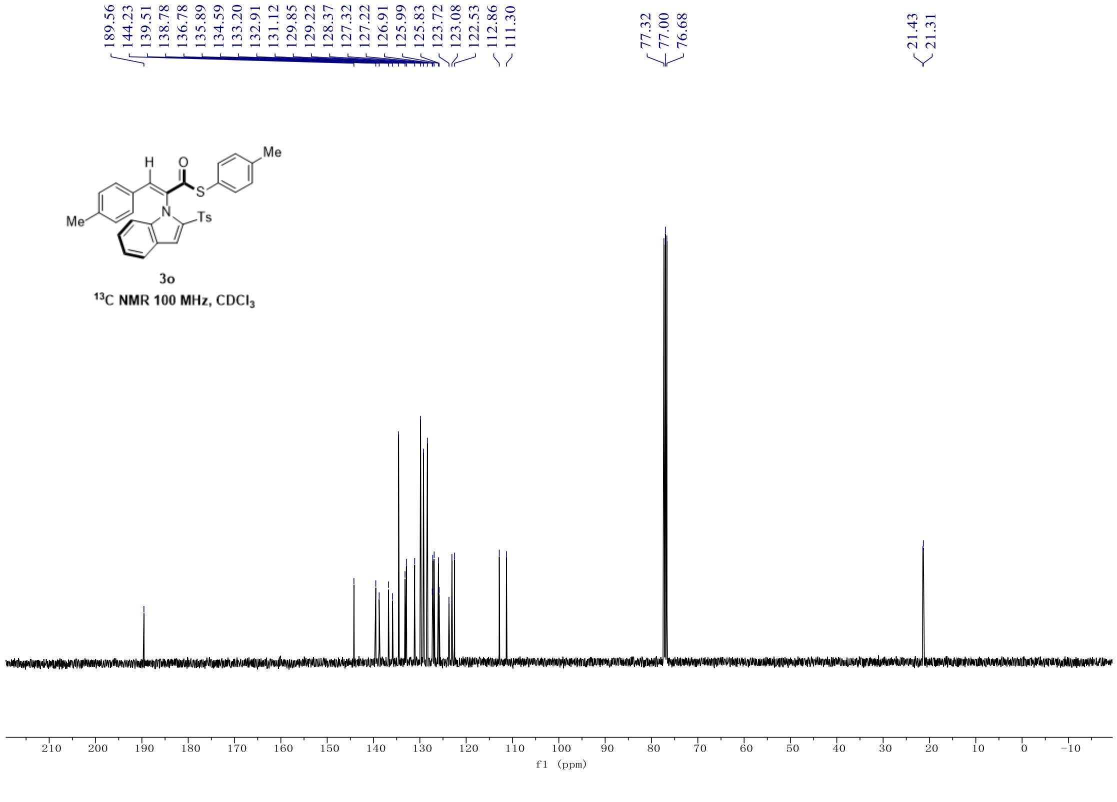
189.56 144.23 139.51 138.78 136.78 135.89 134.59 133.20 132.91 131.12 129.85 129.22 128.37 127.32 127.22 126.91 125.99 125.83 123.72 123.08 122.53 112.86 111.30
77.32 77.00 76.68
21.43 21.31
Me
H
O
S
N
Ts
3o
^{13}C NMR 100 MHz, CDCl$_3$
210 200 190 180 170 160 150 140 130 120 110 100 90 80 70 60 50 40 30 20 10 0 −10
f1 (ppm)

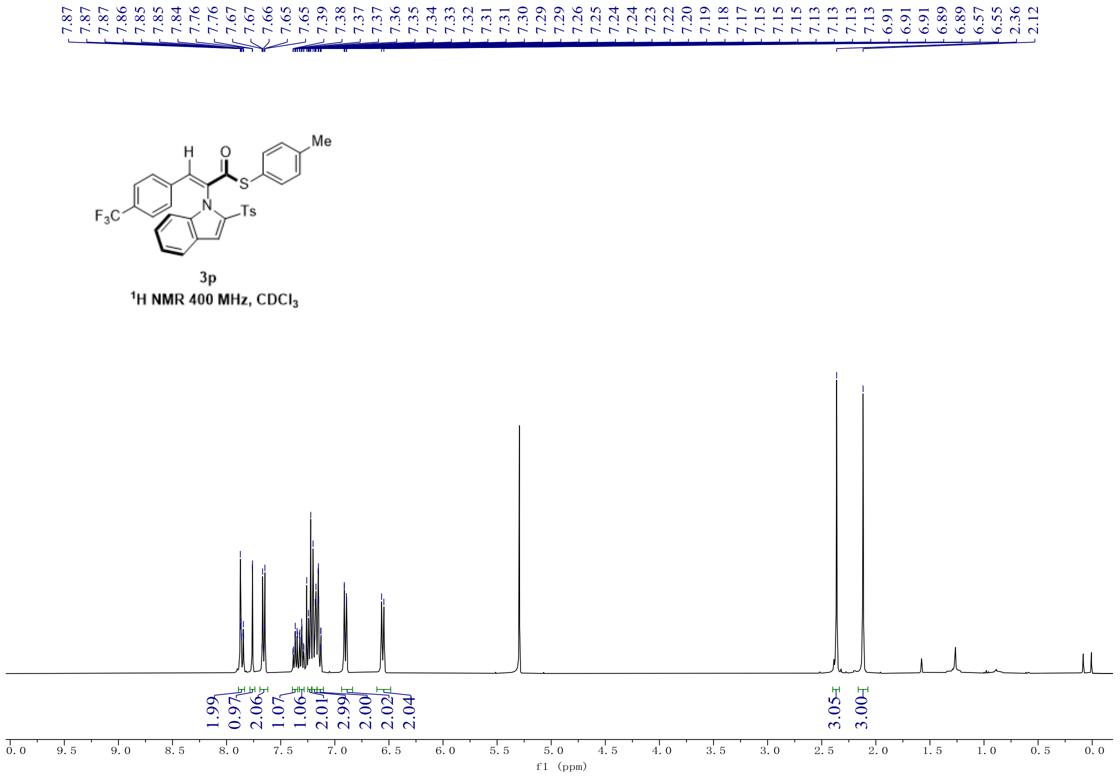
7.87 7.87 7.87 7.86 7.85 7.85 7.84 7.76 7.76 7.67 7.67 7.66 7.65 7.65 7.39 7.38 7.37 7.37 7.36 7.35 7.34 7.33 7.32 7.31 7.31 7.30 7.29 7.29 7.26 7.25 7.24 7.24 7.23 7.22 7.20 7.19 7.18 7.17 7.15 7.15 7.15 7.13 7.13 7.13 7.13 6.91 6.91 6.91 6.89 6.89 6.57 6.55 2.36 2.12
H
O
S
Me
F_3C
N
Ts
3p
^{1}H NMR 400 MHz, $CDCl_3$
1.99 0.97 2.06 1.07 1.06 2.01 2.99 2.00 2.02 2.04 3.05 3.00
f1 (ppm)

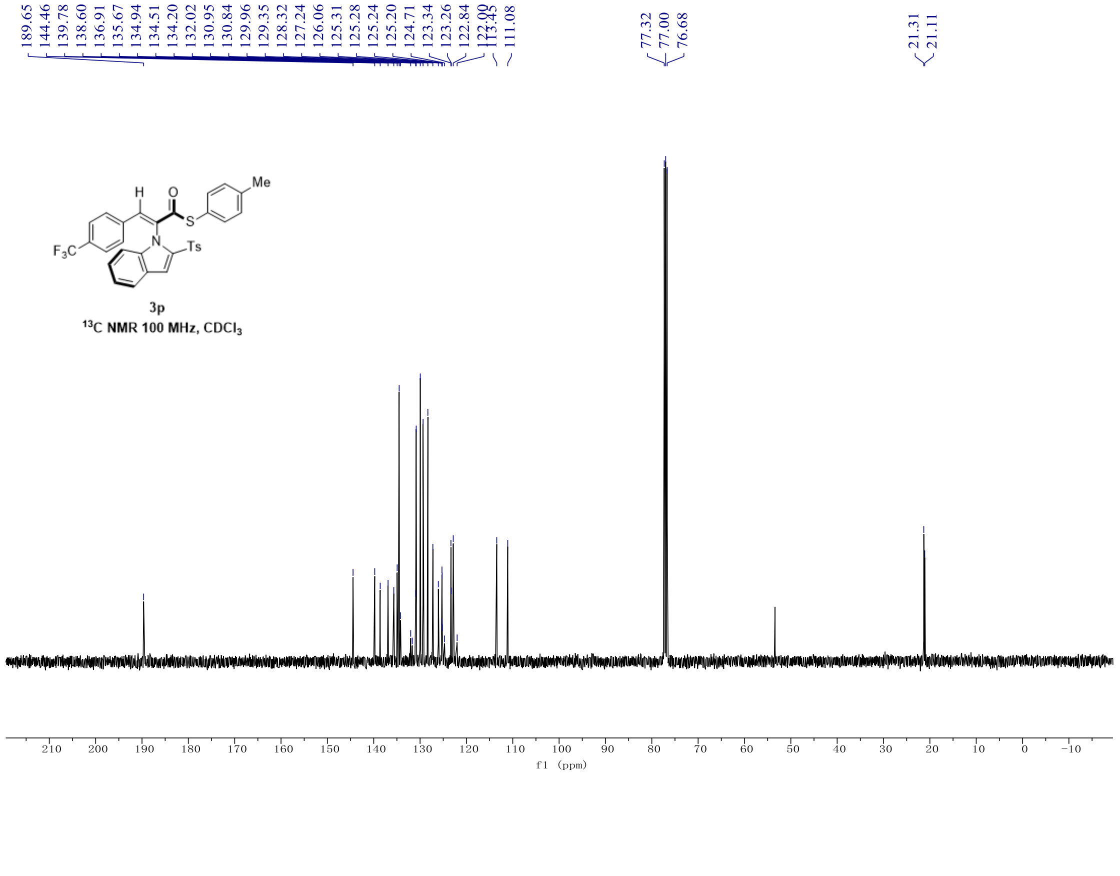
189.65 144.46 139.78 138.60 136.91 135.67 134.94 134.51 134.20 132.02 130.95 130.84 129.96 129.35 128.32 127.24 126.06 125.31 125.28 125.24 125.20 124.71 123.34 123.26 122.84 122.00 113.45 111.08
77.32 77.00 76.68
21.31 21.11
H
O
S
Me
F_3C
N
Ts
3p
^{13}C NMR 100 MHz, $CDCl_3$
f1 (ppm)

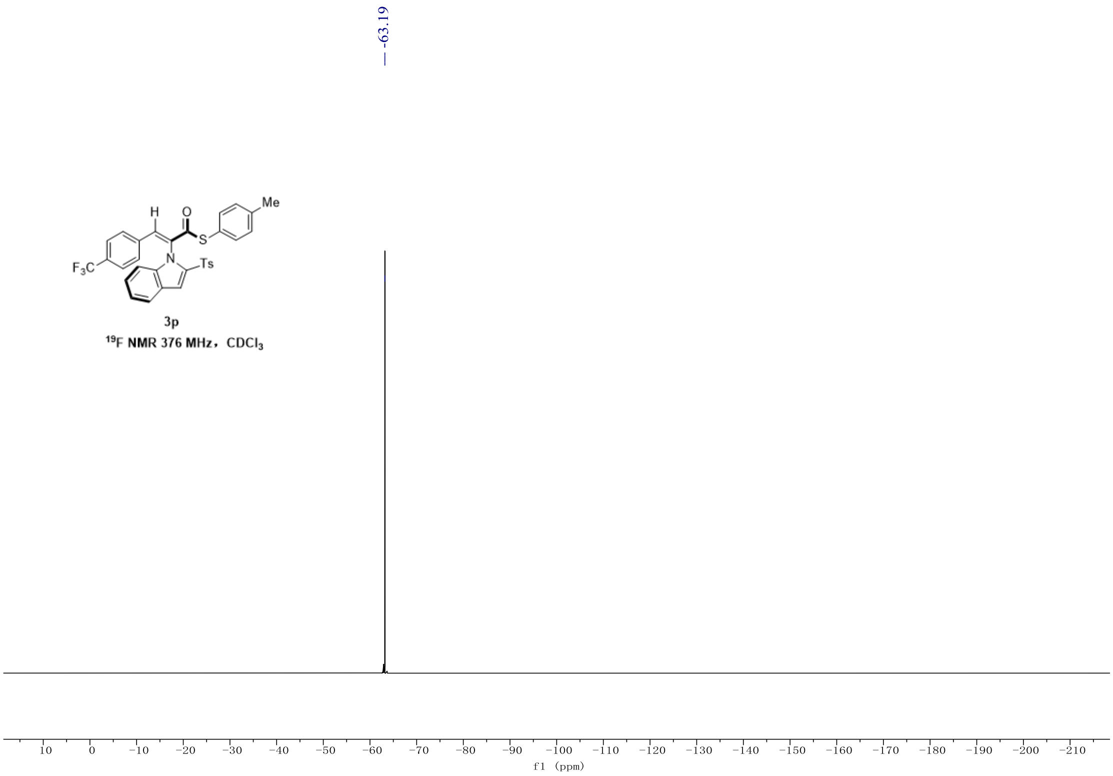
-63.19
Me
H
O
S
N
Ts
F3C
3p
19F NMR 376 MHz，CDCl3
10
0
−10
−20
−30
−40
−50
−60
−70
−80
−90
−100
−110
−120
−130
−140
−150
−160
−170
−180
−190
−200
−210
f1 (ppm)

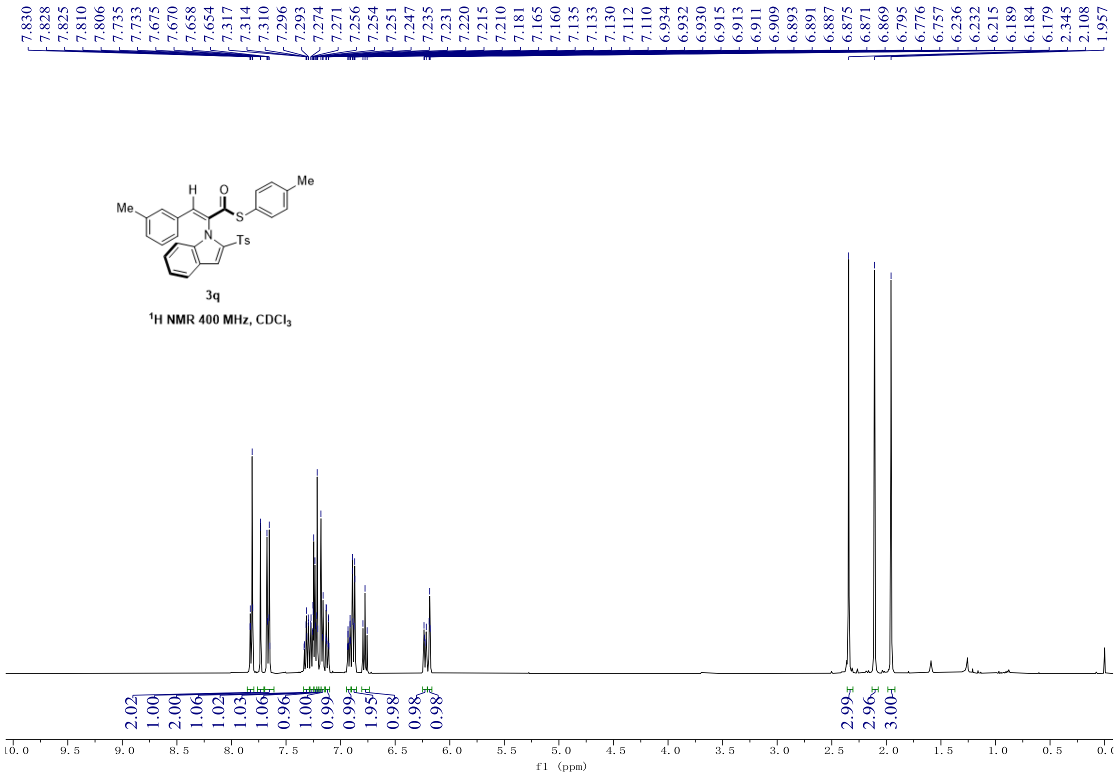
7.830 7.828 7.825 7.810 7.806 7.735 7.733 7.675 7.670 7.658 7.654 7.317 7.314 7.310 7.296 7.293 7.274 7.271 7.256 7.254 7.251 7.247 7.235 7.231 7.220 7.215 7.210 7.181 7.165 7.160 7.135 7.133 7.130 7.112 7.110 6.934 6.932 6.930 6.915 6.913 6.911 6.909 6.893 6.891 6.887 6.875 6.871 6.869 6.795 6.776 6.757 6.236 6.232 6.215 6.189 6.184 6.179 2.345 2.108 1.957
Me
H
O
S
Me
N
Ts
3q
1H NMR 400 MHz, CDCl3
2.02 1.00 2.00 0.96 1.06 1.02 1.03 1.06 1.00 0.99 0.99 1.95 0.98 0.98
2.99 2.96 3.00
f1 (ppm)

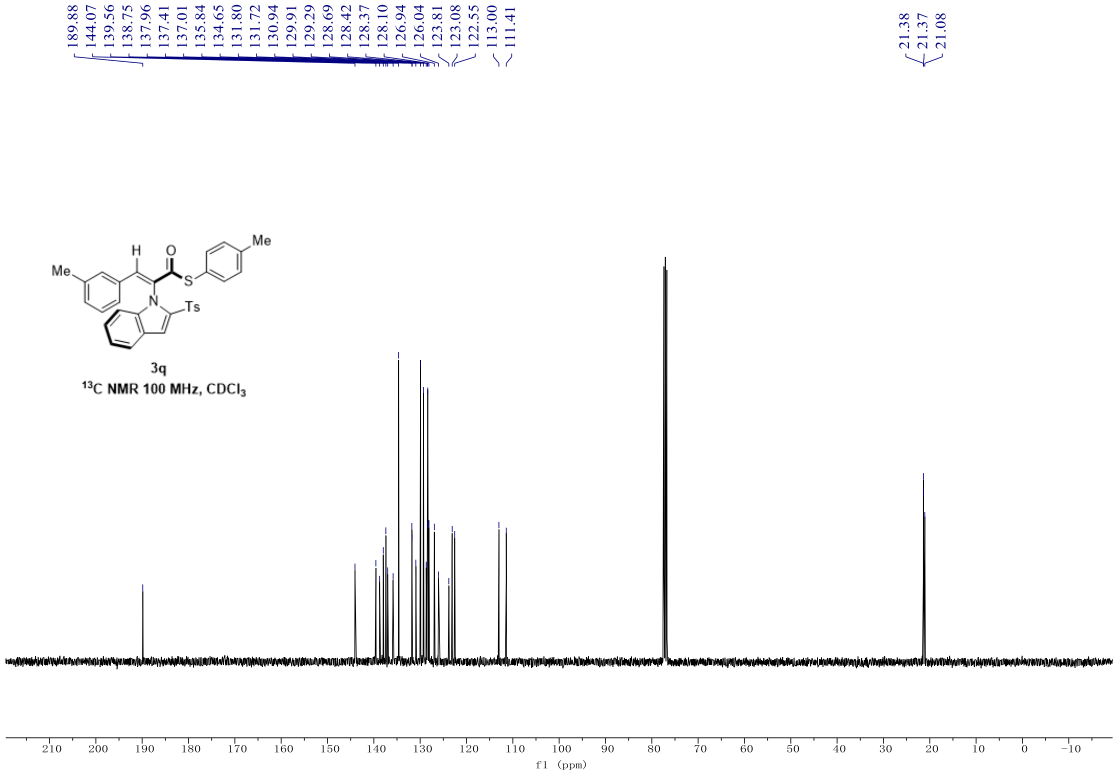
189.88 144.07 139.56 138.75 137.96 137.41 137.01 135.84 134.65 131.80 131.72 130.94 129.91 129.29 128.69 128.42 128.37 128.10 126.94 126.04 123.81 123.08 122.55 113.00 111.41
21.38 21.37 21.08
Me
H
O
S
Me
N
Ts
3q
13C NMR 100 MHz, CDCl3
f1 (ppm)

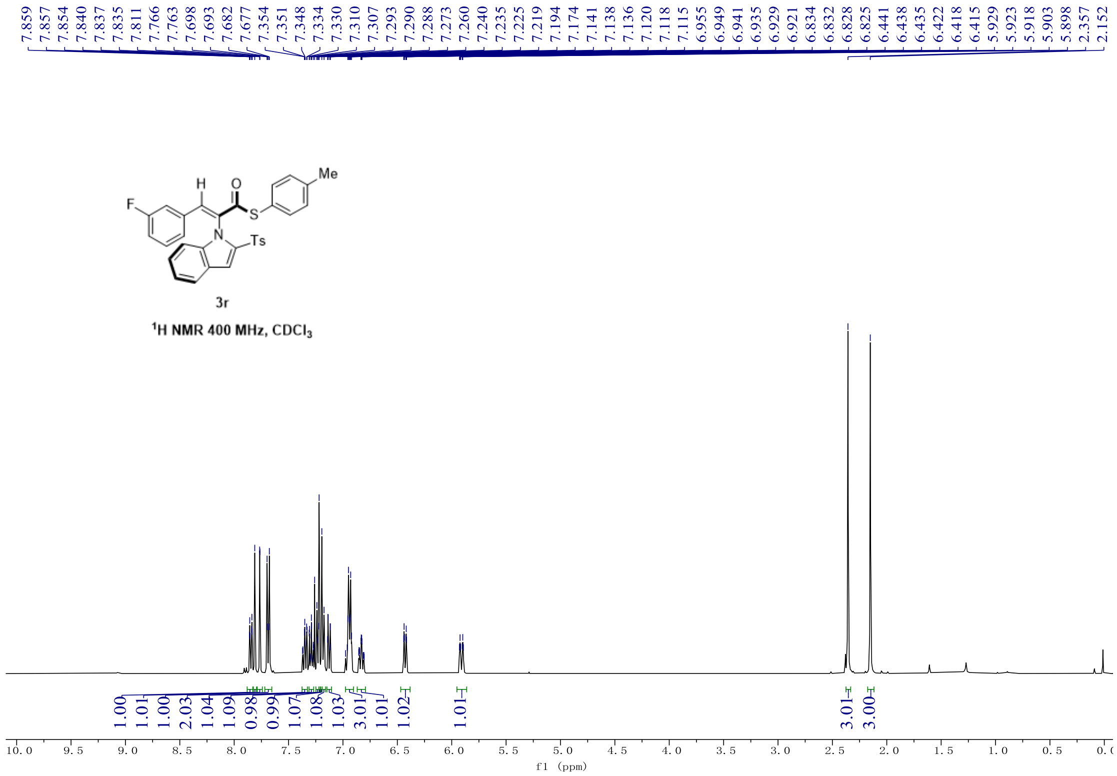
7.859 7.857 7.854 7.840 7.837 7.835 7.811 7.766 7.763 7.698 7.693 7.682 7.677 7.354 7.351 7.348 7.334 7.330 7.310 7.307 7.293 7.290 7.288 7.273 7.260 7.240 7.235 7.225 7.219 7.194 7.174 7.141 7.138 7.136 7.120 7.118 7.115 6.955 6.949 6.941 6.935 6.929 6.921 6.834 6.832 6.828 6.825 6.441 6.438 6.435 6.422 6.418 6.415 5.929 5.923 5.918 5.903 5.898 2.357 2.152
F
H
O
S
Me
N
Ts
3r
¹H NMR 400 MHz, CDCl₃
1.00 1.01 1.00 2.03 1.04 1.09 0.98 0.99 1.07 1.08 1.03 3.01 1.01 1.02 1.01 3.01 3.00
10.0 9.5 9.0 8.5 8.0 7.5 7.0 6.5 6.0 5.5 5.0 4.5 4.0 3.5 3.0 2.5 2.0 1.5 1.0 0.5 0.0
f1 (ppm)

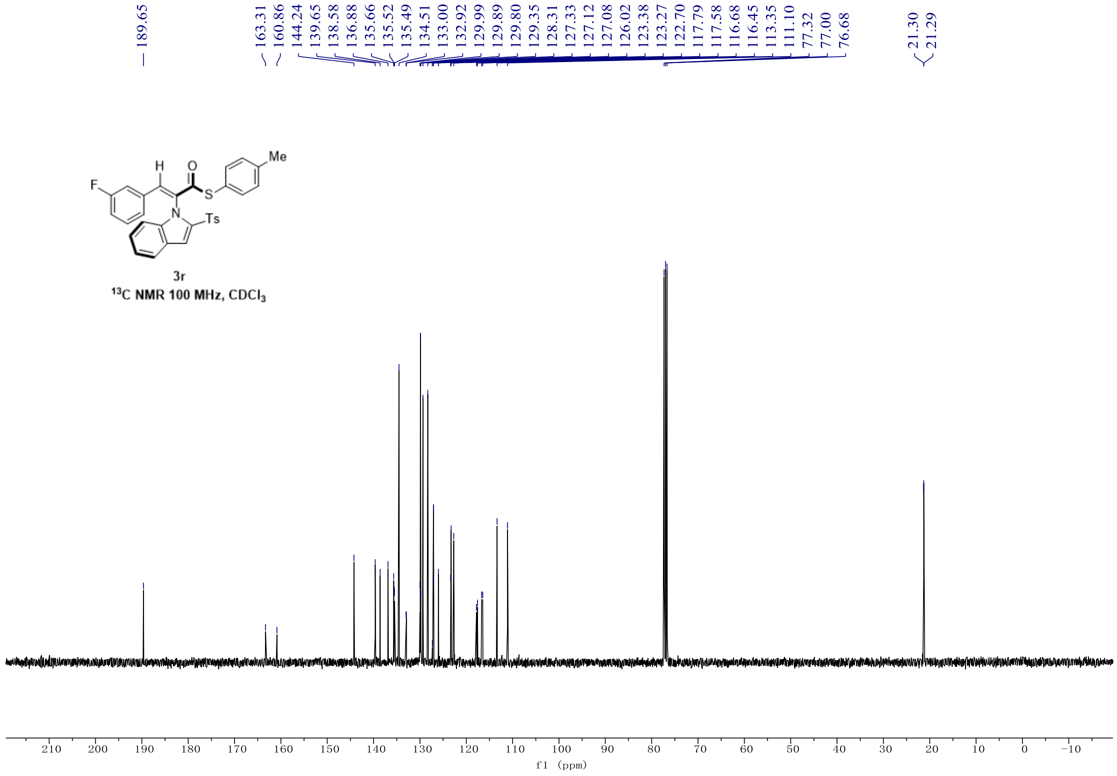
189.65
163.31 160.86 144.24 139.65 138.58 136.88 135.66 135.52 135.49 134.51 133.00 132.92 129.99 129.89 129.80 129.35 128.31 127.33 127.12 127.08 126.02 123.38 123.27 122.70 117.79 117.58 116.68 116.45 113.35 111.10 77.32 77.00 76.68
21.30 21.29
F
H
O
S
Me
N
Ts
3r
¹³C NMR 100 MHz, CDCl₃
210 200 190 180 170 160 150 140 130 120 110 100 90 80 70 60 50 40 30 20 10 0 −10
f1 (ppm)

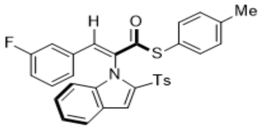

3r

^{19}F NMR 376 MHz, $CDCl_3$

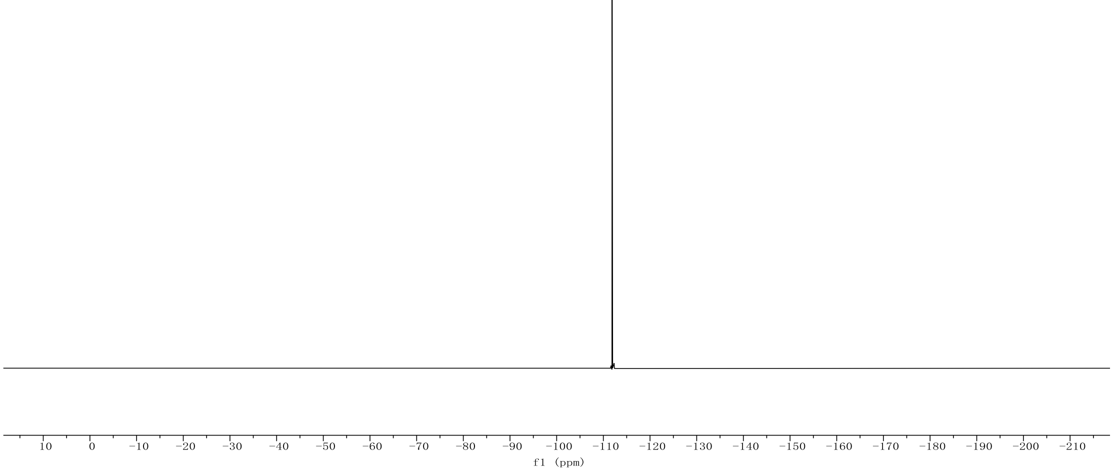

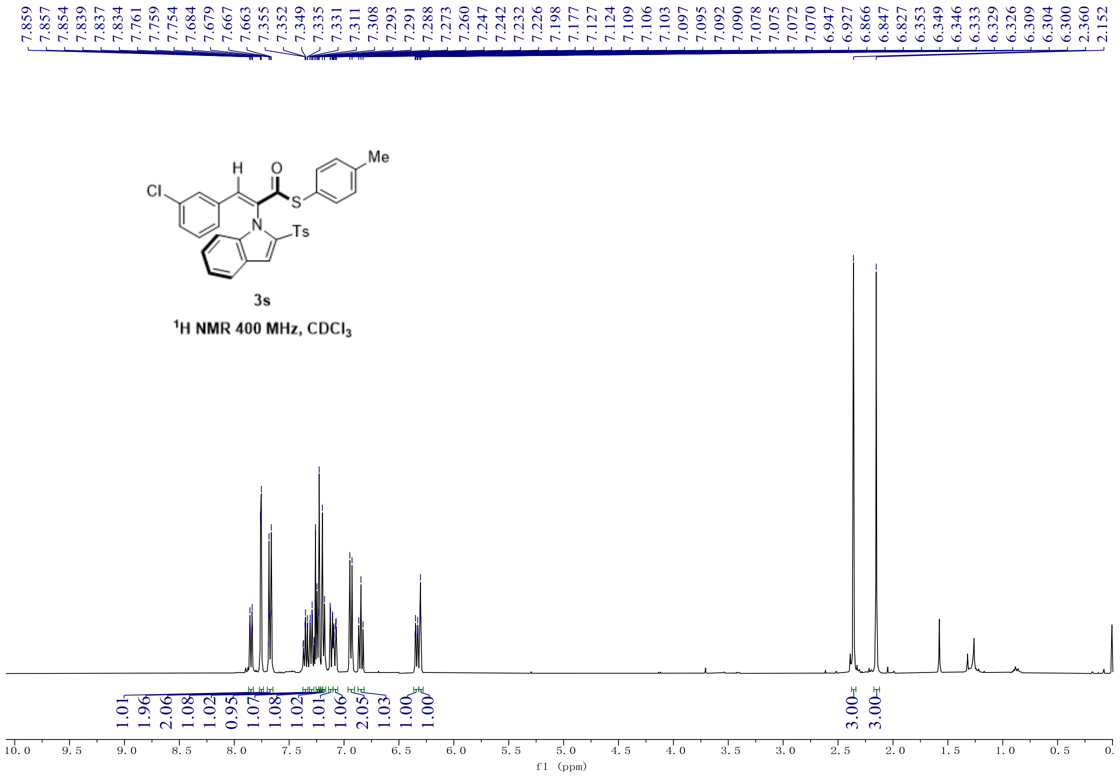
7.859 7.857 7.854 7.839 7.837 7.834 7.761 7.759 7.754 7.684 7.679 7.667 7.663 7.355 7.352 7.349 7.335 7.331 7.311 7.308 7.293 7.291 7.288 7.273 7.260 7.247 7.242 7.232 7.226 7.198 7.177 7.127 7.124 7.109 7.106 7.103 7.097 7.095 7.092 7.090 7.078 7.075 7.072 7.070 6.947 6.927 6.866 6.847 6.827 6.353 6.349 6.346 6.333 6.329 6.326 6.309 6.304 6.300 2.360 2.152
Me
H
O
Cl
S
N
Ts
3s
^{1}H NMR 400 MHz, CDCl$_3$
1.01 1.96 2.06 1.08 1.02 0.95 1.07 1.08 1.02 1.01 1.06 2.05 1.03 1.00 1.00
3.00 3.00
f1 (ppm)

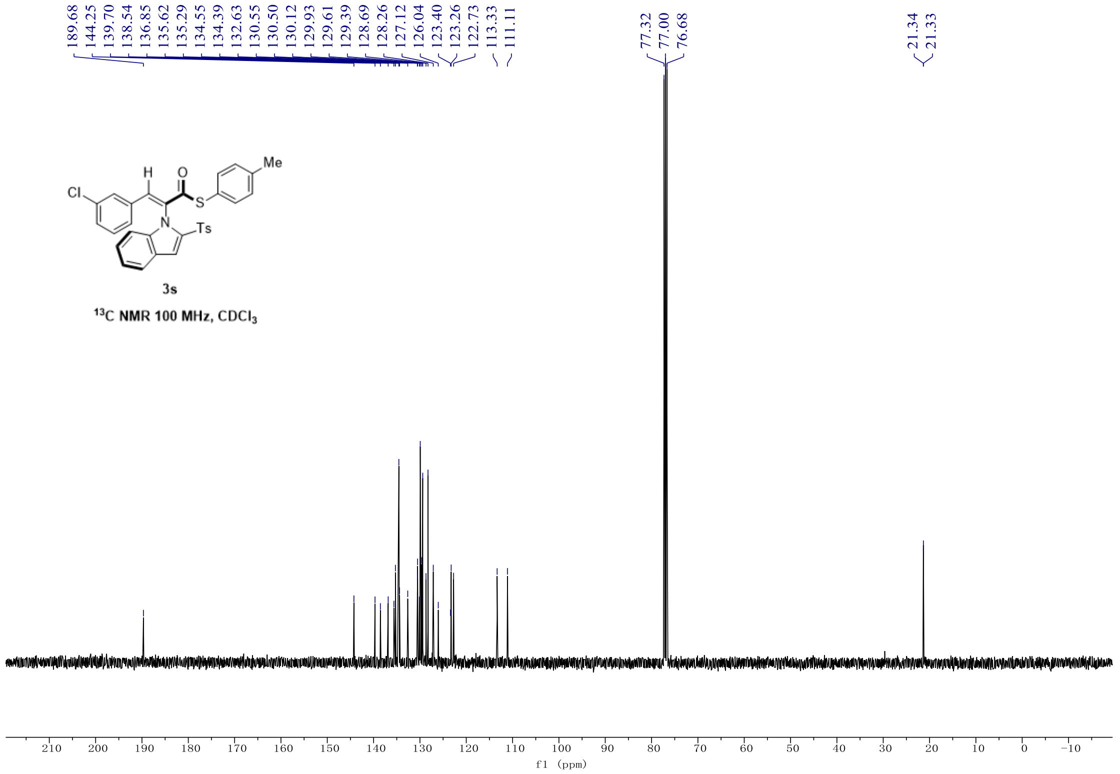
189.68 144.25 139.70 138.54 136.85 135.62 135.29 134.55 134.39 132.63 130.55 130.50 130.12 129.93 129.61 129.39 128.69 128.26 127.12 126.04 123.40 123.26 122.73 113.33 111.11
77.32 77.00 76.68
21.34 21.33
Me
H
O
Cl
S
N
Ts
3s
^{13}C NMR 100 MHz, CDCl$_3$
f1 (ppm)

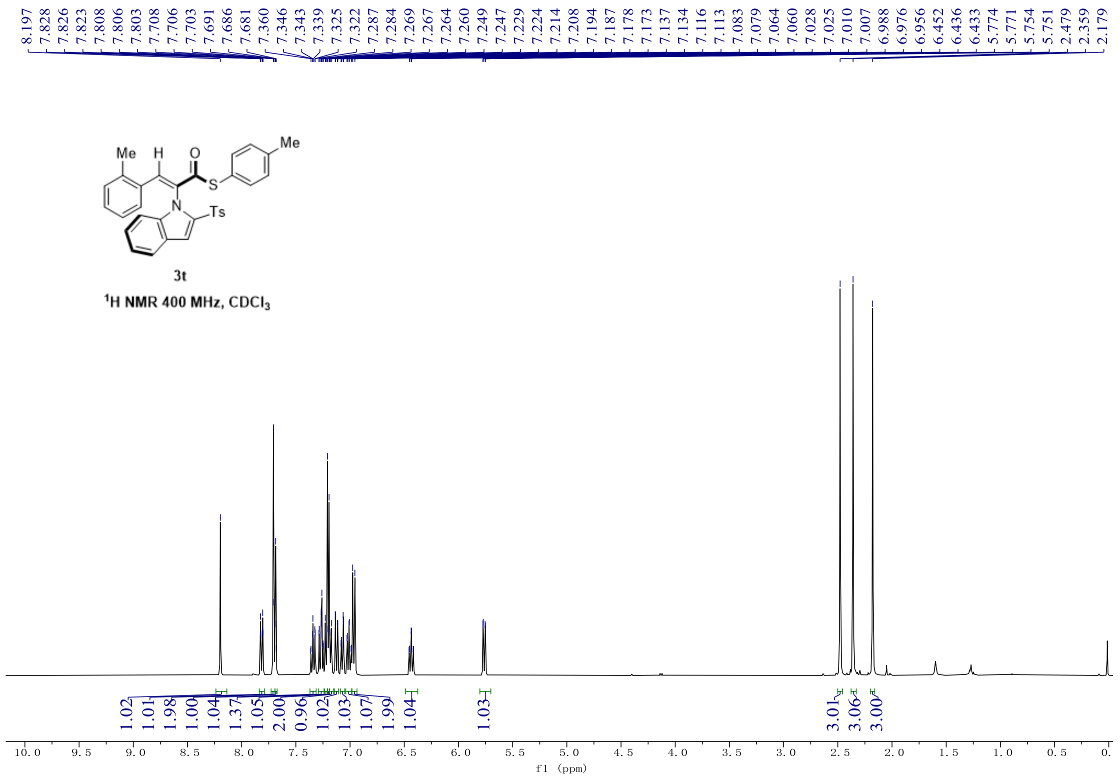
8.197 7.828 7.826 7.823 7.808 7.806 7.803 7.708 7.706 7.703 7.691 7.686 7.681 7.360 7.346 7.343 7.339 7.325 7.322 7.287 7.284 7.269 7.267 7.264 7.260 7.249 7.247 7.229 7.224 7.214 7.208 7.194 7.187 7.178 7.173 7.137 7.134 7.116 7.113 7.083 7.079 7.064 7.060 7.028 7.025 7.010 7.007 6.988 6.976 6.956 6.452 6.436 6.433 5.774 5.771 5.754 5.751 2.479 2.359 2.179
Me
H
O
S
Me
N
Ts
3t
1H NMR 400 MHz, CDCl3
1.02 1.01 1.98 1.00 1.04 1.37 1.05 2.00 0.96 1.02 1.03 1.07 1.99 1.04 1.03
3.01 3.06 3.00
10.0 9.5 9.0 8.5 8.0 7.5 7.0 6.5 6.0 5.5 5.0 4.5 4.0 3.5 3.0 2.5 2.0 1.5 1.0 0.5 0.
f1 (ppm)

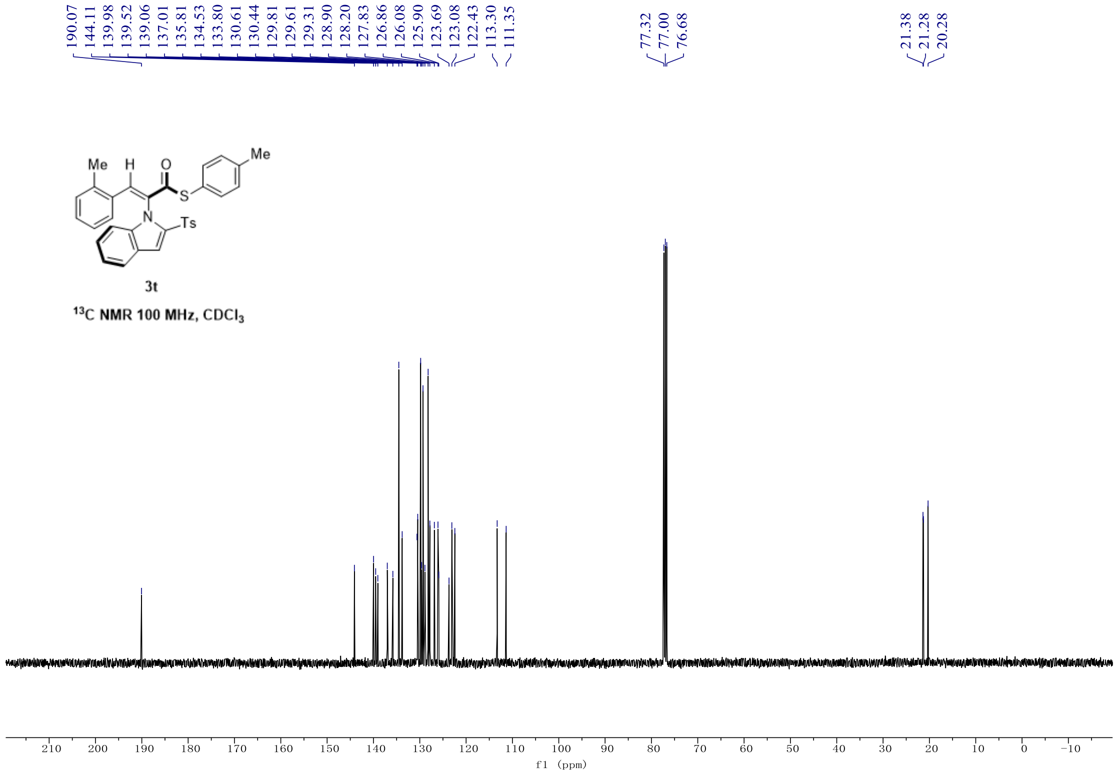
190.07 144.11 139.98 139.52 139.06 137.01 135.81 134.53 133.80 130.61 130.44 129.81 129.61 129.31 128.90 128.20 127.83 126.86 126.08 125.90 123.69 123.08 122.43 113.30 111.35
77.32 77.00 76.68
21.38 21.28 20.28
Me
H
O
S
Me
N
Ts
3t
13C NMR 100 MHz, CDCl3
210 200 190 180 170 160 150 140 130 120 110 100 90 80 70 60 50 40 30 20 10 0 −10
f1 (ppm)

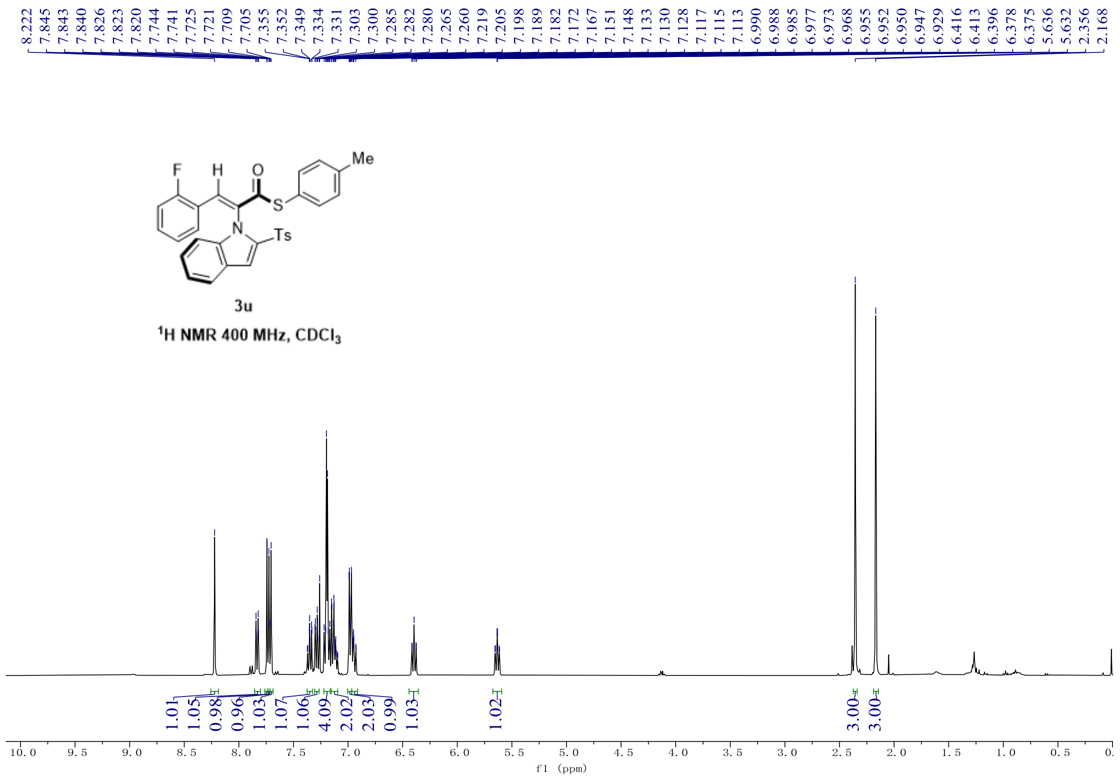
8.222
7.845
7.843
7.840
7.826
7.823
7.820
7.744
7.741
7.725
7.721
7.709
7.705
7.355
7.352
7.349
7.334
7.331
7.303
7.300
7.285
7.282
7.280
7.265
7.260
7.219
7.205
7.198
7.189
7.182
7.172
7.167
7.151
7.148
7.133
7.130
7.128
7.117
7.115
7.113
6.990
6.988
6.985
6.977
6.973
6.968
6.955
6.952
6.950
6.947
6.929
6.416
6.413
6.396
6.378
6.375
5.636
5.632
2.356
2.168
F
H
O
S
Me
N
Ts
3u
1H NMR 400 MHz, CDCl3
1.01
1.05
0.98
0.96
1.03
1.07
1.06
4.09
2.02
2.03
0.99
1.03
1.02
3.00
3.00
f1 (ppm)

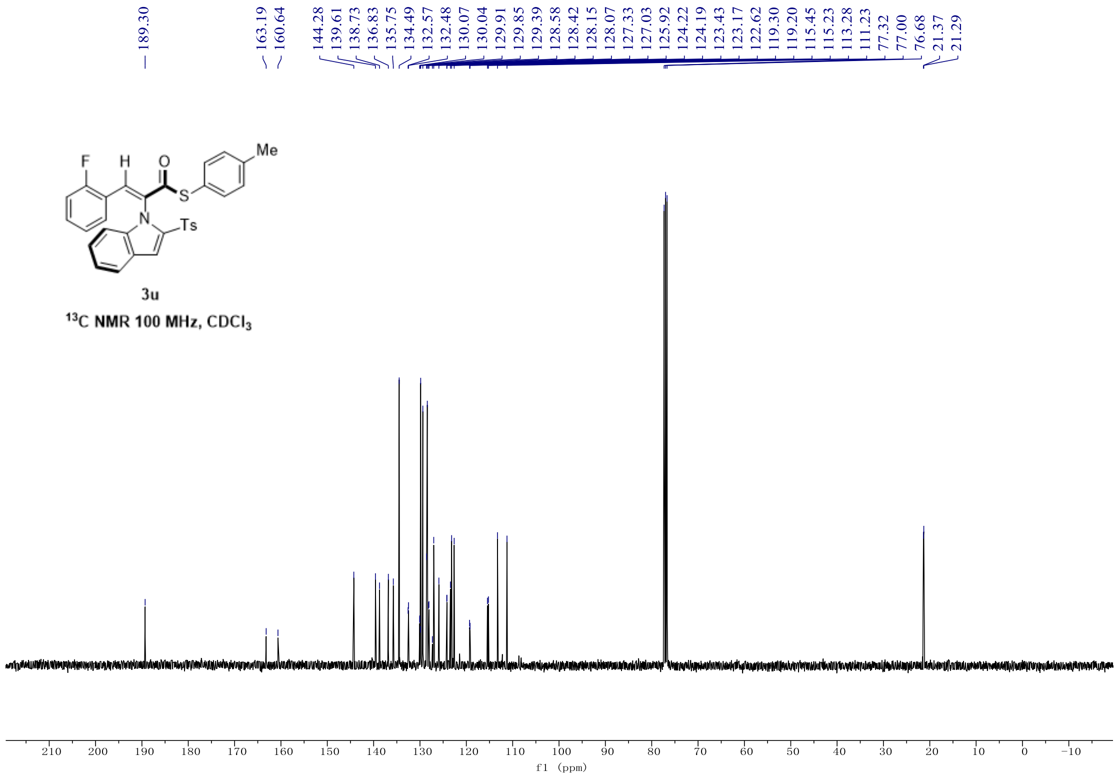
189.30
163.19
160.64
144.28
139.61
138.73
136.83
135.75
134.49
132.57
132.48
130.07
130.04
129.91
129.85
129.39
128.58
128.42
128.15
128.07
127.33
127.03
125.92
124.22
124.19
123.43
123.17
122.62
119.30
119.20
115.45
115.23
113.28
111.23
77.32
77.00
76.68
21.37
21.29
F
H
O
S
Me
N
Ts
3u
13C NMR 100 MHz, CDCl3
f1 (ppm)

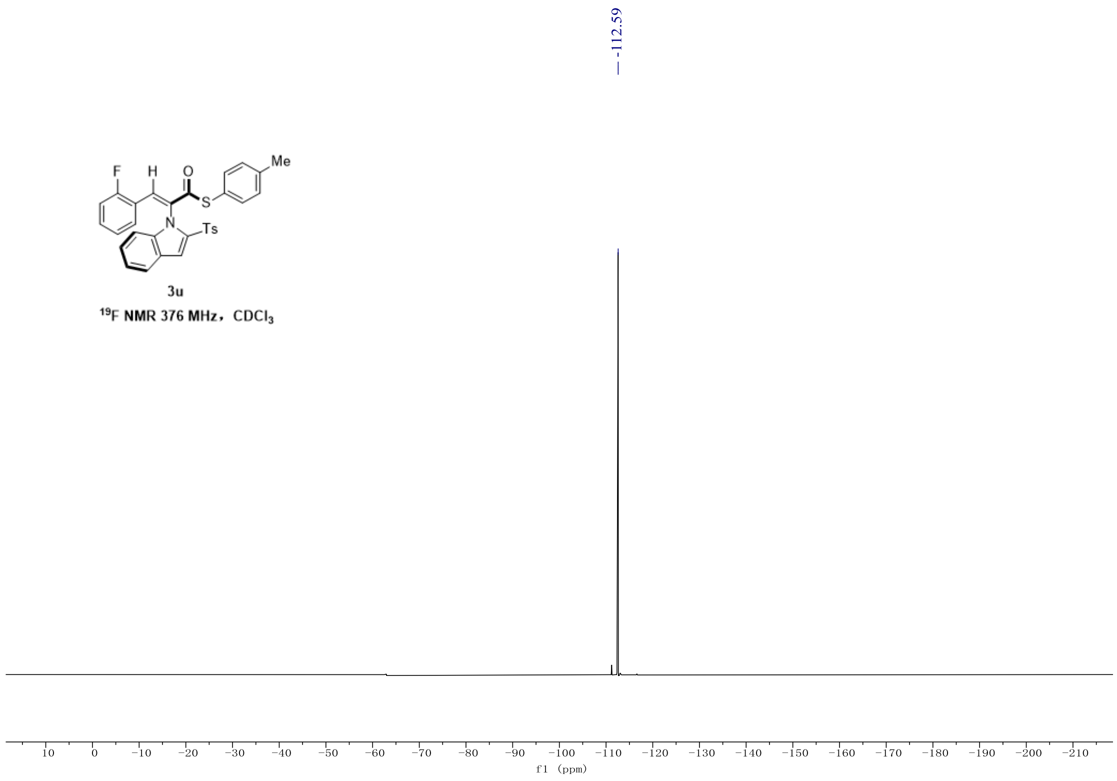

-112.59
F
H
O
S
Me
N
Ts
3u
^{19}F NMR 376 MHz，CDCl$_3$
10
0
−10
−20
−30
−40
−50
−60
−70
−80
−90
−100
−110
−120
−130
−140
−150
−160
−170
−180
−190
−200
−210
f1 (ppm)

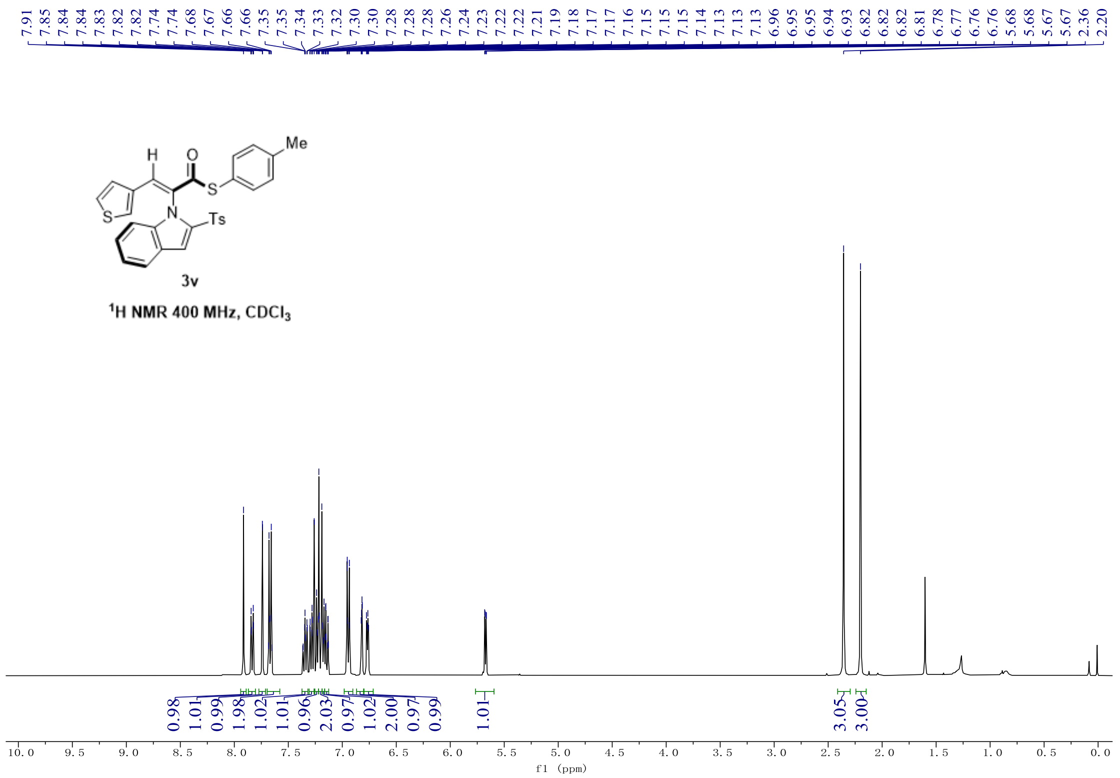
7.91 7.85 7.84 7.83 7.82 7.82 7.74 7.74 7.68 7.67 7.66 7.35 7.35 7.34 7.33 7.32 7.30 7.30 7.28 7.28 7.28 7.26 7.24 7.23 7.22 7.22 7.21 7.19 7.18 7.17 7.17 7.16 7.15 7.15 7.15 7.14 7.13 7.13 7.13 6.96 6.95 6.95 6.94 6.93 6.82 6.82 6.81 6.78 6.77 6.76 5.68 5.68 5.67 5.67 2.36 2.20
H
O
S
Me
S
N
Ts
3v
1H NMR 400 MHz, CDCl3
0.98 1.01 0.99 1.98 1.02 1.01 0.96 2.03 0.97 1.02 2.00 0.97 0.99 1.01 3.05 3.00
10.0 9.5 9.0 8.5 8.0 7.5 7.0 6.5 6.0 5.5 5.0 4.5 4.0 3.5 3.0 2.5 2.0 1.5 1.0 0.5 0.0
f1 (ppm)

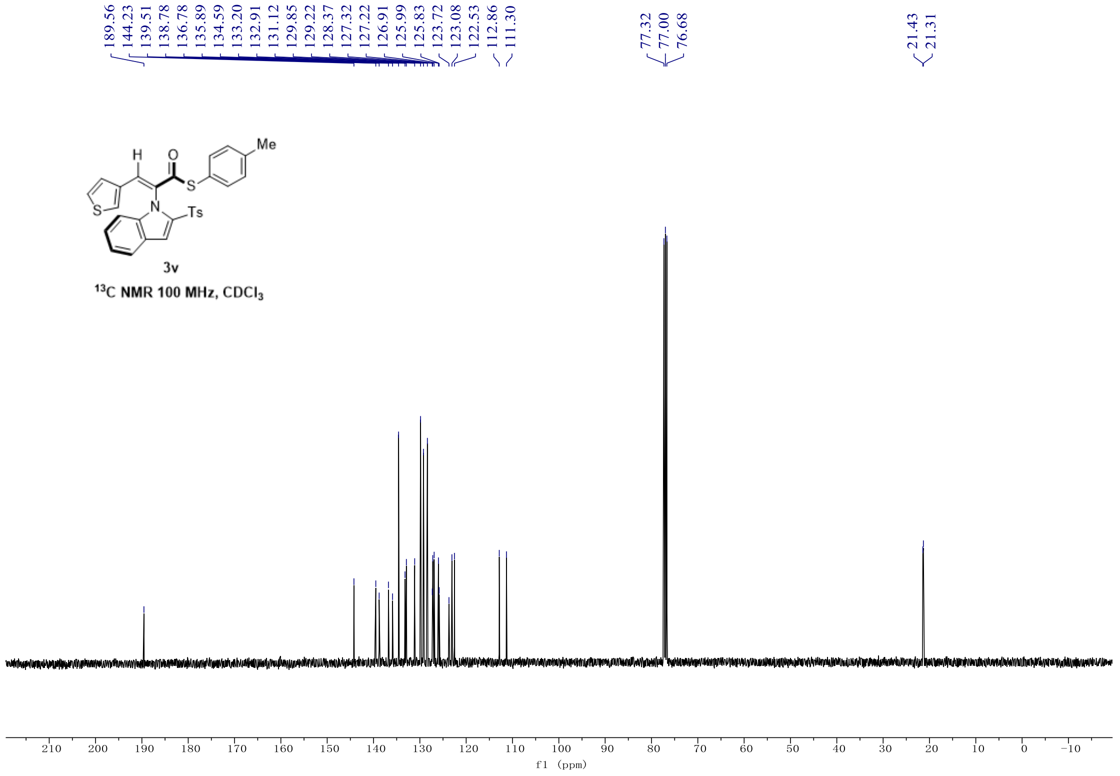
189.56 144.23 139.51 138.78 136.78 135.89 134.59 133.20 132.91 131.12 129.85 129.22 128.37 127.32 127.22 126.91 125.99 125.83 123.72 123.08 122.53 112.86 111.30
77.32 77.00 76.68
21.43 21.31
H
O
S
Me
S
N
Ts
3v
13C NMR 100 MHz, CDCl3
210 200 190 180 170 160 150 140 130 120 110 100 90 80 70 60 50 40 30 20 10 0 -10
f1 (ppm)

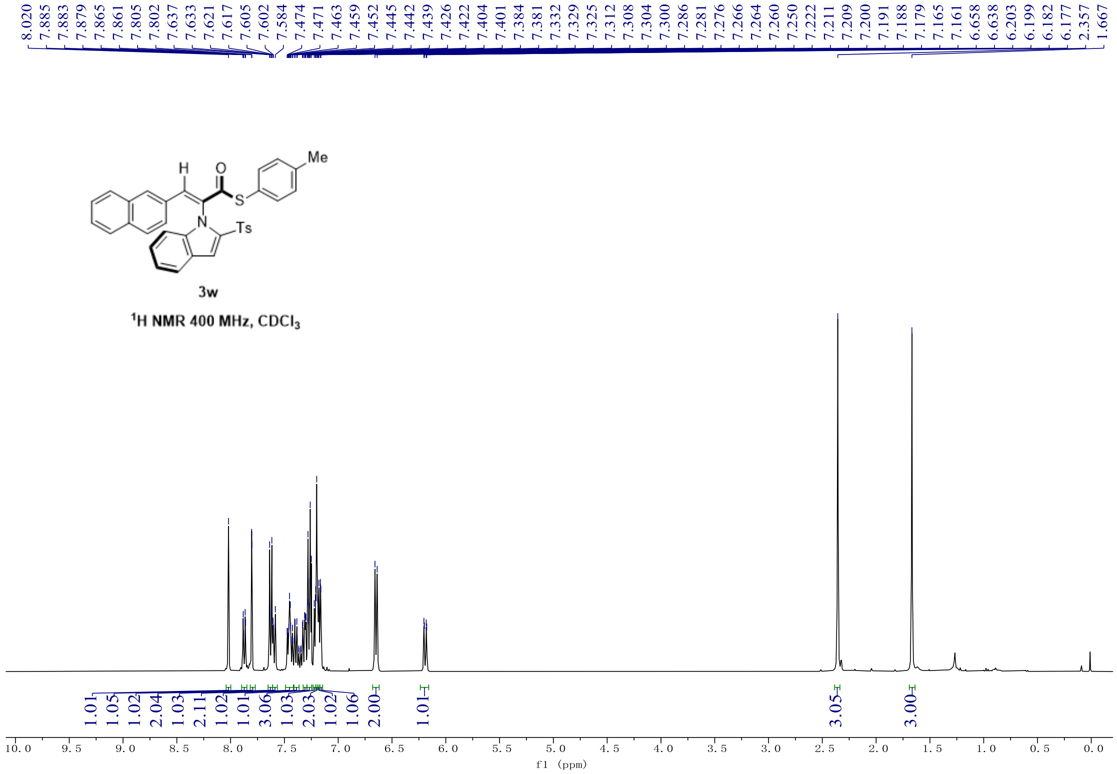
8.020
7.885
7.883
7.879
7.865
7.861
7.805
7.802
7.637
7.633
7.621
7.617
7.605
7.602
7.584
7.474
7.471
7.463
7.459
7.452
7.445
7.442
7.439
7.426
7.422
7.404
7.401
7.384
7.381
7.332
7.329
7.325
7.312
7.308
7.304
7.300
7.286
7.281
7.276
7.266
7.264
7.260
7.250
7.222
7.211
7.209
7.200
7.191
7.188
7.179
7.165
7.161
6.658
6.638
6.203
6.199
6.182
6.177
2.357
1.667
H
O
S
Me
N
Ts
3w
^{1}H NMR 400 MHz, CDCl$_3$
1.01
1.05
1.02
2.04
1.03
2.11
1.02
1.01
3.06
1.03
2.03
1.02
1.06
2.00
1.01
3.05
3.00
f1 (ppm)

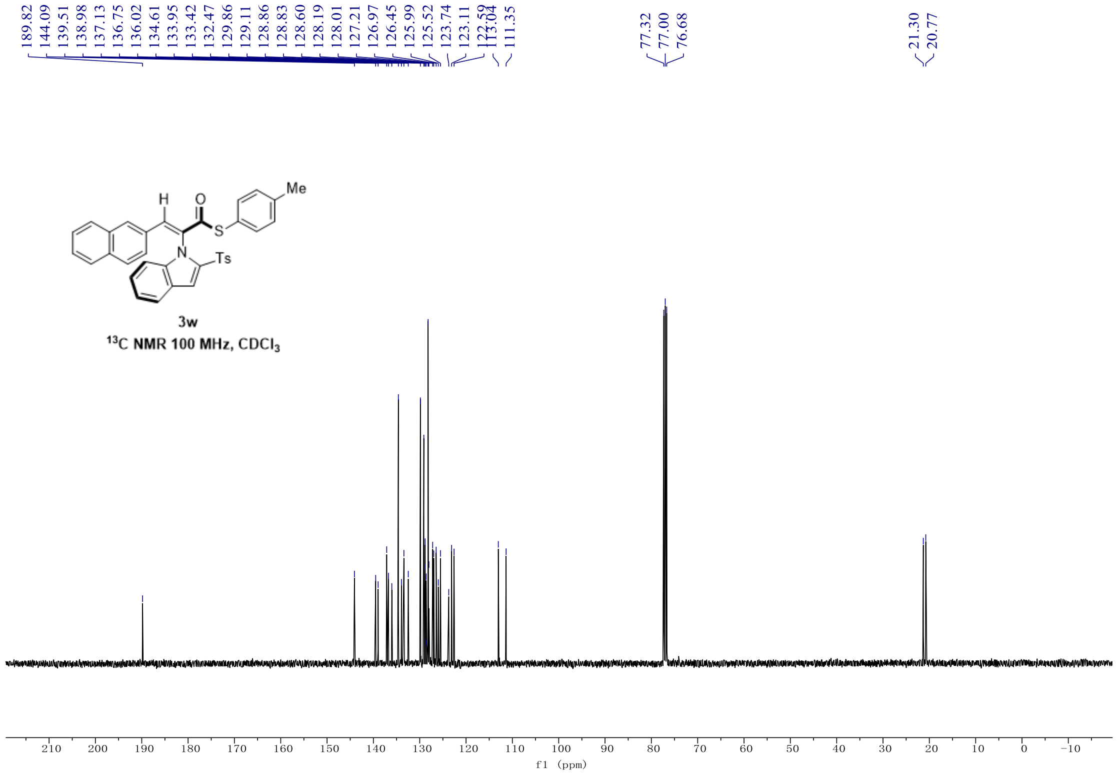
189.82
144.09
139.51
138.98
137.13
136.75
136.02
134.61
133.95
133.42
132.47
129.86
129.11
128.86
128.83
128.60
128.19
128.01
127.21
126.97
126.45
125.99
125.52
123.74
123.11
122.59
113.04
111.35
77.32
77.00
76.68
21.30
20.77
H
O
S
Me
N
Ts
3w
^{13}C NMR 100 MHz, CDCl$_3$
f1 (ppm)

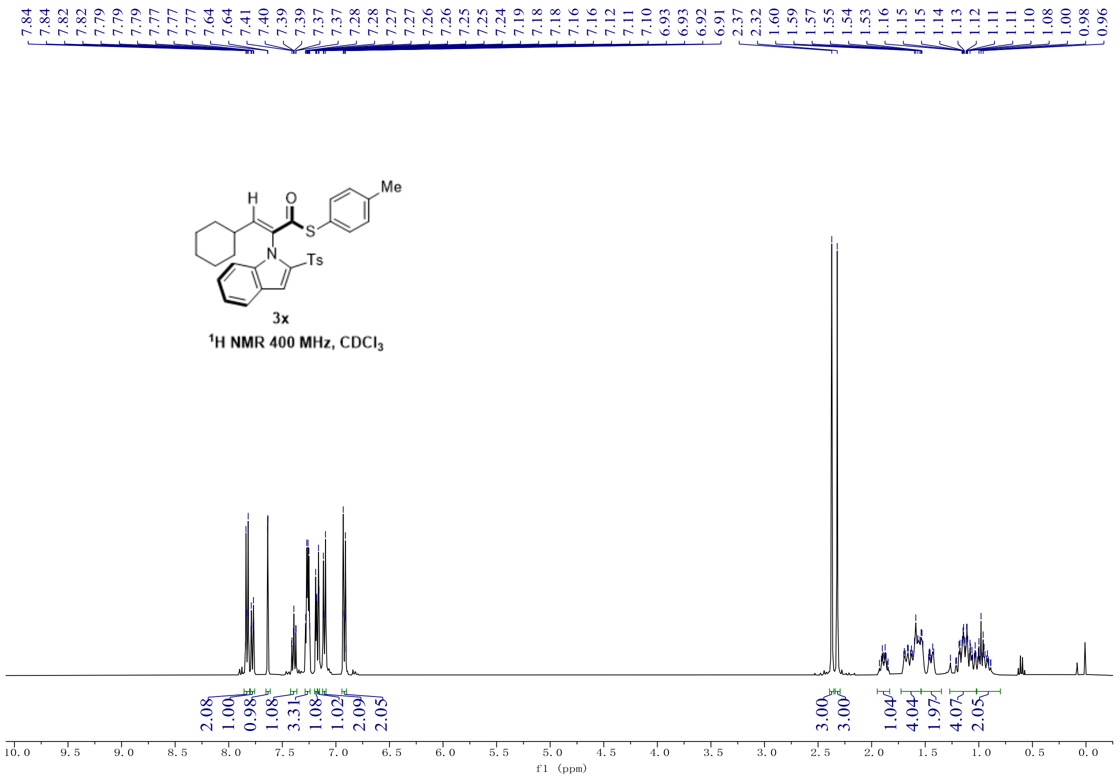
7.84 7.84 7.82 7.82 7.79 7.79 7.77 7.77 7.77 7.64 7.64 7.41 7.40 7.39 7.39 7.37 7.37 7.28 7.28 7.27 7.26 7.26 7.25 7.25 7.24 7.19 7.18 7.16 7.16 7.12 7.11 7.10 6.93 6.93 6.92 6.91 2.37 2.32 1.60 1.59 1.57 1.55 1.54 1.53 1.16 1.15 1.15 1.14 1.13 1.12 1.11 1.11 1.10 1.08 1.00 0.98 0.96
H
O
S
Me
N
Ts
3x
1H NMR 400 MHz, CDCl3
2.08 1.00 0.98 1.08 3.31 1.08 1.02 2.09 2.05
3.00 3.00 1.04 4.04 1.97 4.07 2.05
10.0 9.5 9.0 8.5 8.0 7.5 7.0 6.5 6.0 5.5 5.0 4.5 4.0 3.5 3.0 2.5 2.0 1.5 1.0 0.5 0.0
f1 (ppm)

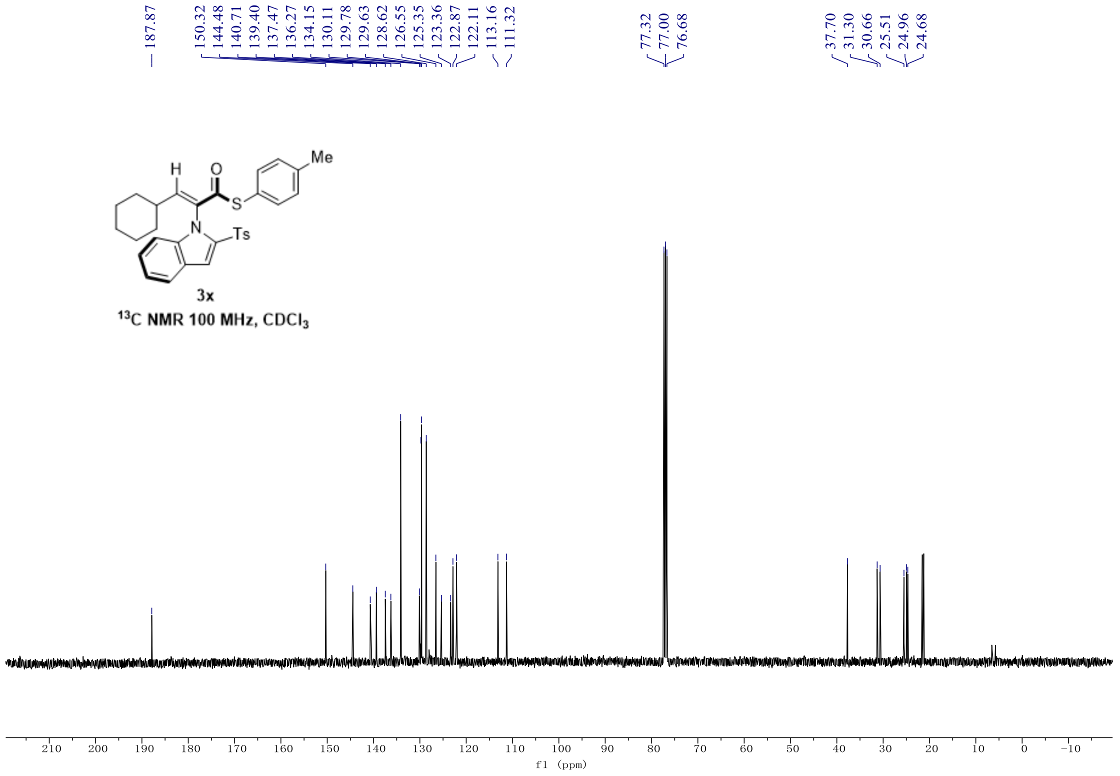
187.87
150.32 144.48 140.71 139.40 137.47 136.27 134.15 130.11 129.78 129.63 128.62 126.55 125.35 123.36 122.87 122.11 113.16 111.32
77.32 77.00 76.68
37.70 31.30 30.66 25.51 24.96 24.68
H
O
S
Me
N
Ts
3x
13C NMR 100 MHz, CDCl3
210 200 190 180 170 160 150 140 130 120 110 100 90 80 70 60 50 40 30 20 10 0 -10
f1 (ppm)

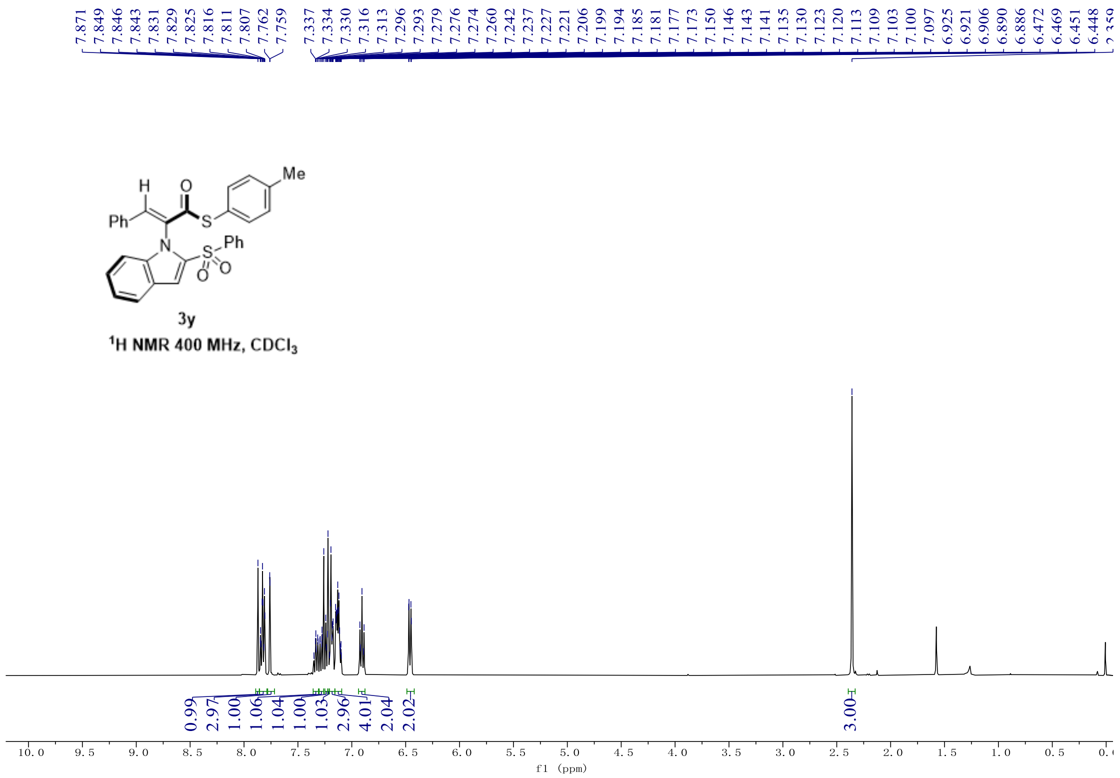
3y
^{1}H NMR 400 MHz, $CDCl_3$

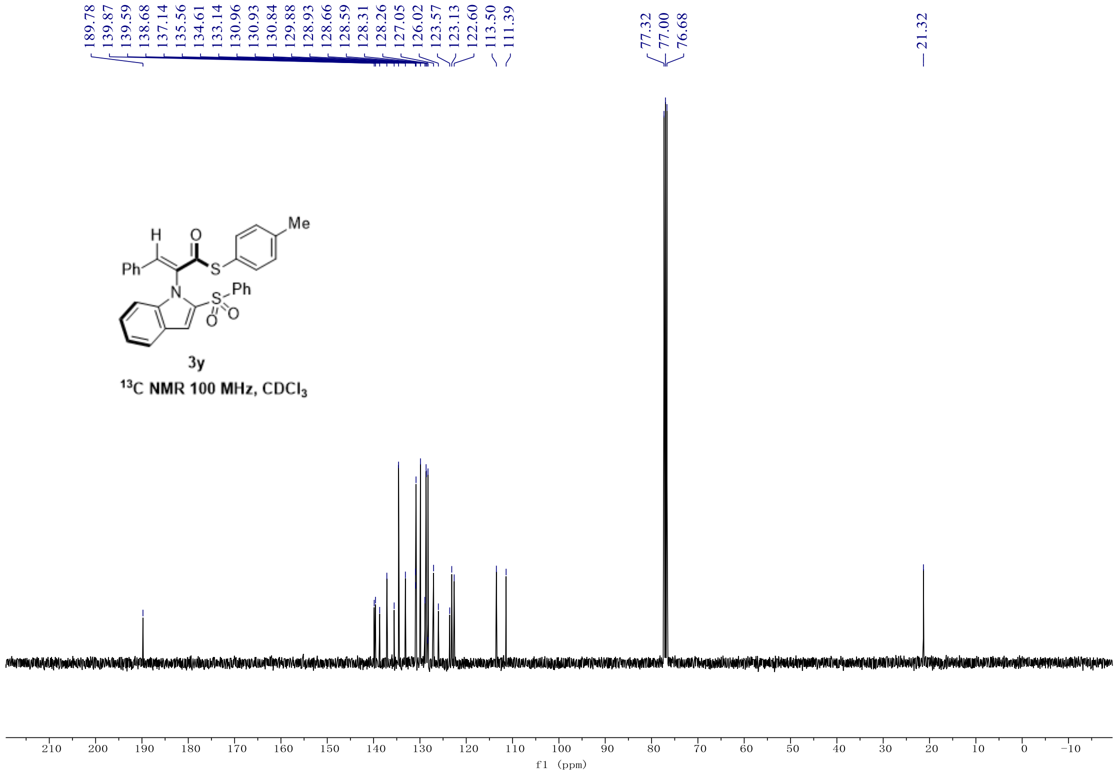
3y
^{13}C NMR 100 MHz, $CDCl_3$

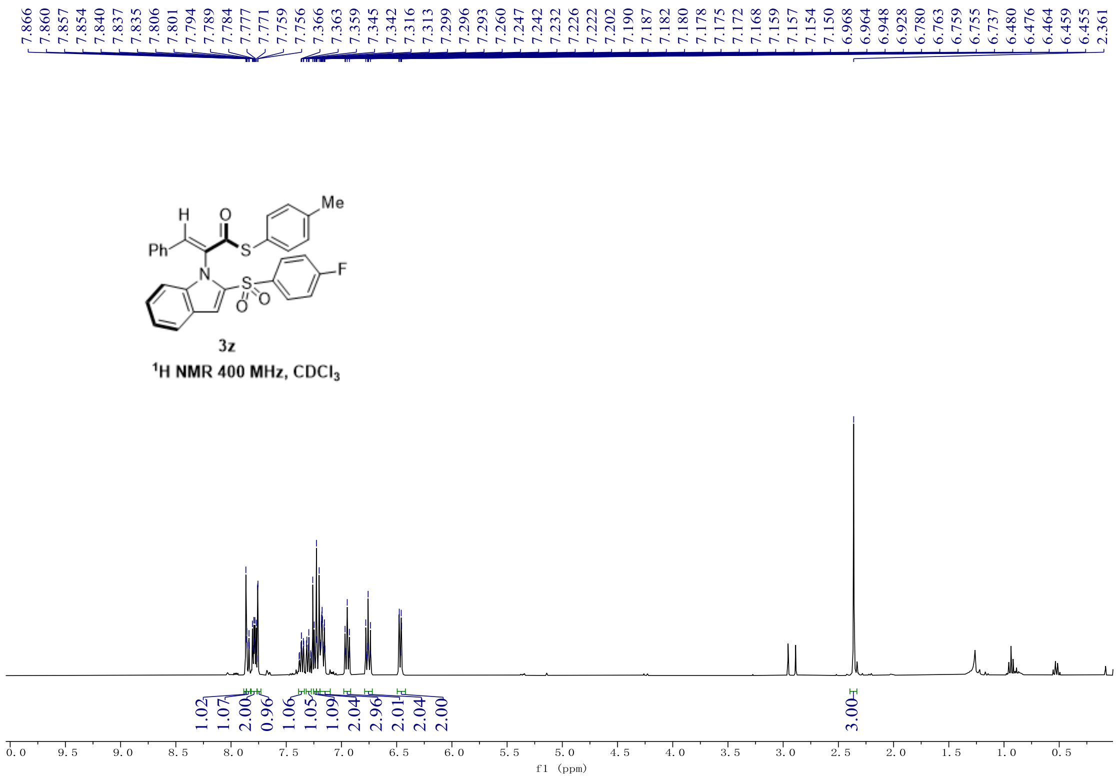
7.866 7.860 7.857 7.854 7.840 7.837 7.835 7.806 7.801 7.794 7.789 7.784 7.777 7.771 7.759 7.756 7.366 7.363 7.359 7.345 7.342 7.316 7.313 7.299 7.296 7.293 7.260 7.247 7.242 7.232 7.226 7.222 7.202 7.190 7.187 7.182 7.180 7.178 7.175 7.172 7.168 7.159 7.157 7.154 7.150 6.968 6.964 6.948 6.928 6.780 6.763 6.759 6.755 6.737 6.480 6.476 6.464 6.459 6.455 2.361
3z
^{1}H NMR 400 MHz, CDCl$_3$
1.02 1.07 2.00 0.96 1.06 1.05 1.09 2.04 2.96 2.01 2.04 2.00 3.00
f1 (ppm)

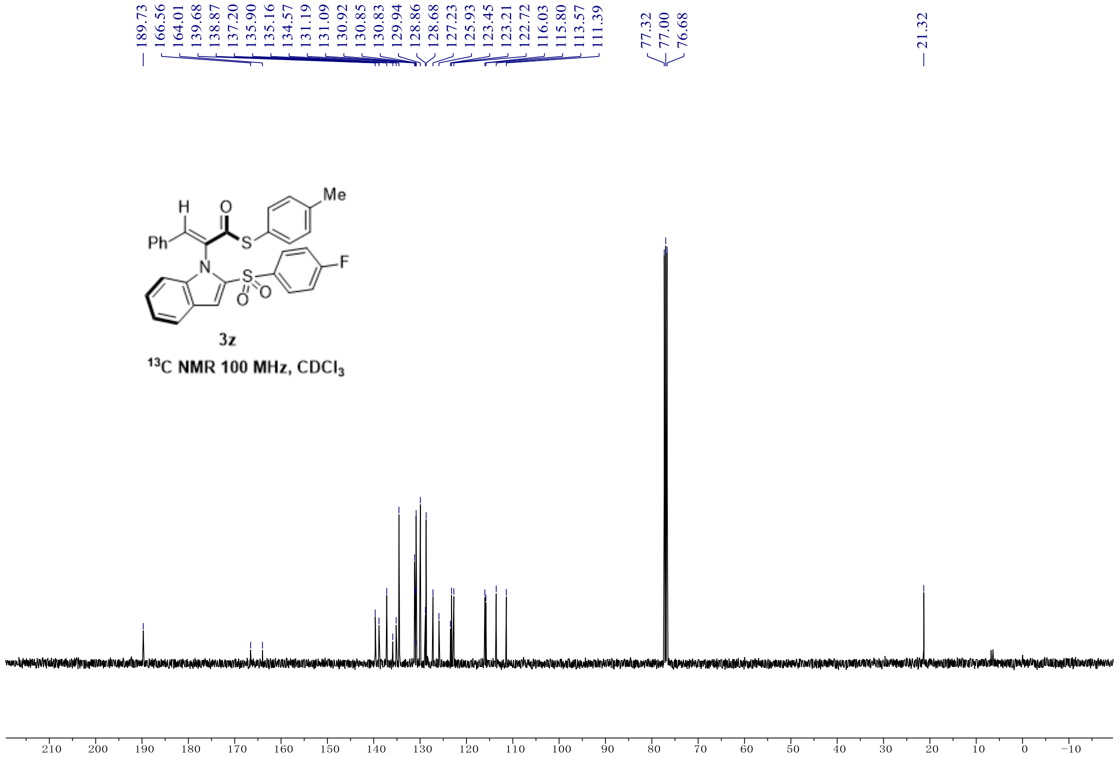
189.73 166.56 164.01 139.68 138.87 137.20 135.90 135.16 134.57 131.19 131.09 130.92 130.85 130.83 129.94 128.86 128.68 127.23 125.93 123.45 123.21 122.72 116.03 115.80 113.57 111.39 77.32 77.00 76.68 21.32
3z
^{13}C NMR 100 MHz, CDCl$_3$
f1 (ppm)

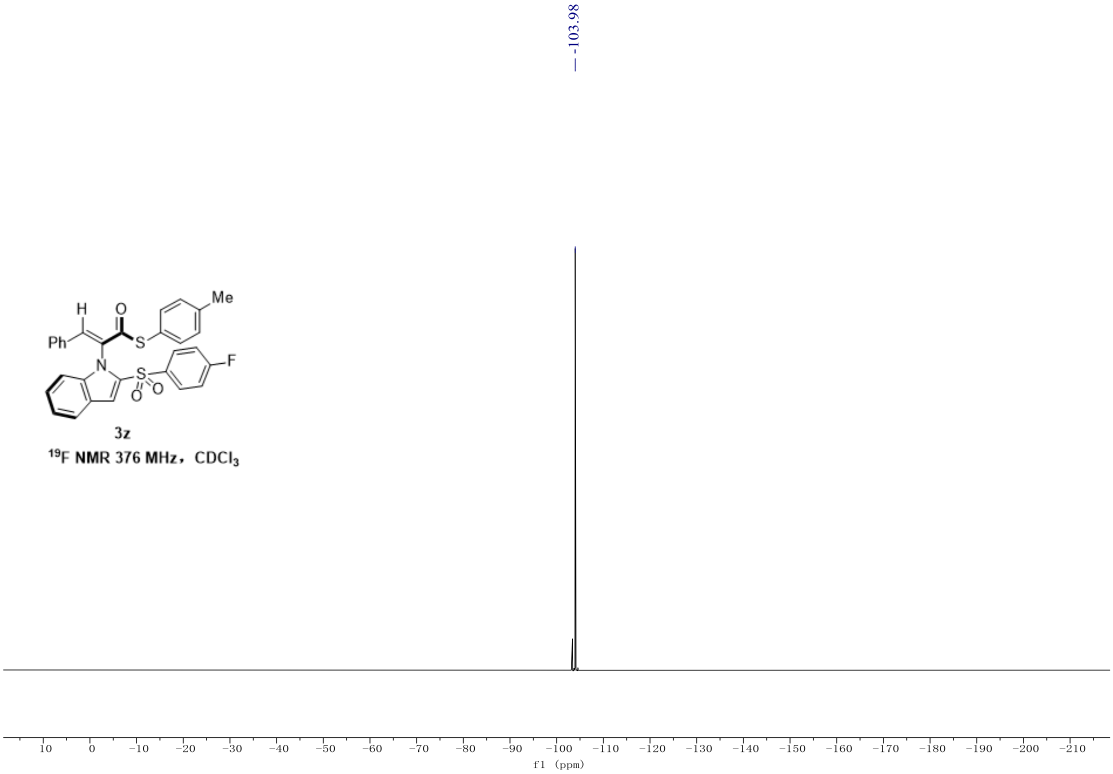

3z

^{19}F NMR 376 MHz，CDCl$_3$

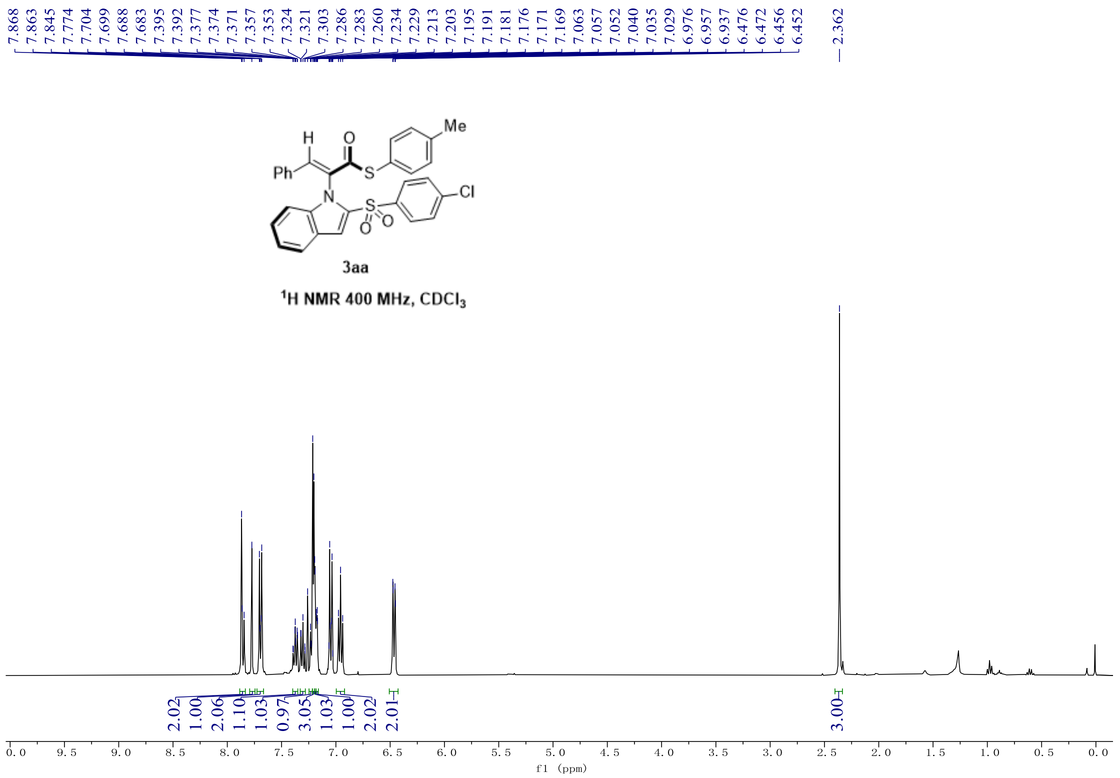
7.868 7.863 7.845 7.774 7.704 7.699 7.688 7.683 7.395 7.392 7.377 7.374 7.371 7.357 7.353 7.324 7.321 7.303 7.286 7.283 7.260 7.234 7.229 7.213 7.203 7.195 7.191 7.181 7.176 7.171 7.169 7.063 7.057 7.052 7.040 7.035 7.029 6.976 6.957 6.937 6.476 6.472 6.456 6.452
2.362
H
O
Me
Ph
S
N
S
Cl
O
O
3aa
^{1}H NMR 400 MHz, CDCl$_3$
2.02 1.00 2.06 1.10 1.03 0.97 3.05 1.03 1.00 2.02 2.01
3.00
f1 (ppm)

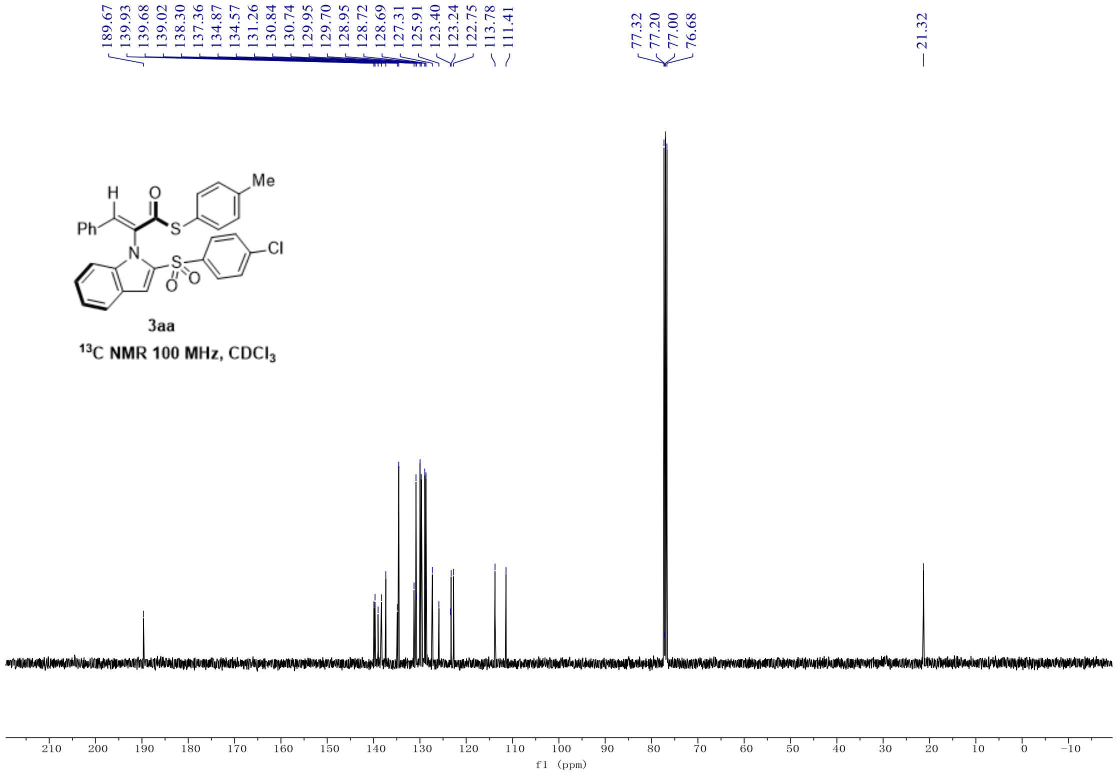
189.67 139.93 139.68 139.02 138.30 137.36 134.87 134.57 131.26 130.84 130.74 129.95 129.70 128.95 128.72 128.69 127.31 125.91 123.40 123.24 122.75 113.78 111.41
77.32 77.20 77.00 76.68
21.32
H
O
Me
Ph
S
N
S
Cl
O
O
3aa
^{13}C NMR 100 MHz, CDCl$_3$
f1 (ppm)

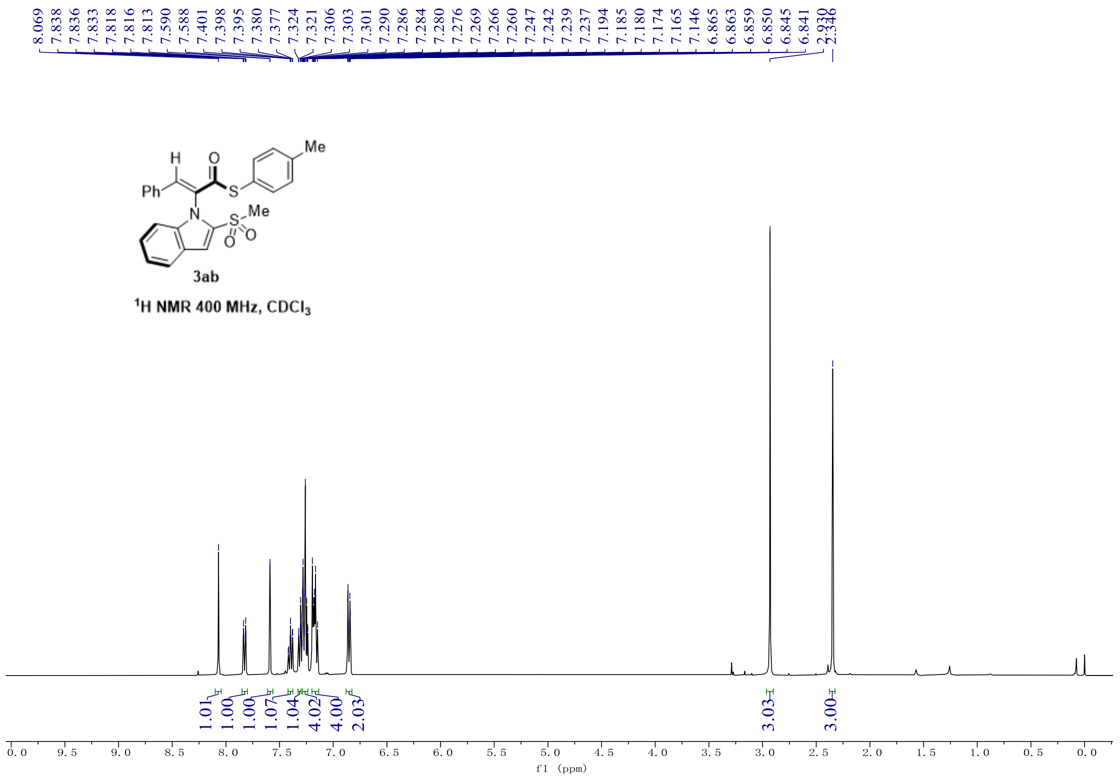

8.069 7.838 7.836 7.833 7.818 7.816 7.813 7.590 7.588 7.401 7.398 7.395 7.380 7.377 7.324 7.321 7.306 7.303 7.301 7.290 7.286 7.284 7.280 7.276 7.269 7.266 7.260 7.247 7.242 7.239 7.237 7.194 7.185 7.180 7.174 7.165 7.146 6.865 6.863 6.859 6.850 6.845 6.841 2.930 2.346
3ab
1H NMR 400 MHz, CDCl3
1.01 1.00 1.00 1.07 1.04 4.02 4.00 2.03 3.03 3.00
f1 (ppm)

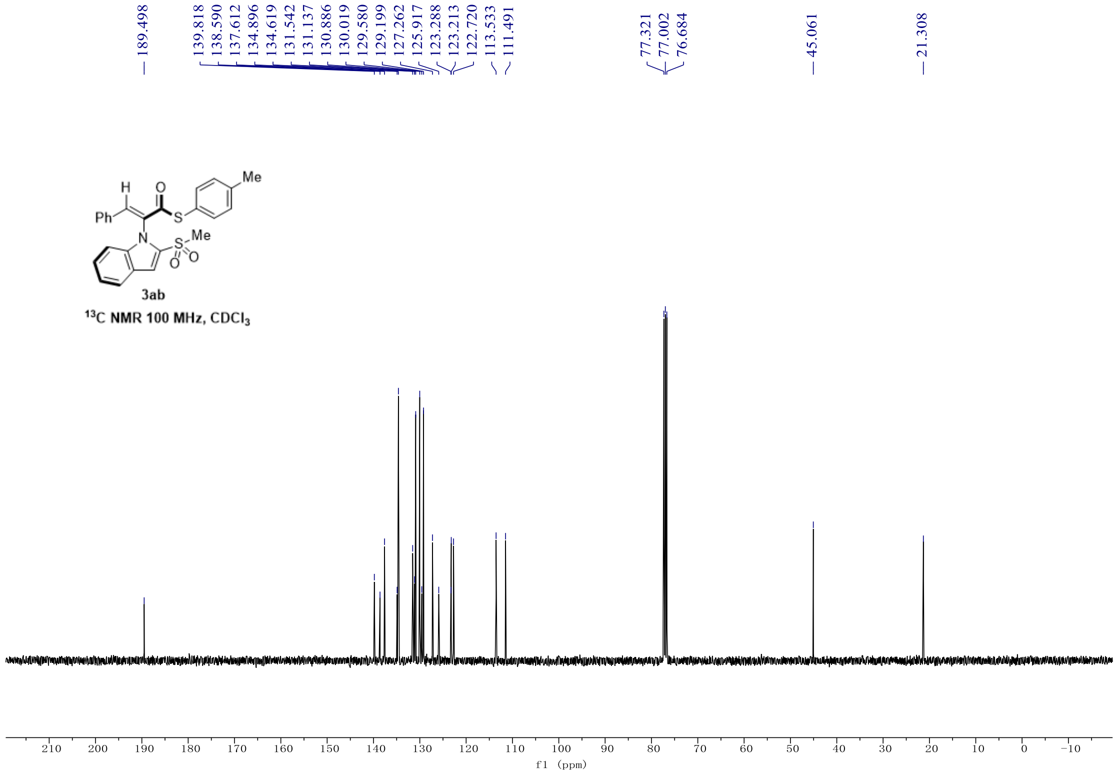

189.498 139.818 138.590 137.612 134.896 134.619 131.542 131.137 130.886 130.019 129.580 129.199 127.262 125.917 123.288 123.213 122.720 113.533 111.491 77.321 77.002 76.684 45.061 21.308
3ab
13C NMR 100 MHz, CDCl3
f1 (ppm)

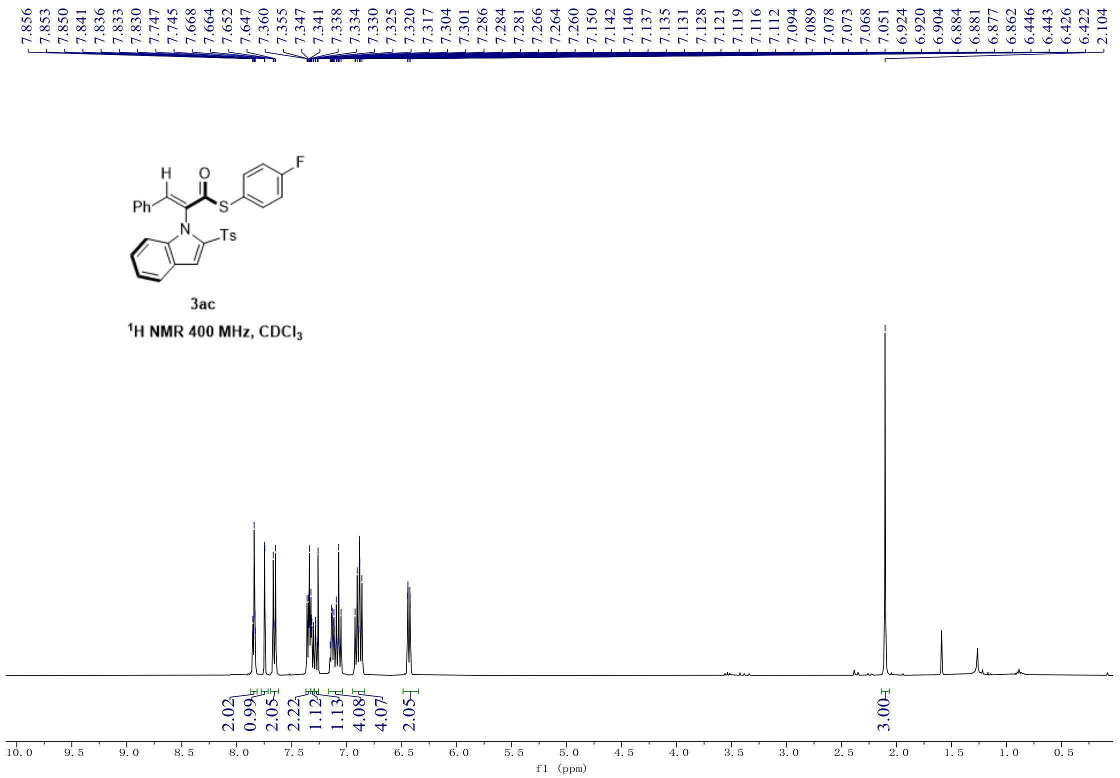
7.856 7.853 7.850 7.841 7.836 7.833 7.830 7.747 7.745 7.668 7.664 7.652 7.647 7.360 7.355 7.347 7.341 7.338 7.334 7.330 7.325 7.320 7.317 7.304 7.301 7.286 7.284 7.281 7.266 7.264 7.260 7.150 7.142 7.140 7.137 7.135 7.131 7.128 7.121 7.119 7.116 7.112 7.094 7.089 7.078 7.073 7.068 7.051 6.924 6.920 6.904 6.884 6.881 6.877 6.862 6.446 6.443 6.426 6.422 2.104
H
O
Ph
S
F
N
Ts
3ac
^{1}H NMR 400 MHz, CDCl$_3$
2.02 0.99 2.05 2.22 1.12 1.13 4.08 4.07 2.05 3.00
10.0 9.5 9.0 8.5 8.0 7.5 7.0 6.5 6.0 5.5 5.0 4.5 4.0 3.5 3.0 2.5 2.0 1.5 1.0 0.5
f1 (ppm)

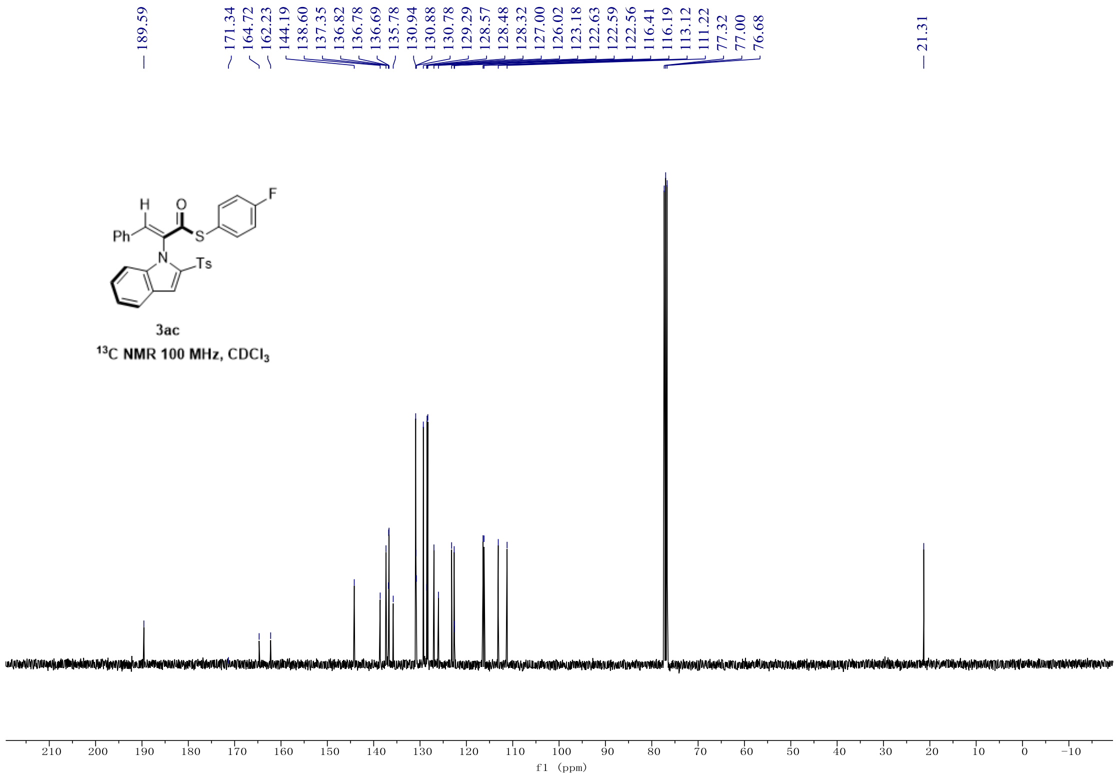
189.59 171.34 164.72 162.23 144.19 138.60 137.35 136.82 136.78 136.69 135.78 130.94 130.88 130.78 129.29 128.57 128.48 128.32 127.00 126.02 123.18 122.63 122.59 122.56 116.41 116.19 113.12 111.22 77.32 77.00 76.68 21.31
H
O
Ph
S
F
N
Ts
3ac
^{13}C NMR 100 MHz, CDCl$_3$
210 200 190 180 170 160 150 140 130 120 110 100 90 80 70 60 50 40 30 20 10 0 -10
f1 (ppm)

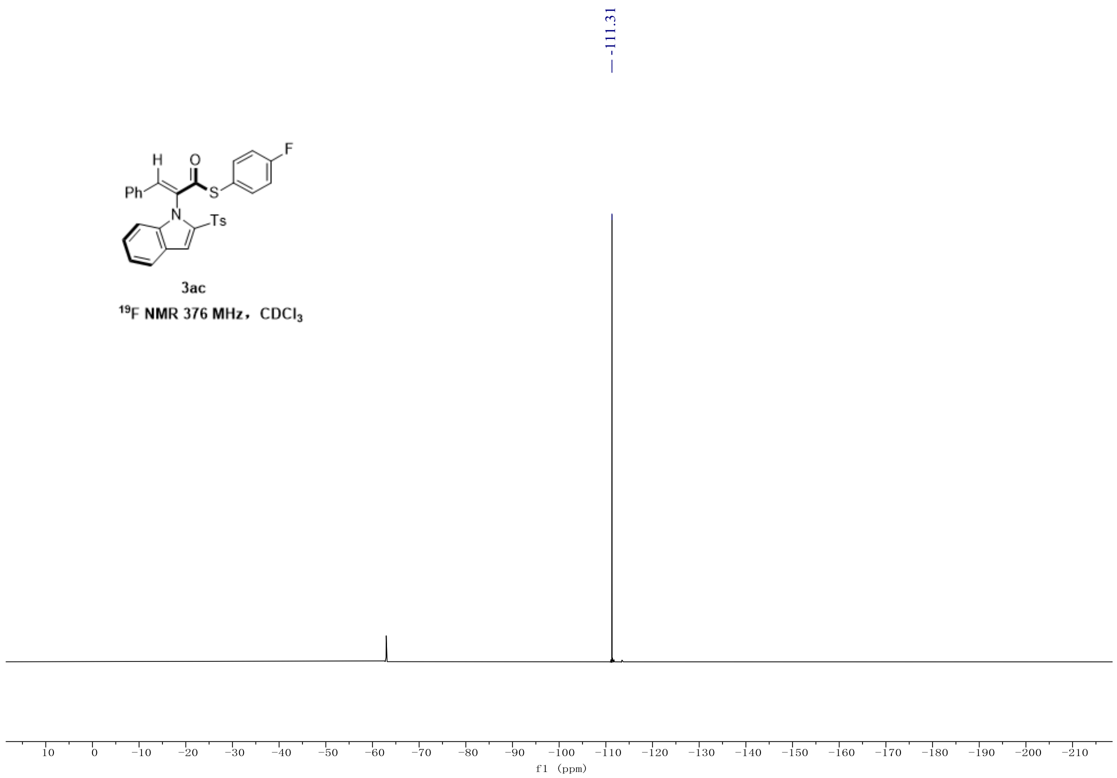
-111.31
H
O
F
Ph
S
N
Ts
3ac
19F NMR 376 MHz，CDCl3
10 0 −10 −20 −30 −40 −50 −60 −70 −80 −90 −100 −110 −120 −130 −140 −150 −160 −170 −180 −190 −200 −210
f1 (ppm)

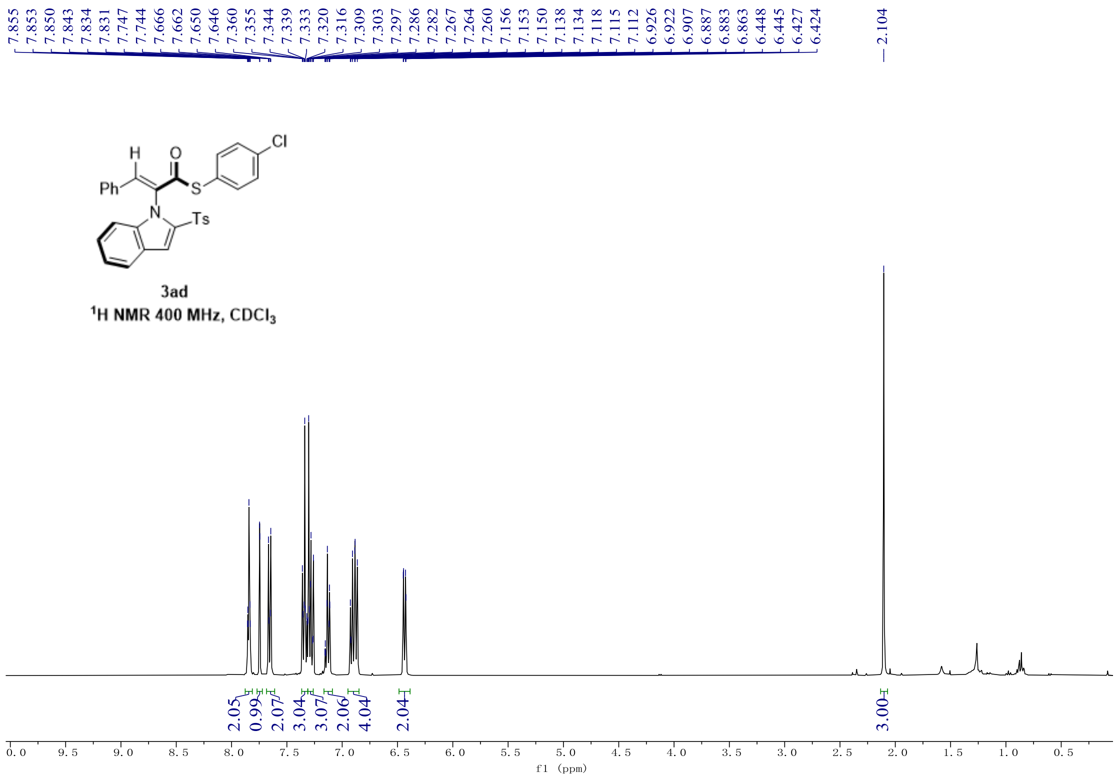
7.855 7.853 7.850 7.843 7.834 7.831 7.747 7.744 7.666 7.662 7.650 7.646 7.360 7.355 7.344 7.339 7.333 7.320 7.316 7.309 7.303 7.297 7.286 7.282 7.267 7.264 7.260 7.156 7.153 7.150 7.138 7.134 7.118 7.115 7.112 6.926 6.922 6.907 6.887 6.883 6.863 6.448 6.445 6.427 6.424
2.104
Ph
H
O
S
Cl
N
Ts
3ad
^{1}H NMR 400 MHz, CDCl$_3$
2.05 0.99 2.07 3.04 3.07 2.06 4.04 2.04
3.00
f1 (ppm)

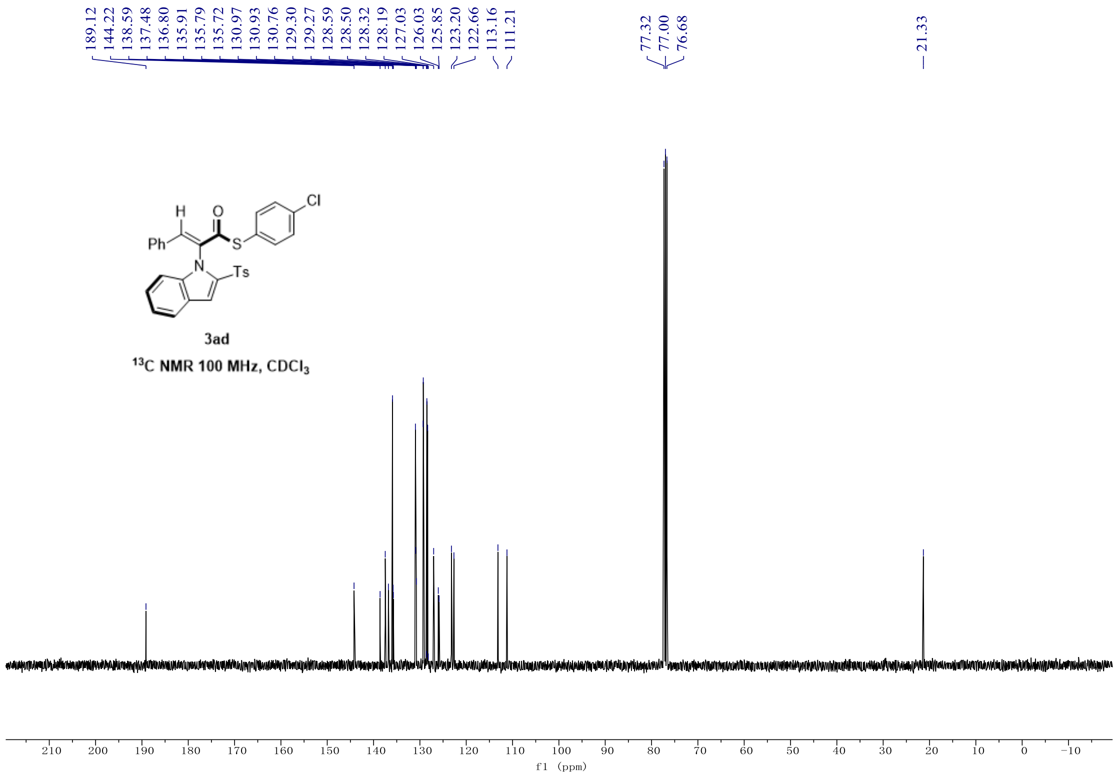
189.12 144.22 138.59 137.48 136.80 135.91 135.79 135.72 130.97 130.93 130.76 129.30 129.27 128.59 128.50 128.32 128.19 127.03 126.03 125.85 123.20 122.66 113.16 111.21
77.32 77.00 76.68
21.33
Ph
H
O
S
Cl
N
Ts
3ad
^{13}C NMR 100 MHz, CDCl$_3$
f1 (ppm)

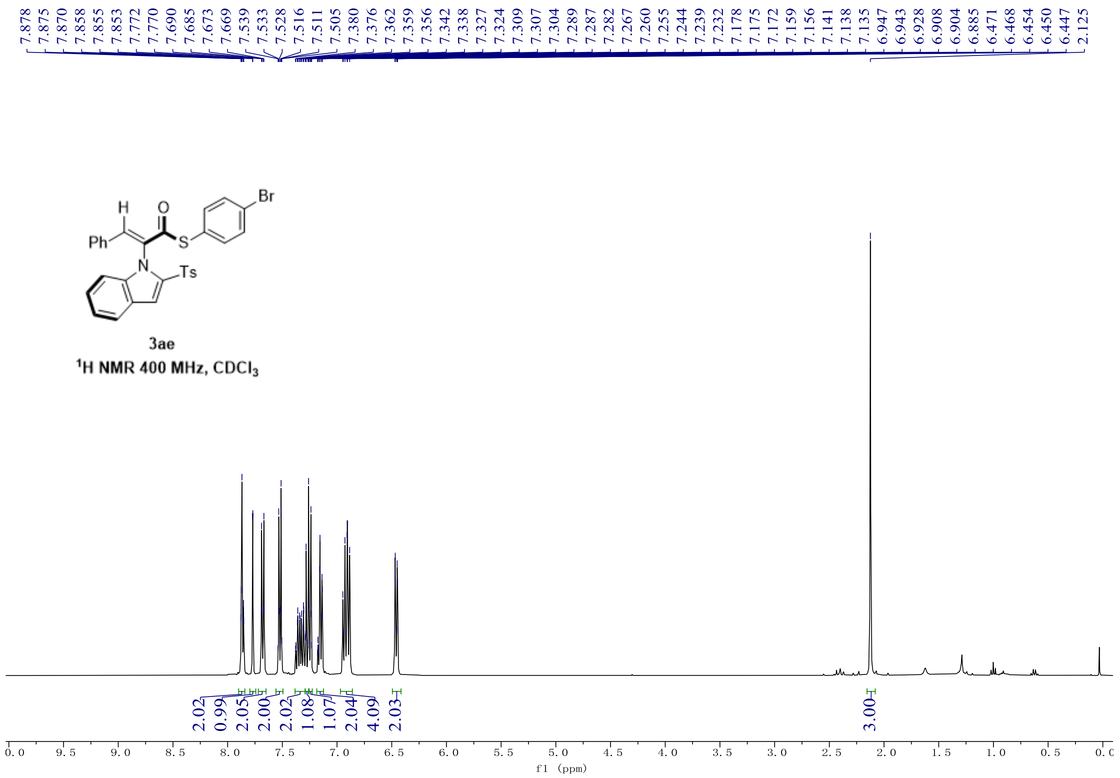

7.878 7.875 7.870 7.858 7.855 7.853 7.772 7.770 7.690 7.685 7.673 7.669 7.539 7.533 7.528 7.516 7.511 7.505 7.380 7.376 7.362 7.359 7.356 7.342 7.338 7.327 7.324 7.309 7.307 7.304 7.289 7.287 7.282 7.267 7.260 7.255 7.244 7.239 7.232 7.178 7.175 7.172 7.159 7.156 7.141 7.138 7.135 6.947 6.943 6.928 6.908 6.904 6.885 6.471 6.468 6.454 6.450 6.447 2.125
H
O
Br
Ph
S
N
Ts
3ae
1H NMR 400 MHz, CDCl3
2.02 0.99 2.05 2.00 2.02 1.08 1.07 2.04 4.09 2.03
3.00
0.0 9.5 9.0 8.5 8.0 7.5 7.0 6.5 6.0 5.5 5.0 4.5 4.0 3.5 3.0 2.5 2.0 1.5 1.0 0.5 0.0
f1 (ppm)

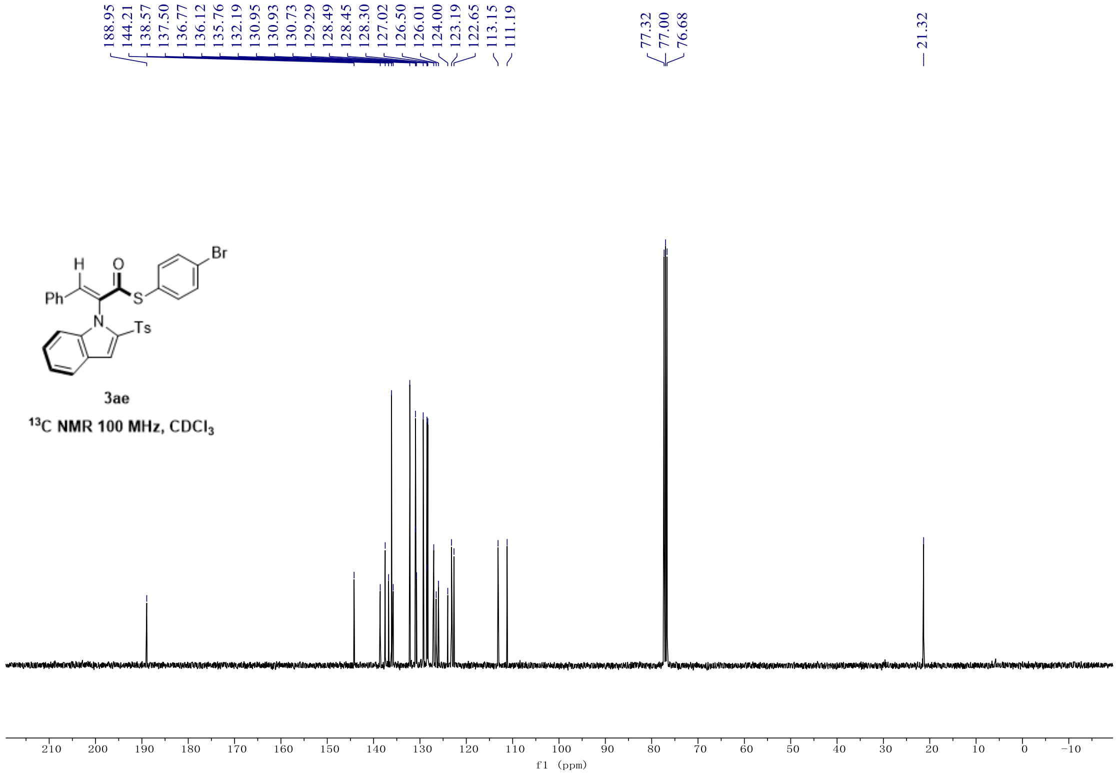

188.95 144.21 138.57 137.50 136.77 136.12 135.76 132.19 130.95 130.93 130.73 129.29 128.49 128.45 128.30 127.02 126.50 126.01 124.00 123.19 122.65 113.15 111.19
77.32 77.00 76.68
21.32
H
O
Br
Ph
S
N
Ts
3ae
13C NMR 100 MHz, CDCl3
210 200 190 180 170 160 150 140 130 120 110 100 90 80 70 60 50 40 30 20 10 0 -10
f1 (ppm)

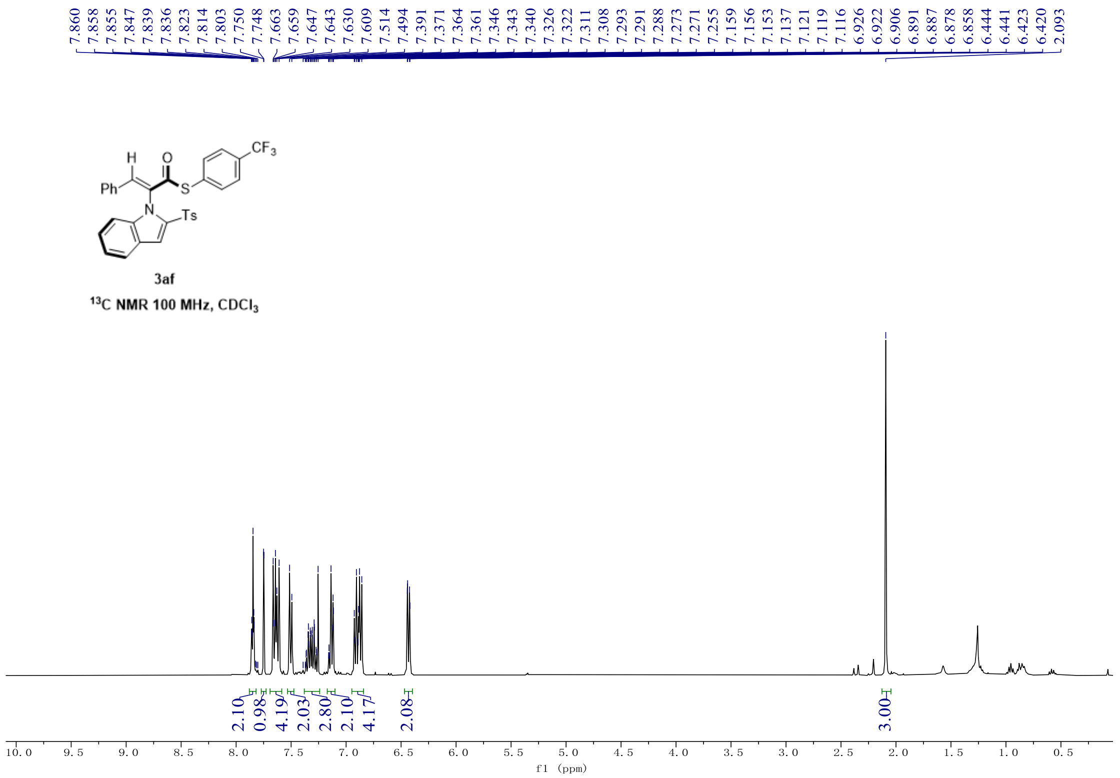
7.860
7.858
7.855
7.847
7.839
7.836
7.823
7.814
7.803
7.750
7.748
7.663
7.659
7.647
7.643
7.630
7.609
7.514
7.494
7.391
7.371
7.364
7.361
7.346
7.343
7.340
7.326
7.322
7.311
7.308
7.293
7.291
7.288
7.273
7.271
7.255
7.159
7.156
7.153
7.137
7.121
7.119
7.116
6.926
6.922
6.906
6.891
6.887
6.878
6.858
6.444
6.441
6.423
6.420
2.093
CF3
H
O
Ph
S
N
Ts
3af
13C NMR 100 MHz, CDCl3
2.10
0.98
4.19
2.03
2.80
2.10
4.17
2.08
3.00
10.0 9.5 9.0 8.5 8.0 7.5 7.0 6.5 6.0 5.5 5.0 4.5 4.0 3.5 3.0 2.5 2.0 1.5 1.0 0.5
f1 (ppm)

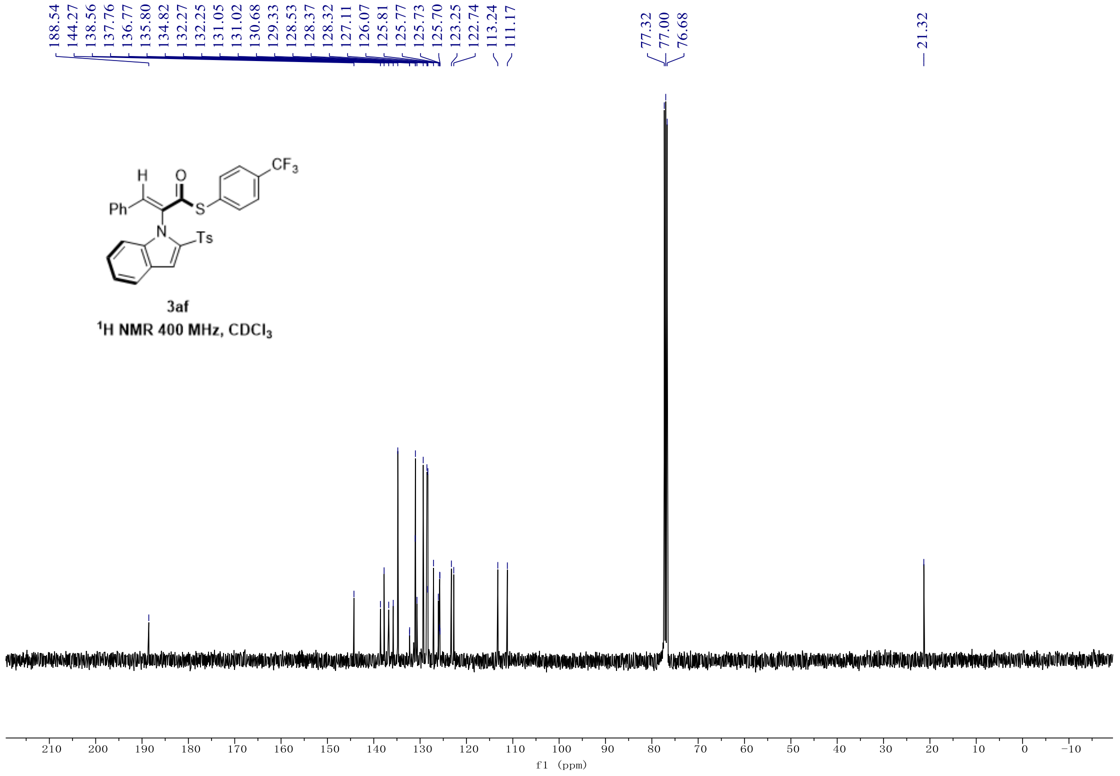
188.54
144.27
138.56
137.76
136.77
135.80
134.82
132.27
132.25
131.05
131.02
130.68
129.33
128.53
128.37
128.32
127.11
126.07
125.81
125.77
125.73
125.70
123.25
122.74
113.24
111.17
77.32
77.00
76.68
21.32
CF3
H
O
Ph
S
N
Ts
3af
1H NMR 400 MHz, CDCl3
210 200 190 180 170 160 150 140 130 120 110 100 90 80 70 60 50 40 30 20 10 0 -10
f1 (ppm)

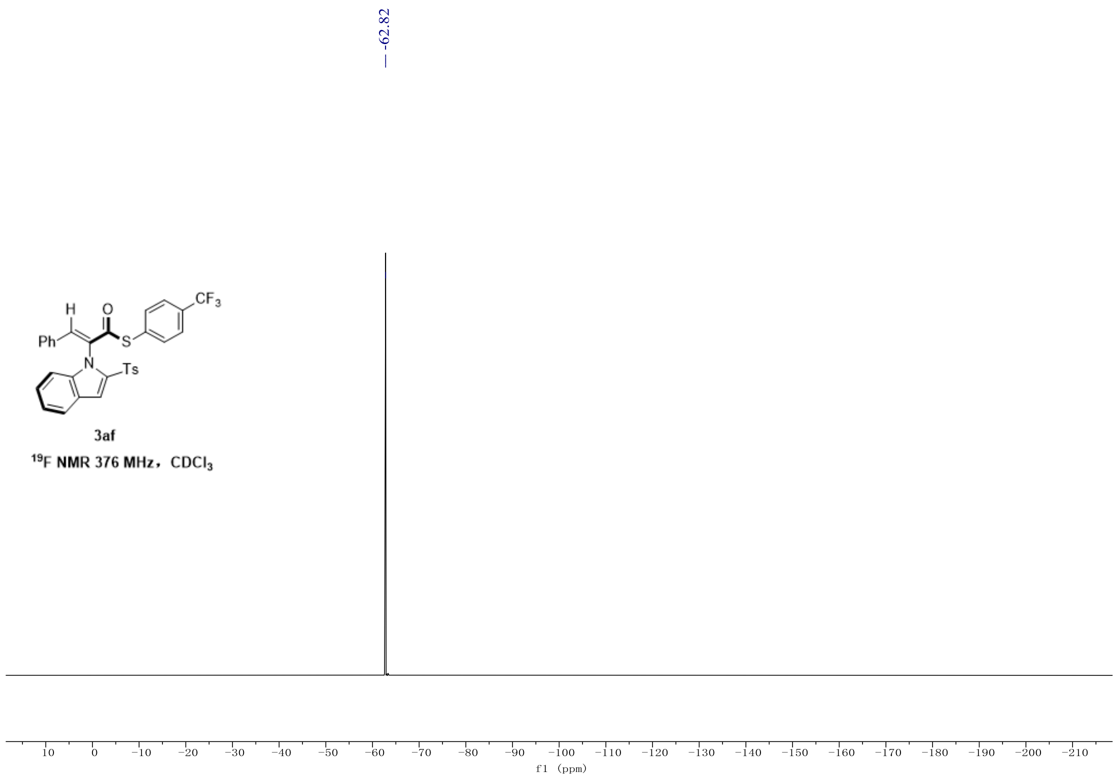
-62.82
CF3
H
O
Ph
S
N
Ts
3af
19F NMR 376 MHz，CDCl3
10 0 −10 −20 −30 −40 −50 −60 −70 −80 −90 −100 −110 −120 −130 −140 −150 −160 −170 −180 −190 −200 −210
f1 (ppm)

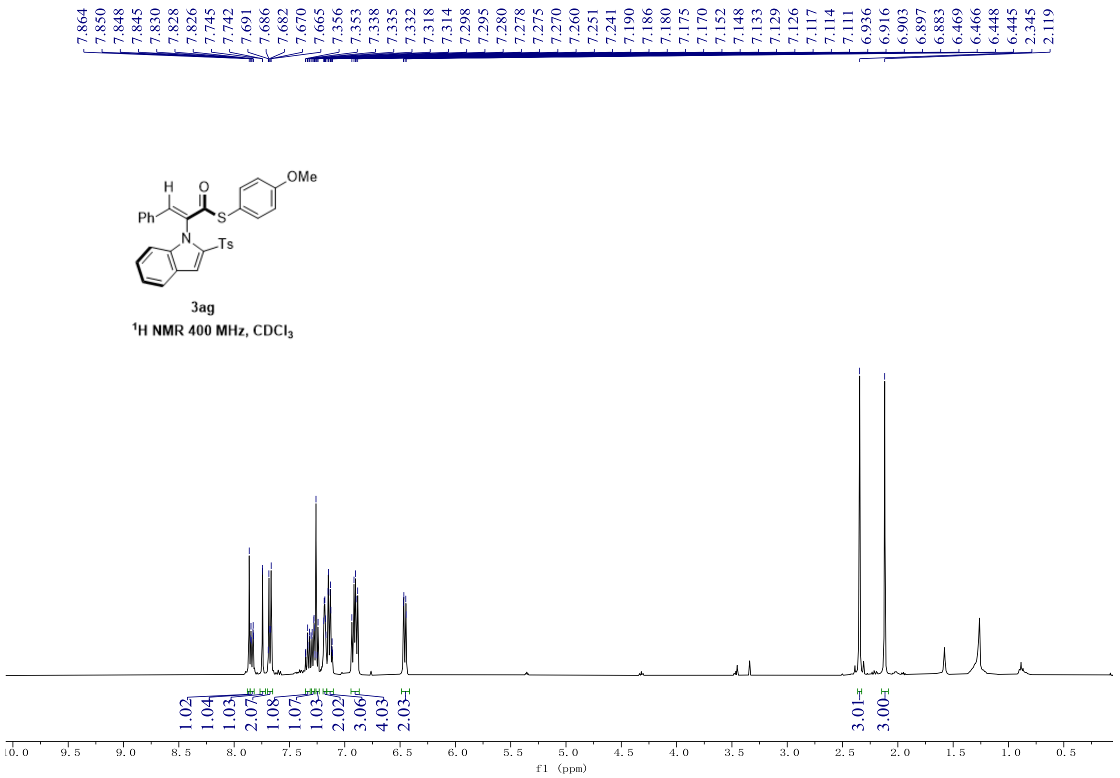
7.864 7.850 7.848 7.845 7.830 7.828 7.826 7.745 7.742 7.691 7.686 7.682 7.670 7.665 7.356 7.353 7.338 7.335 7.332 7.318 7.314 7.298 7.295 7.280 7.278 7.275 7.270 7.260 7.251 7.241 7.190 7.186 7.180 7.175 7.170 7.152 7.148 7.133 7.129 7.126 7.117 7.114 7.111 6.936 6.916 6.903 6.897 6.883 6.469 6.466 6.448 6.445 2.345 2.119
H
O
OMe
Ph
S
N
Ts
3ag
^{1}H NMR 400 MHz, CDCl$_3$
1.02 1.04 1.03 2.07 1.08 1.07 1.03 2.02 3.06 4.03 2.03
3.01 3.00
10.0 9.5 9.0 8.5 8.0 7.5 7.0 6.5 6.0 5.5 5.0 4.5 4.0 3.5 3.0 2.5 2.0 1.5 1.0 0.5
f1 (ppm)

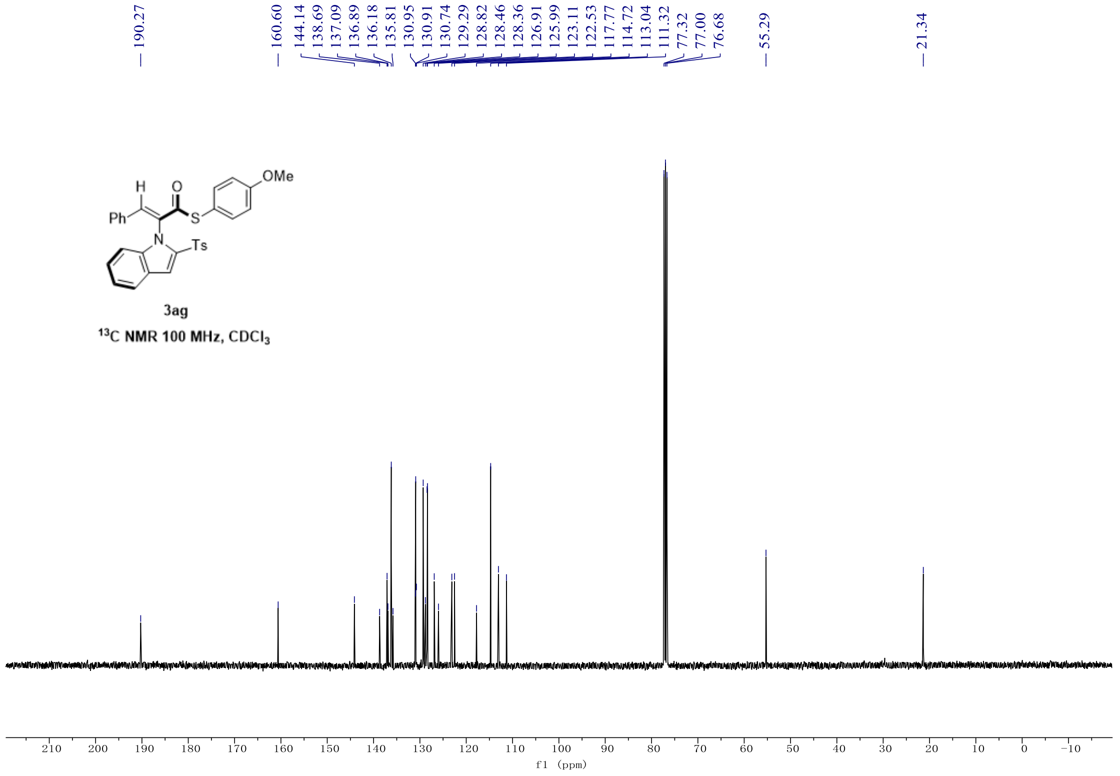
190.27
160.60 144.14 138.69 137.09 136.89 136.18 135.81 130.95 130.91 130.74 129.29 128.82 128.46 128.36 126.91 125.99 123.11 122.53 117.77 114.72 113.04 111.32 77.32 77.00 76.68
55.29
21.34
H
O
OMe
Ph
S
N
Ts
3ag
^{13}C NMR 100 MHz, CDCl$_3$
210 200 190 180 170 160 150 140 130 120 110 100 90 80 70 60 50 40 30 20 10 0 -10
f1 (ppm)

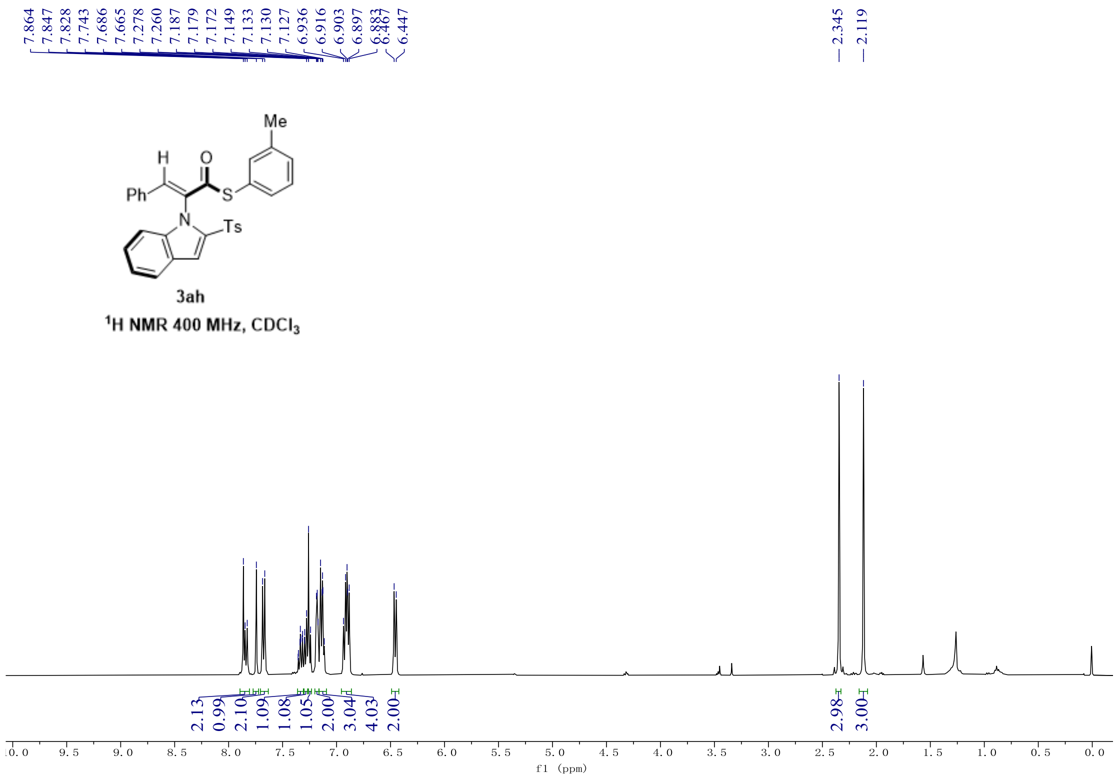
7.864
7.847
7.828
7.743
7.686
7.665
7.278
7.260
7.187
7.179
7.172
7.149
7.133
7.130
7.127
6.936
6.916
6.903
6.897
6.883
6.467
6.447
2.345
2.119
Me
H
O
Ph
S
N
Ts
3ah
^{1}H NMR 400 MHz, CDCl$_3$
2.13
0.99
2.10
1.09
1.08
1.05
2.00
3.04
4.03
2.00
2.98
3.00
f1 (ppm)

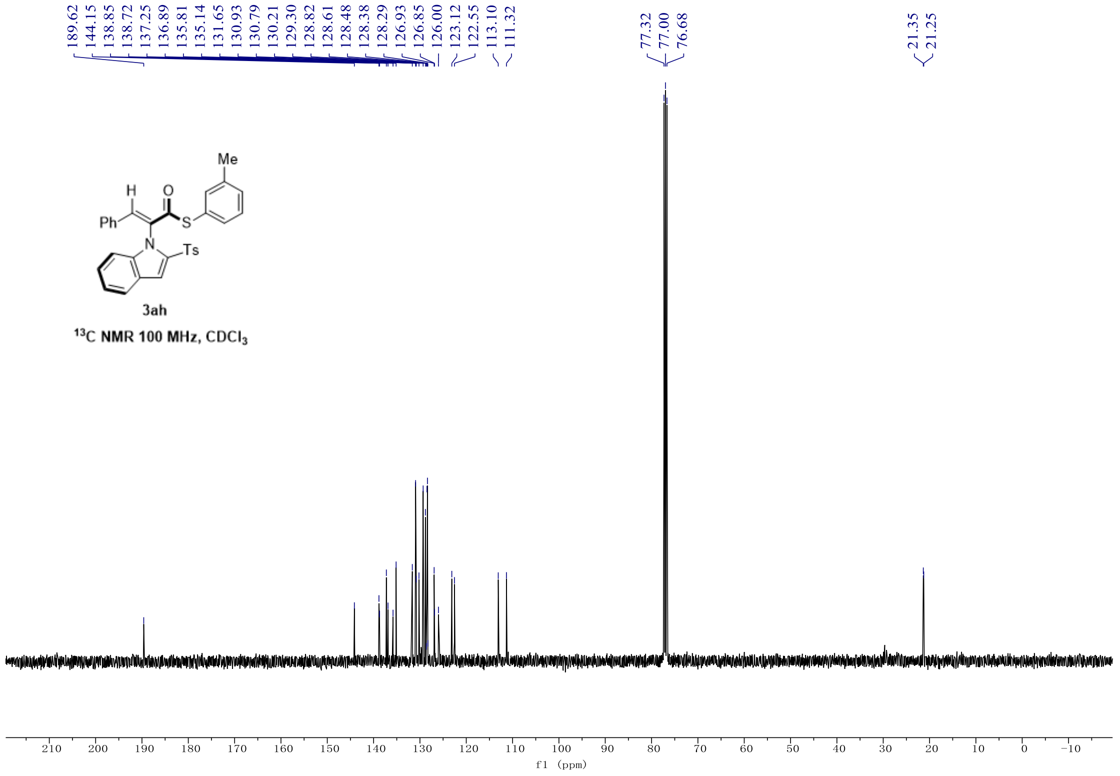
189.62
144.15
138.85
138.72
137.25
136.89
135.81
135.14
131.65
130.93
130.79
130.21
129.30
128.82
128.61
128.48
128.38
128.29
126.93
126.85
126.00
123.12
122.55
113.10
111.32
77.32
77.00
76.68
21.35
21.25
Me
H
O
Ph
S
N
Ts
3ah
^{13}C NMR 100 MHz, CDCl$_3$
f1 (ppm)

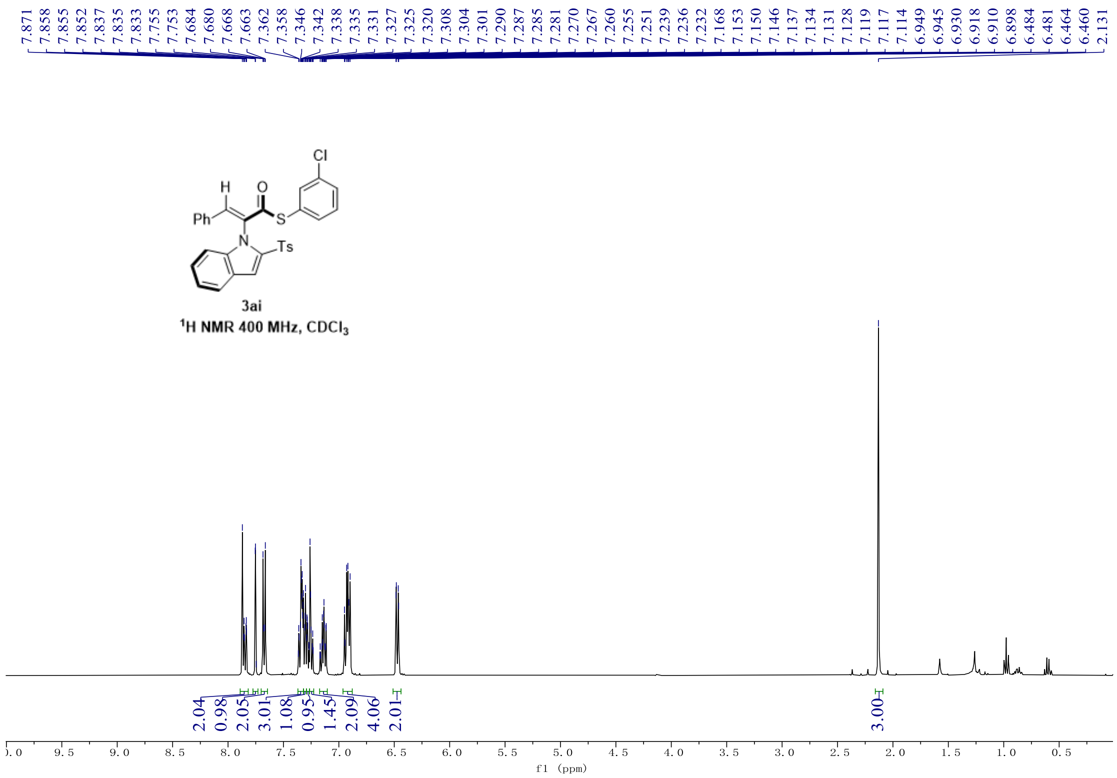
7.871
7.858
7.855
7.852
7.837
7.835
7.833
7.755
7.753
7.684
7.680
7.668
7.663
7.362
7.358
7.346
7.342
7.338
7.335
7.331
7.327
7.325
7.320
7.308
7.304
7.301
7.290
7.287
7.285
7.281
7.270
7.267
7.260
7.255
7.251
7.239
7.236
7.232
7.168
7.153
7.150
7.146
7.137
7.134
7.131
7.128
7.119
7.117
7.114
6.949
6.945
6.930
6.918
6.910
6.898
6.484
6.481
6.464
6.460
2.131
Cl
H
O
Ph
S
N
Ts
3ai
^{1}H NMR 400 MHz, CDCl$_3$
2.04
0.98
2.05
3.01
1.08
0.95
1.45
2.09
4.06
2.01
3.00
f1 (ppm)

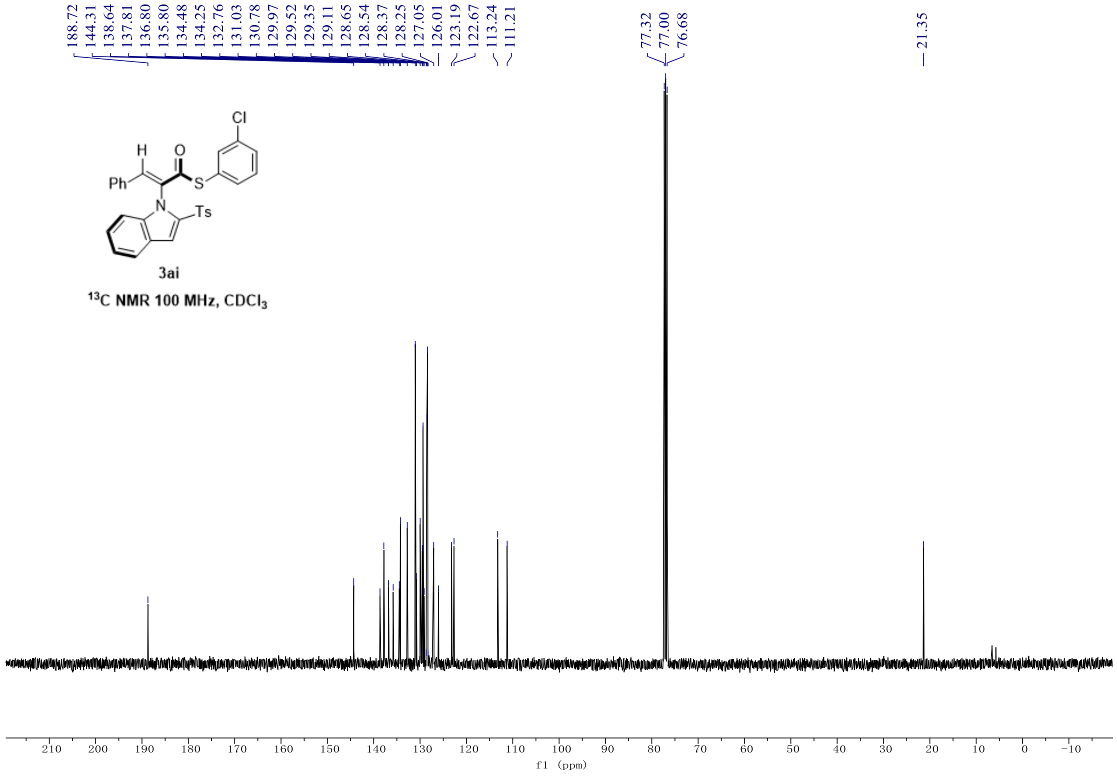
188.72
144.31
138.64
137.81
136.80
135.80
134.48
134.25
132.76
131.03
130.78
129.97
129.52
129.35
129.11
128.65
128.54
128.37
128.25
127.05
126.01
123.19
122.67
113.24
111.21
77.32
77.00
76.68
21.35
Cl
H
O
Ph
S
N
Ts
3ai
^{13}C NMR 100 MHz, CDCl$_3$
f1 (ppm)

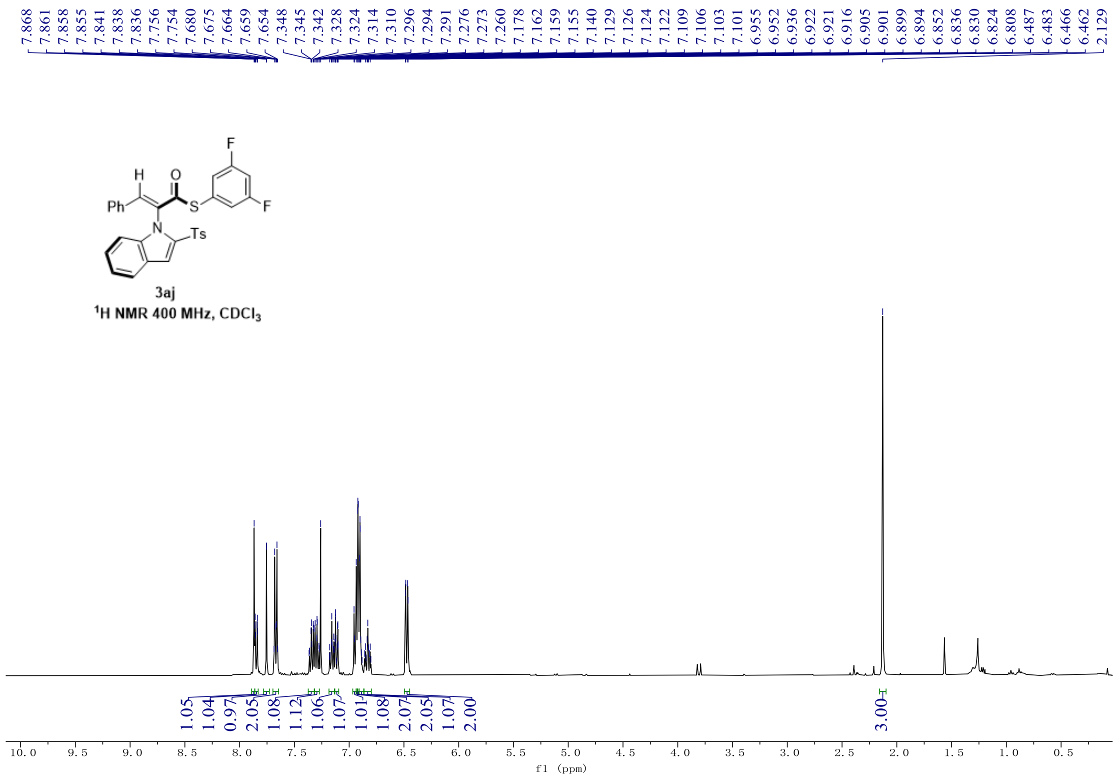
7.868 7.861 7.858 7.855 7.841 7.838 7.836 7.756 7.754 7.680 7.675 7.664 7.659 7.654 7.348 7.345 7.342 7.328 7.324 7.314 7.310 7.296 7.294 7.291 7.276 7.273 7.260 7.178 7.162 7.159 7.155 7.140 7.129 7.126 7.124 7.122 7.109 7.106 7.103 7.101 6.955 6.952 6.936 6.922 6.921 6.916 6.905 6.901 6.899 6.894 6.852 6.836 6.830 6.824 6.808 6.487 6.483 6.466 6.462 2.129
F
H
O
Ph
S
F
N
Ts
3aj
1H NMR 400 MHz, CDCl3
1.05 1.04 0.97 2.05 1.08 1.12 1.06 1.07 1.01 1.08 2.07 2.05 1.07 2.00 3.00
10.0 9.5 9.0 8.5 8.0 7.5 7.0 6.5 6.0 5.5 5.0 4.5 4.0 3.5 3.0 2.5 2.0 1.5 1.0 0.5
f1 (ppm)

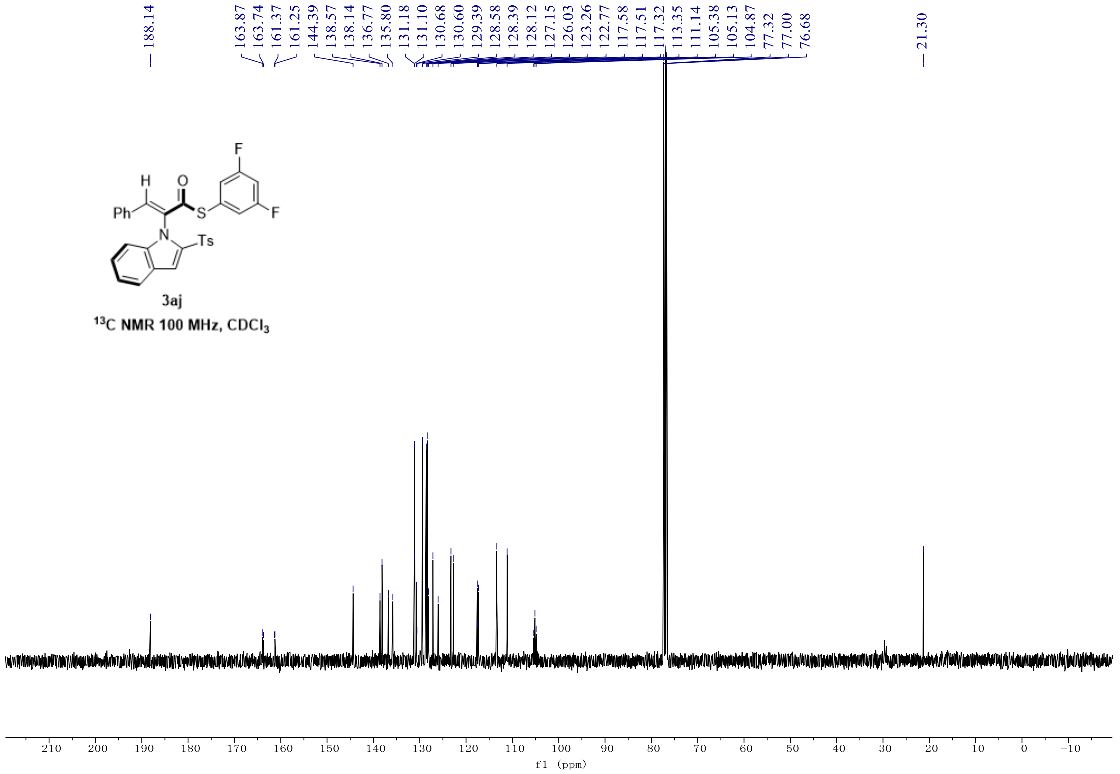
188.14 163.87 163.74 161.37 161.25 144.39 138.57 138.14 136.77 135.80 131.18 131.10 130.68 130.60 129.39 128.58 128.39 128.12 127.15 126.03 123.26 122.77 117.58 117.51 117.32 113.35 111.14 105.38 105.13 104.87 77.32 77.00 76.68 21.30
F
H
O
Ph
S
F
N
Ts
3aj
13C NMR 100 MHz, CDCl3
210 200 190 180 170 160 150 140 130 120 110 100 90 80 70 60 50 40 30 20 10 0 -10
f1 (ppm)

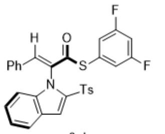

3aj

^{19}F NMR 376 MHz，CDCl$_3$

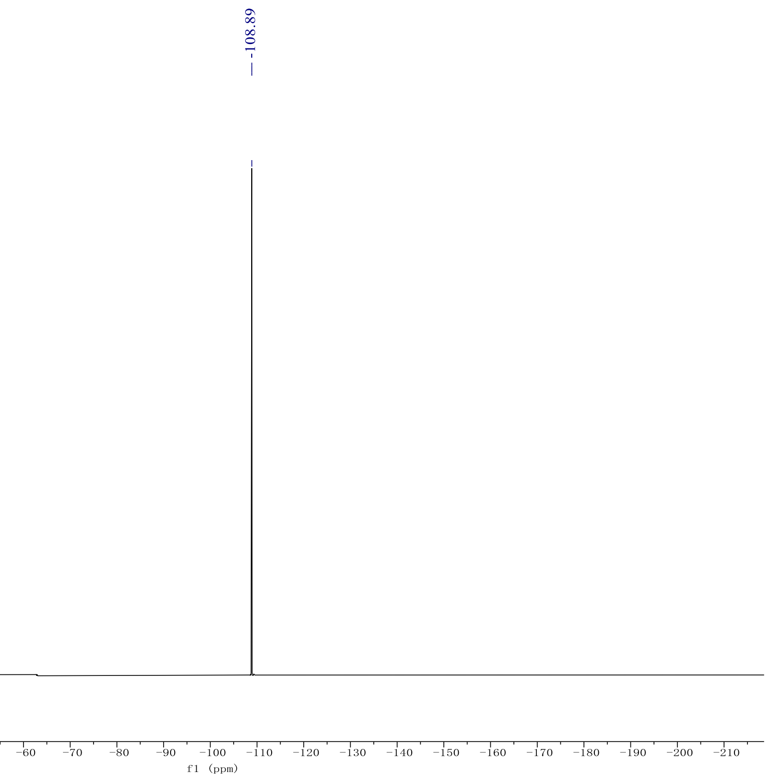

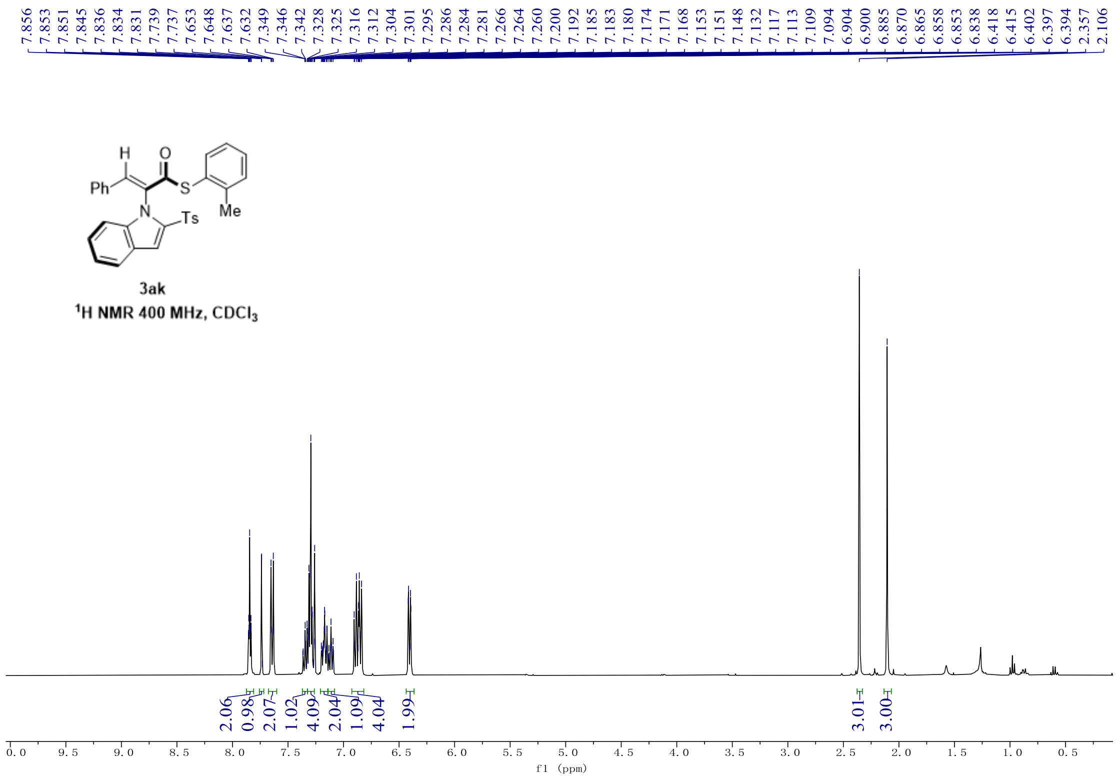
7.856 7.853 7.851 7.845 7.836 7.834 7.831 7.739 7.737 7.653 7.648 7.637 7.632 7.349 7.346 7.342 7.328 7.325 7.316 7.312 7.304 7.301 7.295 7.286 7.284 7.281 7.266 7.264 7.260 7.200 7.192 7.185 7.183 7.180 7.174 7.171 7.168 7.153 7.151 7.148 7.132 7.117 7.113 7.109 7.094 6.904 6.900 6.885 6.870 6.865 6.858 6.853 6.838 6.418 6.415 6.402 6.397 6.394 2.357 2.106
H
O
Ph
S
N
Ts
Me
3ak
1H NMR 400 MHz, CDCl3
2.06 0.98 2.07 1.02 4.09 2.04 1.09 4.04 1.99 3.01 3.00
f1 (ppm)

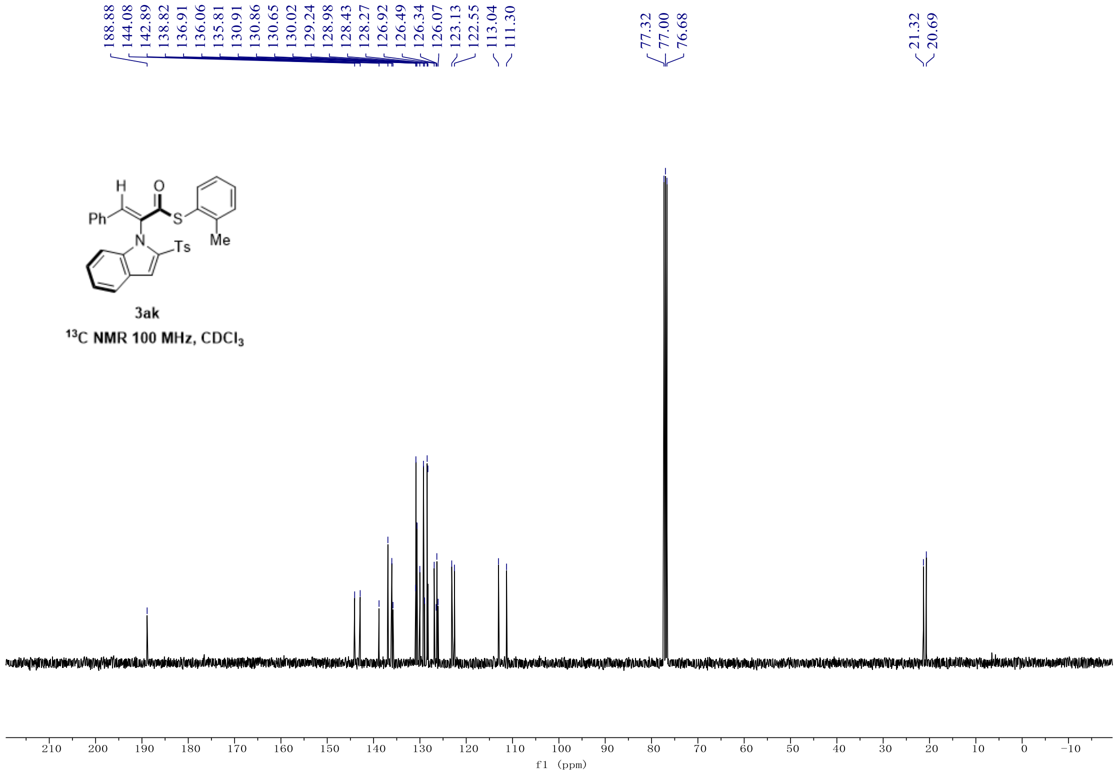
188.88 144.08 142.89 138.82 136.91 136.06 135.81 130.91 130.86 130.65 130.02 129.24 128.98 128.43 128.27 126.92 126.49 126.34 126.07 123.13 122.55 113.04 111.30
77.32 77.00 76.68
21.32 20.69
H
O
Ph
S
N
Ts
Me
3ak
13C NMR 100 MHz, CDCl3
f1 (ppm)

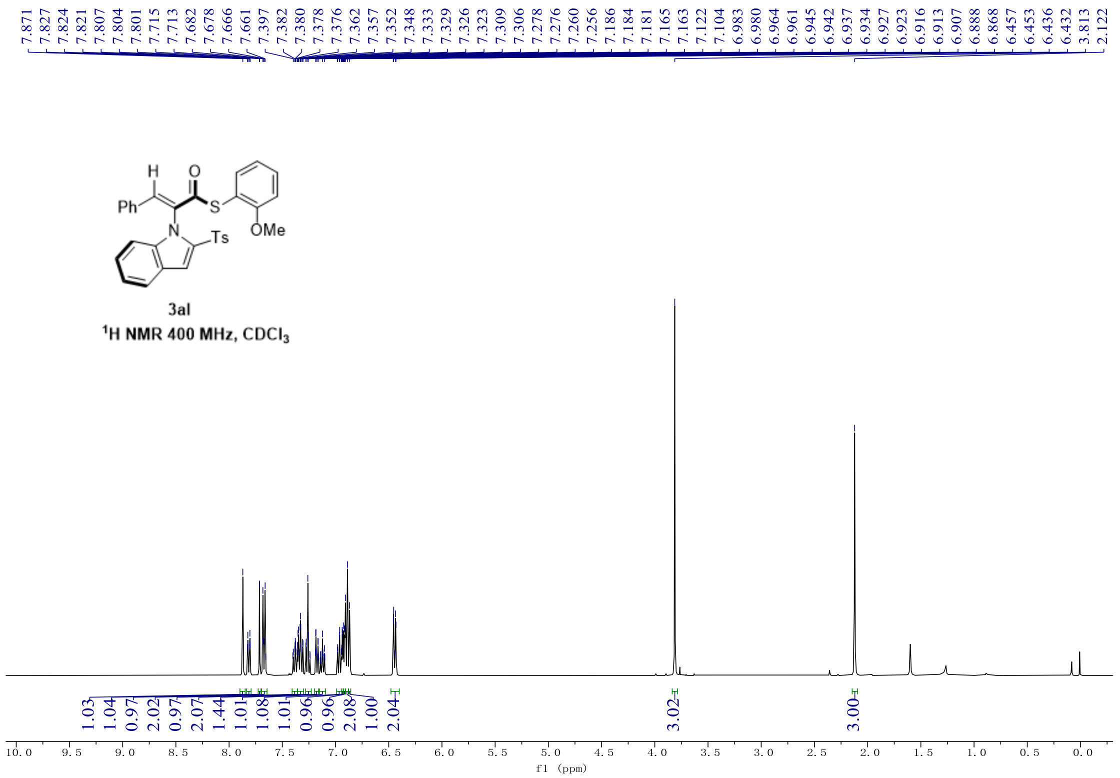
7.871 7.827 7.824 7.821 7.807 7.804 7.801 7.715 7.713 7.682 7.678 7.666 7.661 7.397 7.382 7.380 7.378 7.376 7.362 7.357 7.352 7.348 7.333 7.329 7.326 7.323 7.309 7.306 7.278 7.276 7.260 7.256 7.186 7.184 7.181 7.165 7.163 7.122 7.104 6.983 6.980 6.964 6.961 6.945 6.942 6.937 6.934 6.927 6.923 6.916 6.913 6.907 6.888 6.868 6.457 6.453 6.436 6.432 3.813 2.122
Ph
H
O
S
OMe
N
Ts
3al
1H NMR 400 MHz, CDCl3
1.03 1.04 0.97 2.02 0.97 2.07 1.44 1.01 1.08 1.01 0.96 0.96 2.08 1.00 2.04
3.02
3.00
f1 (ppm)

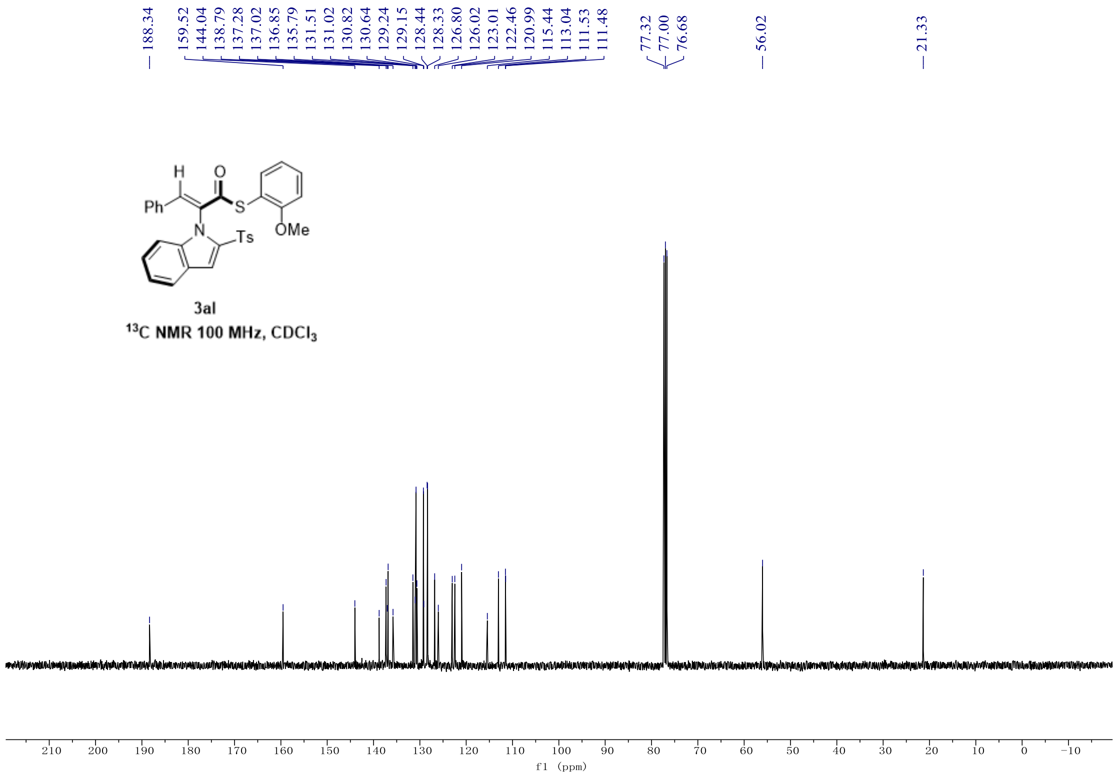
188.34
159.52 144.04 138.79 137.28 137.02 136.85 135.79 131.51 131.02 130.82 130.64 129.24 129.15 128.44 128.33 126.80 126.02 123.01 122.46 120.99 115.44 113.04 111.53 111.48
77.32 77.00 76.68
56.02
21.33
Ph
H
O
S
OMe
N
Ts
3al
13C NMR 100 MHz, CDCl3
f1 (ppm)

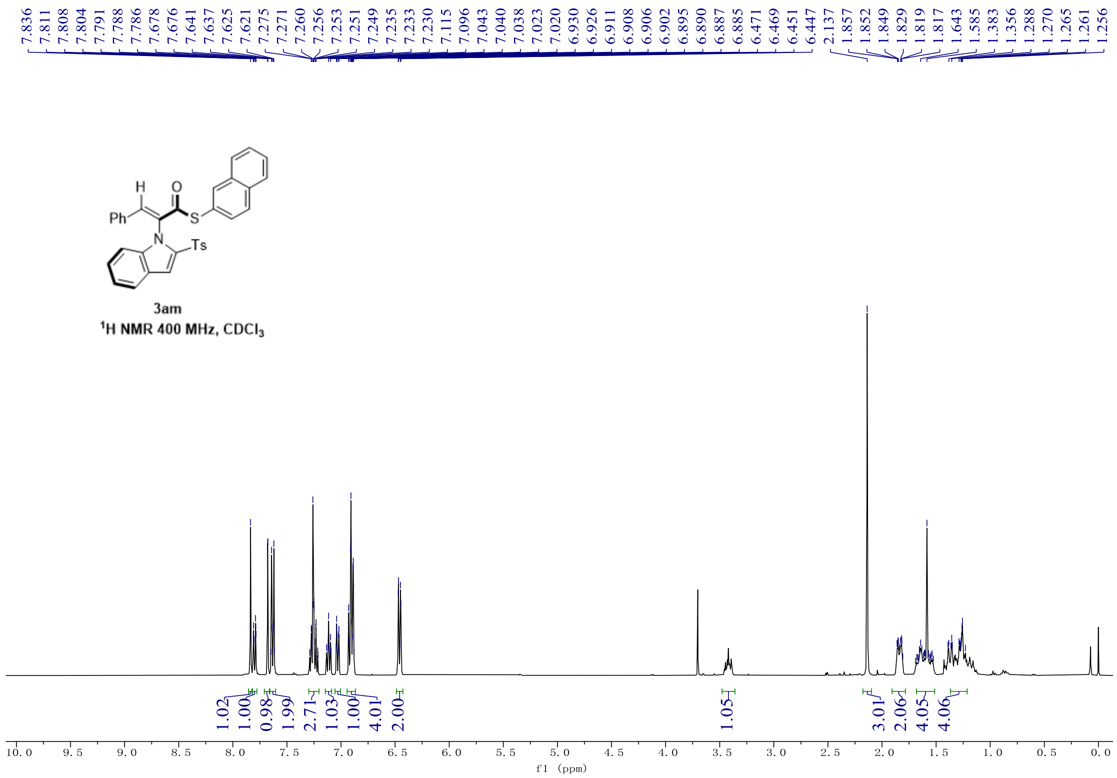
7.836 7.811 7.808 7.804 7.791 7.788 7.786 7.678 7.676 7.641 7.637 7.625 7.621 7.275 7.271 7.260 7.256 7.253 7.251 7.249 7.235 7.233 7.230 7.115 7.096 7.043 7.040 7.038 7.023 7.020 6.930 6.926 6.911 6.908 6.906 6.902 6.895 6.890 6.887 6.885 6.471 6.469 6.451 6.447 2.137 1.857 1.852 1.849 1.829 1.819 1.817 1.643 1.585 1.383 1.356 1.288 1.270 1.265 1.261 1.256
H
O
Ph
S
N
Ts
3am
^{1}H NMR 400 MHz, CDCl$_3$
1.02 1.00 0.98 1.99 2.71 1.03 1.00 4.01 2.00 1.05 3.01 2.06 4.05 4.06
10.0 9.5 9.0 8.5 8.0 7.5 7.0 6.5 6.0 5.5 5.0 4.5 4.0 3.5 3.0 2.5 2.0 1.5 1.0 0.5 0.0
f1 (ppm)

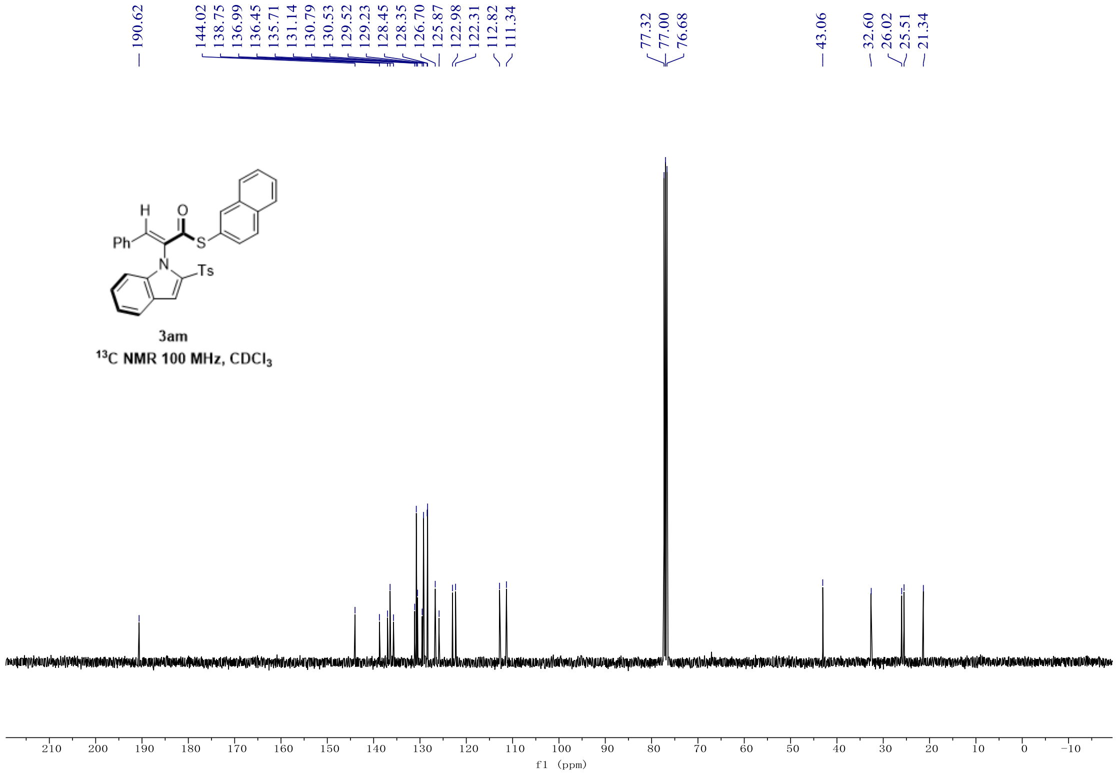
190.62
144.02 138.75 136.99 136.45 135.71 131.14 130.79 130.53 129.52 129.23 128.45 128.35 126.70 125.87 122.98 122.31 112.82 111.34
77.32 77.00 76.68
43.06
32.60 26.02 25.51 21.34
H
O
Ph
S
N
Ts
3am
^{13}C NMR 100 MHz, CDCl$_3$
210 200 190 180 170 160 150 140 130 120 110 100 90 80 70 60 50 40 30 20 10 0 -10
f1 (ppm)

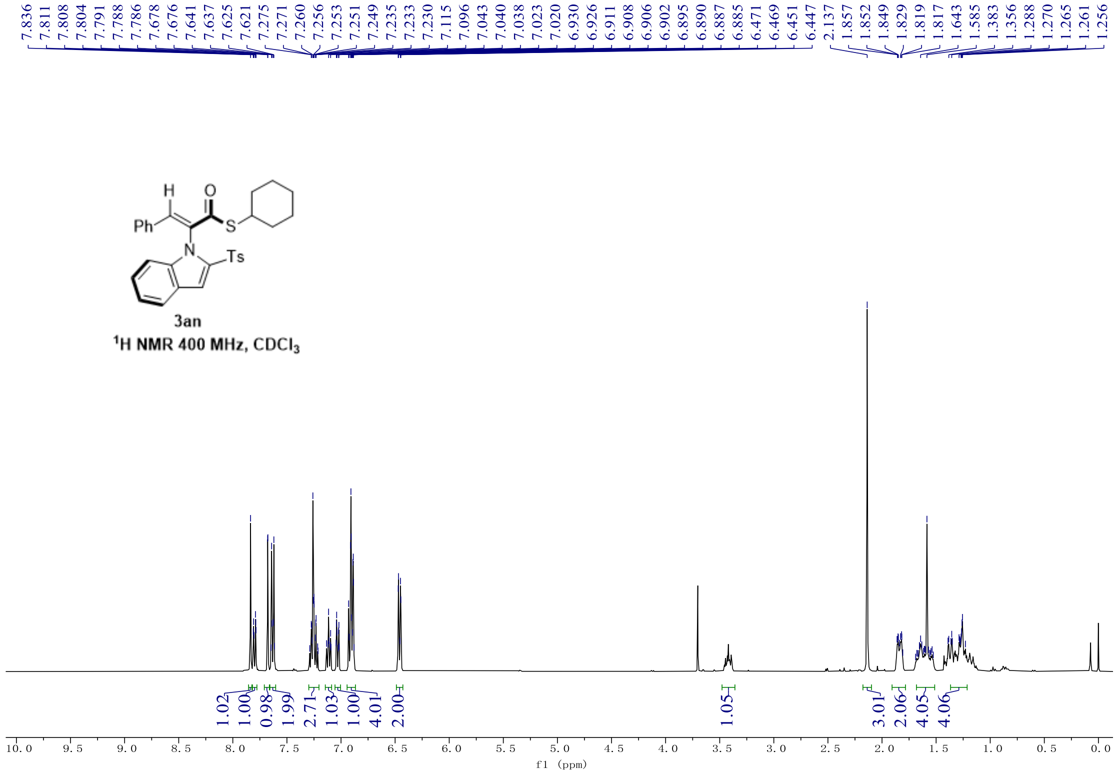
7.836 7.811 7.808 7.804 7.791 7.788 7.786 7.678 7.676 7.641 7.637 7.625 7.621 7.275 7.271 7.260 7.256 7.253 7.251 7.249 7.235 7.233 7.230 7.115 7.096 7.043 7.040 7.038 7.023 7.020 6.930 6.926 6.911 6.908 6.906 6.902 6.895 6.890 6.887 6.885 6.471 6.469 6.451 6.447 2.137 1.857 1.852 1.849 1.829 1.819 1.817 1.643 1.585 1.383 1.356 1.288 1.270 1.265 1.261 1.256
H
O
Ph
S
N
Ts
3an
1H NMR 400 MHz, CDCl3
1.02 1.00 0.98 1.99 2.71 1.03 1.00 4.01 2.00 1.05 3.01 2.06 4.05 4.06
f1 (ppm)

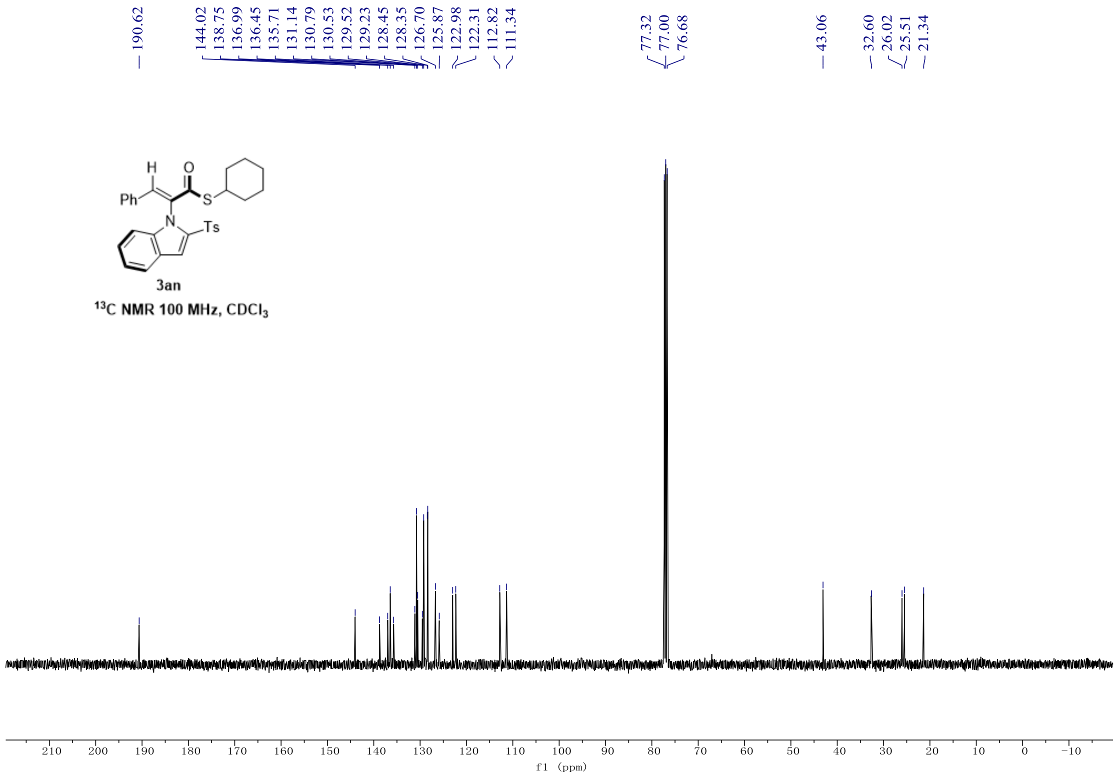
190.62
144.02 138.75 136.99 136.45 135.71 131.14 130.79 130.53 129.52 129.23 128.45 128.35 126.70 125.87 122.98 122.31 112.82 111.34
77.32 77.00 76.68
43.06
32.60 26.02 25.51 21.34
H
O
Ph
S
N
Ts
3an
13C NMR 100 MHz, CDCl3
f1 (ppm)

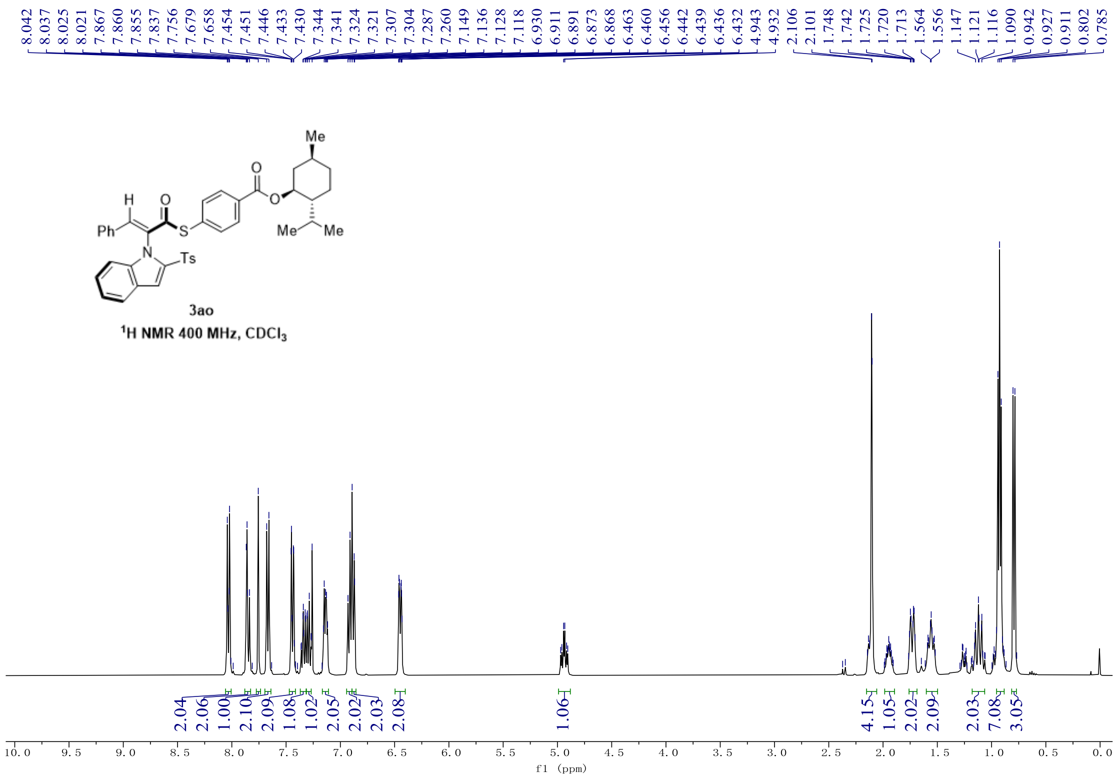
3ao
^{1}H NMR 400 MHz, CDCl$_3$
f1 (ppm)

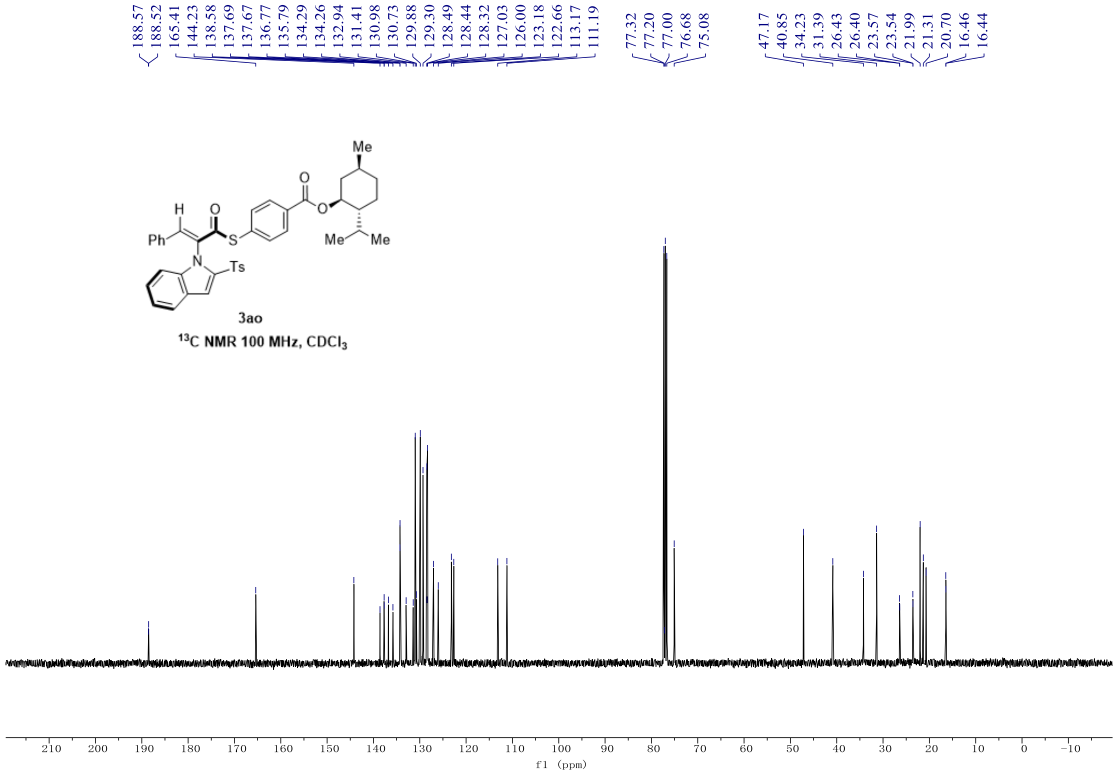
3ao
^{13}C NMR 100 MHz, CDCl$_3$
f1 (ppm)

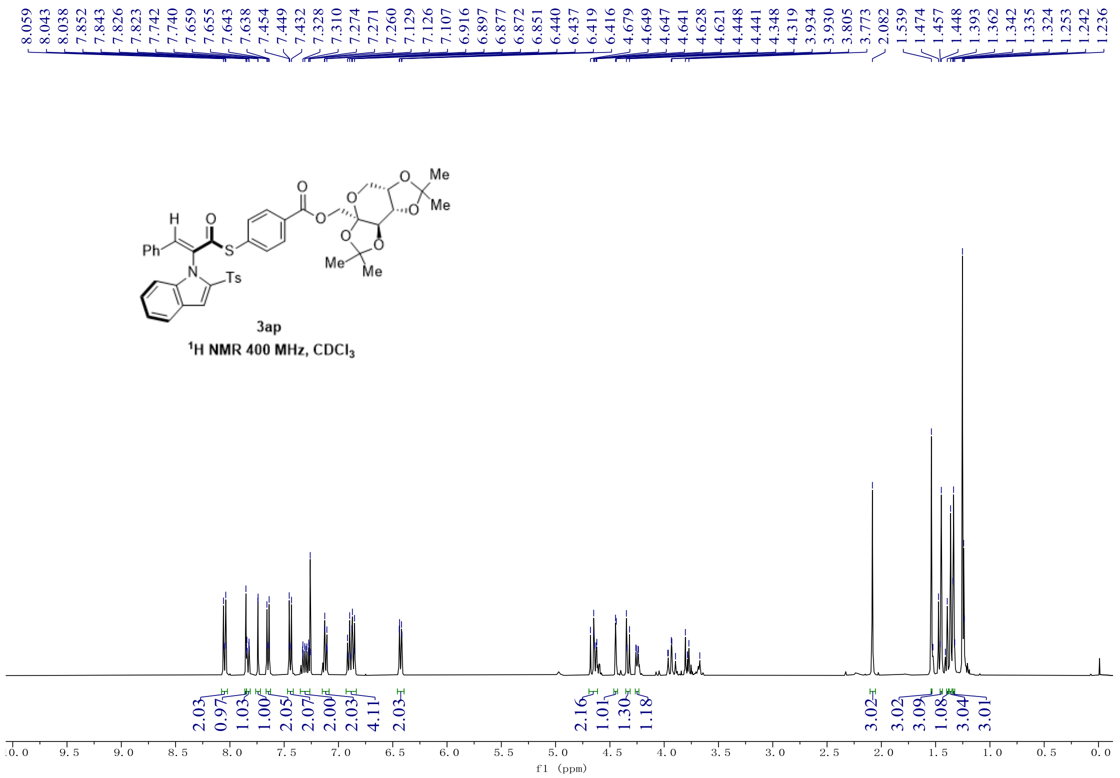
3ap
¹H NMR 400 MHz, CDCl3
f1 (ppm)

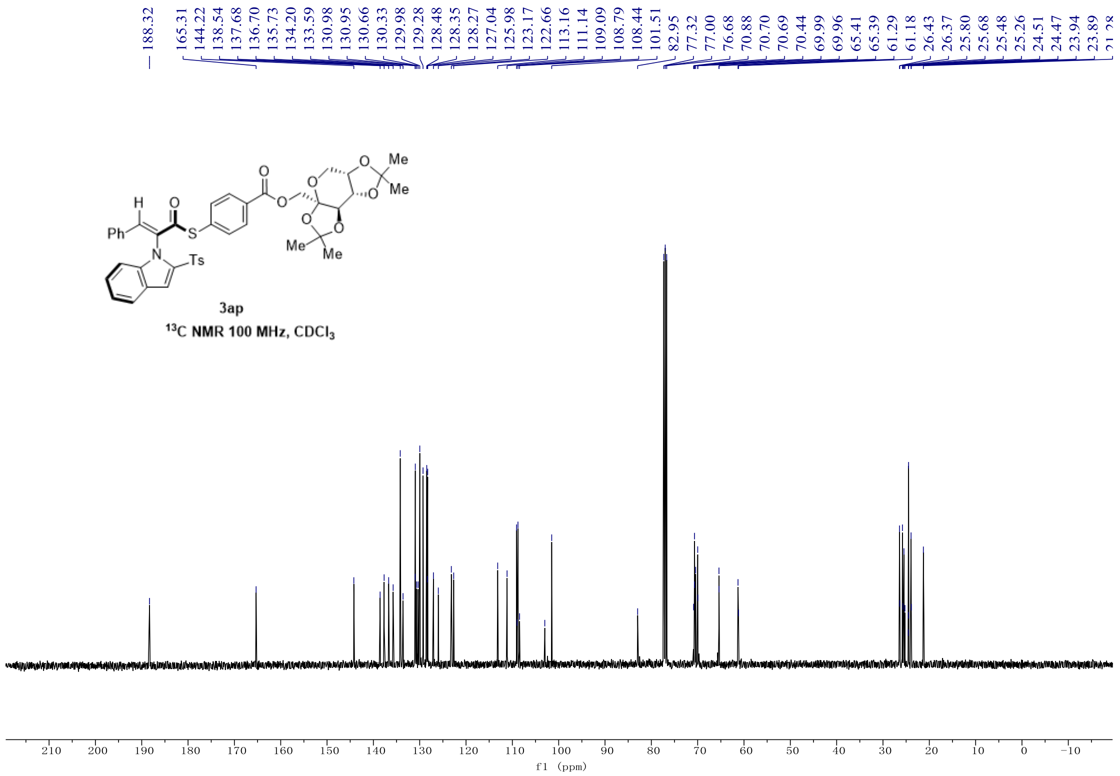
3ap
¹³C NMR 100 MHz, CDCl3
f1 (ppm)

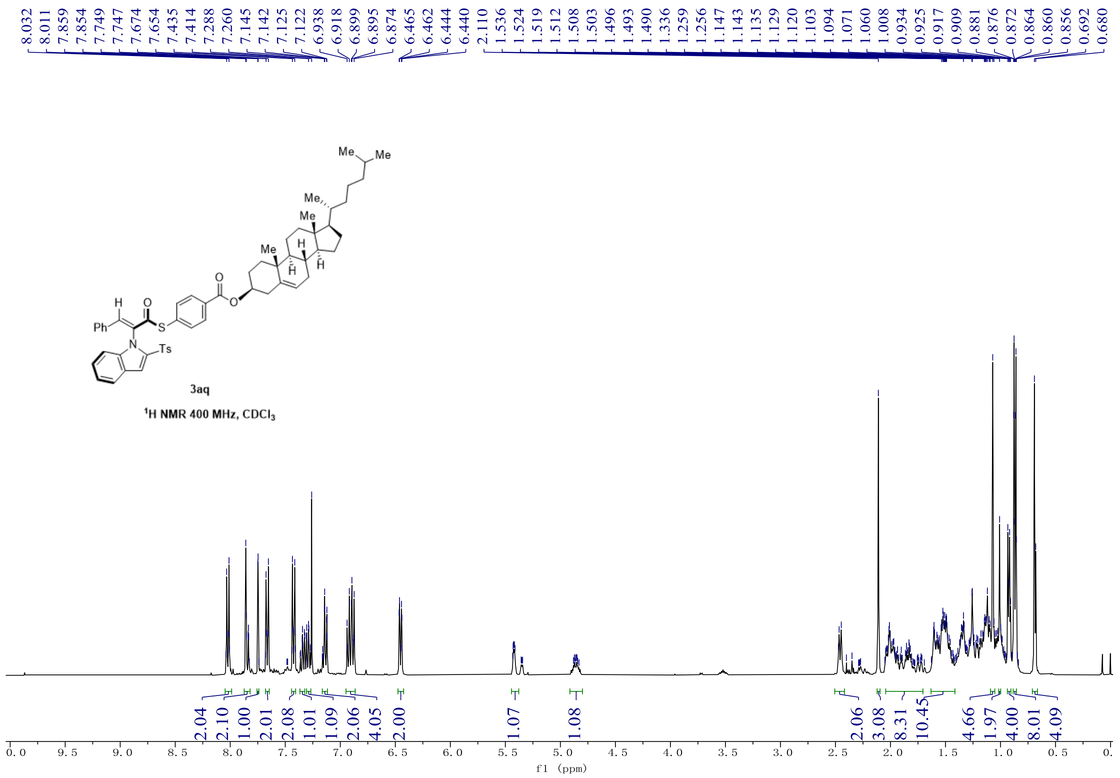
3aq
1H NMR 400 MHz, CDCl3
f1 (ppm)

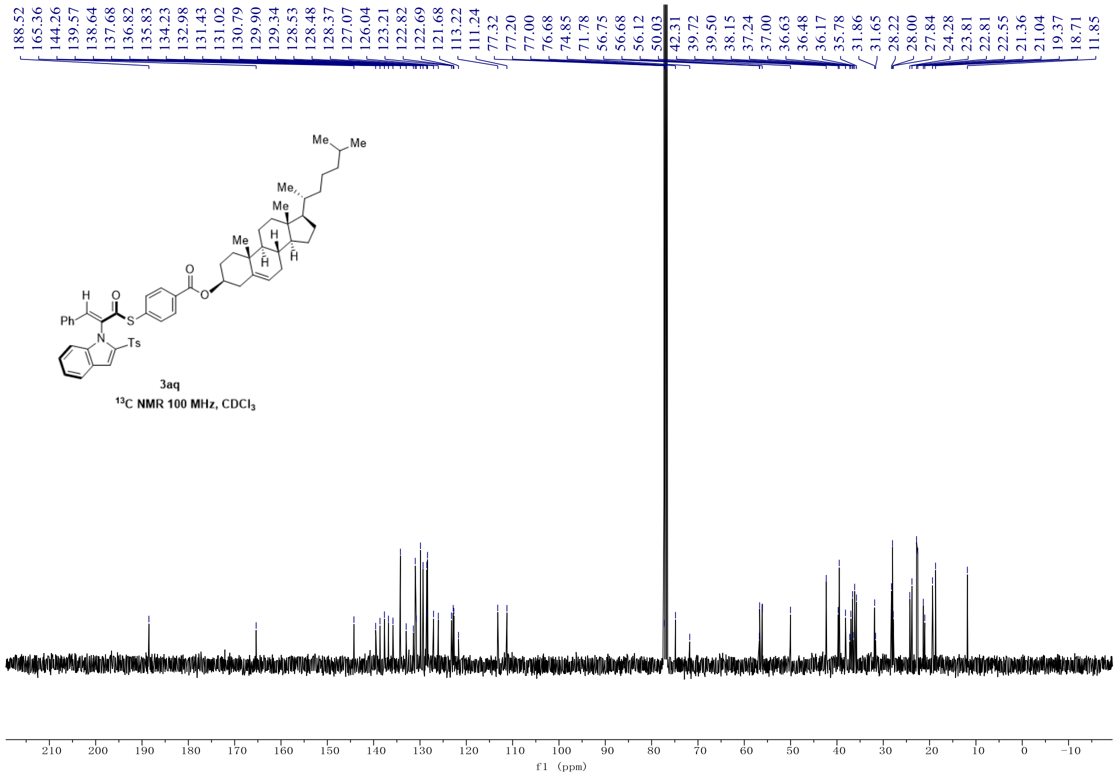
3aq
13C NMR 100 MHz, CDCl3
f1 (ppm)

HPLC conditions: Chiralpak IA-H, 30% ^{i}PrOH/Hx eluent, 0.7 mL/min, 250 nm

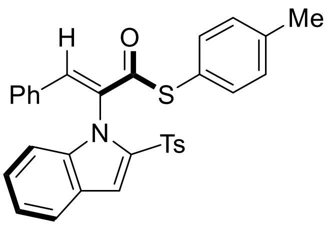

3a

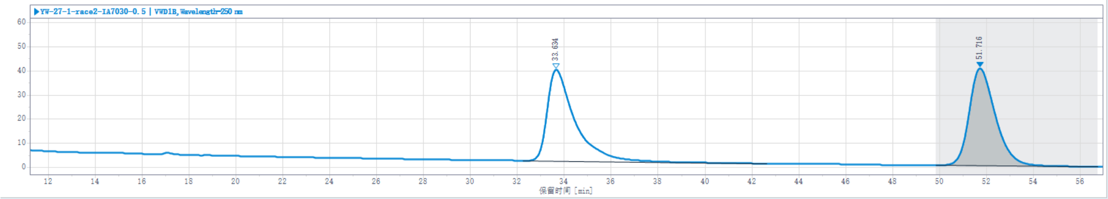

进样结果

峰 汇总

#	名称	RT (min)	峰面积 (mAU·s)	峰面积 %
1		33.634	1384.486	49.957
2		51.716	1386.869	50.043

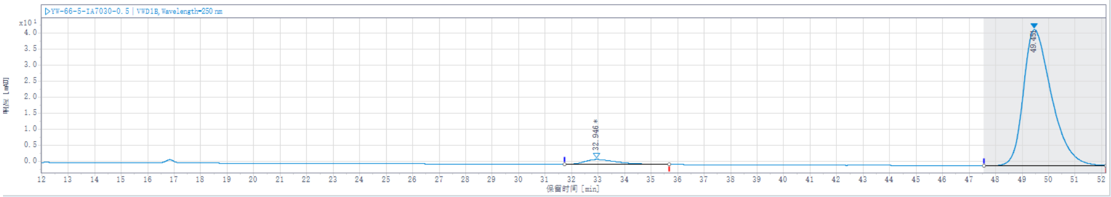

进样结果

峰 汇总

#	名称	RT (min)	峰面积 (mAU·s)	峰面积 %
1		32.946	130.547	3.996
2		49.451	3136.352	96.004

HPLC conditions: Chiralpak IA-H, 30% ^{i}PrOH/Hx eluent, 0.7 mL/min, 250 nm

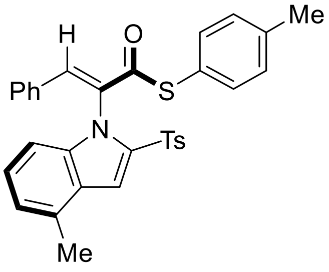

3b

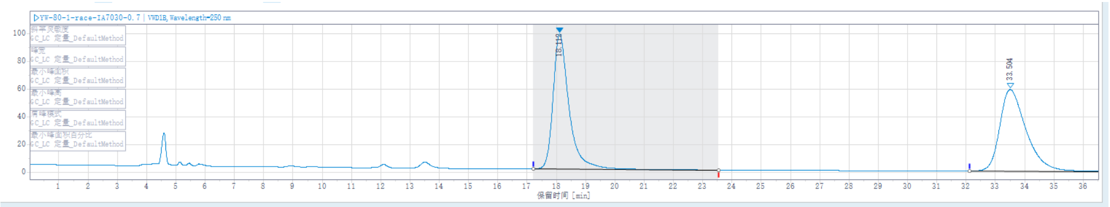

进样结果

峰 汇总

#	名称	RT (min)	峰面积 (mAU·s)	峰面积 %
1		18.112	1343.752	50.037
2		33.504	1341.779	49.963

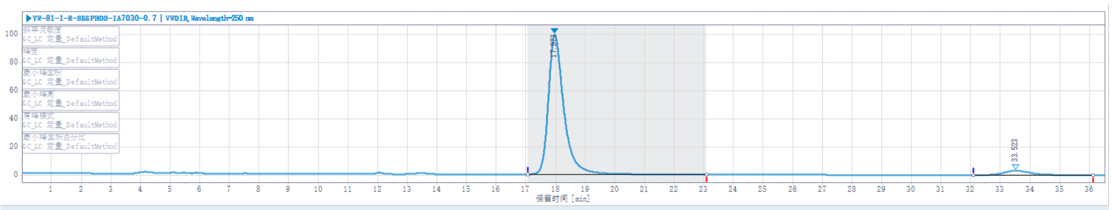

[illegible]样结果

汇总

#	名称	RT (min)	峰面积 (mAU·s)	峰面积 %
1		17.973	2162.910	94.803
2		33.523	118.568	5.197

HPLC conditions: Chiralpak IA-H, 30% ^{i}PrOH/Hx eluent, 0.7 mL/min, 250 nm

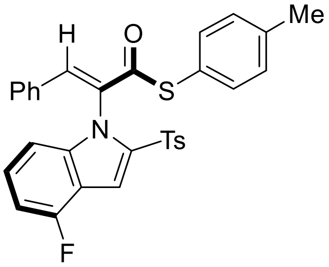

3c

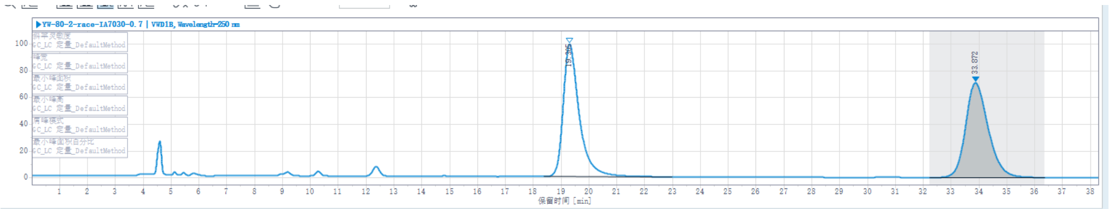

进样结果

峰 汇总

#	名称	RT (min)	峰面积 (mAU·s)	峰面积 %
1		19.305	1332.143	49.903
2		33.872	1337.340	50.097

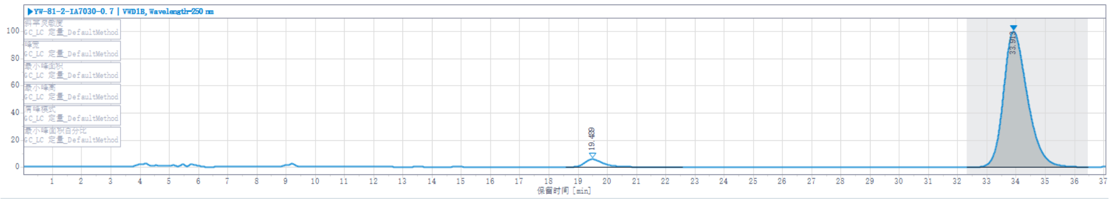

样结果

峰 汇总

#	名称	RT (min)	峰面积 (mAU·s)	峰面积 %
1		19.489	96.029	4.451
2		33.913	2061.567	95.549

HPLC conditions: Chiralpak IA-H, 30% ^{i}PrOH/Hx eluent, 0.7 mL/min, 250 nm

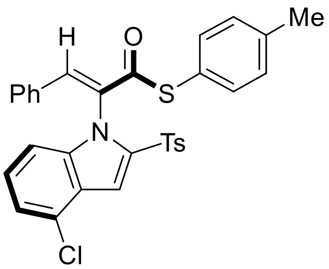

3d

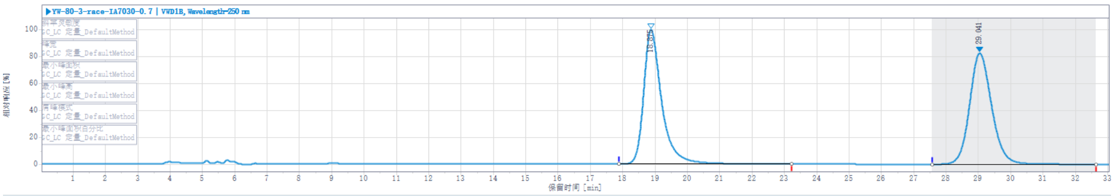

进样结果

峰 汇总

#	名称	RT (min)	峰面积 (mAU·s)	峰面积 %
1		18.875	1252.709	49.720
2		29.041	1266.806	50.280

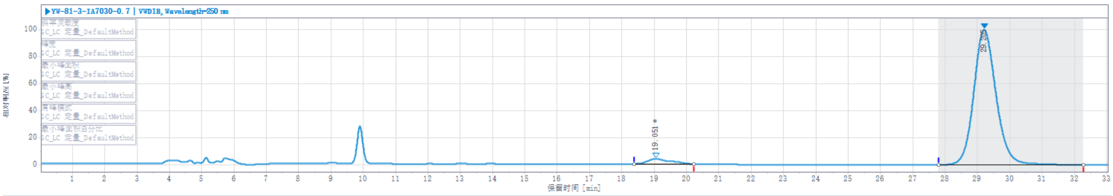

进样结果

峰 汇总

#	名称	RT (min)	峰面积 (mAU·s)	峰面积 %
1		19.051	30.956	3.725
2		29.205	799.961	96.275

HPLC conditions: Chiralpak IA-H, 30% ^{i}PrOH/Hx eluent, 0.7 mL/min, 250 nm

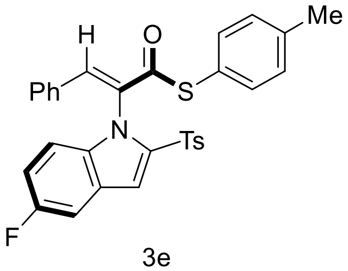

3e

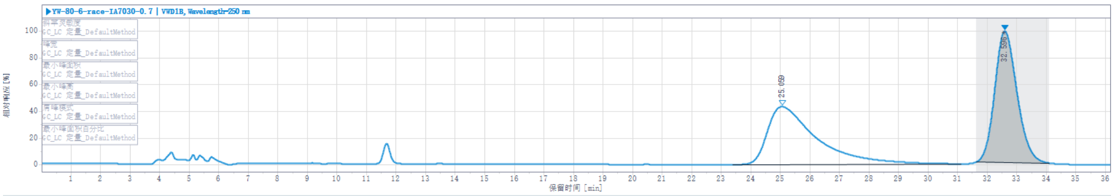

进样结果

峰 汇总

#	名称	RT (min)	峰面积 (mAU·s)	峰面积 %
1		25.059	713.048	49.440
2		32.596	729.200	50.560

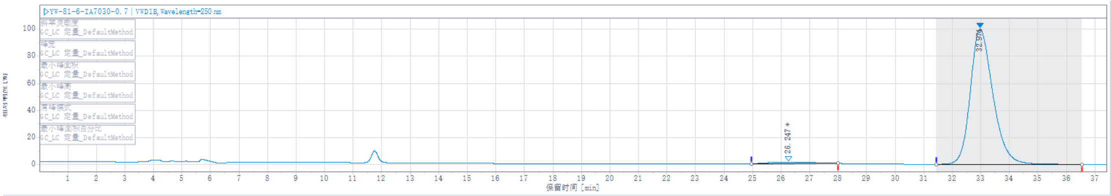

进样结果

峰 汇总

#	名称	RT (min)	峰面积 (mAU·s)	峰面积 %
1		26.247	42.143	2.194
2		32.974	1878.712	97.806

HPLC conditions: Chiralpak IA-H, 30% ^{i}PrOH/Hx eluent, 0.7 mL/min, 250 nm

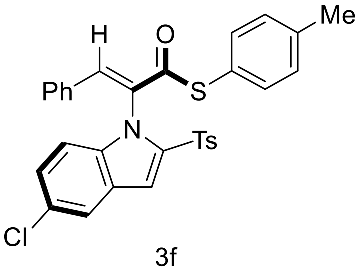

3f

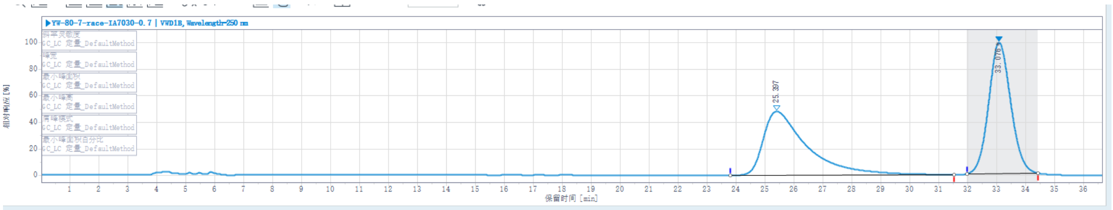

进样结果

峰 汇总

#	名称	RT (min)	峰面积 (mAU·s)	峰面积 %
1		25.397	1066.777	49.423
2		33.076	1091.679	50.577

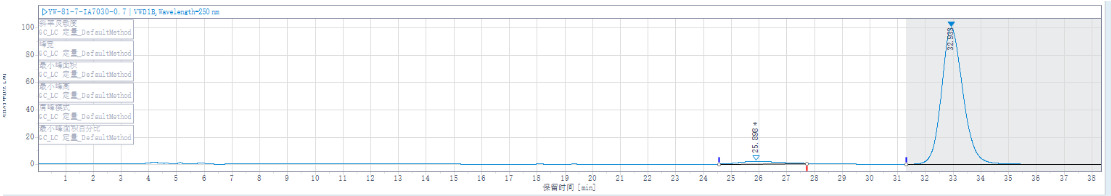

进样结果

峰 汇总

#	名称	RT (min)	峰面积 (mAU·s)	峰面积 %
1		25.898	84.522	3.109
2		32.933	2634.327	96.891

HPLC conditions: Chiralpak IA-H, 30% ^{i}PrOH/Hx eluent, 0.7 mL/min, 250 nm

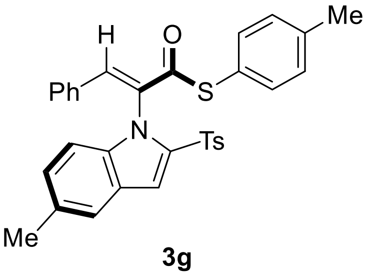

3g

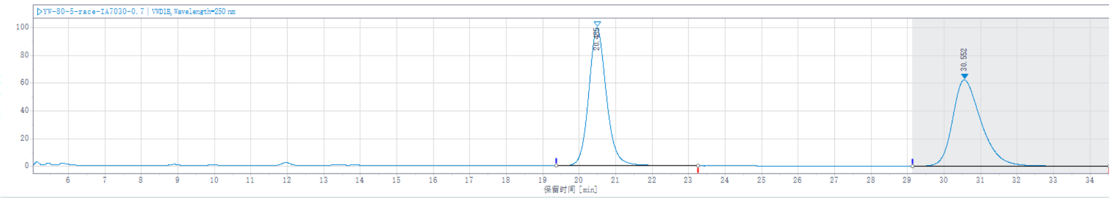

进样结果

峰 汇总

#	名称	RT (min)	峰面积 (mAU·s)	峰面积 %
1		20.485	1015.167	49.623
2		30.552	1030.573	50.377

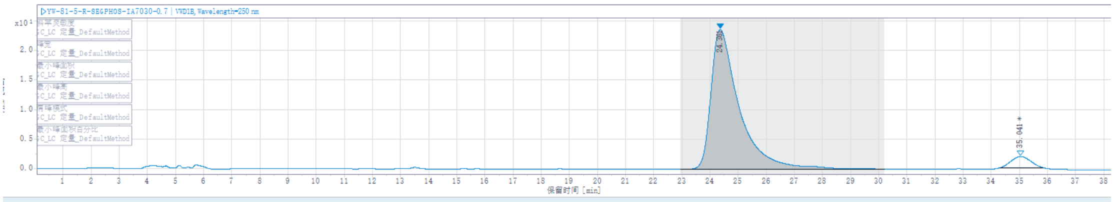

进样结果

峰 汇总

#	名称	RT (min)	峰面积 (mAU·s)	峰面积 %
1		24.361	1659.379	94.760
2		35.041	91.767	5.240

HPLC conditions: Chiralpak IA-H, 30% ^{i}PrOH/Hx eluent, 0.7 mL/min, 250 nm

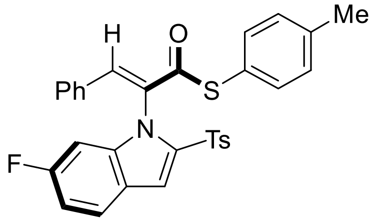

3h

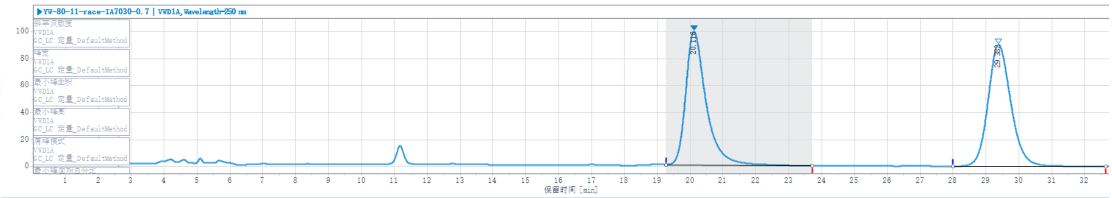

进样结果

峰 汇总

#	名称	RT (min)	峰面积 (mAU·s)	峰面积 %
1		20.116	697.492	49.337
2		29.382	716.251	50.663

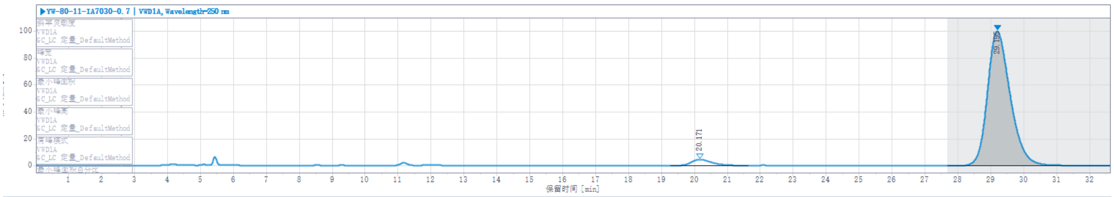

进样结果

峰 汇总

#	名称	RT (min)	峰面积 (mAU·s)	峰面积 %
1		20.171	148.132	3.982
2		29.195	3571.923	96.018

HPLC conditions: Chiralpak IA-H, 30% ^{i}PrOH/Hx eluent, 0.7 mL/min, 250 nm

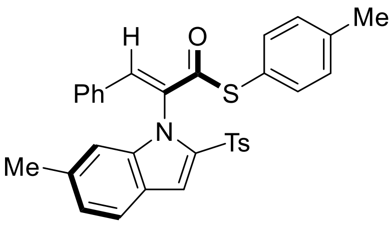

3i

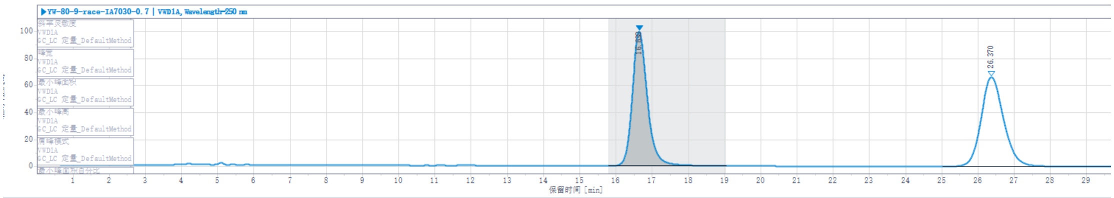

进样结果

峰 汇总

#	名称	RT (min)	峰面积 (mAU·s)	峰面积 %
1		16.638	1565.778	49.970
2		26.370	1567.659	50.030

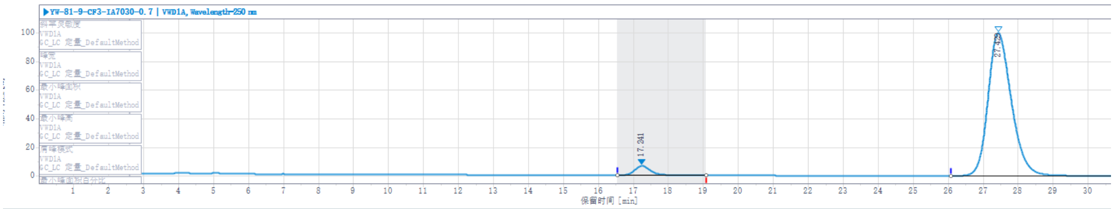

进样结果

峰 汇总

#	名称	RT (min)	峰面积 (mAU·s)	峰面积 %
1		17.241	151.889	4.220
2		27.429	3447.551	95.780

HPLC conditions: Chiralpak IA-H, 30% ^{i}PrOH/Hx eluent, 0.7 mL/min, 250 nm

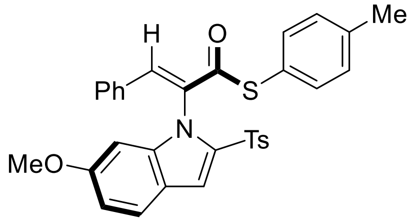

3j

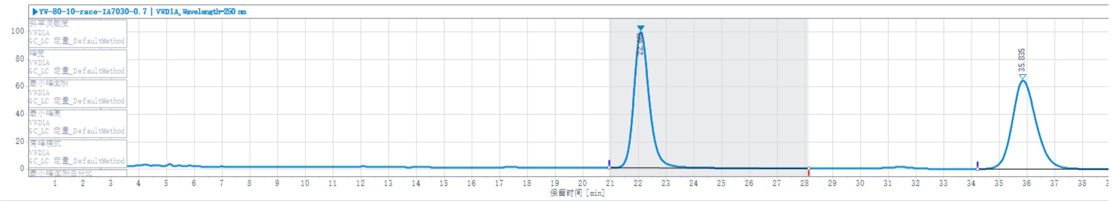

进样结果

峰 汇总

#	名称	RT (min)	峰面积 (mAU·s)	峰面积 %
1		22.061	1317.784	49.904
2		35.835	1322.857	50.096

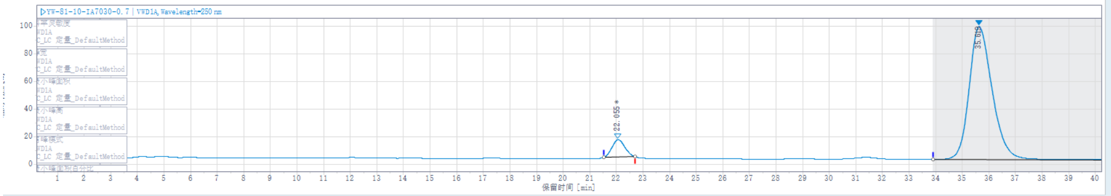

进样结果

峰 汇总

#	名称	RT (min)	峰面积 (mAU·s)	峰面积 %
1		22.055	197.792	6.720
2		35.619	2745.678	93.280

HPLC conditions: Chiralpak IA-H, 30% ^{i}PrOH/Hx eluent, 0.7 mL/min, 250 nm

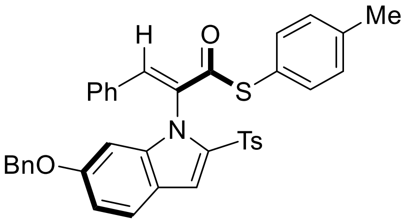

3**k**

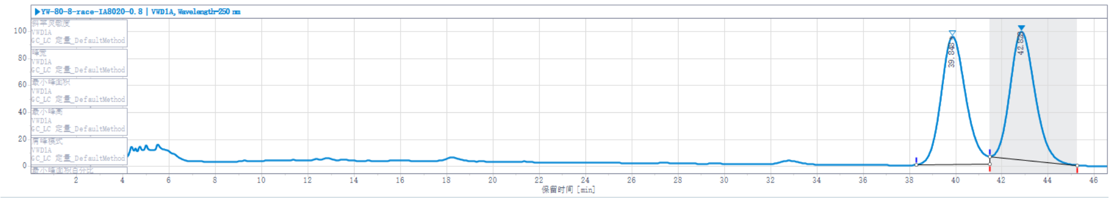

进样结果

峰 汇总

#	名称	RT (min)	峰面积 (mAU·s)	峰面积 %
1		39.848	645.113	50.018
2		42.828	644.655	49.982

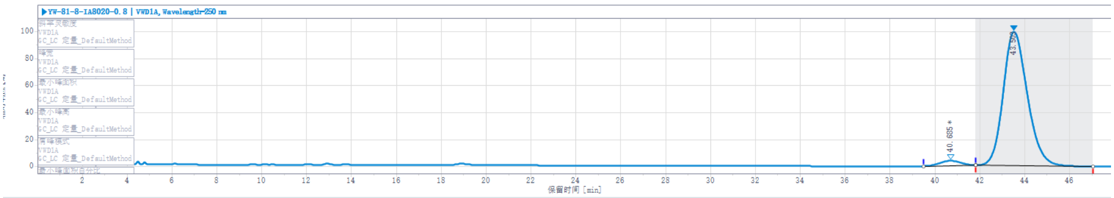

进样结果

峰 汇总

#	名称	RT (min)	峰面积 (mAU·s)	峰面积 %
1		40.685	61.946	3.043
2		43.503	1973.500	96.957

HPLC conditions: Chiralpak IA-H, 30% ^{i}PrOH/Hx eluent, 0.7 mL/min, 250 nm

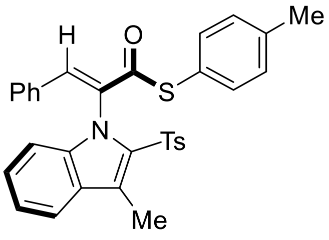

3l

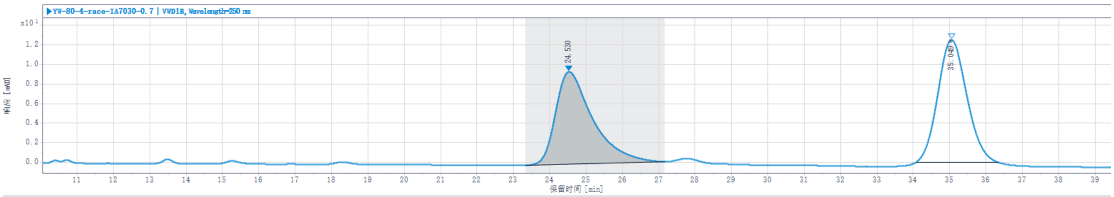

进样结果

峰 汇总

#	名称	RT (min)	峰面积 (mAU·s)	峰面积 %
1		24.530	671.156	49.246
2		35.049	691.703	50.754

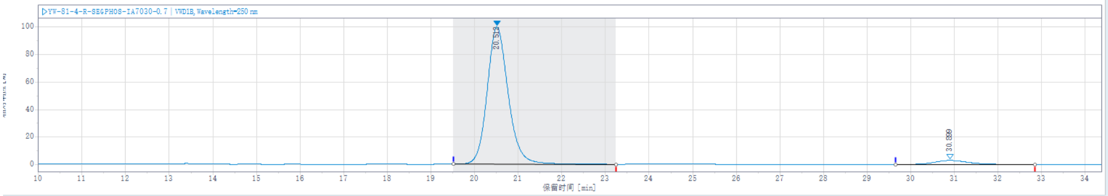

进样结果

峰 汇总

#	名称	RT (min)	峰面积 (mAU·s)	峰面积 %
1		20.512	2533.600	95.420
2		30.899	121.618	4.580

HPLC conditions: Chiralpak IA-H, 30% ^{i}PrOH/Hx eluent, 0.7 mL/min, 250 nm

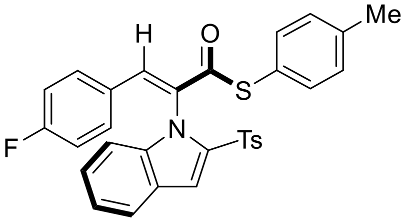

3m

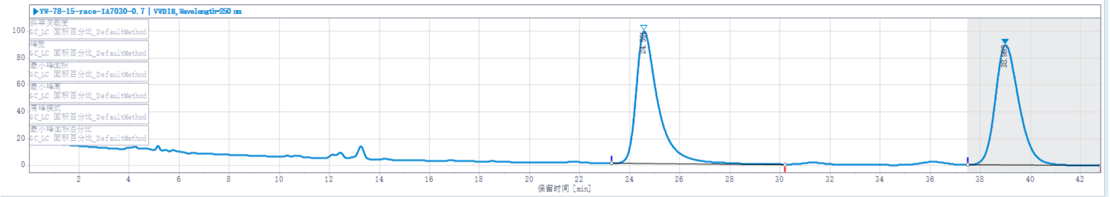

进样结果

峰 汇总

#	名称	RT (min)	峰面积 (mAU·s)	峰面积 %
1		24.561	833.861	50.522
2		38.993	816.638	49.478

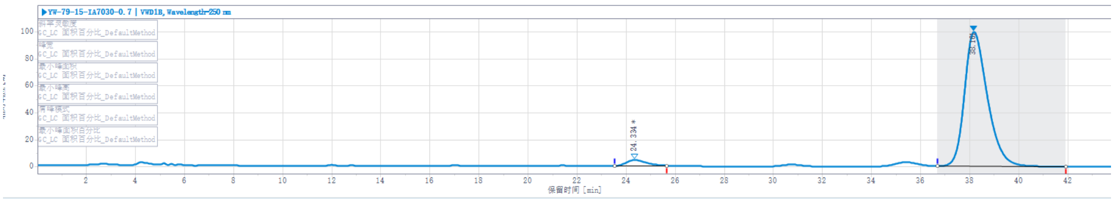

进样结果

峰 汇总

#	名称	RT (min)	峰面积 (mAU·s)	峰面积 %
1		24.334	70.441	3.607
2		38.161	1882.452	96.393

HPLC conditions: Chiralpak IA-H, 30% ^{i}PrOH/Hx eluent, 0.7 mL/min, 250 nm

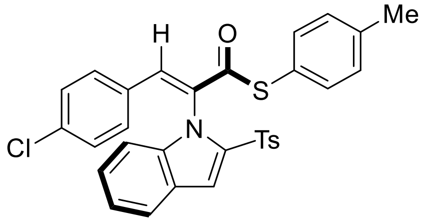

3n

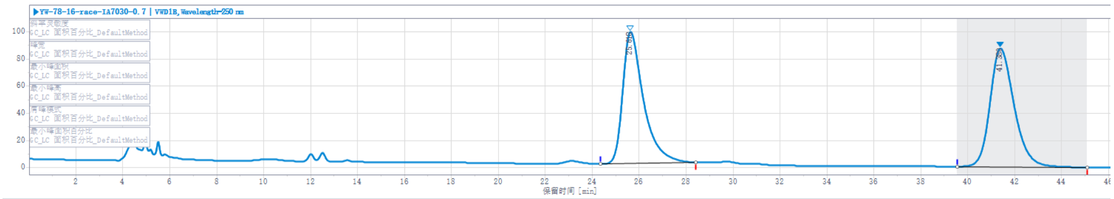

进样结果

峰 汇总

#	名称	RT (min)	峰面积 (mAU·s)	峰面积 %
1		25.618	586.771	48.958
2		41.380	611.752	51.042

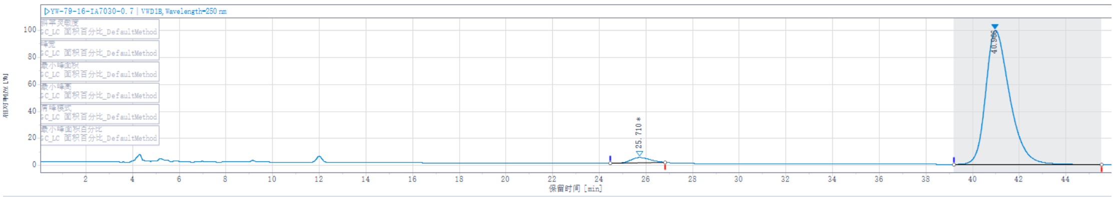

进样结果

峰 汇总

#	名称	RT (min)	峰面积 (mAU·s)	峰面积 %
1		25.710	58.000	3.169
2		40.966	1772.355	96.831

HPLC conditions: Chiralpak IA-H, 30% ^{i}PrOH/Hx eluent, 0.7 mL/min, 250 nm

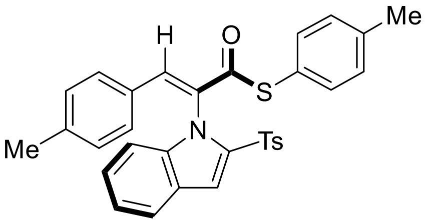

3o

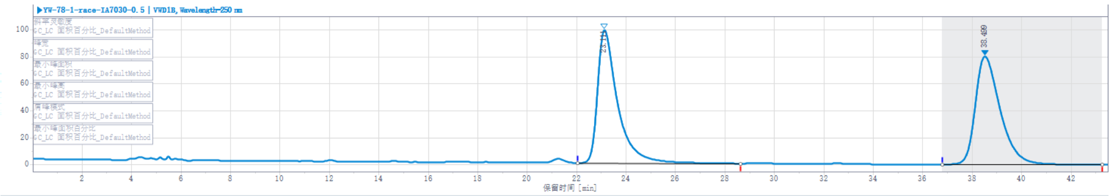

进样结果

峰 汇总

#	名称	RT (min)	峰面积 (mAU·s)	峰面积 %
1		23.111	1457.843	49.856
2		38.499	1466.255	50.144

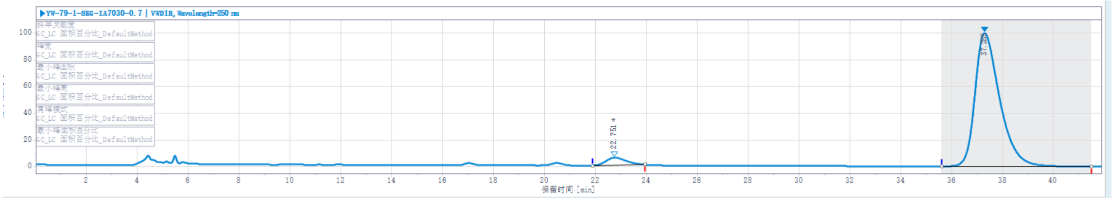

进样结果

峰 汇总

#	名称	RT (min)	峰面积 (mAU·s)	峰面积 %
1		22.751	74.994	4.340
2		37.285	1653.025	95.660

HPLC conditions: Chiralpak IA-H, 30% ^{i}PrOH/Hx eluent, 0.7 mL/min, 250 nm

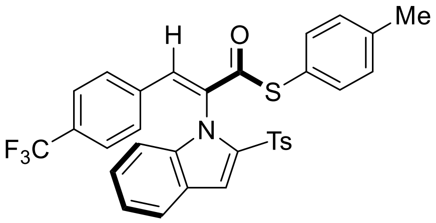

3p

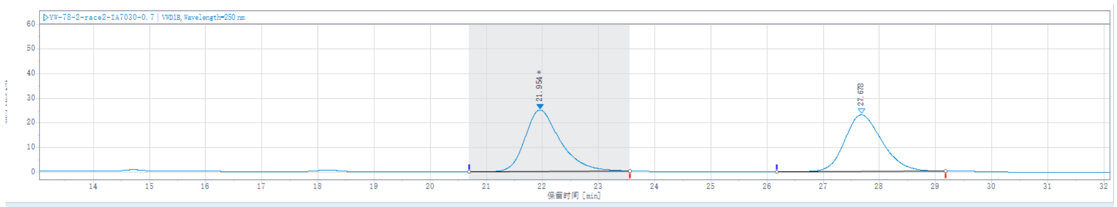

进样结果

峰 汇总

#	名称	RT (min)	峰面积 (mAU·s)	峰面积 %
1		21.954	488.805	50.649
2		27.678	476.273	49.351

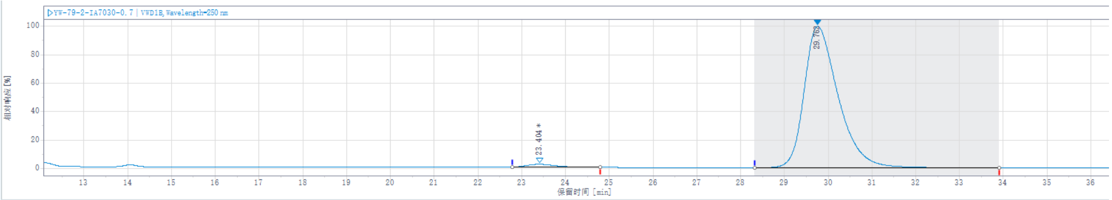

进样结果

峰 汇总

#	名称	RT (min)	峰面积 (mAU·s)	峰面积 %
1		23.404	30.840	1.707
2		29.762	1775.539	98.293

HPLC conditions: Chiralpak IA-H, 30% ^{i}PrOH/Hx eluent, 0.7 mL/min, 250 nm

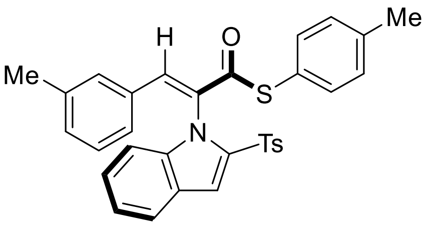

3q

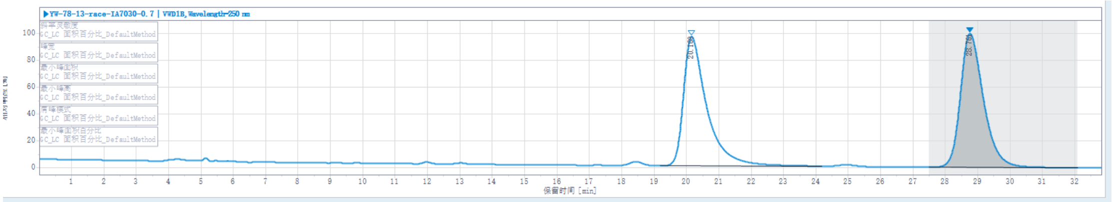

进样结果

峰 汇总

#	名称	RT (min)	峰面积 (mAU·s)	峰面积 %
1		20.160	1015.574	49.434
2		28.761	1038.825	50.566

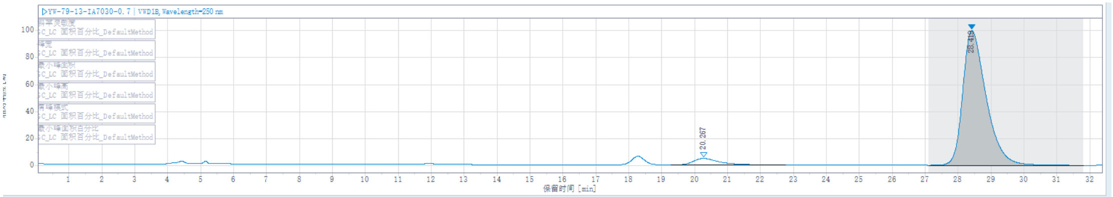

进样结果

峰 汇总

#	名称	RT (min)	峰面积 (mAU·s)	峰面积 %
1		20.267	142.023	5.050
2		28.419	2670.088	94.950

HPLC conditions: Chiralpak IA-H, 30% ^{i}PrOH/Hx eluent, 0.7 mL/min, 250 nm

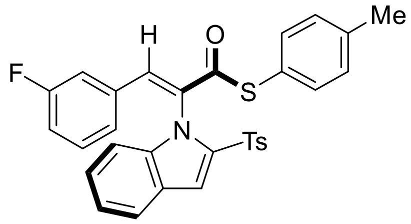

3r

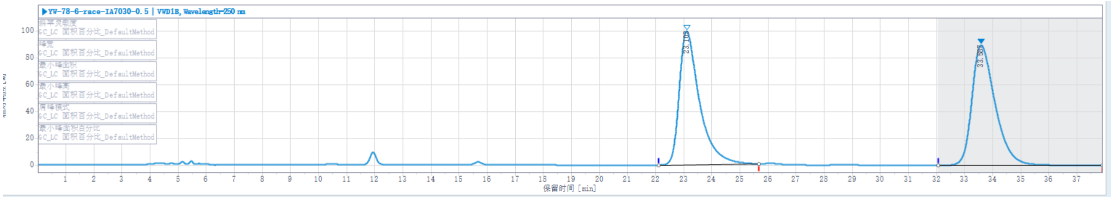

进样结果

峰 汇总

#	名称	RT (min)	峰面积 (mAU·s)	峰面积 %
1		23.103	1679.346	49.040
2		33.587	1745.103	50.960

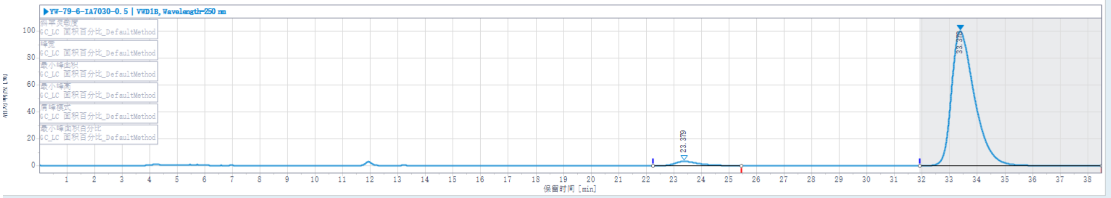

进样结果

峰 汇总

#	名称	RT (min)	峰面积 (mAU·s)	峰面积 %
1		23.379	102.522	3.228
2		33.378	3073.733	96.772

HPLC conditions: Chiralpak IA-H, 30% ^{i}PrOH/Hx eluent, 0.7 mL/min, 250 nm

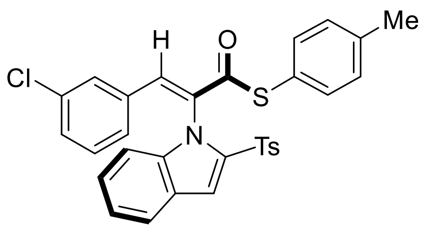

3s

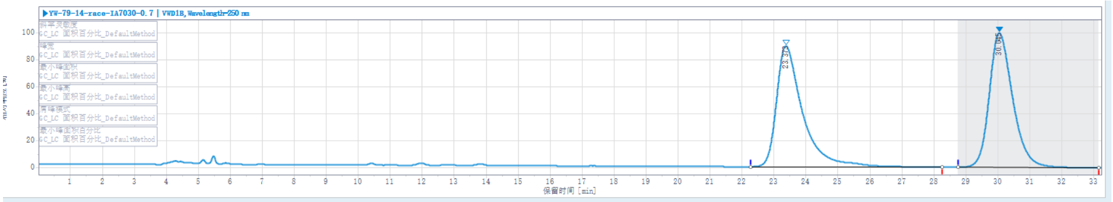

进样结果

峰 汇总

#	名称	RT (min)	峰面积 (mAU·s)	峰面积 %
1		23.373	721.814	50.171
2		30.045	716.885	49.829

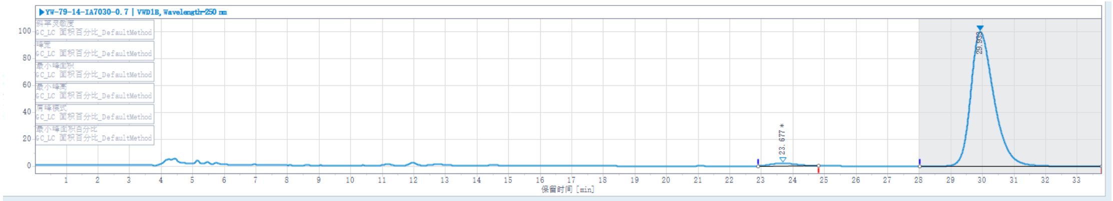

进样结果

峰 汇总

#	名称	RT (min)	峰面积 (mAU·s)	峰面积 %
1		23.677	28.725	2.099
2		29.933	1339.877	97.901

HPLC conditions: Chiralpak IA-H, 30% ^{i}PrOH/Hx eluent, 0.7 mL/min, 250 nm

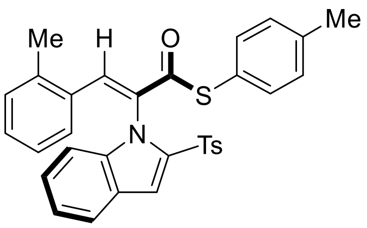

3t

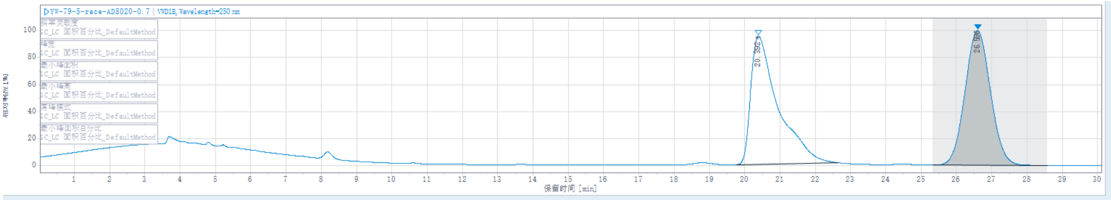

进样结果

峰 汇总

#	名称	RT (min)	峰面积 (mAU·s)	峰面积 %
1		20.392	1376.880	50.631
2		26.596	1342.566	49.369

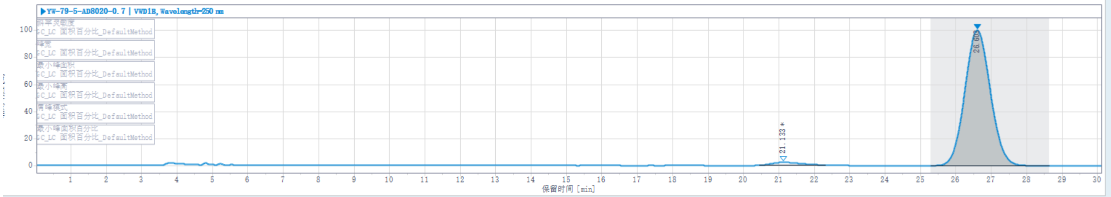

进样结果

峰 汇总

#	名称	RT (min)	峰面积 (mAU·s)	峰面积 %
1		21.133	64.050	2.298
2		26.603	2723.171	97.702

HPLC conditions: Chiralpak IA-H, 30% ^{i}PrOH/Hx eluent, 0.7 mL/min, 250 nm

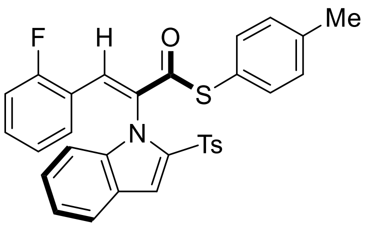

3u

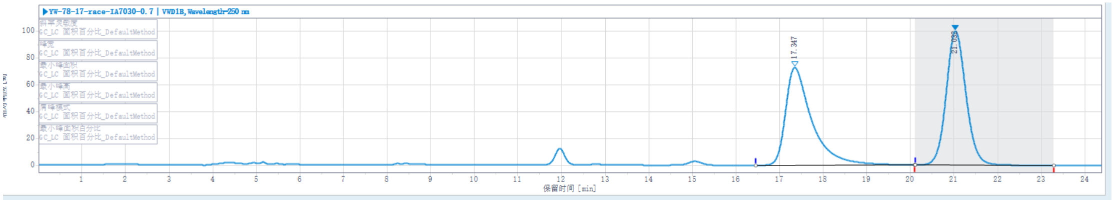

进样结果

峰 汇总

#	名称	RT (min)	峰面积 (mAU·s)	峰面积 %
1		17.347	870.385	49.245
2		21.032	897.068	50.755

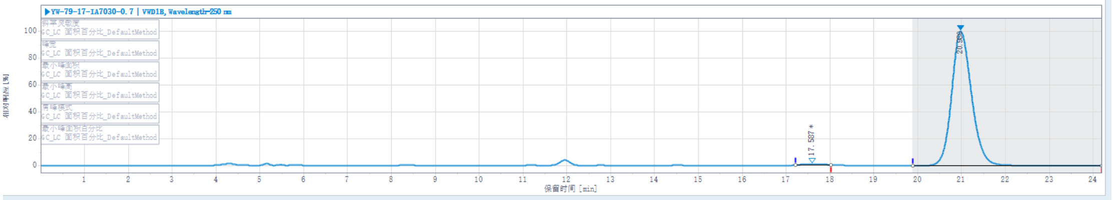

进样结果

峰 汇总

#	名称	RT (min)	峰面积 (mAU·s)	峰面积 %
1		17.587	15.522	0.670
2		20.980	2299.570	99.330

HPLC conditions: Chiralpak IA-H, 30% ^{i}PrOH/Hx eluent, 0.7 mL/min, 250 nm

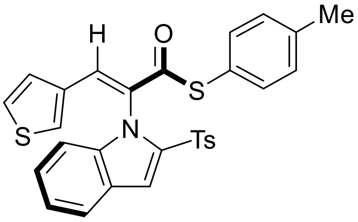

3v

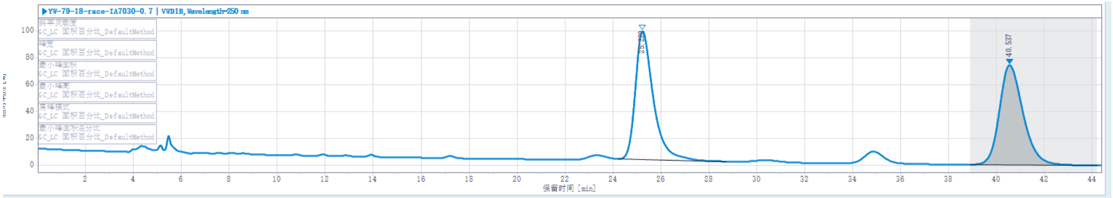

进样结果

峰 汇总

#	名称	RT (min)	峰面积 (mAU·s)	峰面积 %
1		25.222	689.948	49.484
2		40.537	704.332	50.516

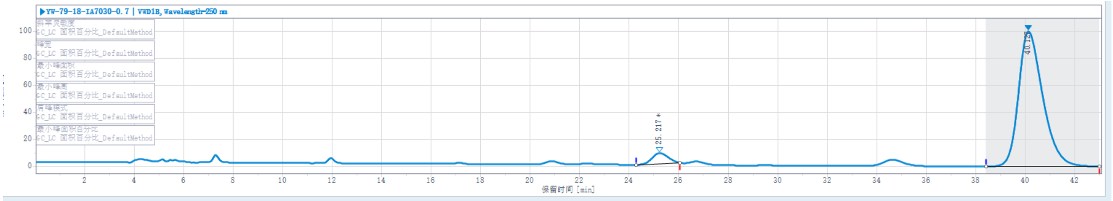

进样结果

峰 汇总

#	名称	RT (min)	峰面积 (mAU·s)	峰面积 %
1		25.217	79.412	4.962
2		40.127	1520.983	95.038

HPLC conditions: Chiralpak IA-H, 30% ^{i}PrOH/Hx eluent, 0.7 mL/min, 250 nm

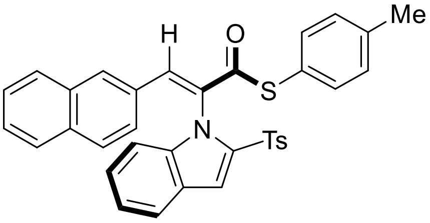

3w

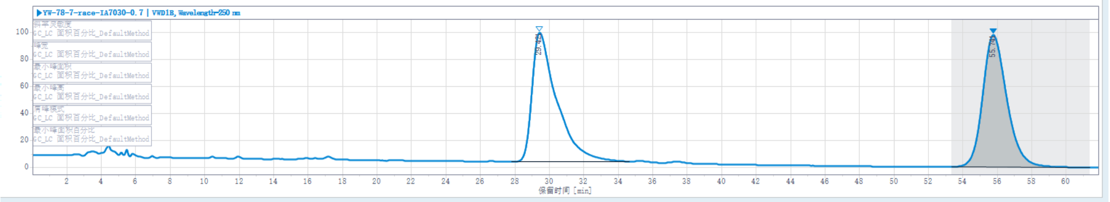

进样结果

峰 汇总

#	名称	RT (min)	峰面积 (mAU·s)	峰面积 %
1		29.421	1150.665	49.872
2		55.741	1156.560	50.128

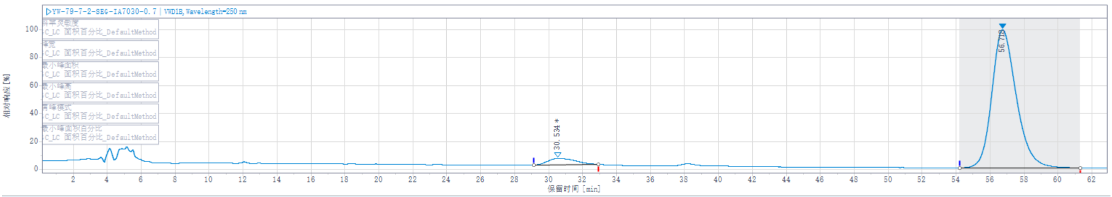

进样结果

峰 汇总

#	名称	RT (min)	峰面积 (mAU·s)	峰面积 %
1		30.534	62.307	4.991
2		56.718	1186.178	95.009

HPLC conditions: Chiralpak IA-H, 30% ^{i}PrOH/Hx eluent, 0.7 mL/min, 250 nm

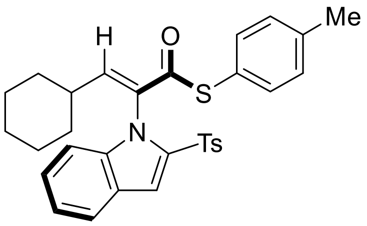

3x

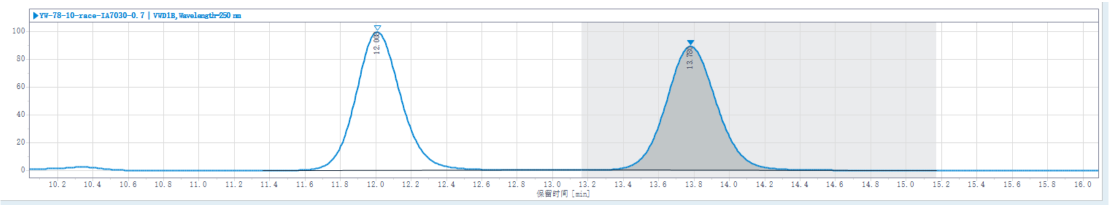

进样结果

峰 汇总

#	名称	RT (min)	峰面积 (mAU·s)	峰面积 %
1		12.008	2148.143	49.907
2		13.780	2156.156	50.093

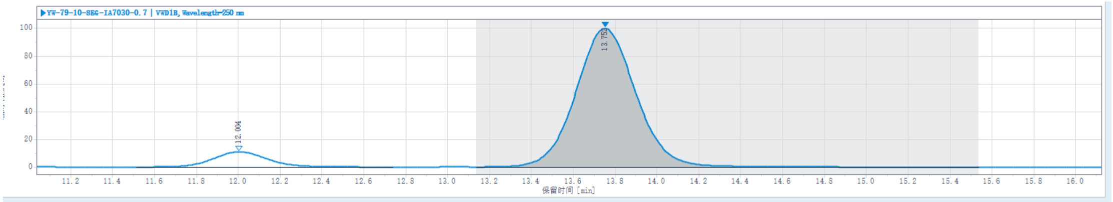

进样结果

峰 汇总

#	名称	RT (min)	峰面积 (mAU·s)	峰面积 %
1		12.004	116.846	9.047
2		13.752	1174.771	90.953

HPLC conditions: Chiralpak IA-H, 30% ^{i}PrOH/Hx eluent, 0.7 mL/min, 250 nm

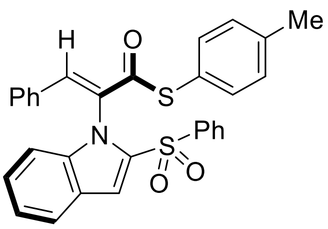

3y

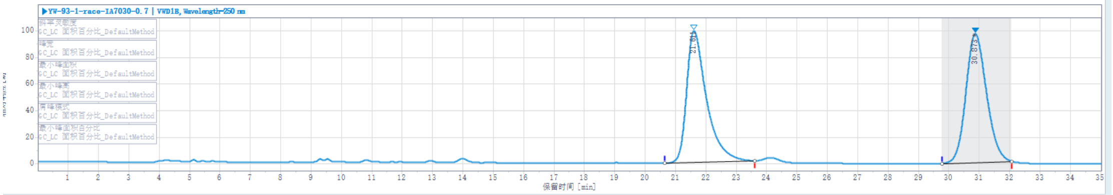

进样结果

峰 汇总

#	名称	RT (min)	峰面积 (mAU·s)	峰面积 %
1		21.611	1012.577	49.718
2		30.873	1024.063	50.282

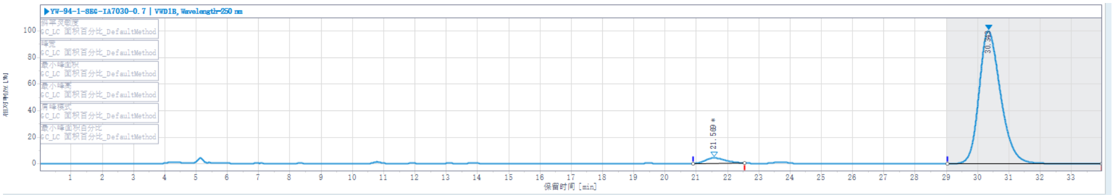

进样结果

峰 汇总

#	名称	RT (min)	峰面积 (mAU·s)	峰面积 %
1		21.569	92.129	3.588
2		30.343	2475.716	96.412

HPLC conditions: Chiralpak IA-H, 30% ^{i}PrOH/Hx eluent, 0.7 mL/min, 250 nm

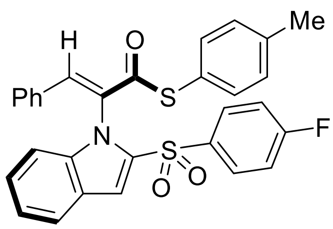

3z

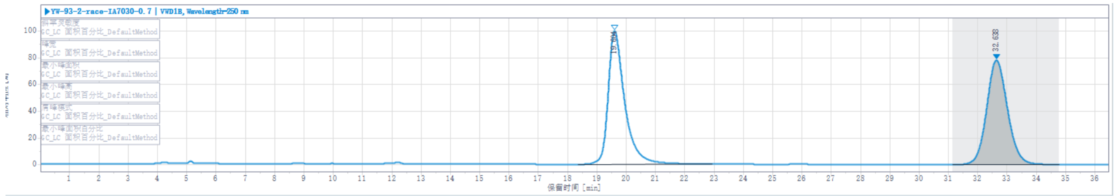

进样结果

峰 汇总

#	名称	RT (min)	峰面积 (mAU·s)	峰面积 %
1		19.604	1254.074	50.549
2		32.638	1226.825	49.451

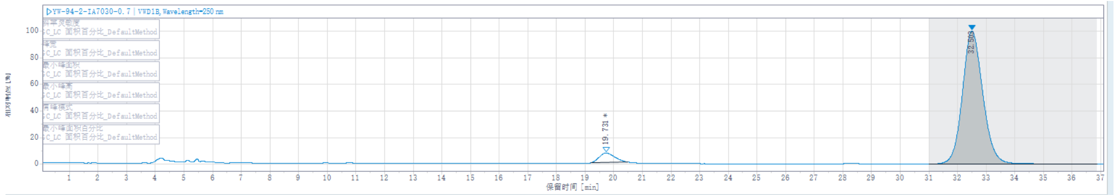

进样结果

峰 汇总

#	名称	RT (min)	峰面积 (mAU·s)	峰面积 %
1		19.731	79.712	5.059
2		32.503	1495.821	94.941

HPLC conditions: Chiralpak IA-H, 30% ^{i}PrOH/Hx eluent, 0.7 mL/min, 250 nm

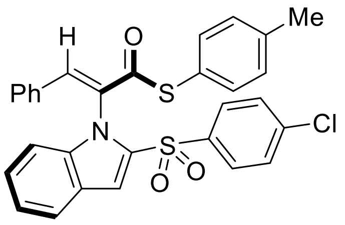

3aa

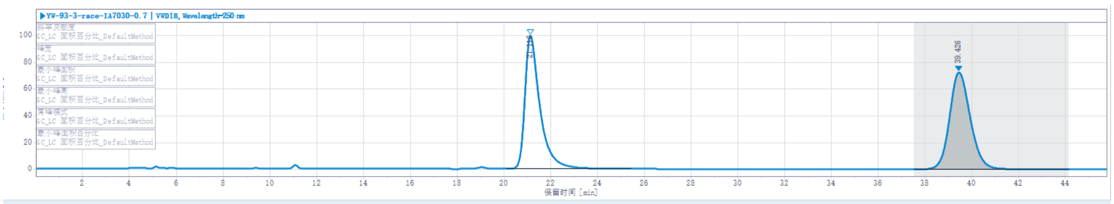

进样结果

峰 汇总

#	名称	RT (min)	峰面积 (mAU·s)	峰面积 %
1		21.116	2260.589	49.870
2		39.426	2272.389	50.130

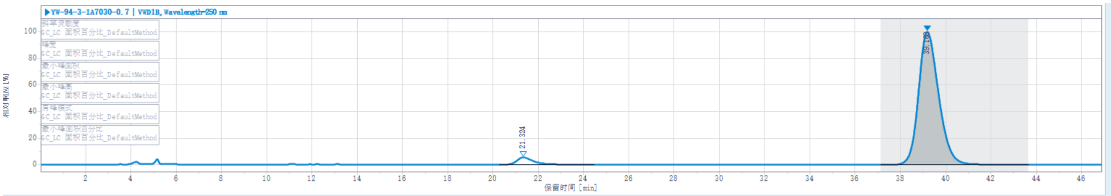

进样结果

峰 汇总

#	名称	RT (min)	峰面积 (mAU·s)	峰面积 %
1		21.324	224.410	4.440
2		39.168	4830.025	95.560

HPLC conditions: Chiralpak IA-H, 30% ^{i}PrOH/Hx eluent, 0.7 mL/min, 250 nm

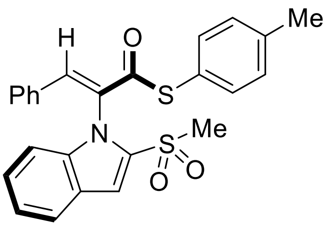

3ab

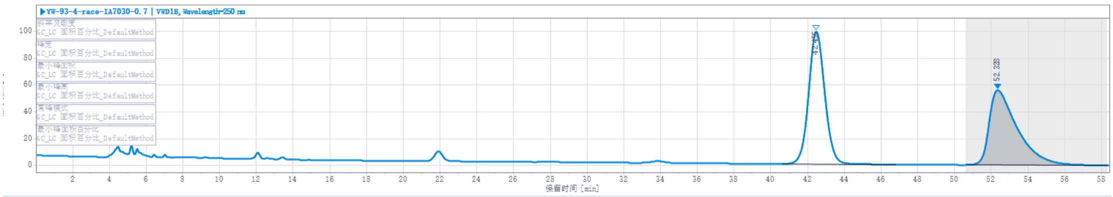

进样结果

峰 汇总

#	名称	RT (min)	峰面积 (mAU·s)	峰面积 %
1		42.455	800.615	50.223
2		52.328	793.521	49.777

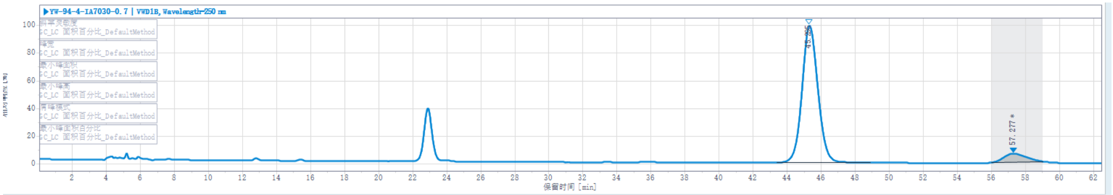

进样结果

峰 汇总

#	名称	RT (min)	峰面积 (mAU·s)	峰面积 %
1		45.295	840.795	92.001
2		57.277	73.101	7.999

HPLC conditions: Chiralpak IA-H, 30% ^{i}PrOH/Hx eluent, 0.7 mL/min, 250 nm

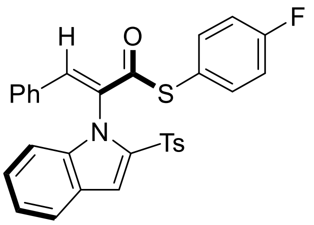

3ac

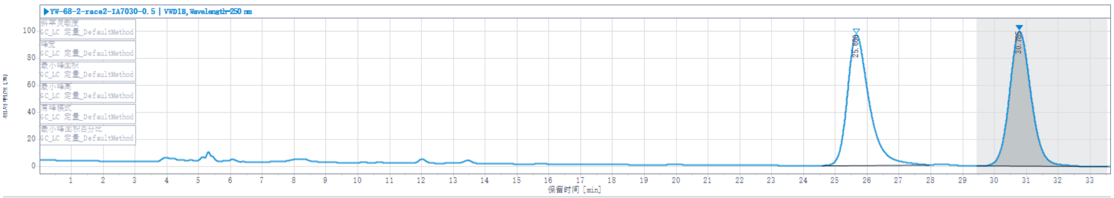

进样结果

峰 汇总

#	名称	RT (min)	峰面积 (mAU·s)	峰面积 %
1		25.660	808.060	49.289
2		30.785	831.374	50.711

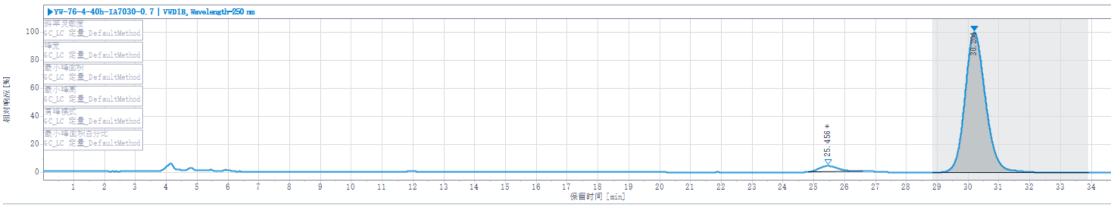

进样结果

峰 汇总

#	名称	RT (min)	峰面积 (mAU·s)	峰面积 %
1		25.456	51.711	3.328
2		30.204	1502.117	96.672

HPLC conditions: Chiralpak IA-H, 30% ^{i}PrOH/Hx eluent, 0.7 mL/min, 250 nm

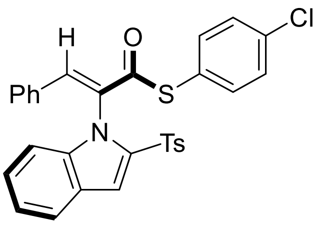

3ad

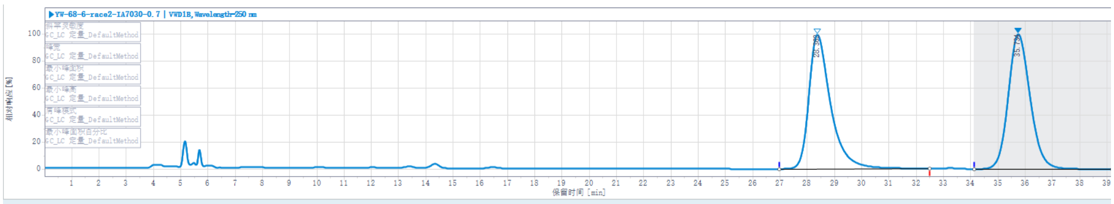

进样结果

峰 汇总

#	名称	RT (min)	峰面积 (mAU·s)	峰面积 %
1		28.362	1153.261	49.836
2		35.734	1160.843	50.164

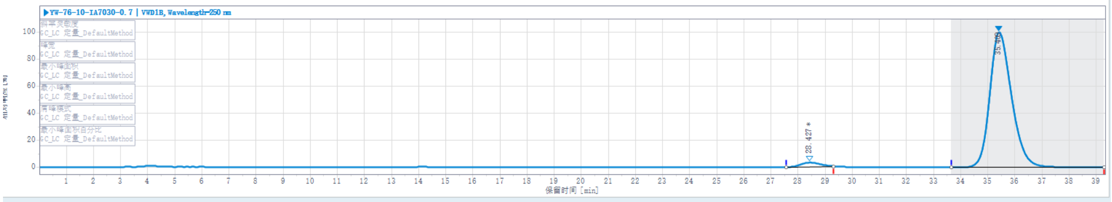

进样结果

峰 汇总

#	名称	RT (min)	峰面积 (mAU·s)	峰面积 %
1		28.427	129.518	2.663
2		35.409	4733.841	97.337

HPLC conditions: Chiralpak IA-H, 30% ^{i}PrOH/Hx eluent, 0.7 mL/min, 250 nm

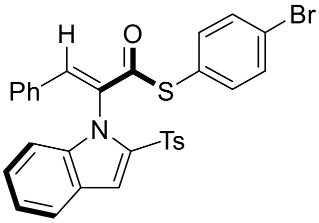

3ae

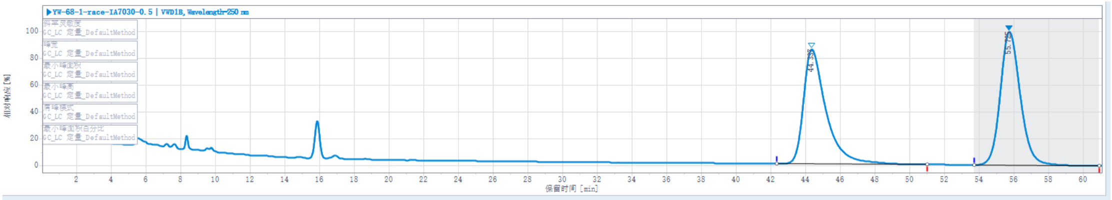

进样结果

峰 汇总

#	名称	RT (min)	峰面积 (mAU·s)	峰面积 %
1		44.337	1069.533	49.210
2		55.725	1103.881	50.790

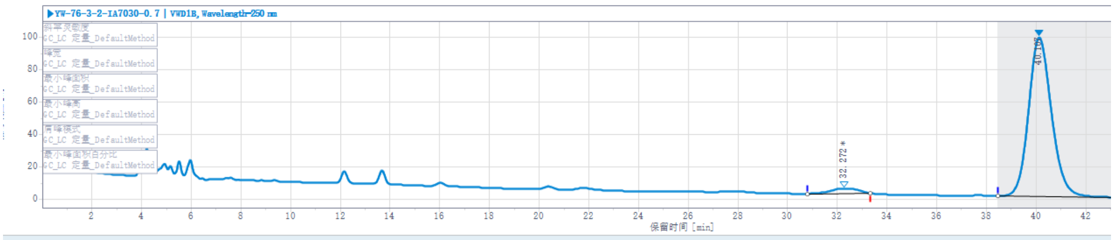

进样结果

峰 汇总

#	名称	RT (min)	峰面积 (mAU·s)	峰面积 %
1		32.272	22.552	3.587
2		40.107	606.156	96.413

HPLC conditions: Chiralpak IA-H, 30% ^{i}PrOH/Hx eluent, 0.7 mL/min, 250 nm

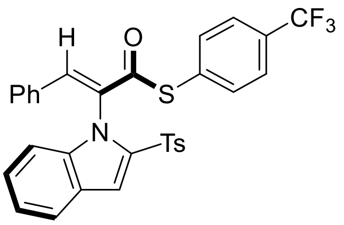

3af

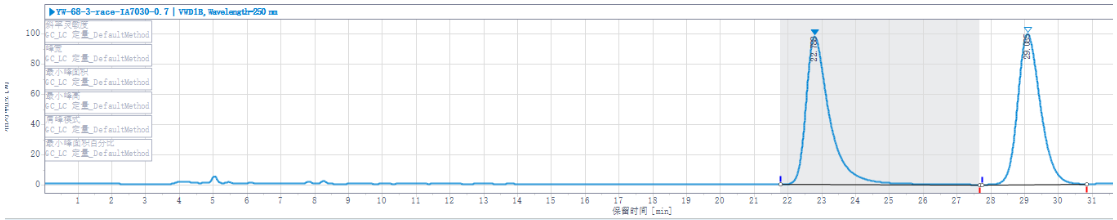

进样结果

峰 汇总

#	名称	RT (min)	峰面积 (mAU·s)	峰面积 %
1		22.788	1780.489	50.636
2		29.085	1735.774	49.364

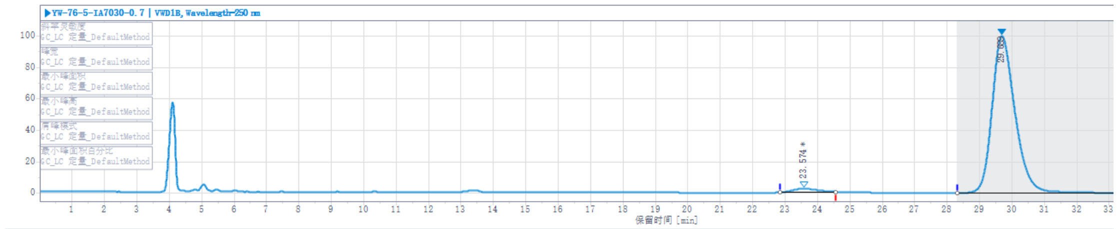

进样结果

峰 汇总

#	名称	RT (min)	峰面积 (mAU·s)	峰面积 %
1		23.574	41.228	2.221
2		29.689	1814.659	97.779

HPLC conditions: Chiralpak IA-H, 30% ^{i}PrOH/Hx eluent, 0.7 mL/min, 250 nm

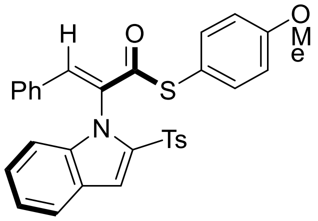

3ag

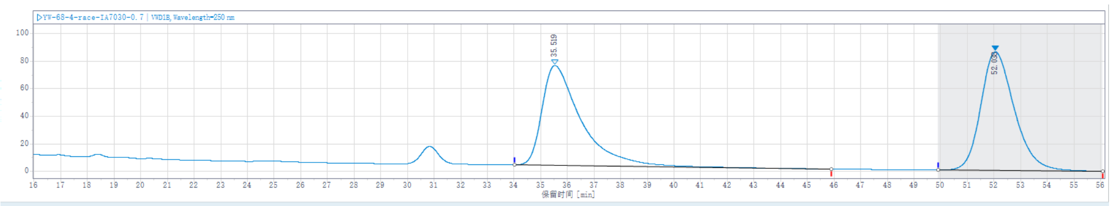

进样结果

峰 汇总

#	名称	RT (min)	峰面积 (mAU·s)	峰面积 %
1		35.519	826.245	49.649
2		52.038	837.940	50.351

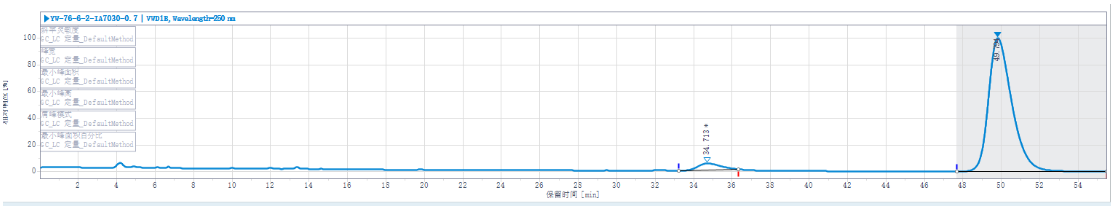

进样结果

峰 汇总

#	名称	RT (min)	峰面积 (mAU·s)	峰面积 %
1		34.713	158.559	4.539
2		49.794	3334.486	95.461

HPLC conditions: Chiralpak IA-H, 30% ^{i}PrOH/Hx eluent, 0.7 mL/min, 250 nm

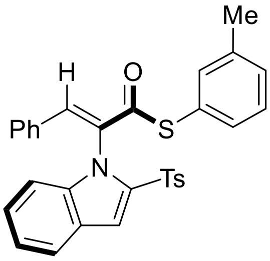

3ah

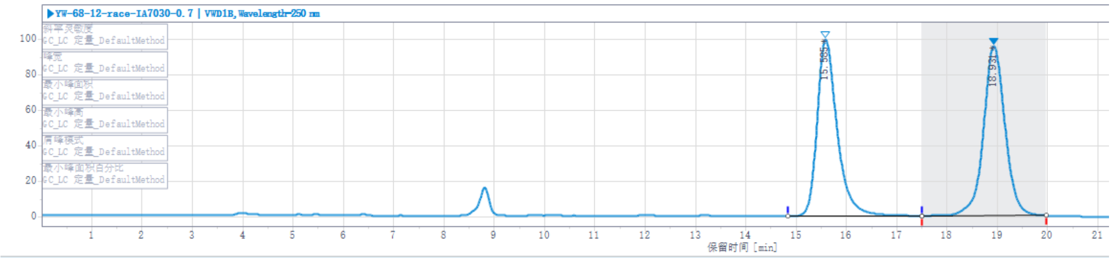

进样结果

峰 汇总

#	名称	RT (min)	峰面积 (mAU·s)	峰面积 %
1		15.585	971.843	49.142
2		18.931	1005.765	50.858

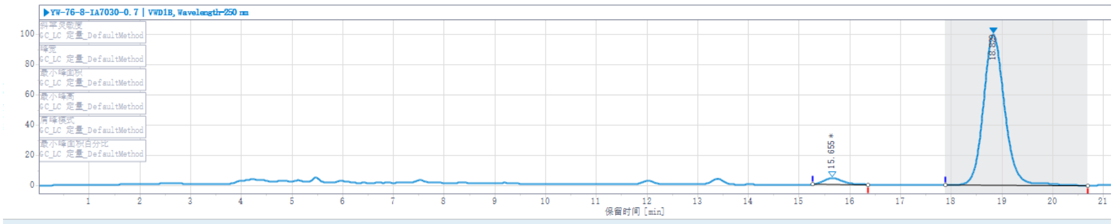

进样结果

峰 汇总

#	名称	RT (min)	峰面积 (mAU·s)	峰面积 %
1		15.655	27.142	3.534
2		18.829	740.802	96.466

HPLC conditions: Chiralpak IA-H, 30% ^{i}PrOH/Hx eluent, 0.7 mL/min, 250 nm

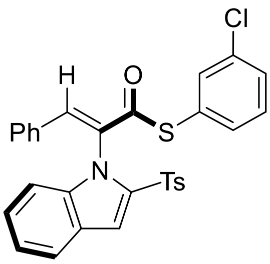

3ai

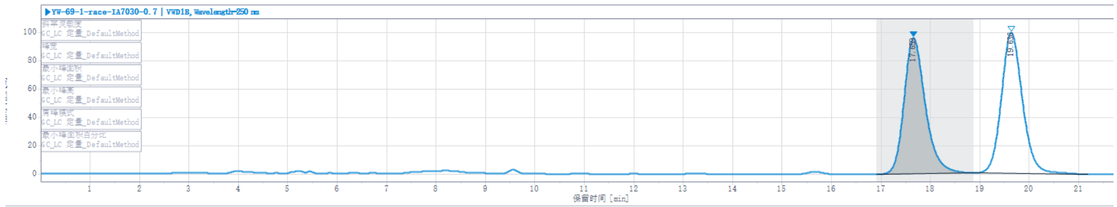

进样结果

峰 汇总

#	名称	RT (min)	峰面积 (mAU·s)	峰面积 %
1		17.659	740.281	49.284
2		19.636	761.780	50.716

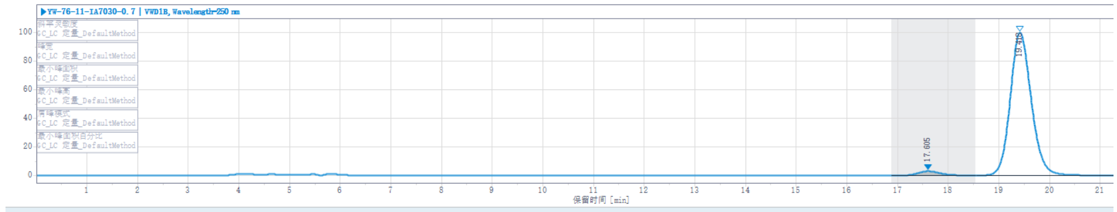

进样结果

峰 汇总

#	名称	RT (min)	峰面积 (mAU·s)	峰面积 %
1		17.605	59.053	3.076
2		19.418	1860.682	96.924

HPLC conditions: Chiralpak IA-H, 30% ^{i}PrOH/Hx eluent, 0.7 mL/min, 250 nm

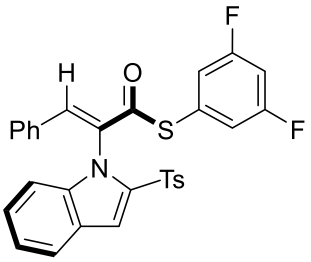

3aj

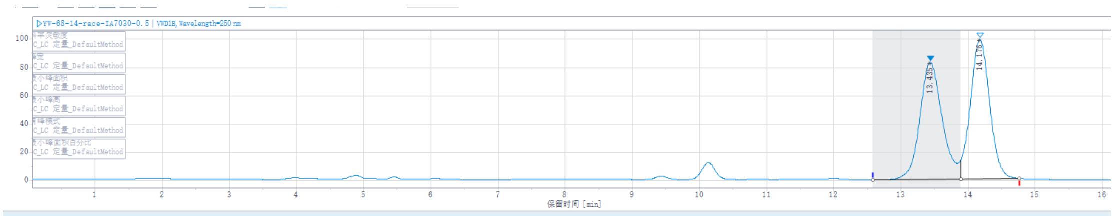

进样结果

峰 汇总

#	名称	RT (min)	峰面积 (mAU·s)	峰面积 %
1		13.435	734.241	49.487
2		14.176	749.451	50.513

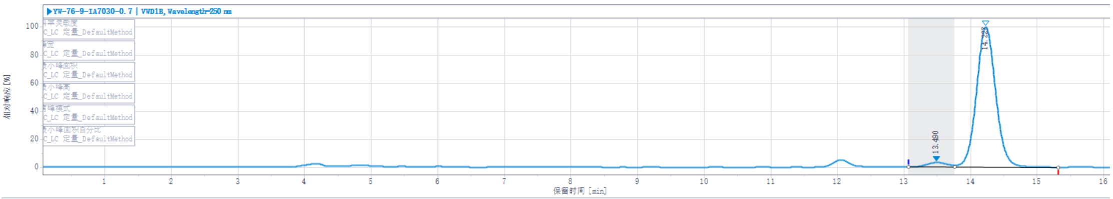

进样结果

峰 汇总

#	名称	RT (min)	峰面积 (mAU·s)	峰面积 %
1		13.490	37.024	3.243
2		14.227	1104.573	96.757

HPLC conditions: Chiralpak IA-H, 30% ^{i}PrOH/Hx eluent, 0.7 mL/min, 250 nm

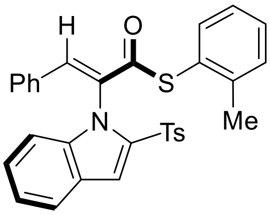

3ak

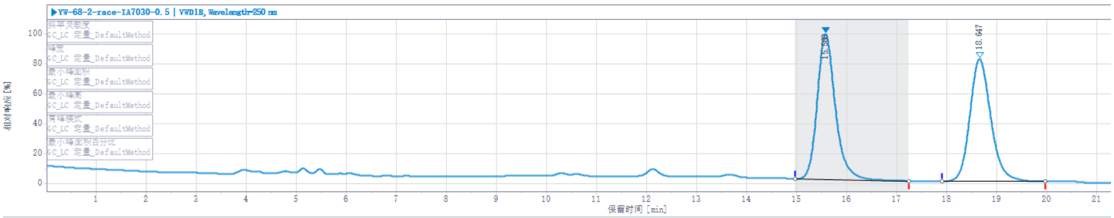

进样结果

峰 汇总

#	名称	RT (min)	峰面积 (mAU·s)	峰面积 %
1		15.568	404.329	50.735
2		18.647	392.607	49.265

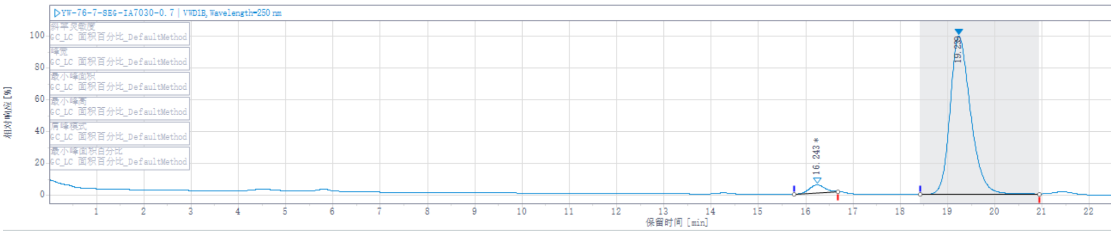

进样结果

峰 汇总

#	名称	RT (min)	峰面积 (mAU·s)	峰面积 %
1		16.243	119.303	3.537
2		19.239	3254.051	96.463

HPLC conditions: Chiralpak IA-H, 30% ^{i}PrOH/Hx eluent, 0.7 mL/min, 250 nm

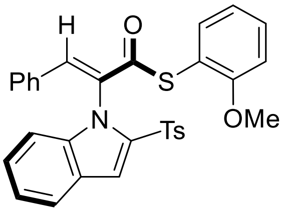

3al

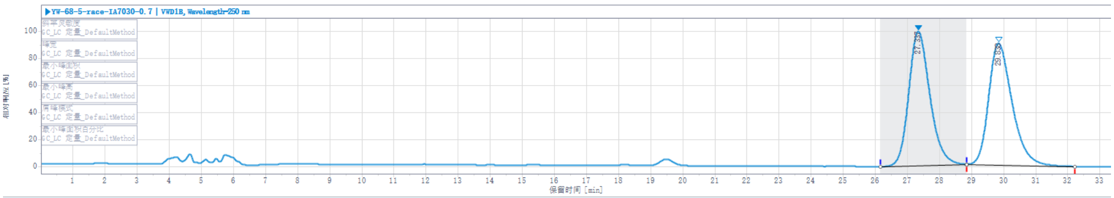

进样结果

峰 汇总

#	名称	RT (min)	峰面积 (mAU·s)	峰面积 %
1		27.337	514.934	49.901
2		29.838	516.981	50.099

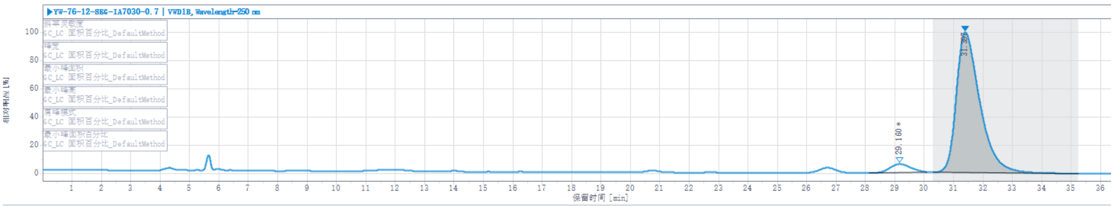

进样结果

峰 汇总

#	名称	RT (min)	峰面积 (mAU·s)	峰面积 %
1		29.160	106.517	5.005
2		31.397	2021.801	94.995

HPLC conditions: Chiralpak IA-H, 30% ^{i}PrOH/Hx eluent, 0.7 mL/min, 250 nm

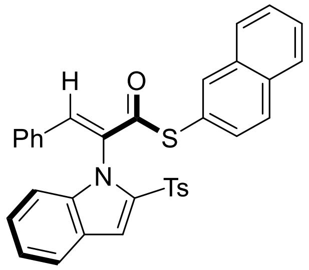

3am

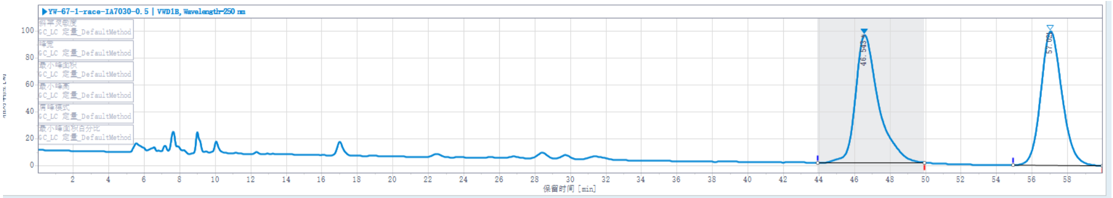

进样结果

峰 汇总

#	名称	RT (min)	峰面积 (mAU·s)	峰面积 %
1		46.543	849.607	50.867
2		57.021	820.655	49.133

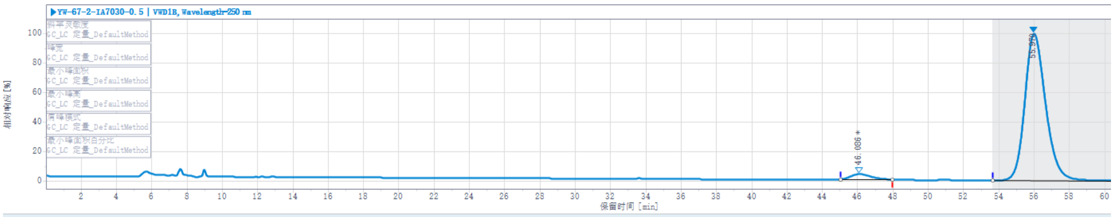

进样结果

峰 汇总

#	名称	RT (min)	峰面积 (mAU·s)	峰面积 %
1		46.086	89.916	3.317
2		55.970	2620.983	96.683

HPLC conditions: Chiralpak IA-H, 30% ^{i}PrOH/Hx eluent, 0.7 mL/min, 250 nm

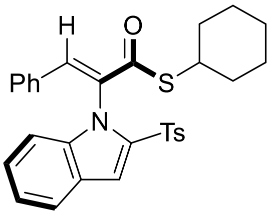

3an

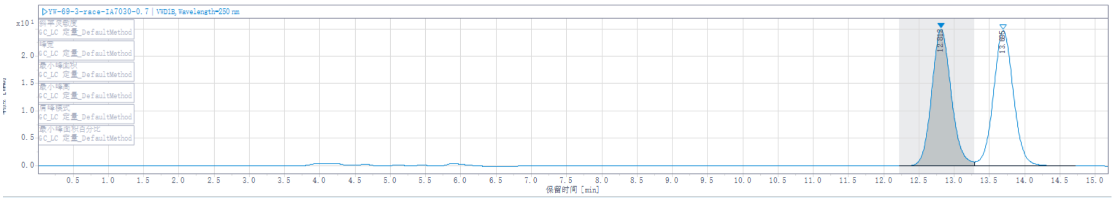

进样结果

峰 汇总

#	名称	RT (min)	峰面积 (mAU·s)	峰面积 %
1		12.812	483.218	49.700
2		13.695	489.058	50.300

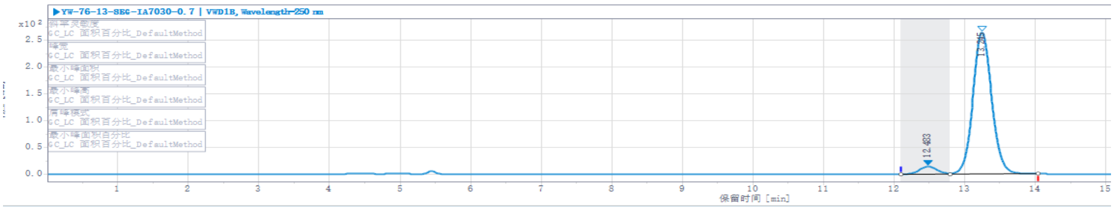

进样结果

峰 汇总

#	名称	RT (min)	峰面积 (mAU·s)	峰面积 %
1		12.483	245.411	4.772
2		13.245	4897.466	95.228

6. Synthesis of 5

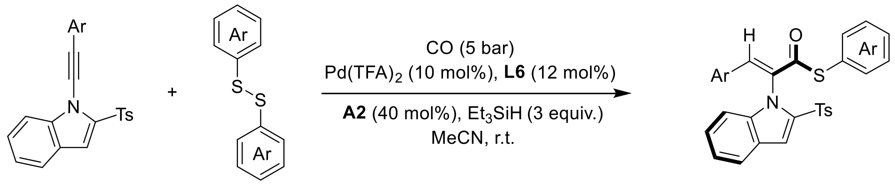

1 4 5

Typical Procedure B: To a mixture of **1** (0.1 mmol), **4** (0.15 mmol), $Pd(TFA)_2$ (0.01 mmol), L**1** (0.012 mmol) and **A2** (0.04 mmol) was added MeCN (0.5 mL), last added Et_3SiH (0.3 mmol). After flushing the autoclave three times with CO, a pressure of 5 bar of CO was adjusted at ambient temperature. Then, the reaction was performed for 24 h at roomtempure. After the reaction was complete, the pressure of autoclave was released carefully. The mixture was diluted with EtOAc (5 mL) and extracted with EtOAc (5 mL x 3). The combined organic layers were washed with brine (10 mL), dried (Na_2SO_4), and concentrated. Further purification by flash column chromatography on silica gel (petroleum ether: EtOAc) provided **3**.

7. Characterization, NMR spectra, and HPLC of products 5

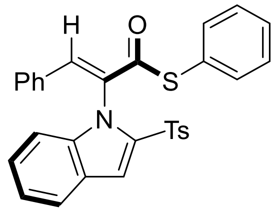

S-phenyl (Z)-3-phenyl-2-(2-tosyl-1H-indol-1-yl)prop-2-enethioate (5a) was synthesized by following Procedure B. The crude material was purified by column chromatography (SiO_2, petroleum ether: EtOAc = 5:1) to provide **5a** as a yellow solid (42 mg, 82% yield).

^{1}H NMR (400 MHz, $CDCl_3$) δ 7.87 (s, 1H), 7.83 (ddd, *J* = 8.7, 7.1, 1.3 Hz, 3H), 7.76 (d, *J* = 0.9 Hz, 1H), 7.36 – 7.31 (m, 1H), 7.30 – 7.26 (m, 1H), 7.23 (d, *J* = 6.0 Hz, 1H), 7.22 (s, 1H), 7.21 – 7.16 (m, 3H), 7.13 (tdd, *J* = 7.7, 3.2, 1.5 Hz, 4H), 6.91 (t, *J* = 7.9 Hz, 2H), 6.49 – 6.42 (m, 2H), 2.36 (s, 3H).

^{13}C NMR (100 MHz, $CDCl_3$) δ 189.4, 144.2, 138.7, 137.4, 136.9, 135.8, 134.6, 130.9, 130.9, 130.8, 129.3, 129.3, 129.0, 128.8, 128.5, 128.4, 127.3, 127.0, 126.0, 123.1, 122.6, 113.1, 111.3, 21.3.

HRMS: (ESI) m/z: $[M+H]^+$ Calcd for $C_{30}H_{24}NO_3S_2^+$ 510.1198; Found 510.1197.

Optical $[\alpha]_{25}^{D}$ = -12.6 ° (c = 0.25, CH_2Cl_2, 92% ee)

HPLC (IA-H, iPrOH/n-hexane = 30/70, flow rate = 0.7 mL/min, 250 nm) t_R = 26.7 min (major), 22.3 min (minor).

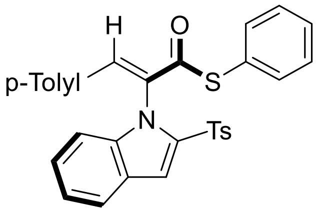

S-phenyl (Z)-3-(p-tolyl)-2-(2-tosyl-1H-indol-1-yl)prop-2-enethioate (5b) was synthesized by following Procedure B. The crude material was purified by column chromatography (SiO_2, petroleum ether: EtOAc = 5:1) to provide **5b** as a yellow solid (45 mg, 85% yield).

^{1}H NMR (400 MHz, $CDCl_3$) δ 7.88 – 7.81 (m, 2H), 7.74 (d, *J* = 0.9 Hz, 1H), 7.71 – 7.65 (m, 2H), 7.39 – 7.30 (m, 6H), 7.30 – 7.26 (m, 1H), 7.14 (dd, *J* = 8.2, 1.1 Hz, 1H), 6.91 (d, *J* = 8.1 Hz, 2H), 6.74 (d, *J* = 8.0 Hz, 2H), 6.35 (d, *J* = 8.2 Hz, 2H), 2.19 (s, 3H), 2.13 (s, 3H).

^{13}C NMR (100 MHz, $CDCl_3$) δ 189.4, 144.1, 141.8, 138.7, 137.5, 136.9, 135.9, 134.6, 131.1, 129.3, 129.3, 129.2, 129.0, 128.4, 128.3, 127.8, 127.4, 126.9, 126.0, 123.1, 122.5, 113.0, 111.3, 21.4, 21.3.

HRMS: (ESI) m/z: $[M+H]^+$ Calcd for $C_{31}H_{26}NO_3S_2^+$ 524.1354; Found 524.1355.

Optical $[\alpha]_{25}^{D}$ = -56.0 ° (c = 0.25, CH_2Cl_2, 92% ee)

HPLC (IA-H, iPrOH/n-hexane = 30/70, flow rate = 0.7 mL/min, 250 nm) t_R = 27.1 min (major), 21.3 min (minor).

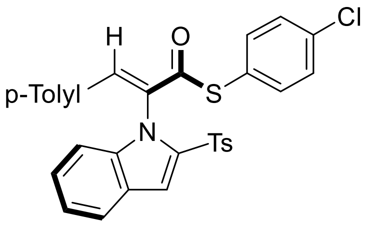

S-(4-chlorophenyl) (Z)-3-(p-tolyl)-2-(2-tosyl-1H-indol-1-yl)prop-2-enethioate (5c) was synthesized by following Procedure B. The crude material was purified by column chromatography (SiO_2, petroleum ether: EtOAc = 5:1) to provide **5c** as a yellow solid (44 mg, 79% yield).

^{1}H NMR (400 MHz, $CDCl_3$) δ 7.84 (s, 1H), 7.68 – 7.64 (m, 2H), 7.63 – 7.57 (m, 2H), 7.52 – 7.46 (m, 2H), 7.24 – 7.19 (m, 2H), 7.14 (tdd, *J* = 7.5, 6.6, 1.4 Hz, 2H), 7.00 (d, *J* = 8.5 Hz, 1H), 6.90 (t, *J* = 7.9 Hz, 2H), 6.86 (dd, *J* = 7.3, 1.4 Hz, 2H), 6.44 (dd, *J* = 8.3, 1.4 Hz, 2H), 2.16 (s, 3H), 2.10 (s, 3H).

^{13}C NMR (100 MHz, $CDCl_3$) δ 189.3, 144.1, 137.4, 137.1, 136.8, 136.1, 135.6, 132.2, 131.0, 130.9, 130.8, 129.4, 129.0, 128.6, 128.5, 128.3, 128.3, 126.6, 126.3, 124.0, 122.6, 112.7, 110.9, 21.4, 21.3.

HRMS: (ESI) m/z: $[M+H]^+$ Calcd for $C_{31}H_{25}ClNO_3S_2^+$ 558.0964; Found 558.0966.

Optical $[\alpha]_{25}^{D}$ = -52.5 ° (c = 0.25, CH_2Cl_2, 90% ee)

HPLC (IA-H, iPrOH/n-hexane = 30/70, flow rate = 0.7 mL/min, 250 nm) t_R = 36.5 min (major), 27.0 min (minor).

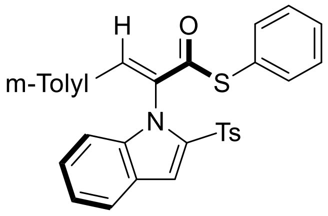

S-phenyl (Z)-3-(m-tolyl)-2-(2-tosyl-1H-indol-1-yl)prop-2-enethioate (5d) was synthesized by following Procedure B. The crude material was purified by column chromatography (SiO_2, petroleum ether: EtOAc = 5:1) to provide **5d** as a yellow solid (41 mg, 78% yield).

^{1}H NMR (400 MHz, $CDCl_3$) δ 7.83 (d, *J* = 6.7 Hz, 2H), 7.75 (d, *J* = 0.8 Hz, 1H), 7.70 – 7.65 (m, 2H), 7.37 (s, 5H), 7.34 – 7.30 (m, 1H), 7.29 – 7.26 (m, 1H), 7.13 (dd, *J* = 8.1, 1.2 Hz, 1H), 6.94 (d, *J* = 7.6 Hz, 1H), 6.89 (d, *J* = 8.1 Hz, 2H), 6.79 (t, *J* = 7.7 Hz, 1H), 6.24 (d, *J* = 7.8 Hz, 1H), 6.19 (d, *J* = 1.8 Hz, 1H), 2.11 (s, 3H), 1.97 (s, 3H).

^{13}C NMR (100 MHz, $CDCl_3$) δ 189.5, 144.0, 138.7, 137.9, 137.5, 136.9, 135.8, 134.7, 131.7, 131.7, 130.8, 129.3, 129.2, 129.0, 128.6, 128.4, 128.3, 128.1, 127.4, 126.9, 126.0, 123.0, 122.5, 113.0, 111.3, 21.3, 21.0.

HRMS: (ESI) m/z: $[M+H]^+$ Calcd for $C_{31}H_{25}NO_3S_2^+$ 524.1354; Found 523.1353.

Optical $[\alpha]_{25}^{D}$ = -20.8 ° (c = 0.25, CH_2Cl_2, 90% ee)

HPLC (IA-H, iPrOH/n-hexane = 30/70, flow rate = 0.7 mL/min, 250 nm) t_R = 21.4 min (major), 18.4 min (minor).

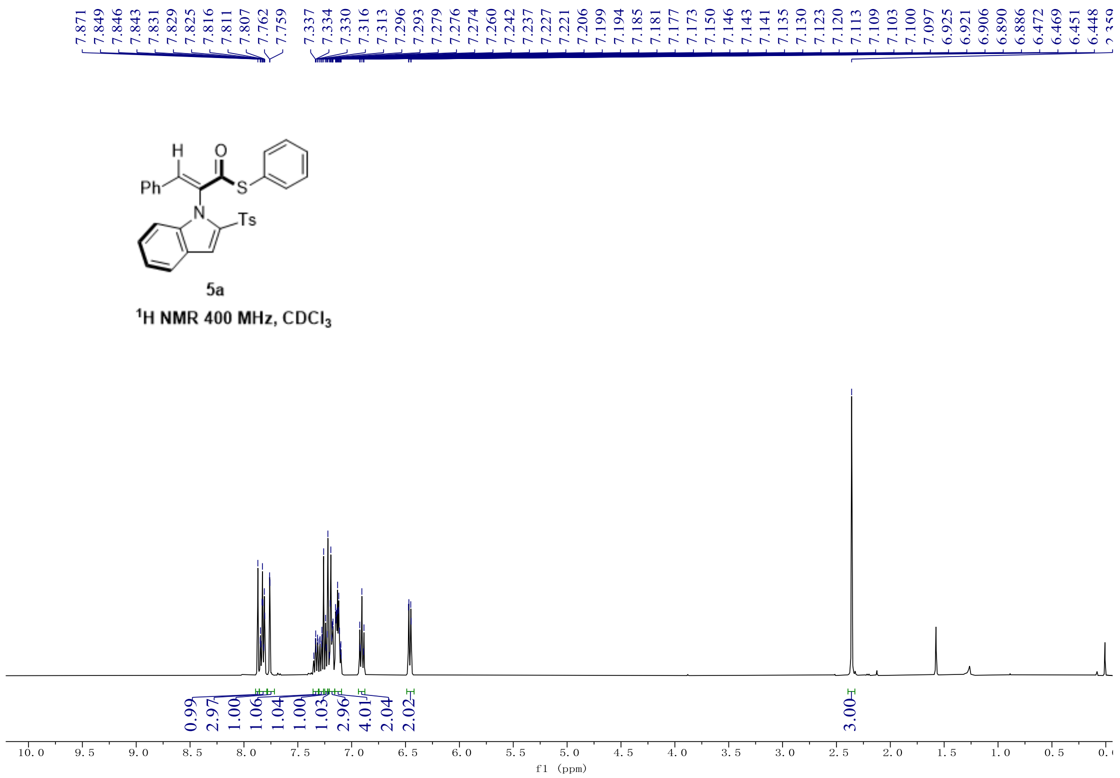
7.871 7.849 7.846 7.843 7.831 7.829 7.825 7.816 7.811 7.807 7.762 7.759 7.337 7.334 7.330 7.316 7.313 7.296 7.293 7.279 7.276 7.274 7.260 7.242 7.237 7.227 7.221 7.206 7.199 7.194 7.185 7.181 7.177 7.173 7.150 7.146 7.143 7.141 7.135 7.130 7.123 7.120 7.113 7.109 7.103 7.100 7.097 6.925 6.921 6.906 6.890 6.886 6.472 6.469 6.451 6.448
H
O
Ph
S
N
Ts
5a
^{1}H NMR 400 MHz, $CDCl_3$
0.99 2.97 1.00 1.06 1.04 1.00 1.03 2.96 4.01 2.04 2.02
3.00
10.0 9.5 9.0 8.5 8.0 7.5 7.0 6.5 6.0 5.5 5.0 4.5 4.0 3.5 3.0 2.5 2.0 1.5 1.0 0.5
f1 (ppm)

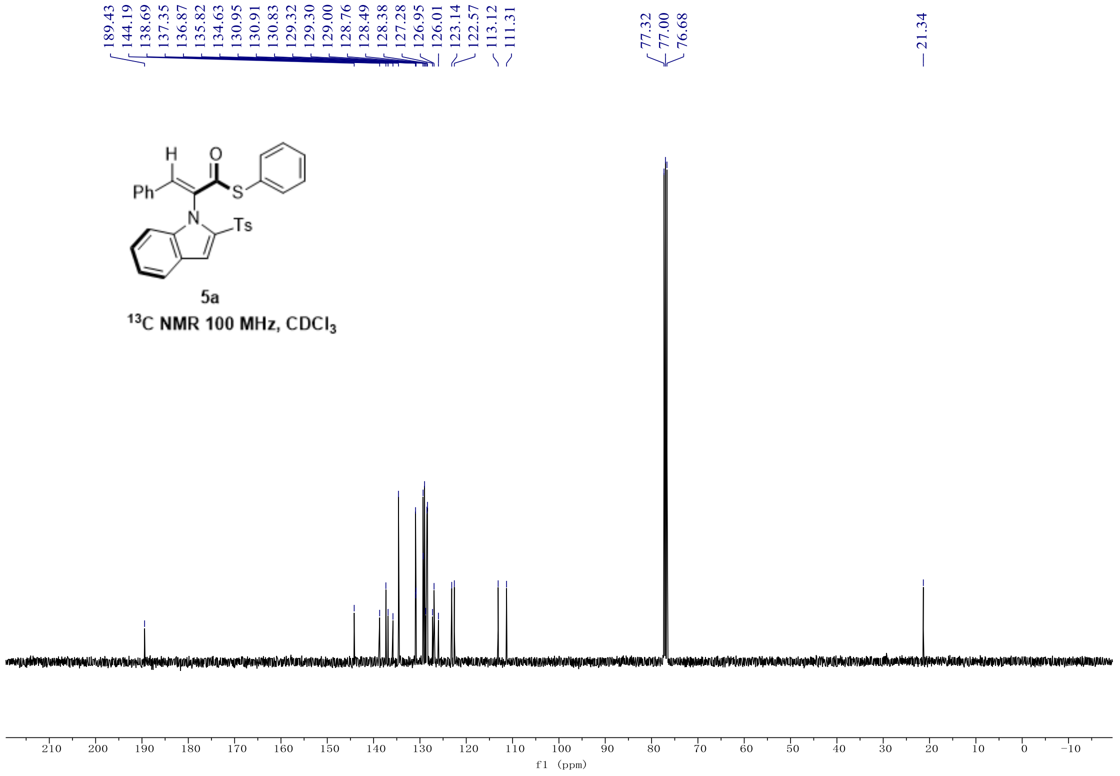
189.43 144.19 138.69 137.35 136.87 135.82 134.63 130.95 130.91 130.83 129.32 129.30 129.00 128.76 128.49 128.38 127.28 126.95 126.01 123.14 122.57 113.12 111.31
77.32 77.00 76.68
21.34
H
O
Ph
S
N
Ts
5a
^{13}C NMR 100 MHz, $CDCl_3$
210 200 190 180 170 160 150 140 130 120 110 100 90 80 70 60 50 40 30 20 10 0 -10
f1 (ppm)

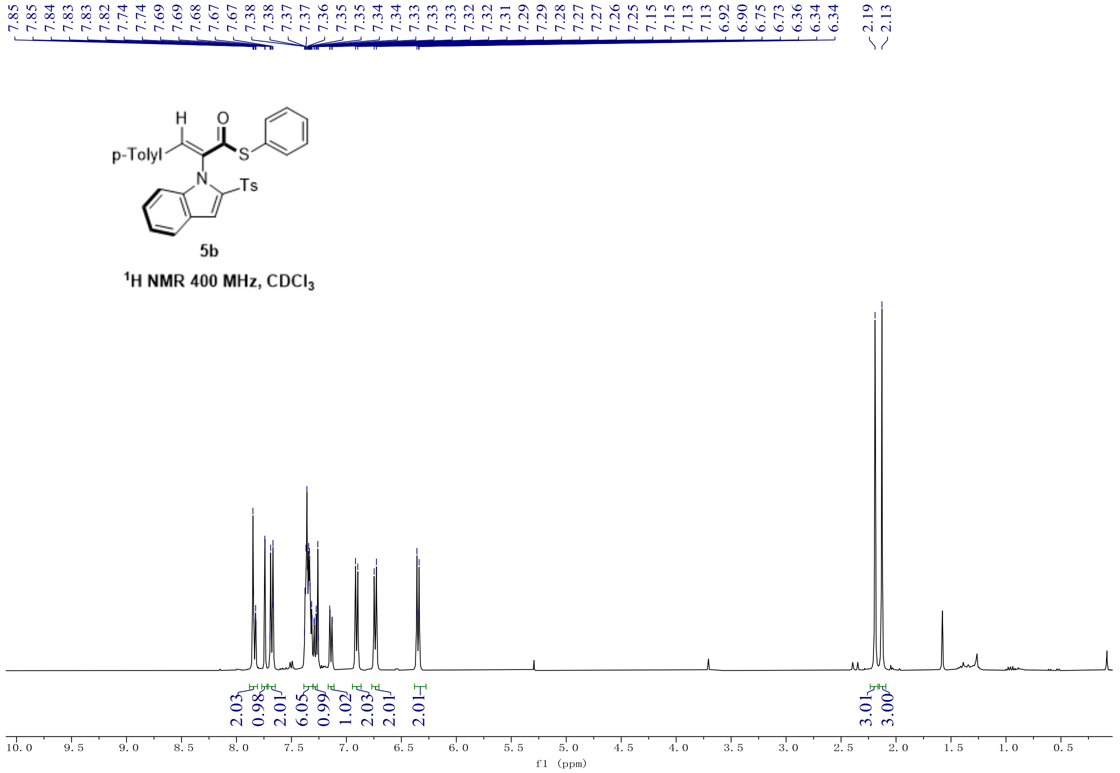
7.85 7.85 7.84 7.83 7.83 7.82 7.74 7.74 7.69 7.69 7.68 7.67 7.67 7.38 7.38 7.37 7.37 7.36 7.35 7.35 7.34 7.34 7.33 7.33 7.33 7.32 7.32 7.31 7.29 7.29 7.28 7.27 7.27 7.26 7.25 7.15 7.15 7.13 7.13 6.92 6.90 6.75 6.73 6.36 6.34 6.34 2.19 2.13
H
O
p-Tolyl
S
N
Ts
5b
^{1}H NMR 400 MHz, CDCl$_3$
2.03 0.98 2.01 6.05 0.99 1.02 2.03 2.01 2.01 3.01 3.00
10.0 9.5 9.0 8.5 8.0 7.5 7.0 6.5 6.0 5.5 5.0 4.5 4.0 3.5 3.0 2.5 2.0 1.5 1.0 0.5
f1 (ppm)

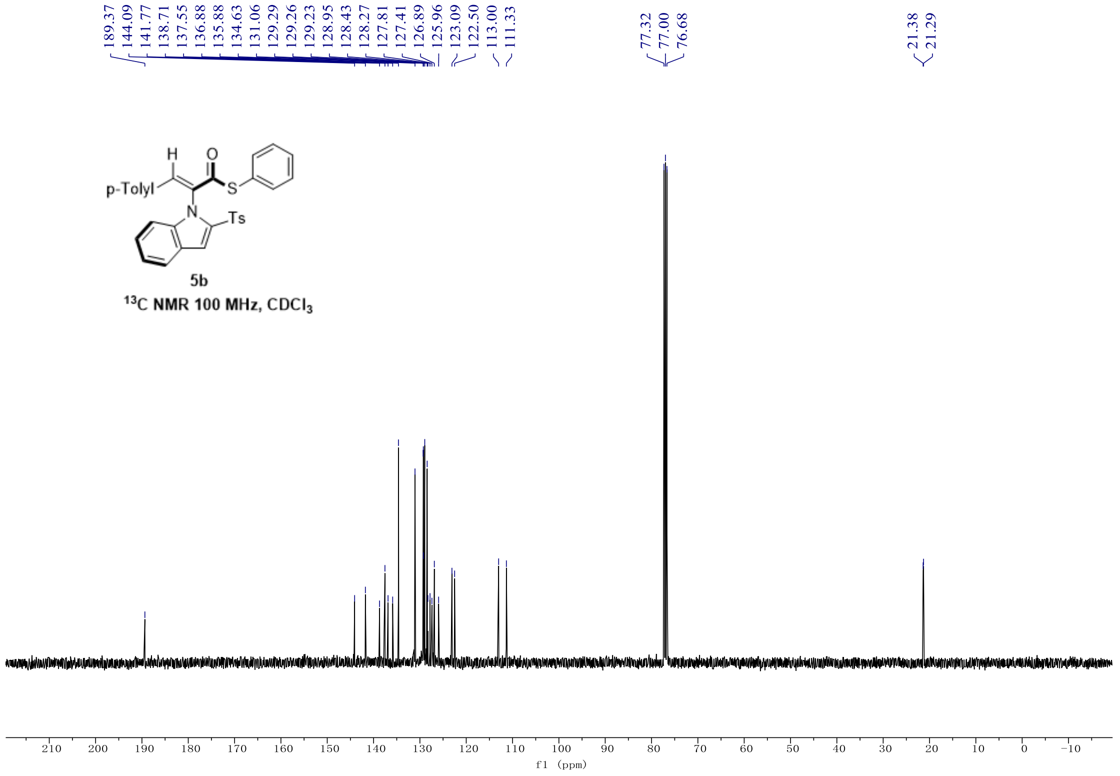
189.37 144.09 141.77 138.71 137.55 136.88 135.88 134.63 131.06 129.29 129.26 129.23 128.95 128.43 128.27 127.81 127.41 126.89 125.96 123.09 122.50 113.00 111.33 77.32 77.00 76.68 21.38 21.29
H
O
p-Tolyl
S
N
Ts
5b
^{13}C NMR 100 MHz, CDCl$_3$
210 200 190 180 170 160 150 140 130 120 110 100 90 80 70 60 50 40 30 20 10 0 -10
f1 (ppm)

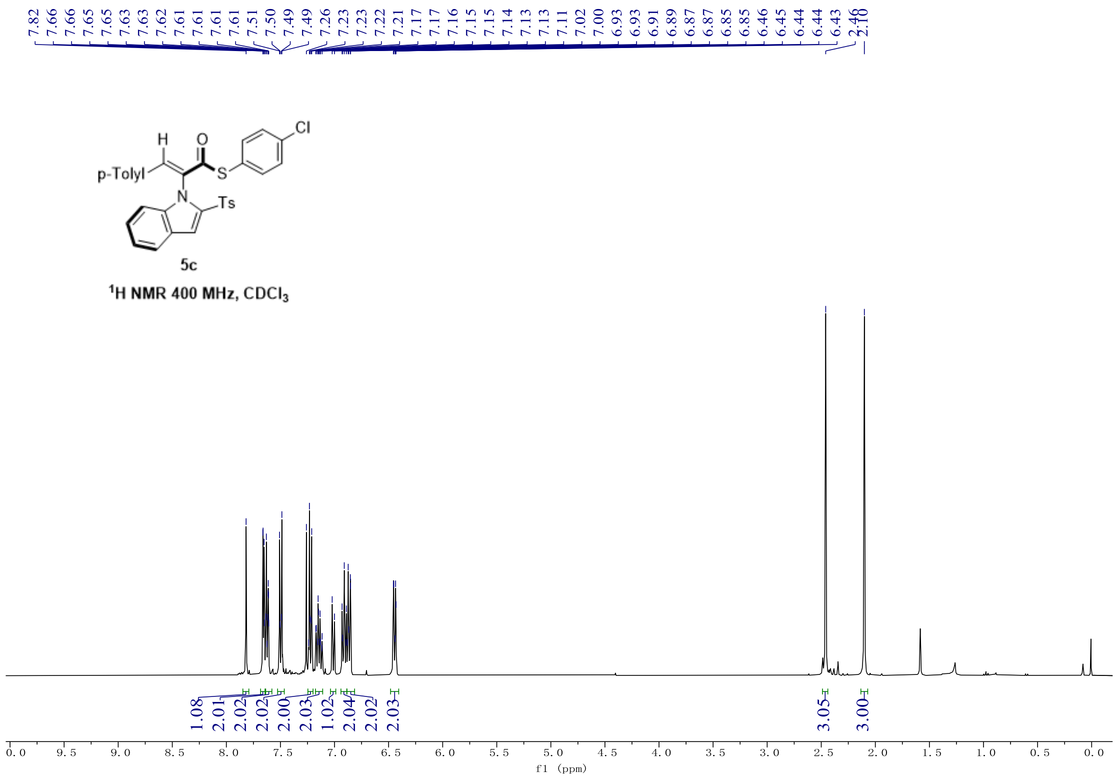
7.82 7.66 7.66 7.65 7.65 7.63 7.63 7.62 7.61 7.61 7.61 7.61 7.51 7.50 7.49 7.49 7.26 7.23 7.23 7.22 7.21 7.17 7.17 7.16 7.15 7.15 7.14 7.13 7.13 7.11 7.02 7.00 6.93 6.93 6.91 6.89 6.87 6.87 6.85 6.85 6.46 6.45 6.44 6.43 2.46 2.10
H
O
Cl
p-Tolyl
S
N
Ts
5c
1H NMR 400 MHz, CDCl3
1.08 2.01 2.02 2.02 2.00 2.03 1.02 2.04 2.02 2.03
3.05 3.00
f1 (ppm)

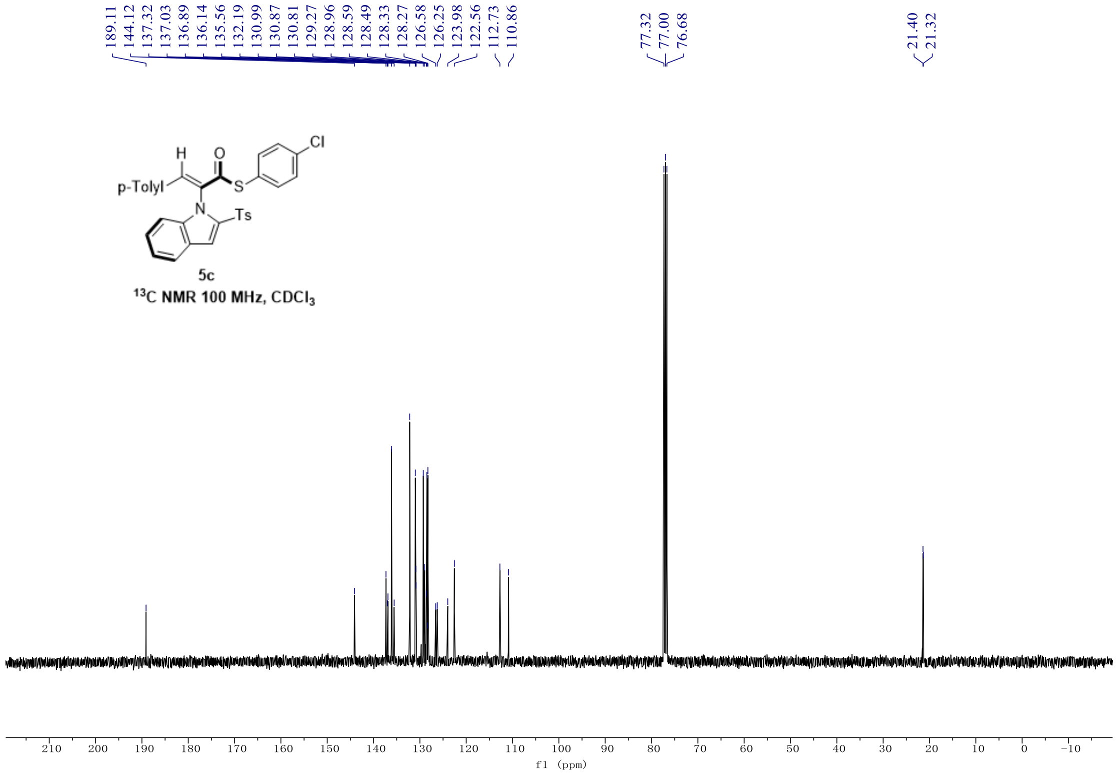
189.11 144.12 137.32 137.03 136.89 136.14 135.56 132.19 130.99 130.87 130.81 129.27 128.96 128.59 128.49 128.33 128.27 126.58 126.25 123.98 122.56 112.73 110.86
77.32 77.00 76.68
21.40 21.32
H
O
Cl
p-Tolyl
S
N
Ts
5c
13C NMR 100 MHz, CDCl3
f1 (ppm)

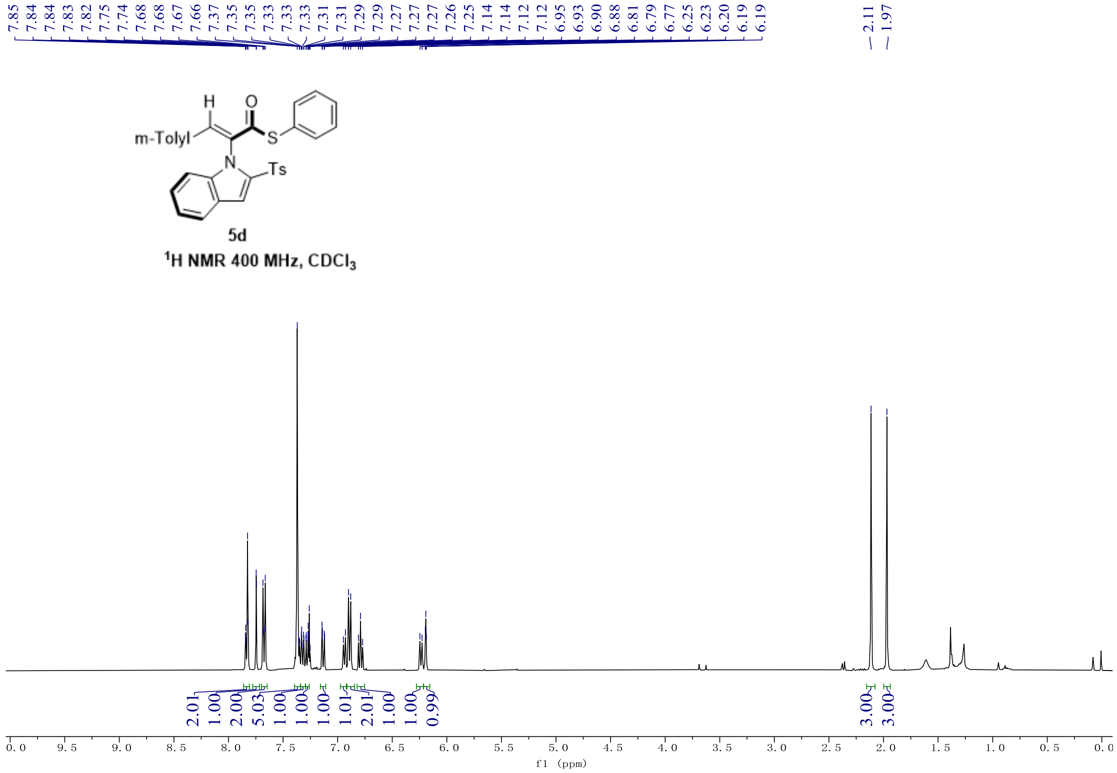
7.85
7.84
7.84
7.83
7.82
7.75
7.74
7.68
7.68
7.67
7.66
7.37
7.35
7.35
7.33
7.33
7.33
7.31
7.31
7.29
7.29
7.27
7.27
7.27
7.26
7.25
7.14
7.14
7.12
7.12
6.95
6.93
6.90
6.88
6.81
6.79
6.77
6.25
6.23
6.20
6.19
6.19
2.11
1.97
m-Tolyl
H
O
S
N
Ts
5d
1H NMR 400 MHz, CDCl3
2.01
1.00
2.00
5.03
1.00
1.00
1.00
1.01
2.01
1.00
1.00
0.99
3.00
3.00
f1 (ppm)

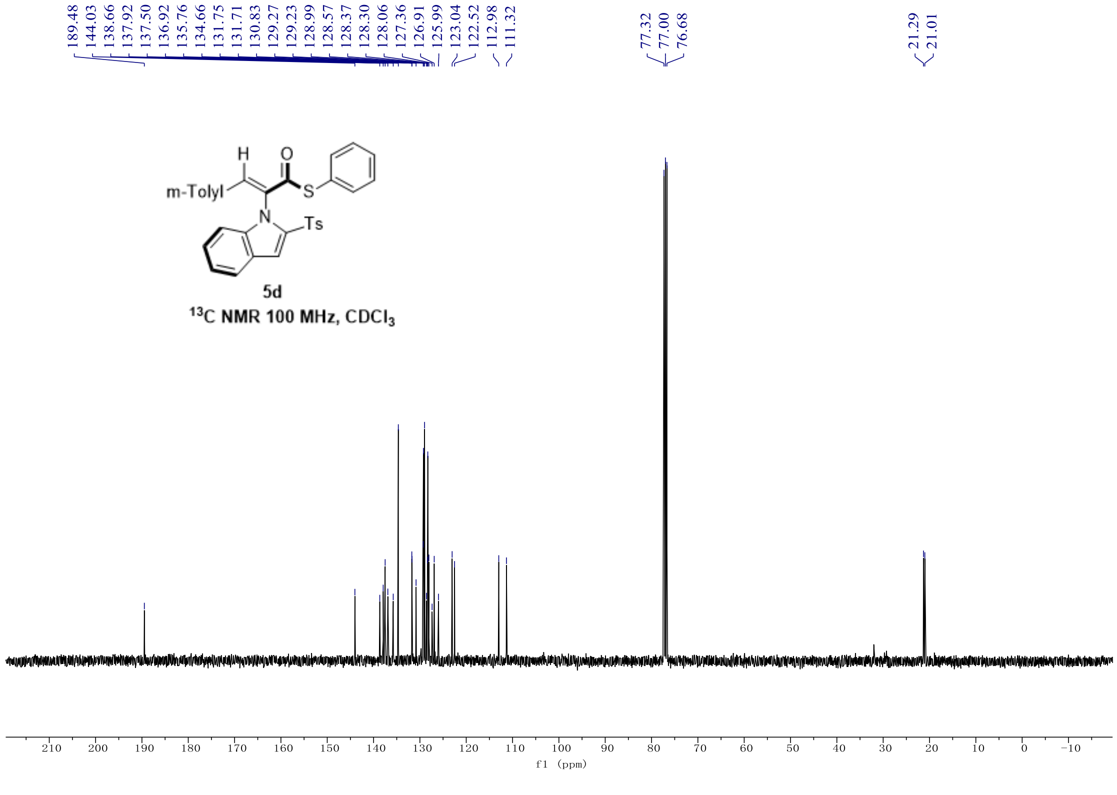
189.48
144.03
138.66
137.92
137.50
136.92
135.76
134.66
131.75
131.71
130.83
129.27
129.23
128.99
128.57
128.37
128.30
128.06
127.36
126.91
125.99
123.04
122.52
112.98
111.32
77.32
77.00
76.68
21.29
21.01
m-Tolyl
H
O
S
N
Ts
5d
13C NMR 100 MHz, CDCl3
f1 (ppm)

HPLC conditions: Chiralpak IA-H, 30% ^{i}PrOH/Hx eluent, 0.7 mL/min, 250 nm

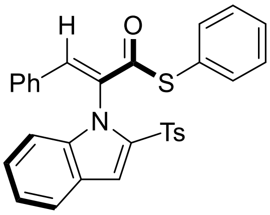

5a

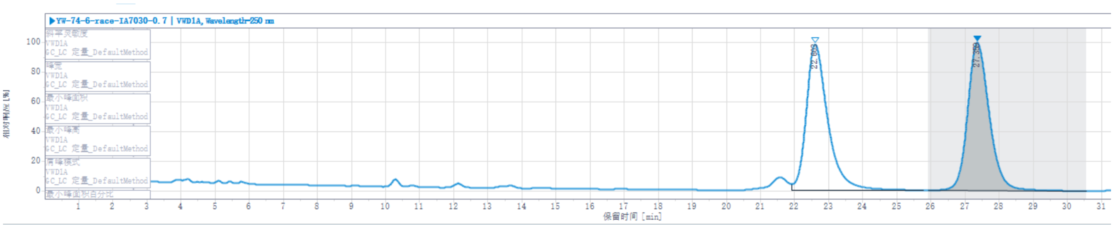

进样结果

峰 汇总

#	名称	RT (min)	峰面积 (mAU·s)	峰面积 %
1		22.603	822.312	49.643
2		27.359	834.125	50.357

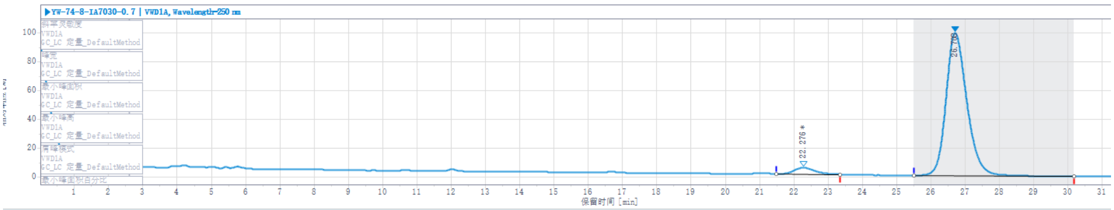

进样结果

峰 汇总

#	名称	RT (min)	峰面积 (mAU·s)	峰面积 %
1		22.276	49.559	4.197
2		26.708	1131.190	95.803

HPLC conditions: Chiralpak IA-H, 30% ^{i}PrOH/Hx eluent, 0.7 mL/min, 250 nm

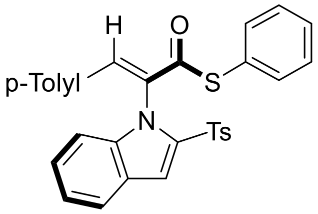

5b

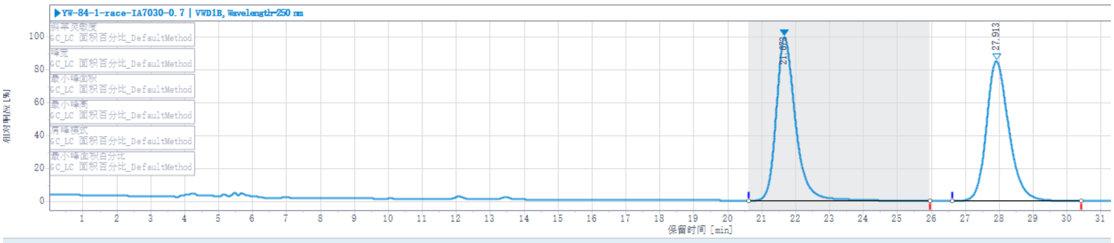

进样结果

#	名称	RT (min)	峰面积 (mAU·s)	峰面积 %
1		21.673	565.059	50.275
2		27.913	558.884	49.725

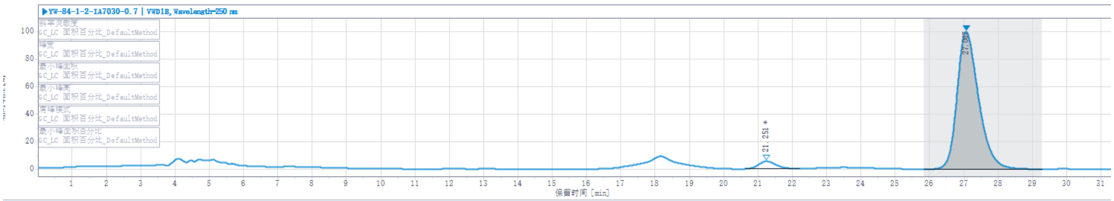

进样结果

#	名称	RT (min)	峰面积 (mAU·s)	峰面积 %
1		21.251	76.924	4.204
2		27.067	1753.071	95.796

HPLC conditions: Chiralpak IA-H, 30% ^{i}PrOH/Hx eluent, 0.7 mL/min, 250 nm

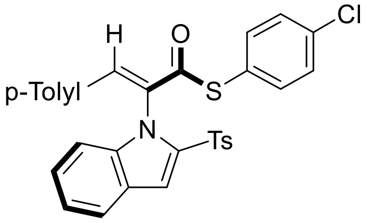

5c

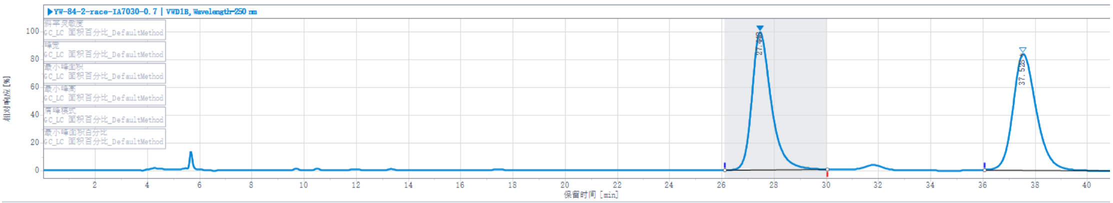

进样结果

峰 汇总

#	名称	RT (min)	峰面积 (mAU·s)	峰面积 %
1		27.448	1015.193	49.517
2		37.528	1034.993	50.483

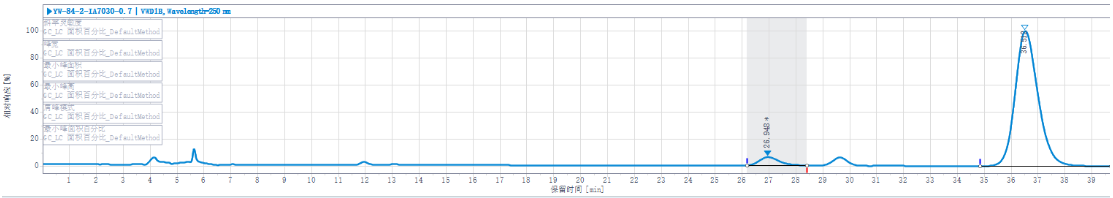

进样结果

峰 汇总

#	名称	RT (min)	峰面积 (mAU·s)	峰面积 %
1		26.948	46.859	5.107
2		36.512	870.677	94.893

HPLC conditions: Chiralpak IA-H, 30% ^{i}PrOH/Hx eluent, 0.7 mL/min, 250 nm

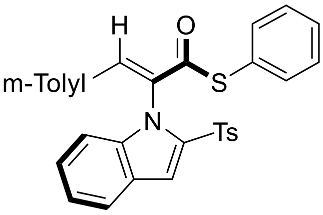

5d

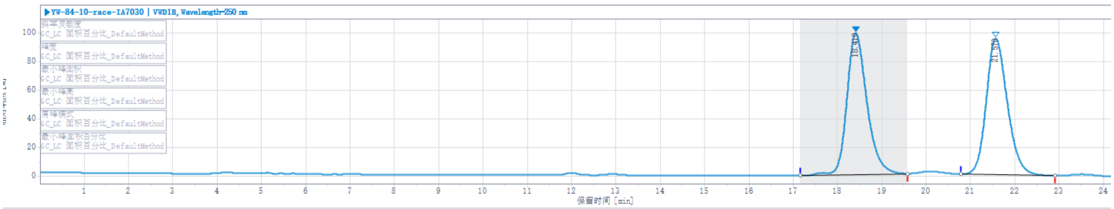

进样结果

峰 汇总

#	名称	RT (min)	峰面积 (mAU·s)	峰面积 %
1		18.410	870.478	49.777
2		21.570	878.265	50.223

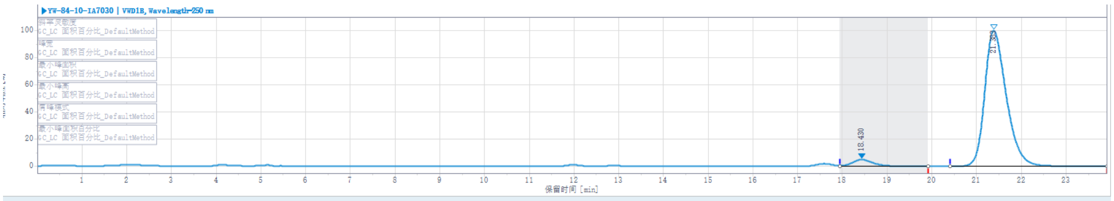

进样结果

峰 汇总

#	名称	RT (min)	峰面积 (mAU·s)	峰面积 %
1		18.430	169.159	4.652
2		21.383	3467.340	95.348

8. Synthesis of 7

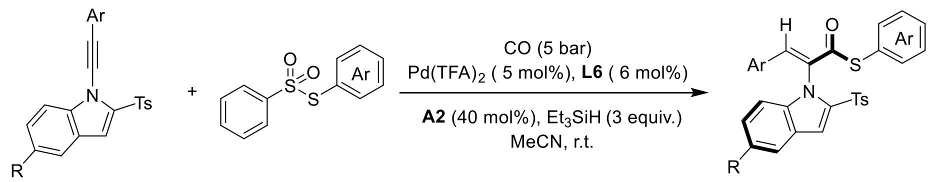

1 **6** **7**

Typical Procedure C: To a mixture of **1** (0.1 mmol), **6** (0.15 mmol), $Pd(TFA)_2$ (0.005 mmol), L**6** (0.006 mmol) and **A2** (0.04 mmol) was added MeCN (1 mL), last added Et_3SiH (0.3 mmol). After flushing the autoclave three times with CO, a pressure of 5 bar of CO was adjusted at ambient temperature. Then, the reaction was performed for 24 h at roomtempure. After the reaction was complete, the pressure of autoclave was released carefully. The mixture was diluted with EtOAc (5 mL) and extracted with EtOAc (5 mL x 3). The combined organic layers were washed with brine (10 mL), dried (Na_2SO_4), and concentrated. Further purification by flash column chromatography on silica gel (petroleum ether: EtOAc) provided **7**.

9. Characterization, NMR spectra, and HPLC of products 7

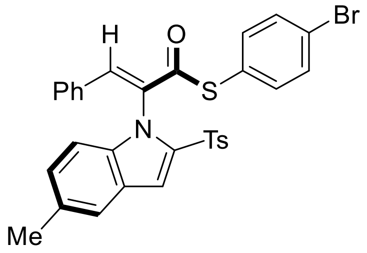

S-(4-bromophenyl) (Z)-2-(5-methyl-2-tosyl-1H-indol-1-yl)-3-phenylprop-2-enethioate (7a) was synthesized by following Procedure C. The crude material was purified by column chromatography (SiO_2, petroleum ether: EtOAc = 5:1) to provide **7a** as a yellow solid (48 mg, 80% yield).

^{1}H NMR (400 MHz, $CDCl_3$) δ 7.81 (s, 1H), 7.68 – 7.64 (m, 2H), 7.63 – 7.57 (m, 2H), 7.52 – 7.46 (m, 2H), 7.24 – 7.19 (m, 2H), 7.14 (tdd, *J* = 7.5, 6.6, 1.4 Hz, 2H), 7.00 (d, *J* = 8.5 Hz, 1H), 6.90 (t, *J* = 7.9 Hz, 2H), 6.86 (dd, *J* = 7.3, 1.4 Hz, 2H), 6.44 (dd, *J* = 8.3, 1.4 Hz, 2H), 2.45 (s, 3H), 2.10 (s, 3H).

^{13}C NMR (100 MHz, $CDCl_3$) δ 189.1, 144.1, 137.3, 137.0, 136.9, 136.1, 135.6, 132.2, 131.0, 130.9, 130.8, 129.3, 129.0, 128.6, 128.5, 128.3, 128.3, 126.6, 126.3, 124.0, 122.6, 112.7, 110.9, 21.4, 21.3.

HRMS: (ESI) m/z: $[M+H]^+$ Calcd for $C_{31}H_{25}BrNO_3S_2^+$ 602.0459; Found 602.0458.

Optical $[\alpha]^D_{25}$ = -41.2 ° (c = 0.25, CH_2Cl_2, 90% ee)

HPLC (IA-H, iPrOH/n-hexane = 30/70, flow rate = 0.7 mL/min, 250 nm) t_R = 36.4 min (major), 31.2 min (minor).

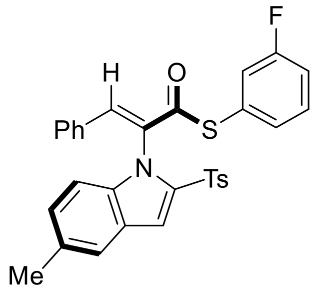

S-(4-bromophenyl) (Z)-2-(5-methyl-2-tosyl-1H-indol-1-yl)-3-phenylprop-2-enethioate (7b) was synthesized by following Procedure C. The crude material was purified by column chromatography (SiO_2, petroleum ether: EtOAc = 5:1) to provide **7b** as a yellow solid (52 mg, 96% yield).

^{1}H NMR (400 MHz, $CDCl_3$) δ 7.84 (s, 1H), 7.67 (s, 2H), 7.65 (d, *J* = 1.9 Hz, 1H), 7.61 (d, *J* = 1.6 Hz, 1H), 7.33 (ddd, *J* = 9.0, 7.8, 6.0 Hz, 1H), 7.15 (tdt, *J* = 7.7, 2.5, 1.4 Hz, 3H), 7.11 – 7.05 (m, 2H), 7.02 (d, *J* = 8.5 Hz, 1H), 6.93 (t, *J* = 7.8 Hz, 2H), 6.89 (d, *J* = 8.1 Hz, 2H), 6.51 – 6.44 (m, 2H), 2.46 (s, 3H), 2.12 (s, 3H).

^{13}C NMR (100 MHz, $CDCl_3$) δ 188.9, 162.4 (d, *J* = 248.7 Hz), 144.2, 137.6, 137.1, 136.9, 135.6, 132.2, 131.0, 130.9 (d, *J* = 8.5 Hz), 130.3 (d, *J* = 3.2 Hz), 130.2, 130.1, 129.3, 129.2, 129.0, 128.6, 128.5, 128.3, 126.3, 122.6, 121.5 (d, *J* = 22.8 Hz), 116.4 (d, *J* = 20.9 Hz), 112.8, 110.9, 21.4, 21.3.

^{19}F NMR (376 MHz, $CDCl_3$) δ -111.84.

HRMS: (ESI) m/z: $[M+H]^+$ Calcd for $C_{31}H_{25}FNO_3S_2^+$ 542.1260; Found 542.1259.

Optical $[\alpha]_{25}^{D}$ = -34.2 ° (c = 0.25, CH_2Cl_2, 90% ee)

HPLC (IA-H, iPrOH/n-hexane = 30/70, flow rate = 0.7 mL/min, 250 nm) t_R = 18.9 min (major), 17.7 min (minor).

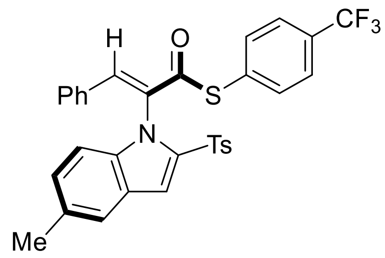

S-(4-(trifluoromethyl)phenyl) (Z)-2-(5-methyl-2-tosyl-1H-indol-1-yl)-3-phenylprop-2-enethioate (7c) was synthesized by following Procedure C. The crude material was purified by column chromatography (SiO_2, petroleum ether: EtOAc = 5:1) to provide **7c** as a yellow solid (46 mg, 78% yield).

^{1}H NMR (400 MHz, $CDCl_3$) δ 7.83 (s, 1H), 7.67 (s, 1H), 7.66 (s, 1H), 7.63 (s, 2H), 7.62 (s, 1H), 7.61 (s, 1H), 7.51 (d, J = 8.2 Hz, 2H), 7.19 – 7.13 (m, 2H), 7.02 (d, J = 8.5 Hz, 1H), 6.91 (t, J = 7.7 Hz, 2H), 6.86 (d, J = 8.0 Hz, 2H), 6.47 – 6.42 (m, 2H), 2.47 (s, 3H), 2.09 (s, 3H).

^{13}C NMR (100 MHz, $CDCl_3$) δ 188.7, 144.2, 137.6, 137.0, 136.9, 135.6, 134.8, 134.7, 132.3, 131.0, 131.0, 130.7, 129.3, 129.0, 128.5, 128.5, 128.4, 128.3, 126.3, 125.7 (q, J = 3.9 Hz), 123.4 (q, J = 272.6 Hz), 122.6, 112.8, 110.8, 21.4, 21.3.

^{19}F NMR (376 MHz, $CDCl_3$) δ -62.81.

HRMS: (ESI) m/z: $[M+H]^+$ Calcd for $C_{32}H_{25}F_3NO_3S_2^+$ 592.1228; Found 592.1228.

Optical $[\alpha]_{25}^{D}$ = -67.5 ° (c = 0.25, CH_2Cl_2, 90% ee)

HPLC (IA-H, iPrOH/n-hexane = 30/70, flow rate = 0.7 mL/min, 250 nm) t_R = 26.5 min (major), 23.4 min (minor).

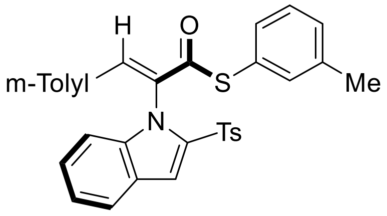

S-(m-tolyl) (Z)-3-(m-tolyl)-2-(2-tosyl-1H-indol-1-yl)prop-2-enethioate (7d) was synthesized by following Procedure C. The crude material was purified by column chromatography (SiO_2, petroleum ether: EtOAc = 5:1) to provide **5b** as a yellow solid (46 mg, 86% yield).

^{1}H NMR (400 MHz, $CDCl_3$) δ 7.78 – 7.72 (m, 2H), 7.66 (d, J = 0.9 Hz, 1H), 7.63 – 7.56 (m, 2H), 7.30 – 7.19 (m, 2H), 7.18 – 7.15 (m, 1H), 7.14 – 7.02 (m, 4H), 6.85 (d, J = 7.7 Hz, 1H), 6.81 (d, J = 8.1 Hz, 2H), 6.71 (t, J = 7.7 Hz, 1H), 6.15 (d, J = 7.8 Hz, 1H), 6.11 (d, J = 1.8 Hz, 1H), 2.27 (s, 3H), 2.04 (s, 3H), 1.89 (s, 3H).

^{13}C NMR (100 MHz, $CDCl_3$) δ 189.7, 144.0, 138.8, 138.7, 137.9, 137.4, 136.9, 135.7, 135.2, 131.7, 131.7, 131.5, 130.9, 130.2, 129.2, 128.8, 128.6, 128.4, 128.3, 128.1, 126.9, 126.9, 126.0, 123.0, 122.5, 113.0, 111.3, 21.3, 21.2, 21.0.

HRMS: (ESI) m/z: $[M+H]^+$ Calcd for $C_{32}H_{28}NO_3S_2^+$ 538.1511; Found 538.1510.

Optical $[\alpha]_{25}^{D}$ = -25.3 ° (c = 0.25, CH_2Cl_2, 92% ee)

HPLC (IA-H, iPrOH/n-hexane = 30/70, flow rate = 0.7 mL/min, 250 nm) t_R = 26.8 min (major), 21.0 min (minor).

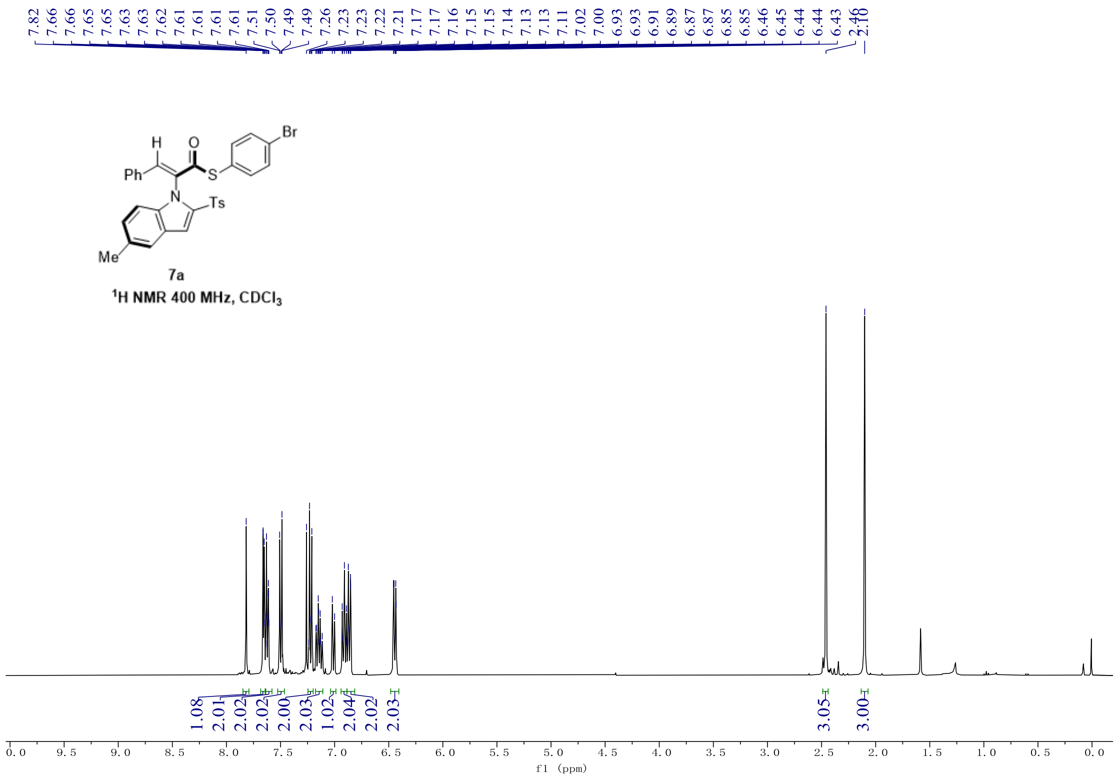
7.82 7.66 7.66 7.65 7.65 7.63 7.63 7.62 7.61 7.61 7.61 7.61 7.51 7.50 7.49 7.49 7.26 7.23 7.23 7.22 7.21 7.17 7.17 7.16 7.15 7.15 7.14 7.13 7.13 7.11 7.02 7.00 6.93 6.93 6.91 6.89 6.87 6.87 6.85 6.85 6.46 6.45 6.44 6.44 6.43 2.46 2.10
H
O
Br
Ph
S
N
Ts
Me
7a
1H NMR 400 MHz, CDCl3
1.08 2.01 2.02 2.02 2.00 2.03 1.02 2.04 2.02 2.03
3.05 3.00
0.0 9.5 9.0 8.5 8.0 7.5 7.0 6.5 6.0 5.5 5.0 4.5 4.0 3.5 3.0 2.5 2.0 1.5 1.0 0.5 0.0
f1 (ppm)

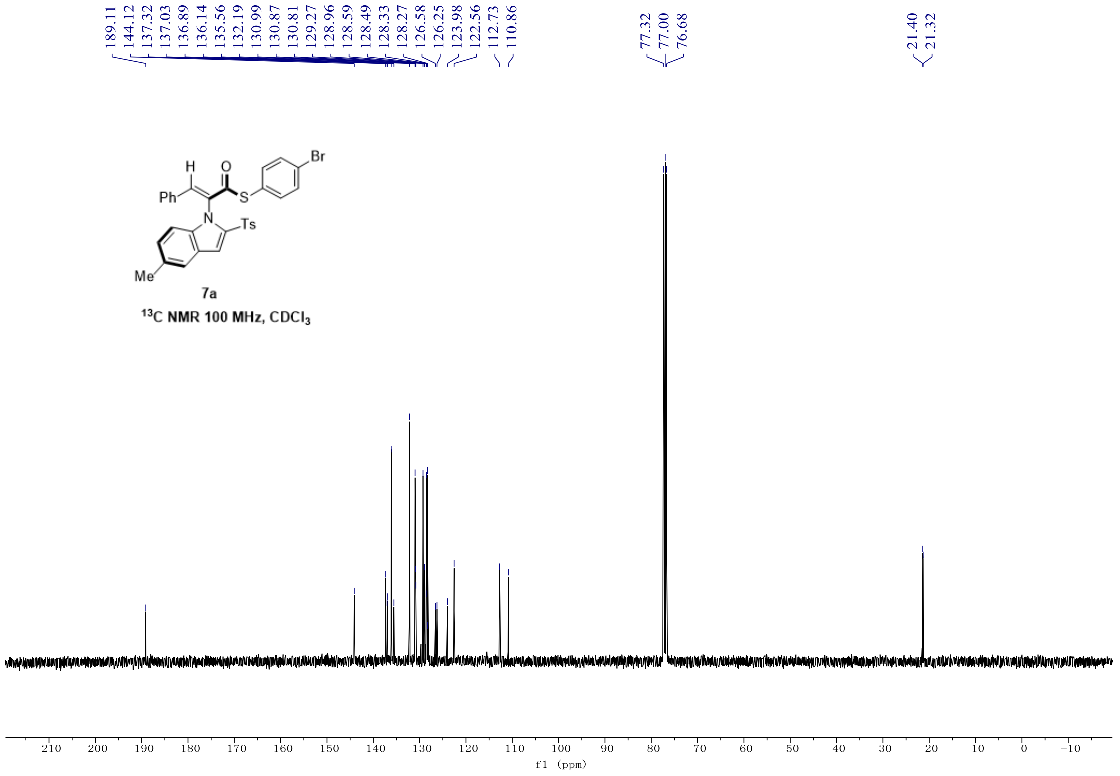
189.11 144.12 137.32 137.03 136.89 136.14 135.56 132.19 130.99 130.87 130.81 129.27 128.96 128.59 128.49 128.33 128.27 126.58 126.25 123.98 122.56 112.73 110.86
77.32 77.00 76.68
21.40 21.32
H
O
Br
Ph
S
N
Ts
Me
7a
13C NMR 100 MHz, CDCl3
210 200 190 180 170 160 150 140 130 120 110 100 90 80 70 60 50 40 30 20 10 0 -10
f1 (ppm)

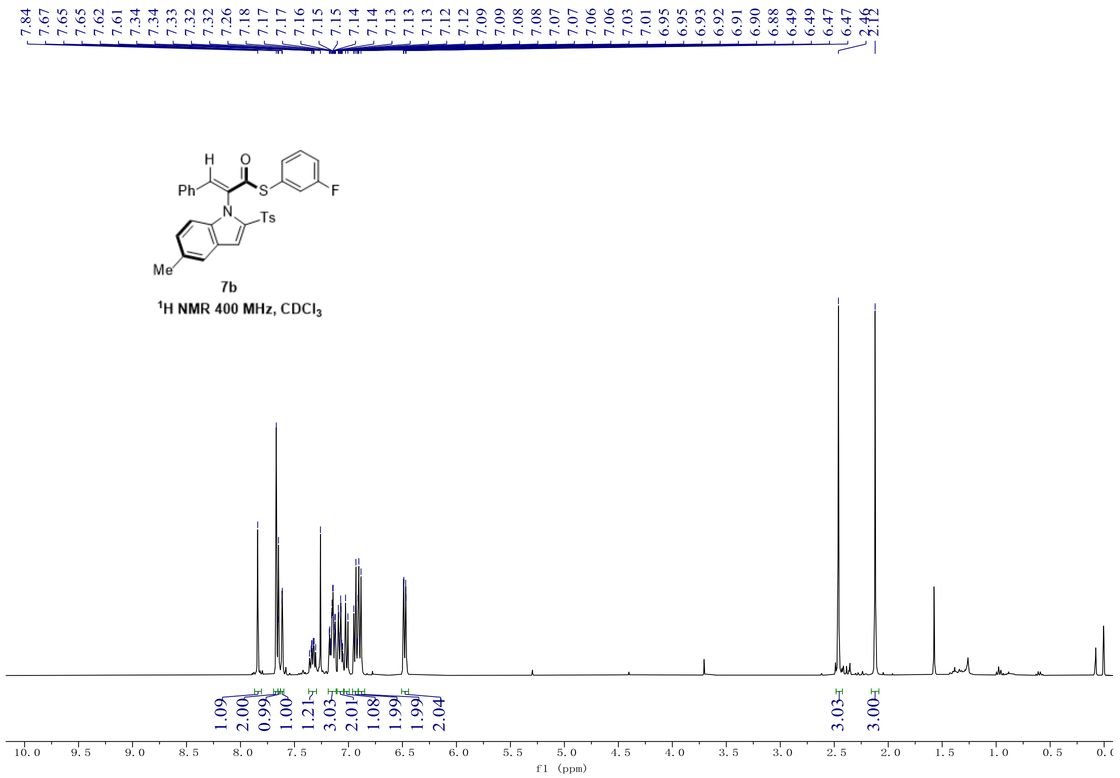
7b
¹H NMR 400 MHz, CDCl₃
f1 (ppm)

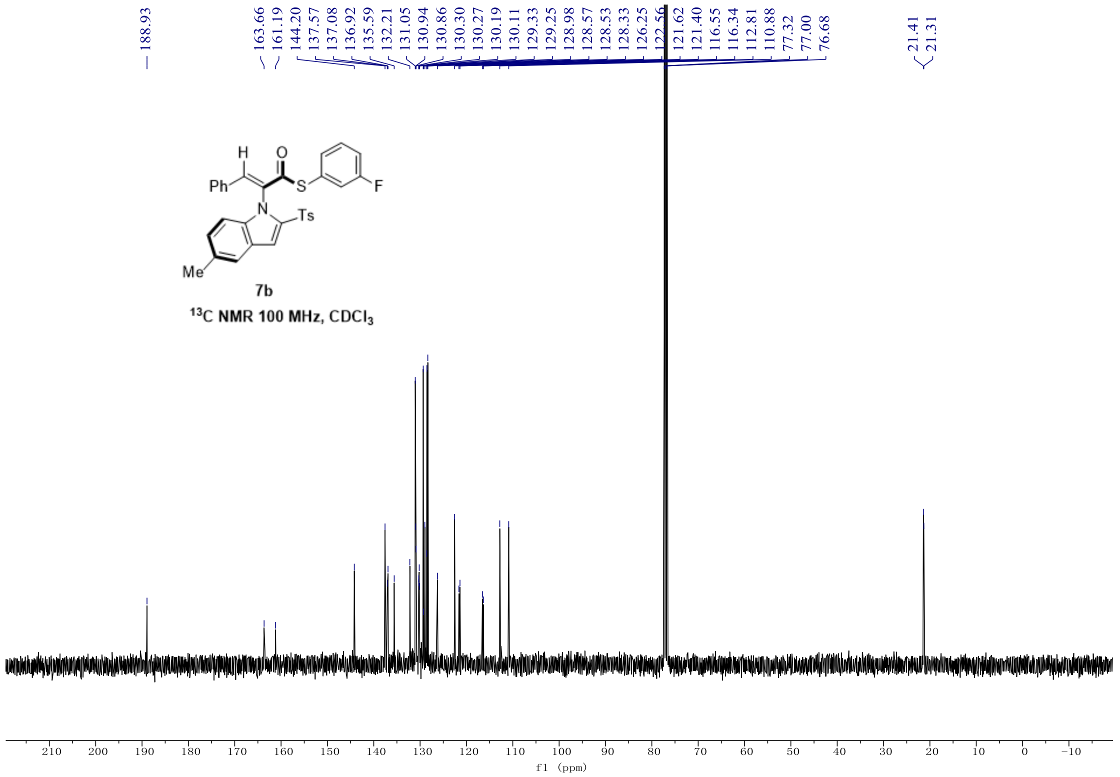
7b
¹³C NMR 100 MHz, CDCl₃
f1 (ppm)

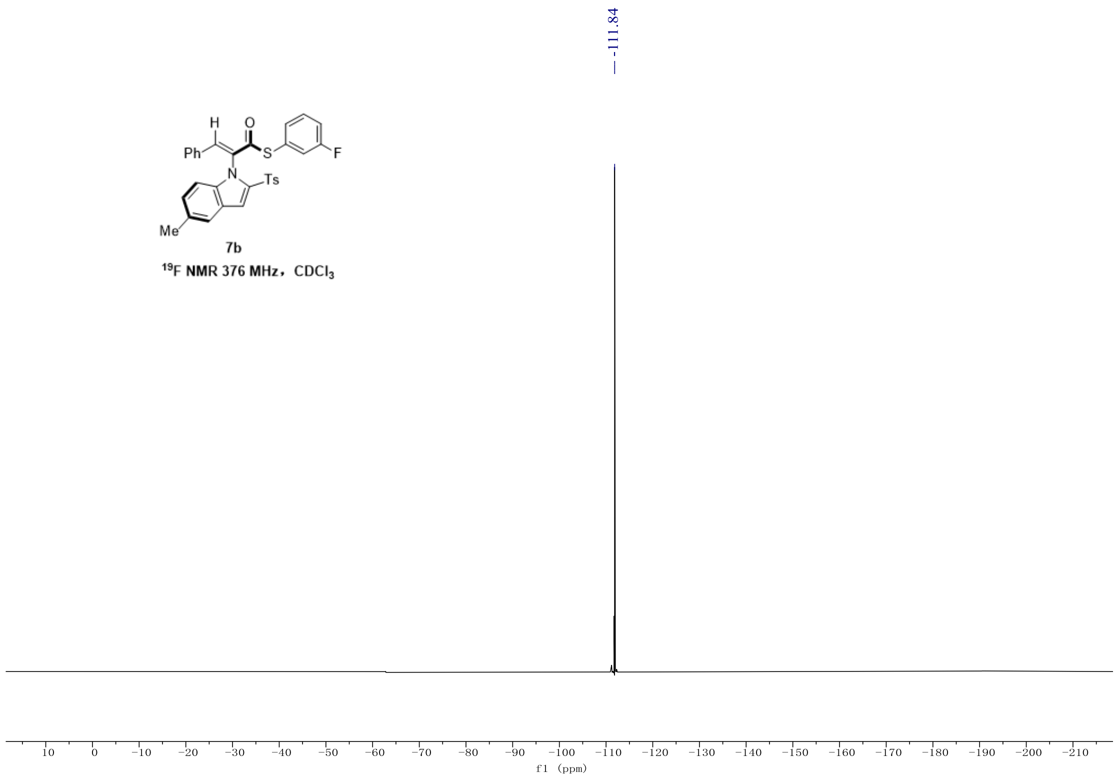
-111.84
Ph
H
O
S
F
N
Ts
Me
7b
19F NMR 376 MHz，CDCl3
10 0 −10 −20 −30 −40 −50 −60 −70 −80 −90 −100 −110 −120 −130 −140 −150 −160 −170 −180 −190 −200 −210
f1 (ppm)

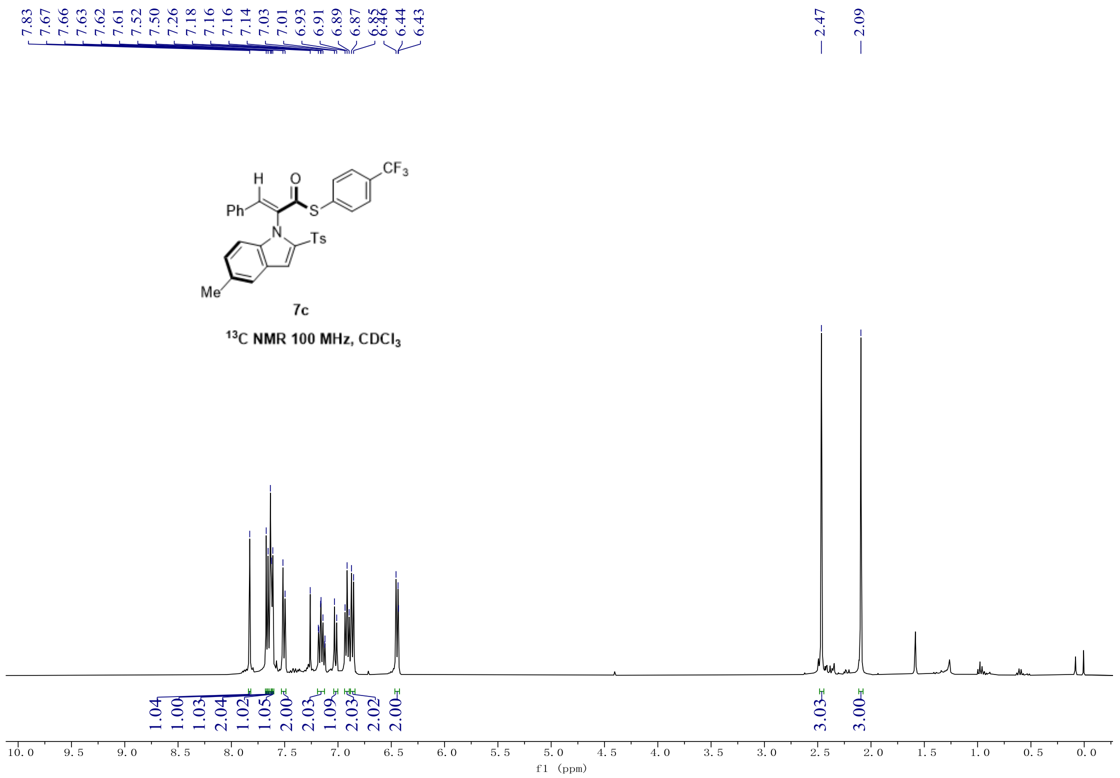
7.83
7.67
7.66
7.63
7.62
7.61
7.52
7.50
7.26
7.18
7.16
7.16
7.14
7.03
7.01
6.93
6.91
6.89
6.87
6.85
6.46
6.44
6.43
2.47
2.09
H
O
CF3
Ph
S
N
Ts
Me
7c
13C NMR 100 MHz, CDCl3
1.04
1.00
1.03
2.04
1.02
1.05
2.00
2.03
1.09
2.03
2.02
2.00
3.03
3.00
f1 (ppm)

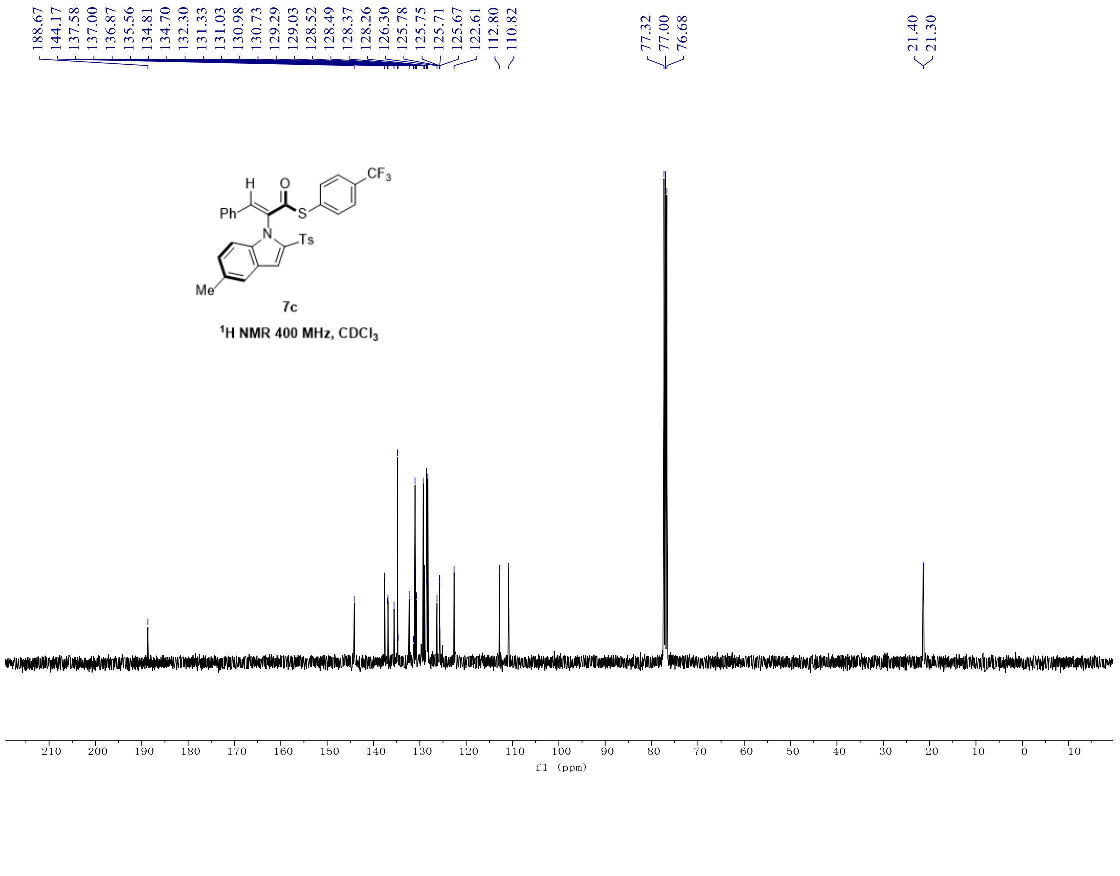
188.67
144.17
137.58
137.00
136.87
135.56
134.81
134.70
132.30
131.33
131.03
130.98
130.73
129.29
129.03
128.52
128.49
128.37
128.26
126.30
125.78
125.75
125.71
125.67
122.61
112.80
110.82
77.32
77.00
76.68
21.40
21.30
H
O
CF3
Ph
S
N
Ts
Me
7c
1H NMR 400 MHz, CDCl3
f1 (ppm)

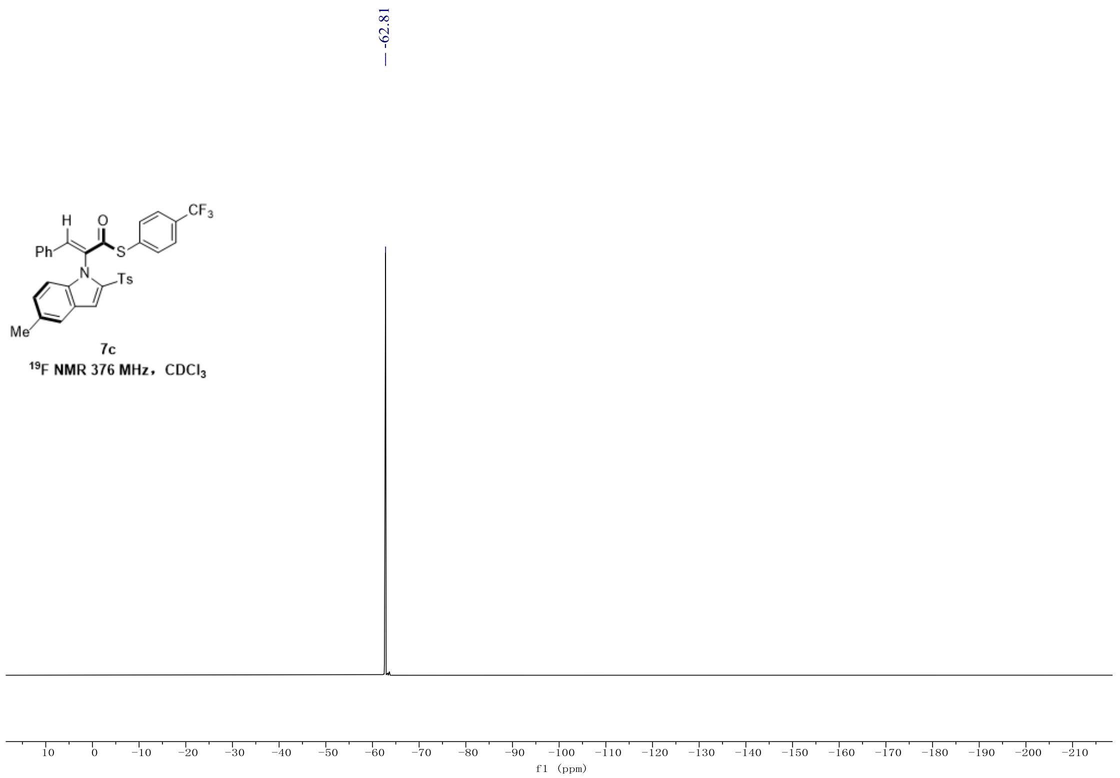
-62.81
CF3
H
O
Ph
S
N
Ts
Me
7c
19F NMR 376 MHz，CDCl3
10 0 −10 −20 −30 −40 −50 −60 −70 −80 −90 −100 −110 −120 −130 −140 −150 −160 −170 −180 −190 −200 −210
f1 (ppm)

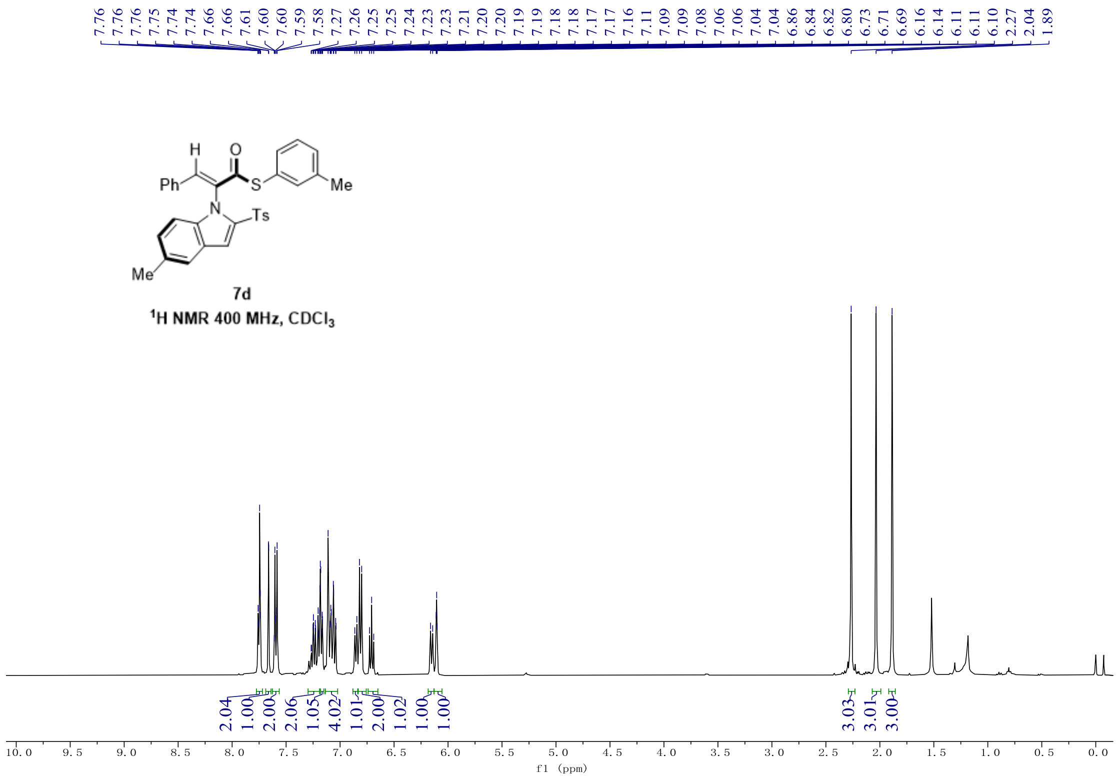
7.76 7.76 7.76 7.75 7.74 7.74 7.66 7.66 7.61 7.60 7.60 7.59 7.58 7.27 7.26 7.25 7.25 7.24 7.23 7.23 7.21 7.20 7.20 7.19 7.19 7.18 7.18 7.17 7.17 7.16 7.11 7.09 7.09 7.08 7.06 7.06 7.04 7.04 6.86 6.84 6.82 6.80 6.73 6.71 6.69 6.16 6.14 6.11 6.11 6.10 2.27 2.04 1.89
H
O
S
Me
Ph
N
Ts
Me
7d
1H NMR 400 MHz, CDCl3
2.04 1.00 2.00 2.06 1.05 4.02 1.01 2.00 1.02 1.00 1.00
3.03 3.01 3.00
10.0 9.5 9.0 8.5 8.0 7.5 7.0 6.5 6.0 5.5 5.0 4.5 4.0 3.5 3.0 2.5 2.0 1.5 1.0 0.5 0.0
f1 (ppm)

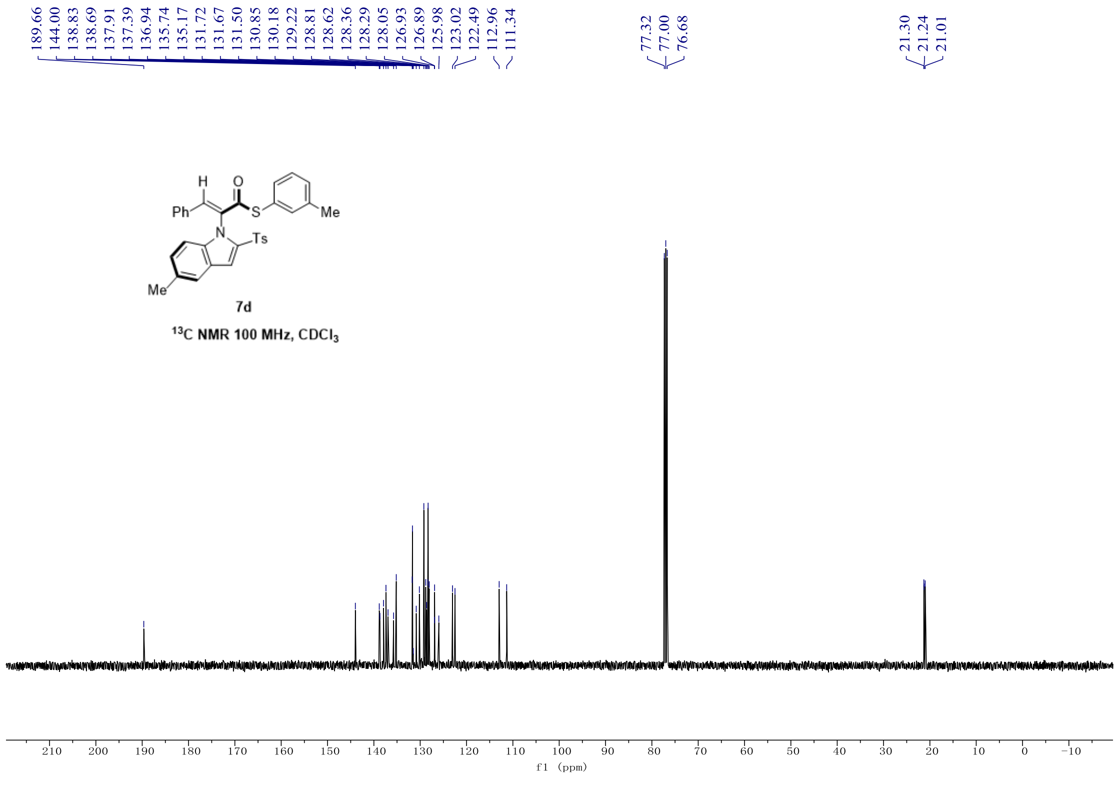
189.66 144.00 138.83 138.69 137.91 137.39 136.94 135.74 135.17 131.72 131.67 131.50 130.85 130.18 129.22 128.81 128.62 128.36 128.29 128.05 126.93 126.89 125.98 123.02 122.49 112.96 111.34
77.32 77.00 76.68
21.30 21.24 21.01
H
O
S
Me
Ph
N
Ts
Me
7d
13C NMR 100 MHz, CDCl3
210 200 190 180 170 160 150 140 130 120 110 100 90 80 70 60 50 40 30 20 10 0 -10
f1 (ppm)

HPLC conditions: Chiralpak IA-H, 30% ^{i}PrOH/Hx eluent, 0.7 mL/min, 250 nm

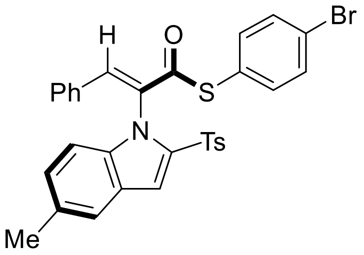

7a

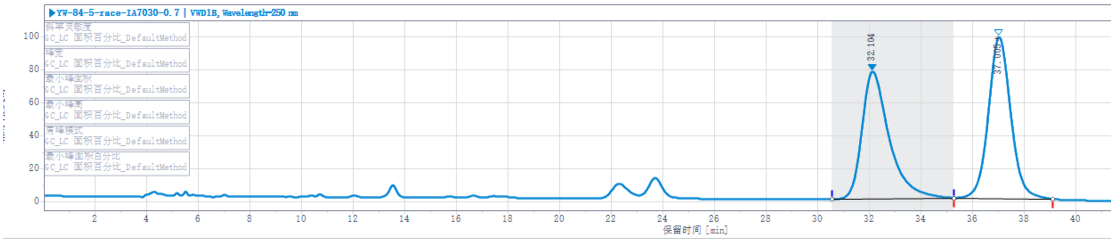

进样结果

峰 汇总

#	名称	RT (min)	峰面积 (mAU·s)	峰面积 %
1		32.104	629.225	49.051
2		37.005	653.571	50.949

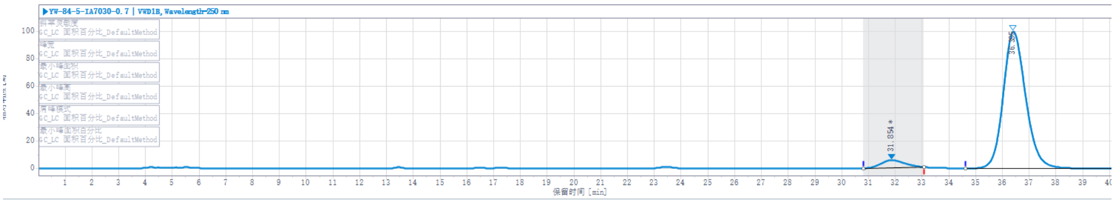

进样结果

峰 汇总

#	名称	RT (min)	峰面积 (mAU·s)	峰面积 %
1		31.854	231.084	5.494
2		36.395	3975.157	94.506

HPLC conditions: Chiralpak IA-H, 30% ^{i}PrOH/Hx eluent, 0.7 mL/min, 250 nm

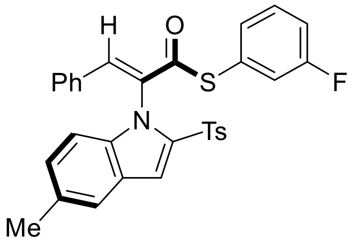

7b

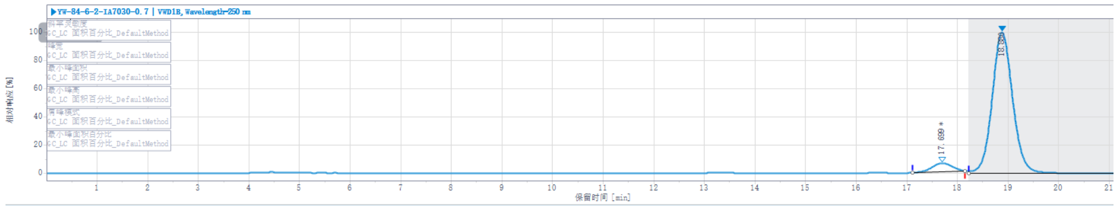

进样结果

峰 汇总

#	名称	RT (min)	峰面积 (mAU·s)	峰面积 %
1		17.699	141.159	5.568
2		18.880	2393.879	94.432

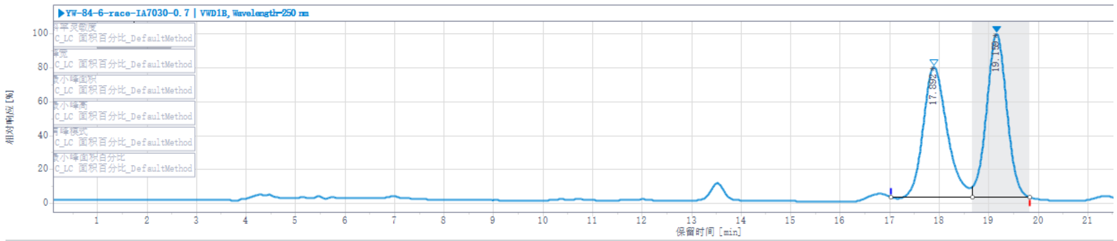

进样结果

峰 汇总

#	名称	RT (min)	峰面积 (mAU·s)	峰面积 %
1		17.892	316.415	48.700
2		19.159	333.310	51.300

HPLC conditions: Chiralpak IA-H, 30% ^{i}PrOH/Hx eluent, 0.7 mL/min, 250 nm

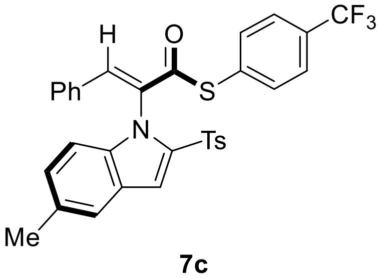

7c

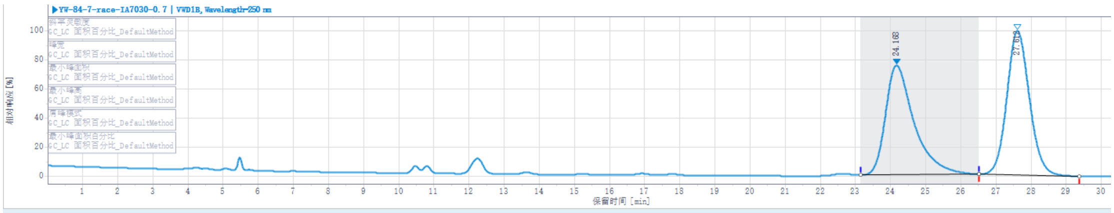

进样结果

峰 汇总

#	名称	RT (min)	峰面积 (mAU·s)	峰面积 %
1		24.168	868.801	49.119
2		27.612	899.968	50.881

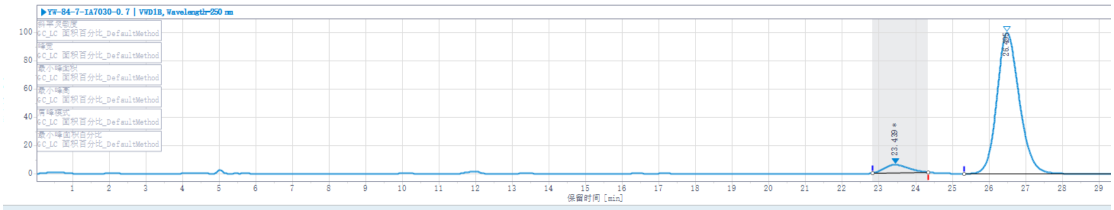

进样结果

峰 汇总

#	名称	RT (min)	峰面积 (mAU·s)	峰面积 %
1		23.439	184.718	5.652
2		26.495	3083.244	94.348

HPLC conditions: Chiralpak IA-H, 30% ^{i}PrOH/Hx eluent, 0.7 mL/min, 250 nm

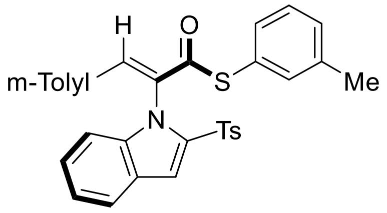

7d

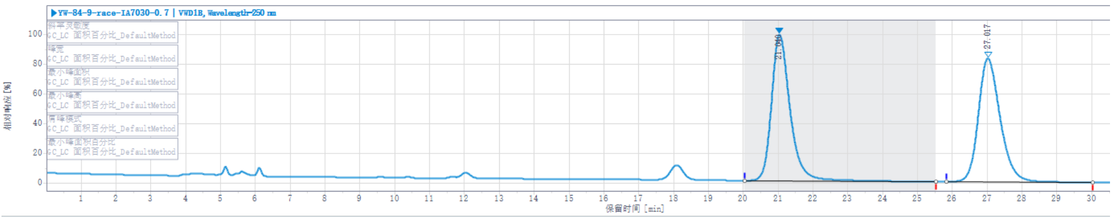

进样结果

峰 汇总

#	名称	RT (min)	峰面积 (mAU·s)	峰面积 %
1		21.040	959.067	50.285
2		27.017	948.186	49.715

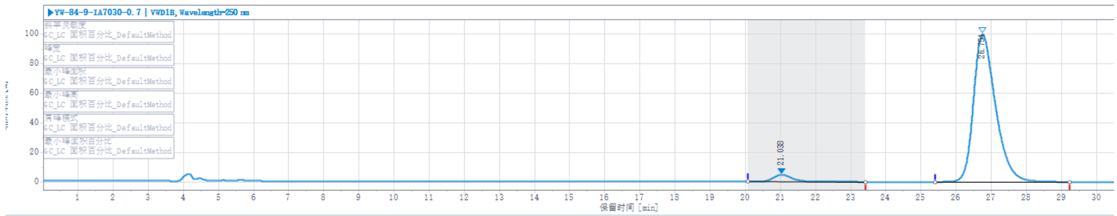

进样结果

峰 汇总

#	名称	RT (min)	峰面积 (mAU·s)	峰面积 %
1		21.038	128.381	4.001
2		26.754	3080.227	95.999

10. Gram scale reaction and Synthetic transformations

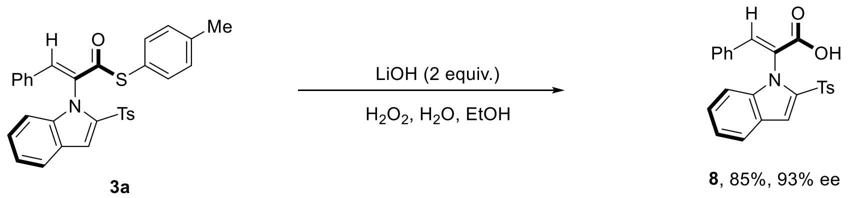

3a **8**, 85%, 93% ee

Procedure D : A solution of **3a** (0.10 mmol 1.0 equiv) in EtOH (1 mL), was added a previously prepared solution of LiOH in H_2O_2 (LiOH 4.0 equiv; 30% H_2O_2 65 uL; H_2O 130 uL). The mixture was stirred at rt for 12h, then quenched with HCl (1M). Afterwards extraction with CH_2Cl_2 and usual work up are performed. The residue purified by column chromatography with MeOH/DCM = 1/10 as the eluent to get **8**.

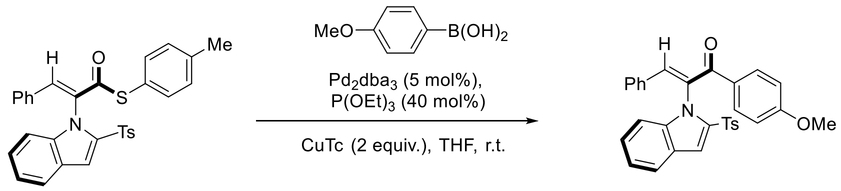

3a **9**, 81%, 80% ee

Procedure E : In an argon-filled glove box, to a Schlenk tube were added chiral thioesters **3a** (0.1 mmol), 4 Methoxyphenylboronic acid (0.15 mmol), Pd_2dba_3 (5 mol%), $P(OEt)_3$ (40 mol%), CuTc (2.0 equiv.), THF (2.0 mL). The reaction was maintained at room temperature and reacted overnight. The reaction was monitored by TLC. When the reaction was finished, the mixture was quenched by NH_4Cl (aq.) and extracted with EA (3x10 mL). The combined extracts are dried over Na_2SO_4 and concentrated. Purification by column chromatography on silica gel affords product chiral acetylenic ketone **9**.

11. Characterization and NMR spectra of products 8-9

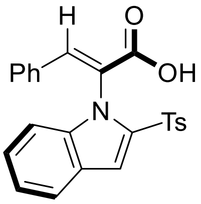

(Z)-3-phenyl-2-(2-tosyl-1H-indol-1-yl)acrylic acid (8) was synthesized by following Procedure D. The crude material was purified by column chromatography (SiO_2, petroleum ether: EtOAc = 5:1) to provide **8** as a yellow solid (36 mg, 85% yield).

^{1}H NMR (400 MHz, DMSO-d6) δ 7.88 (s, 1H), 7.78 (dd, J = 7.7, 1.2 Hz, 1H), 7.58 (d, J = 7.9 Hz, 2H), 7.53 (s, 1H), 7.24 – 7.11 (m, 2H), 7.07 (t, J = 7.4 Hz, 1H), 6.99 (t, J = 5.7 Hz, 3H), 6.87 (t, J = 7.6 Hz, 2H), 6.42 (d, J = 7.8 Hz, 2H), 2.12 (s, 3H).

^{13}C NMR (100 MHz, DMSO-d6) δ 166.3, 144.2, 138.5, 137.6, 135.8, 132.9, 129.8, 129.7, 129.6, 128.6, 128.1, 126.2, 125.9, 123.1, 121.9, 114.4, 113.6, 112.0, 111.2, 21.4.

HRMS: (ESI) m/z: $[M+H]^+$ Calcd for $C_{24}H_{20}NO_4S^+$ 418.1113; Found 418.1112.

Optical $[\alpha]_{25}^{D}$ = -45.4 ° (c = 0.25, CH_2Cl_2, 95% ee)

HPLC (IA-H, iPrOH/n-hexane = 30/70, flow rate = 0.7 mL/min, 250 nm) t_R = 28.0 min (major), 15.3 min (minor).

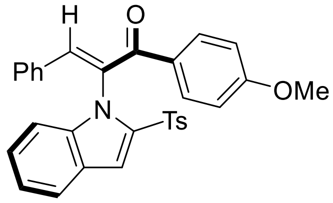

(Z)-1-(4-methoxyphenyl)-3-phenyl-2-(2-tosyl-1H-indol-1-yl)prop-2-en-1-one (9) was synthesized by following Procedure E. The crude material was purified by column chromatography (SiO_2, petroleum ether: EtOAc = 5:1) to provide **9** as a yellow solid (41 mg, 81% yield).

^{1}H NMR (400 MHz, $CDCl_3$) δ 7.92 – 7.83 (m, 2H), 7.75 – 7.71 (m, 1H), 7.69 (d, J = 1.7 Hz, 1H), 7.67 (d, J = 2.5 Hz, 2H), 7.53 (d, J = 0.9 Hz, 1H), 7.21 – 7.18 (m, 1H), 7.17 – 7.15 (m, 1H), 7.15 – 7.08 (m, 1H), 6.98 (d, J = 3.3 Hz, 2H), 6.97 – 6.95 (m, 3H), 6.94 (d, J = 1.9 Hz, 2H), 6.63 (dt, J = 8.5, 1.7 Hz, 2H), 3.88 (s, 3H), 2.20 (s, 3H).

^{13}C NMR (100 MHz, $CDCl_3$) δ 190.6, 162.9, 144.1, 141.3, 138.5, 137.6, 135.0, 131.9, 131.9, 131.8, 130.4, 130.1, 129.6, 129.3, 128.5, 127.8, 126.5, 126.2, 122.9, 122.0, 113.6, 112.8, 111.5, 55.4, 21.4.

HRMS: (ESI) m/z: $[M+H]^+$ Calcd for $C_{31}H_{26}NO_4S^+$ 508.1583; Found 508.1584.

Optical $[\alpha]_{25}^{D}$ = -5.3 ° (c = 0.25, CH_2Cl_2, 80% ee)

HPLC (IA-H, iPrOH/n-hexane = 30/70, flow rate = 0.7 mL/min, 250 nm) t_R = 38.4 min (major), 50.6 min (minor).

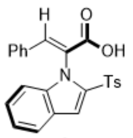

8

^{13}C NMR 100 MHz, DMSO-d6

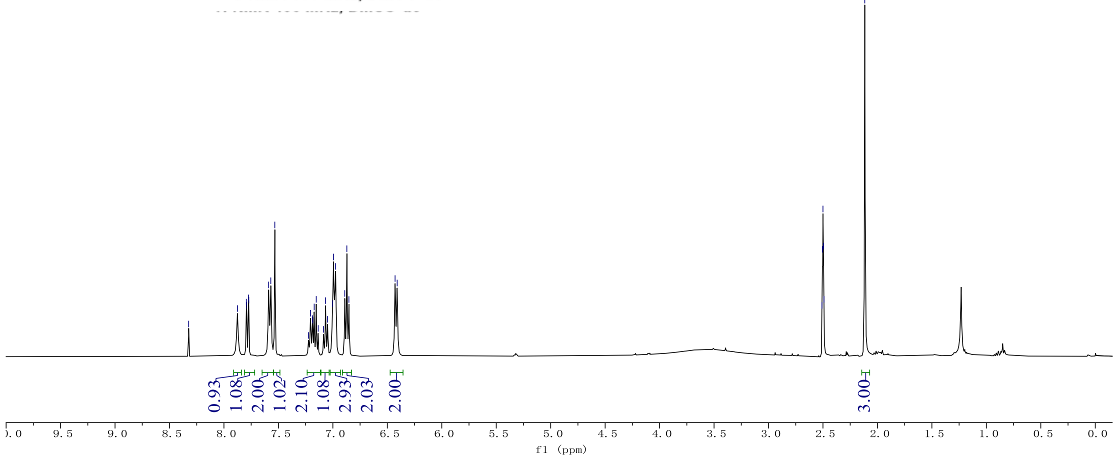

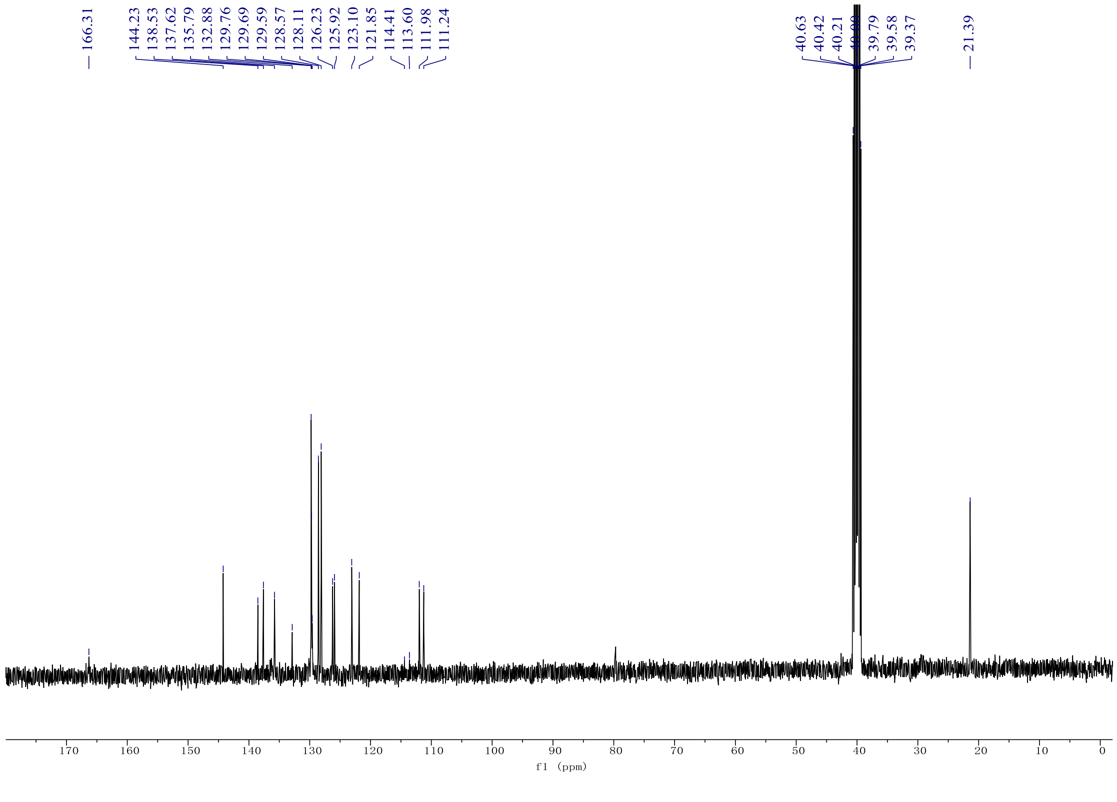

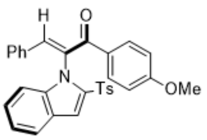

9

^{1}H NMR 400 MHz, $CDCl_3$

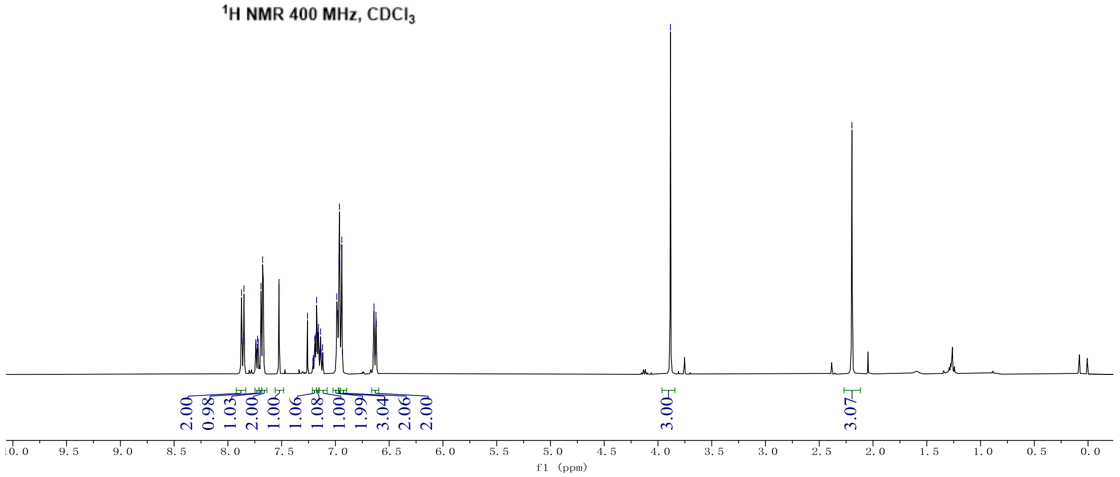

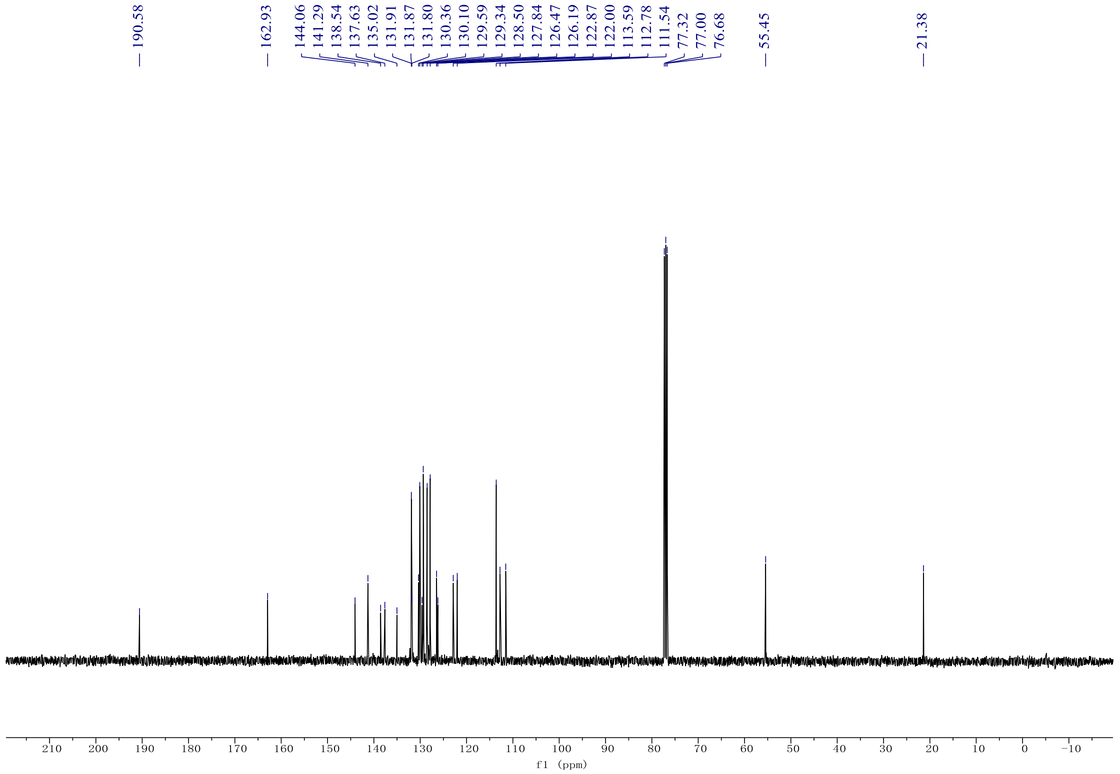

HPLC conditions: Chiralpak AD-H, 30% ^{i}PrOH/Hx eluent, 0.8 mL/min, 250 nm

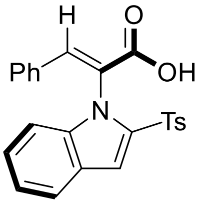

8

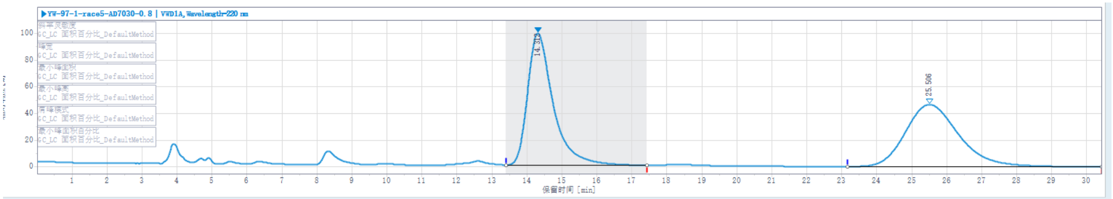

进样结果

峰 汇总

#	名称	RT (min)	峰面积 (mAU·s)	峰面积 %
1		14.312	1337.572	50.225
2		25.506	1325.601	49.775

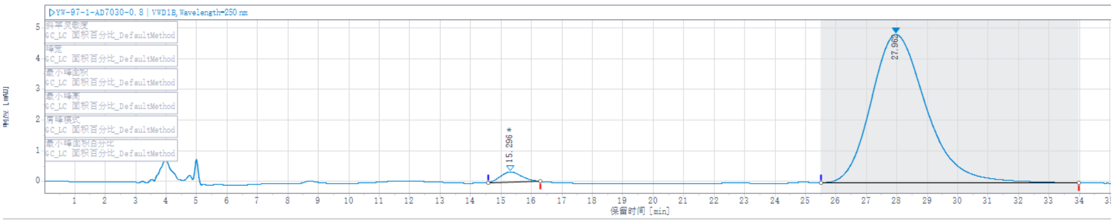

进样结果

峰 汇总

#	名称	RT (min)	峰面积 (mAU·s)	峰面积 %
1		15.296	15.851	2.554
2		27.962	604.770	97.446

HPLC conditions: Chiralpak IA-H, 30% ^{i}PrOH/Hx eluent, 0.7 mL/min, 250 nm

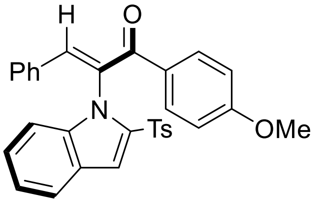

9

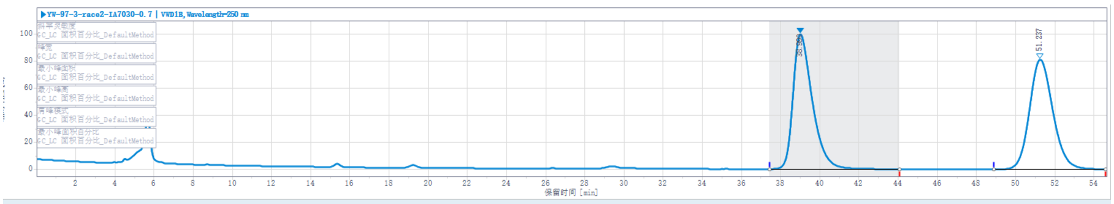

进样结果

峰 汇总

#	名称	RT (min)	峰面积 (mAU·s)	峰面积 %
1		38.990	1889.902	50.271
2		51.237	1869.524	49.729

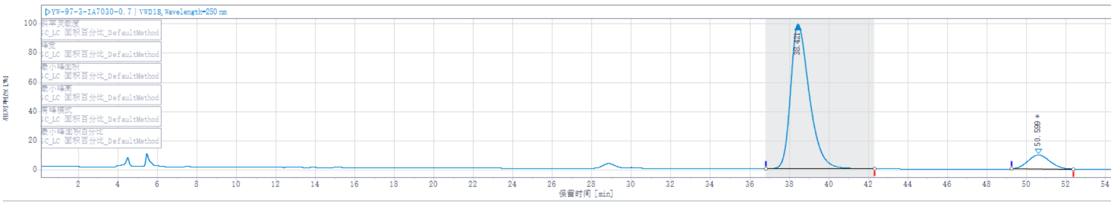

进样结果

峰 汇总

#	名称	RT (min)	峰面积 (mAU·s)	峰面积 %
1		38.421	1320.516	89.891
2		50.599	148.511	10.109

12. Control experiments

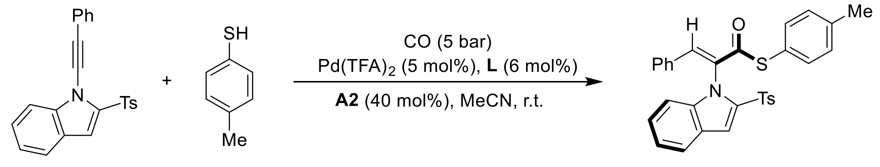

1a **2a** **3a**, 0%

To a mixture of **1a** (0.1 mmol), **2a** (0.15 mmol), $Pd(TFA)_2$ (0.005 mmol), L**7** (0.006 mmol) and **A2** (0.04 mmol) was added MeCN (0.5 mL). After flushing the autoclave three times with CO, a pressure of 5 bar of CO was adjusted at ambient temperature. Then, the reaction was performed for 24 h at roomtempure. After the reaction was complete, the pressure of autoclave was released carefully. The mixture was diluted with EtOAc (5 mL) and extracted with EtOAc (5 mL x 3). The combined organic layers were washed with brine (10 mL), dried (Na_2SO_4), and concentrated. Further purification by flash column chromatography on silica gel (petroleum ether: EtOAc) provided **3a**.

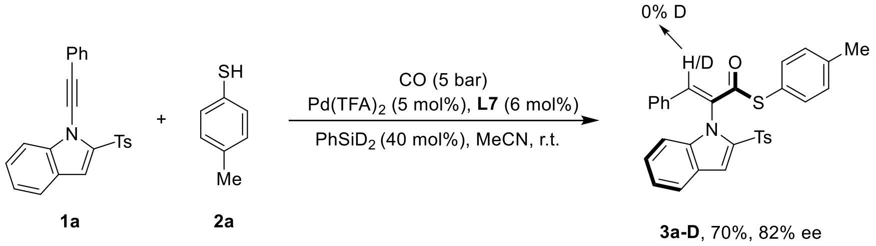

To a mixture of **1a** (0.1 mmol), **2a** (0.15 mmol), Pd(TFA)$_2$ (0.005 mmol), L**7** (0.006 mmol) and PhSiD$_2$ (0.04 mmol) was added MeCN (0.5 mL). After flushing the autoclave three times with CO, a pressure of 5 bar of CO was adjusted at ambient temperature. Then, the reaction was performed for 24 h at roomtempure. After the reaction was complete, the pressure of autoclave was released carefully. The mixture was diluted with EtOAc (5 mL) and extracted with EtOAc (5 mL x 3). The combined organic layers were washed with brine (10 mL), dried (Na$_2$SO$_4$), and concentrated. Further purification by flash column chromatography on silica gel (petroleum ether: EtOAc) provided **3a-D**.

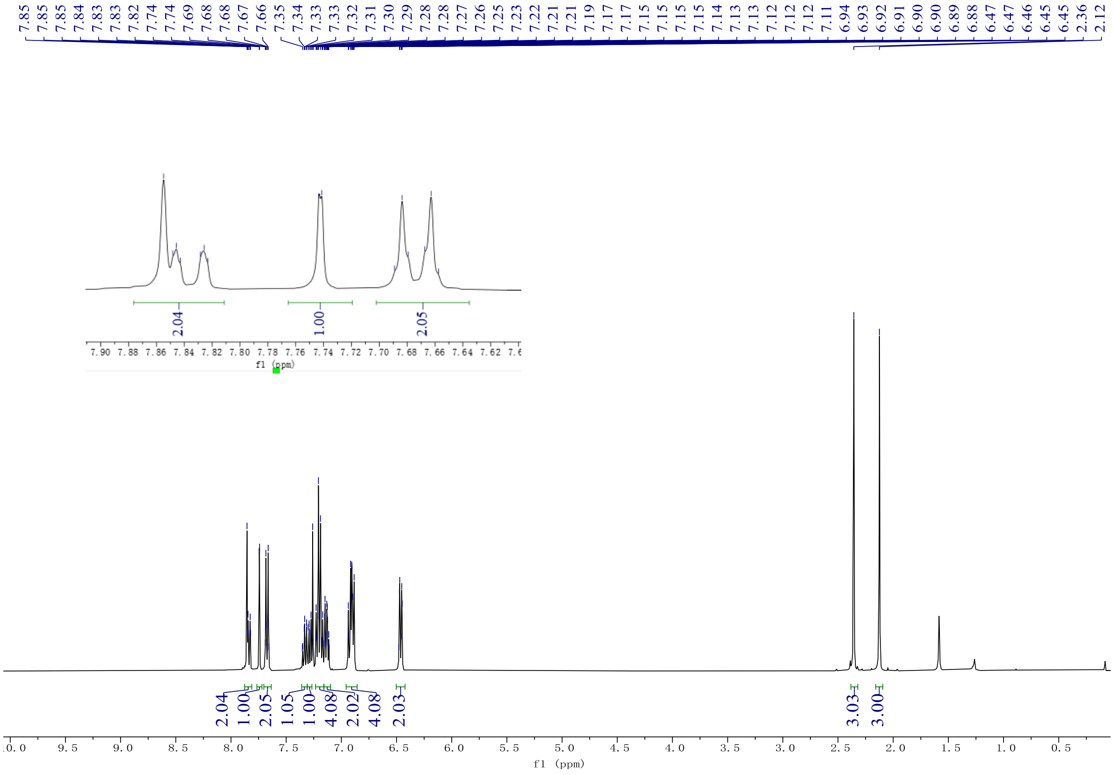

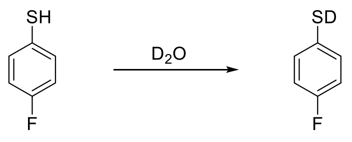

1ac **1ac-D**

1a (1.0 mL, 9.78 mmol) was dissolved in D_2O (1.8 mL, 97.8 mmol), the mixture was vigorously stirred for 2 hours at room temperature. The clear solution was concentrated in vacuo and resulted in product with 85% D (**1ac-D**) incorporation which was determined by 1H NMR. 1H NMR (400 MHz, $CHCl_3$) δ 7.29 – 7.22 (m, 2H), 6.97 – 6.89 (m, 2H).

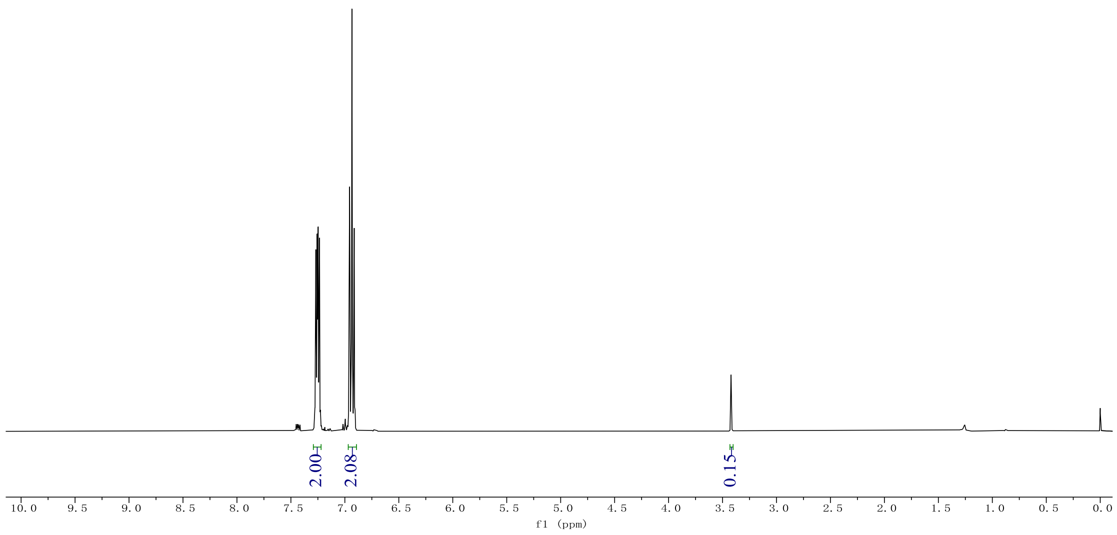

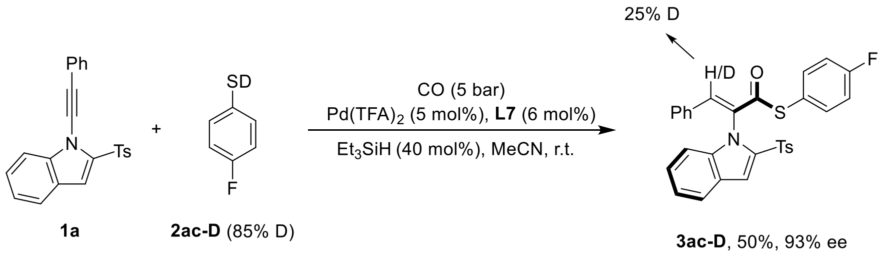

To a mixture of **1a** (0.1 mmol), **2ac-D** (0.15 mmol), Pd(TFA)$_2$ (0.005 mmol), L**7** (0.006 mmol) and Et$_3$SiH(0.04 mmol) was added MeCN (0.5 mL). After flushing the autoclave three times with CO, a pressure of 5 bar of CO was adjusted at ambient temperature. Then, the reaction was performed for 24 h at roomtempure. After the reaction was complete, the pressure of autoclave was released carefully. The mixture was diluted with EtOAc (5 mL) and extracted with EtOAc (5 mL x 3). The combined organic layers were washed with brine (10 mL), dried (Na$_2$SO$_4$), and concentrated. Further purification by flash column chromatography on silica gel (petroleum ether: EtOAc) provided **3ac-D**.

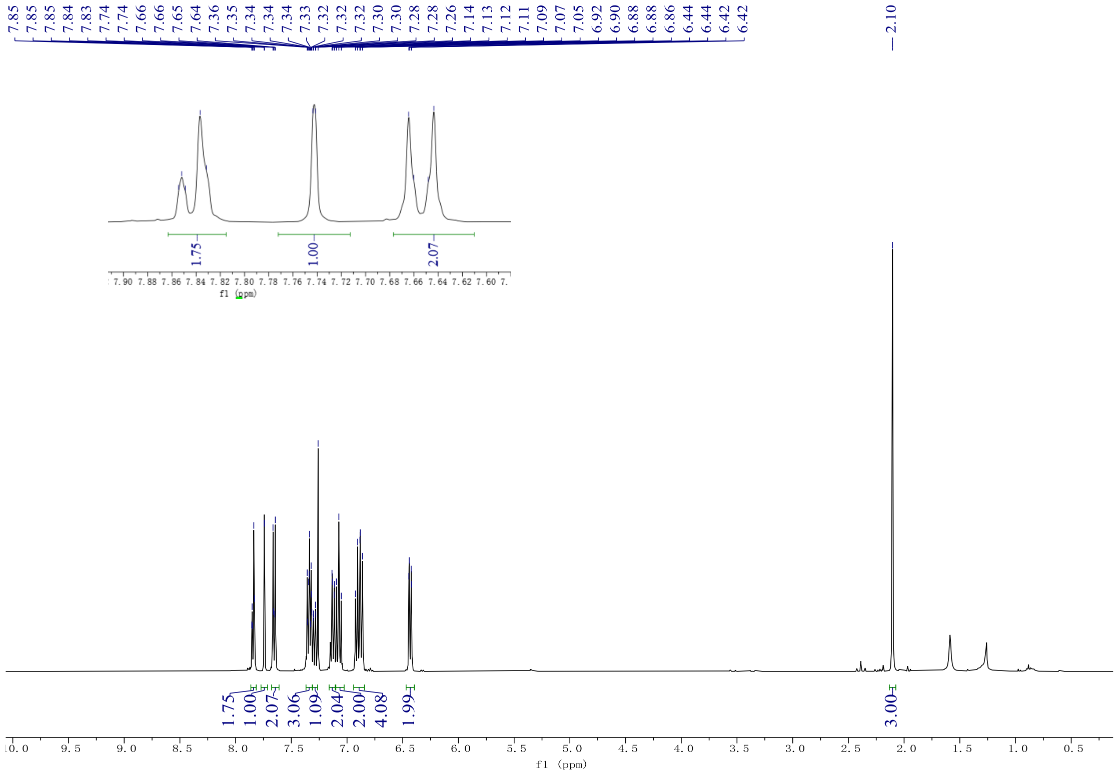

13. Crystallographic data for 3a

checkCIF/PLATON report

Structure factors have been supplied for datablock(s) YW-76-1_auto

THIS REPORT IS FOR GUIDANCE ONLY. IF USED AS PART OF A REVIEW PROCEDURE FOR PUBLICATION, IT SHOULD NOT REPLACE THE EXPERTISE OF AN EXPERIENCED CRYSTALLOGRAPHIC REFEREE.

No syntax errors found.   CIF dictionary   Interpreting this report

Datablock: YW-76-1_auto

```
Bond precision:   C-C = 0.0029 A          Wavelength=1.54178

Cell:            a=10.5386(1)       b=13.3450(1)      c=20.1212(2)
                 alpha=84.619(1)    beta=89.541(1)    gamma=76.074(1)
Temperature:     293 K

                  Calculated                   Reported
Volume            2734.21(4)                   2734.21(4)
Space group       P -1                         P -1
Hall group        -P 1                         -P 1
Moiety formula    C31 H25 N O3 S2              ?
Sum formula       C31 H25 N O3 S2              C31 H25 N O3 S2
Mr                523.64                       523.64
Dx,g cm-3         1.272                        1.272
Z                 4                            4
Mu (mm-1)         2.023                        2.023
F000              1096.0                       1096.0
F000'             1101.51
h,k,lmax          13,16,25                     13,16,25
Nref              11458                        11079
Tmin,Tmax         0.964,0.980                  0.631,1.000
Tmin'             0.960

Correction method= # Reported T Limits: Tmin=0.631 Tmax=1.000
AbsCorr = MULTI-SCAN

Data completeness= 0.967           Theta(max)= 76.258

                                                  wR2(reflections)=
R(reflections)= 0.0489( 9967)                     0.1416( 11079)

S = 1.024                  Npar= 669
```

```
The following ALERTS were generated. Each ALERT has the format
       test-name_ALERT_alert-type_alert-level.
Click on the hyperlinks for more details of the test.
```

Alert level C

```
PLAT241_ALERT_2_C High   'MainMol' Ueq as Compared to Neighbors of        S1 Check
PLAT242_ALERT_2_C Low    'MainMol' Ueq as Compared to Neighbors of        C4 Check
PLAT906_ALERT_3_C Large K Value in the Analysis of Variance ......     2.098 Check
PLAT911_ALERT_3_C Missing FCF Refl Between Thmin & STh/L=    0.600        16 Report
               -1  3  0,  -2  2  1,   2  2  1,   9 13  3,   0 -1  4,   1-14  7,
               12  2  7,   4-12  8,  10 -4  9,  10 -3  9,  10 -3 10,  10 -2 10,
                4 15 10,  10 -2 11,  -8 -6 17,  -5  4 21,
```

Alert level G

```
PLAT154_ALERT_1_G The s.u.'s on the Cell Angles are Equal ..(Note)     0.001 Degree
PLAT199_ALERT_1_G Reported _cell_measurement_temperature ..... (K)       293 Check
PLAT200_ALERT_1_G Reported   _diffrn_ambient_temperature ..... (K)       293 Check
PLAT380_ALERT_4_G Incorrectly? Oriented X(sp2)-Methyl Moiety .....        C7 Check
PLAT380_ALERT_4_G Incorrectly? Oriented X(sp2)-Methyl Moiety .....       C31 Check
PLAT883_ALERT_1_G Absent Datum for _atom_sites_solution_primary ..    Please Do !
PLAT912_ALERT_4_G Missing # of FCF Reflections Above STh/L=  0.600       363 Note
PLAT913_ALERT_3_G Missing # of Very Strong Reflections in FCF ....         3 Note
               -1  3  0,   2  2  1,   0 -1  4,
PLAT941_ALERT_3_G Average HKL Measurement Multiplicity ...........       3.3 Low
PLAT969_ALERT_5_G The 'Henn et al.' R-Factor-gap value ...........     4.062 Note
              Predicted wR2: Based on SigI**2  3.49 or SHELX Weight 13.82
PLAT978_ALERT_2_G Number C-C Bonds with Positive Residual Density.         4 Info
```

```
   0 ALERT level A = Most likely a serious problem - resolve or explain
   0 ALERT level B = A potentially serious problem, consider carefully
   4 ALERT level C = Check. Ensure it is not caused by an omission or oversight
  11 ALERT level G = General information/check it is not something unexpected

   4 ALERT type 1 CIF construction/syntax error, inconsistent or missing data
   3 ALERT type 2 Indicator that the structure model may be wrong or deficient
   4 ALERT type 3 Indicator that the structure quality may be low
   3 ALERT type 4 Improvement, methodology, query or suggestion
   1 ALERT type 5 Informative message, check
```

Datablock: YW-76-1_auto - ellipsoid plot

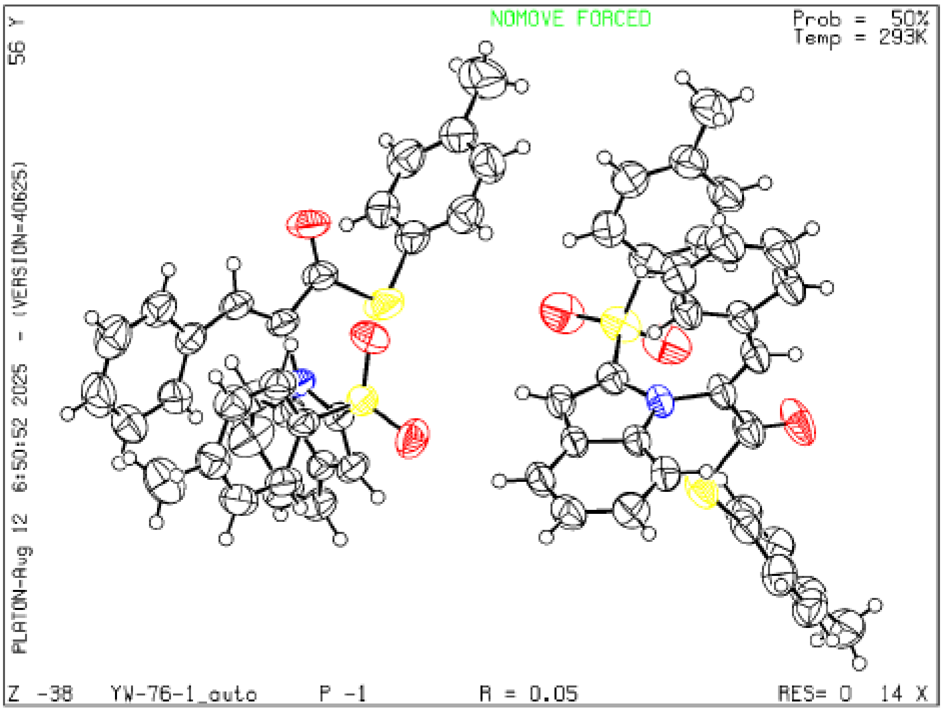

14. Rotational barriers

The enantiomerisation barrier, corresponding to the barrier to rotation for the following atropisomers, was obtained by kinetic of racemisation of an enantiomer.The slope of the firstorder kinetic line gives the racemisation constant (kracemisation = 2×kenantiomerisation). Eyring equation gives the enantiomerisation barrier (ΔG‡enaniomerization) from enantiomerisation constant (kenantiomerisation), R = 8.31451 $J.K^{-1}\ mol^{-1}$, h =6.62608×10^{-34} Js and k_B =1.38066×10^{-23} J/K. Reactions were conducted at 5 mg/mL concentration. Enantiomeric excess values were determined by chiral HPLC.

$$\Delta G^{\ddagger}\text{enaniomerization} = RT \times \ln \frac{\mathrm{k}_B \times T}{\mathrm{h} \times k_{enantiomerisation}}$$

Racemization of **3a** in toluene at 100 °C

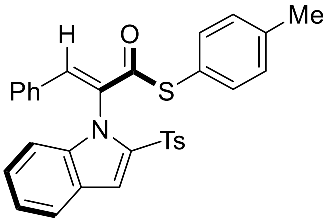

3a

ΔG‡= 30.7 kcal/mol

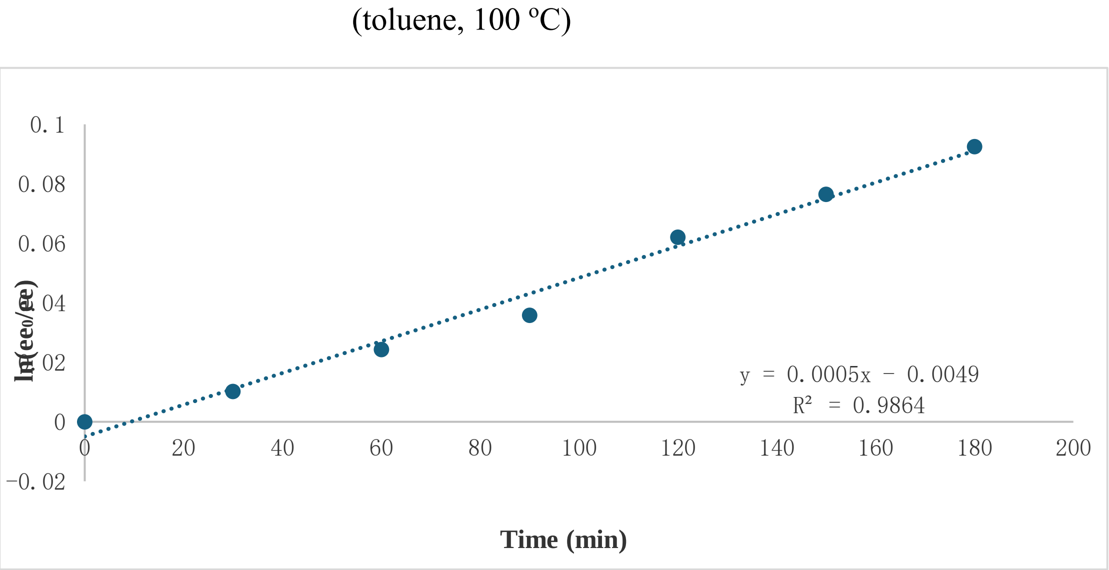

Kracemisation = 0.0005 min^{-1}

Kenantiomerisation = 0.00025 min^{-1}

ΔG‡enaniomerization= 30.7 kcal/mol

Time (min)	ee (%)	ln(ee$_0$/ee)
0	85.311	0
30	84.438	0.0189
60	83.254	0.023
90	82.290	0.0357
120	80.162	0.0506
150	79.016	0.0624
180	77.758	

Racemization of **3i** in toluene at 120 °C

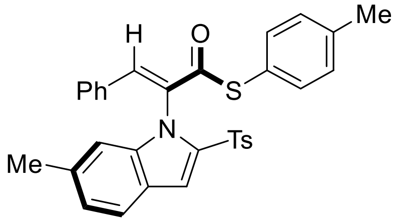

3i

ΔG‡= 30.8 kcal/mol

(toluene, 120 °C)

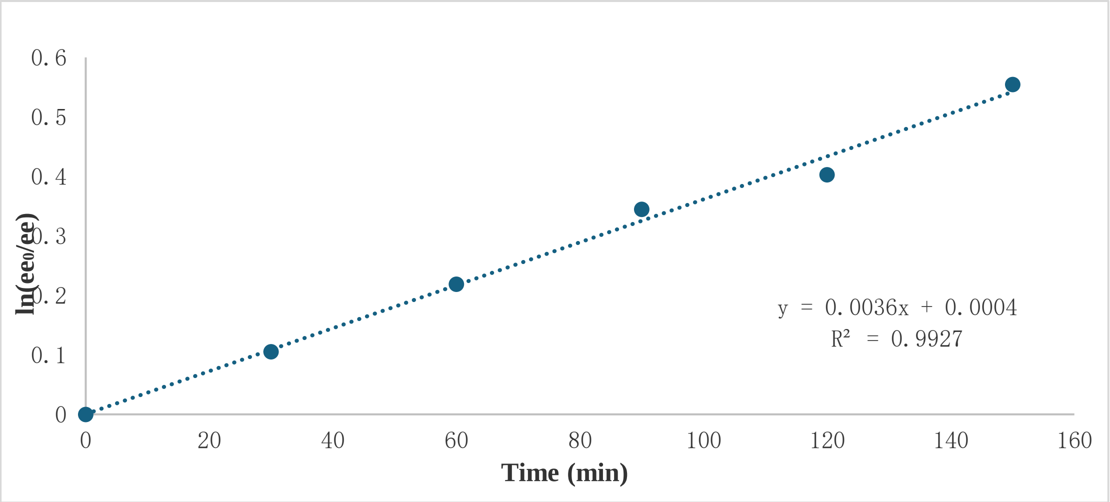

Kracemisation = 0.0036 min^{-1}

Kenantiomerisation = 0.0018 min^{-1}

ΔG‡enaniomerization= 30.8 kcal/mol

Time (min)	ee (%)	$\ln(ee_0/ee)$
0	71.3	0
30	64.146	0.0593
60	57.270	0.3905
90	50.496	0.4091
120	47.662	0.5094
150	42.368	0.6731

Racemization of **3m** in toluene at 120 °C

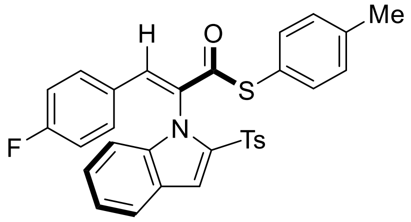

3m

ΔG‡= 31.5 kcal/mol

(toluene, 120 °C)

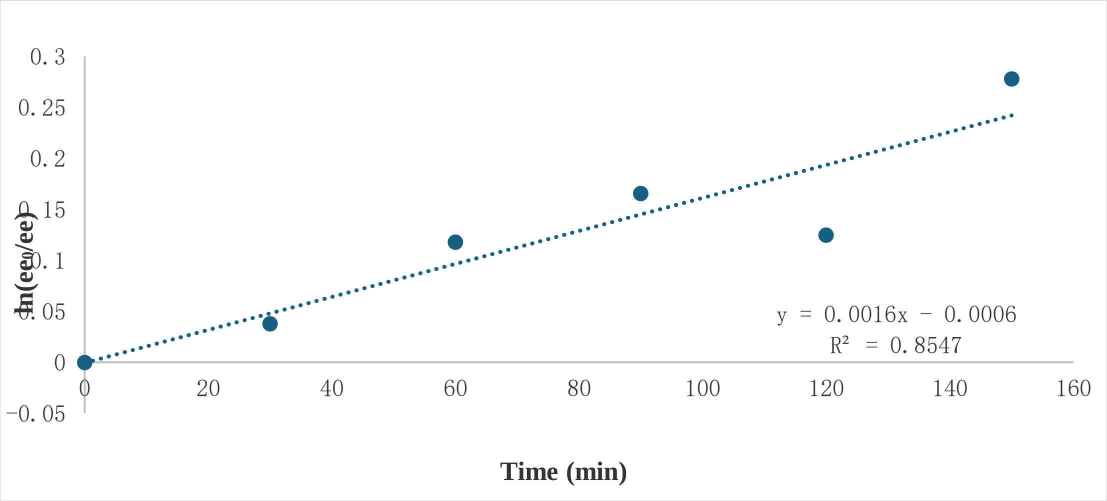

Kracemisation = 0.0016 min^{-1}

Kenantiomerisation = 0.0008 min^{-1}

ΔG‡enaniomerization= 31.5 kcal/mol

Time (min)	ee (%)	$\ln(ee_0/ee)$
0	40.103	0
15	32.946	0.194
30	31.476	0.2397
45	29.806	0.2942
60	29.274	0.3122
75	28.536	0.4555
90	27.942	0.4765
105	27.105	0.5069
120	27.105	0.5312

15. Enantiomeric conversion half-life calculation

The Eyring Equation relates the activation free energy and rate constant:

$$k = \kappa \frac{k_B T}{h} e^{-\frac{\Delta G^{\neq}}{RT}} \quad (1)$$

(1) In this equation, $\Delta G^{\ddagger}$ is the Gibbs energy of activation, κ is the transmission coefficient, k_B is Boltzmann's constant, and h is Planck's constant. The transmission coefficient is often assumed to be equal to one as it reflects what fraction of the flux through the transition state proceeds to the product without recrossing the transition state.

The epimerization of atropoisomer is a first order reaction, which makes the half-life only relates to the reaction rate constant:

$$t_{1/2} = \ln(2)/k \quad (2)$$

The $\Delta G^{\ddagger}$ of **3a** at 298 K was estimate 30.7 kcal/mol. Based on Equations 1 and 2, we calculated the half-life of **3a**, $t_{1/2}$ = 116 years.
The $\Delta G^{\ddagger}$ of **3m** at 298 K was estimate 31.5 kcal/mol. Based on Equations 1 and 2, we calculated the half-life of **3m**, $t_{1/2}$ = 446 years.
The $\Delta G^{\ddagger}$ of **3i** at 298 K was estimate 30.8 kcal/mol. Based on Equations 1 and 2, we calculated the half-life of **3i**, $t_{1/2}$ = 137 years.

16. References

[1] L.-W. Zhan, C.-J. Lu, J. Feng, R.-R. Liu, *Angew. Chem. Int. Ed.* **2023**, *62*, e202312930.
